# Multiple Genes with Potential Tumor Suppressive Activity Are Present on Chromosome 10q Loss in Neuroblastoma and Are Associated with Poor Prognosis

**DOI:** 10.3390/cancers15072035

**Published:** 2023-03-29

**Authors:** Marzia Ognibene, Patrizia De Marco, Loredana Amoroso, Davide Cangelosi, Federico Zara, Stefano Parodi, Annalisa Pezzolo

**Affiliations:** 1 U.O.C. Genetica Medica, IRCCS Istituto Giannina Gaslini, 16147 Genova, Italy; 2 U.O.C. Oncologia Pediatrica, IRCCS Istituto Giannina Gaslini, 16147 Genova, Italy; 3 Unità di Bioinformatica Clinica, IRCCS Istituto Giannina Gaslini, 16147 Genova, Italy; 4 Direzione Scientifica, IRCCS Giannina Gaslini, 16147 Genova, Italy; 5 IRCCS Istituto Giannina Gaslini, 16147 Genova, Italy

**Keywords:** neuroblastoma, chromosome 10q loss, tumor suppressor genes, *CCSER2*

## Abstract

**Simple Summary:**

Specific recurrent segmental aberrations (SCA) have been associated with poor survival in neuroblastoma (NB). The effect of many other not recurrent SCA has not been clarified yet. Here we examined the role of the loss of chromosome 10q in a cohort of 260 NB primary tumors, observing a lower survival rate for the patients expressing this rare SCA. We identified a cluster of 75 genes in the long arm of chromosome 10, whose deletion was associated with poorer survival. Six genes, and in particular *CCSER2*, appeared to be good candidates to play a role as tumor suppressor genes in NB and possible targets for future therapeutic interventions.

**Abstract:**

Neuroblastoma (NB) is a tumor affecting the peripheral sympathetic nervous system that substantially contributes to childhood cancer mortality. Despite recent advances in understanding the complexity of NB, the mechanisms determining its progression are still largely unknown. Some recurrent segmental chromosome aberrations (SCA) have been associated with poor survival. However, the prognostic role of most SCA has not yet been investigated. We examined a cohort of 260 NB primary tumors at disease onset for the loss of chromosome 10q, by array-comparative genomic hybridization (a-CGH) and Single Nucleotide Polymorphism (SNP) array and we found that 26 showed 10q loss, while the others 234 displayed different SCA. We observed a lower event-free survival for NB patients displaying 10q loss compared to patients with tumors carrying other SCA. Furthermore, analyzing the region of 10q loss, we identified a cluster of 75 deleted genes associated with poorer outcome. Low expression of six of these genes, above all *CCSER2*, was significantly correlated to worse survival using in silico data from 786 NB patients. These potential tumor suppressor genes can be partly responsible for the poor prognosis of NB patients with 10q loss.

## 1. Introduction

Neuroblastoma (NB) is a pediatric tumor originating from immature nerve cells of neural crest characterized by genetic mutations and defective differentiation occurring during early development, representing about 7–8% of children’s cancers [1,2,3]. The age at diagnosis is 18 months on average and NB occurs before 6–8 years of life in 90% of cases. In half of the patients, NB is diagnosed in its metastatic form and determines 15% of deaths in pediatric oncology [4,5]. Current therapies, although articulated and including various approaches, are still not sufficient to definitively eradicate it in a large percentage of cases [6]. Clinical and biological factors, such as age at diagnosis, disease stage, tumor histo-pathology, DNA ploidy, and chromosomal abnormalities define the prognosis and specific treatment of NB patients [7,8,9,10,11,12]. Genome-wide association, high-throughput sequencing, and microarray-based studies have identified hereditable and somatically acquired genetic [7,8] and chromosomal changes that characterize NB [9,10,11,12]. Among these, only a few recurrent segmental chromosome aberrations (SCA) have been demonstrated to help discriminate between low and high-risk NB patients with fatal outcome [9,10,11,12]. The identification of chromosomal variations in NB samples gave information relevant to the diagnosis and disease prognosis. NB tumors with gain or loss of entire chromosomes are generally associated with a good prognosis, while tumors with SCA have poor outcome [13,14,15,16,17,18,19,20]. Recurrent SCA in NB involve losses of chromosome 1p, 3p, 4p, 6q, 11q, and 19p, and gains of 1q, 2p, 7q, and 17q that together with amplification of *MYCN* oncogene, specific amplifications on 2p and 12q and loss of whole chromosome X, are all markers of high-risk disease [13,14,15,16,17,18,19,20]. It is important to underline that amplification of *MYCN* and specific genes on chromosomes 2p and 12q [12,14,15], as well as loss of chromosomes 6q and 11q [12,13,17] are associated with the most aggressive form of NB. Genomic amplification and focal alterations affecting cell cycle genes (*CDKN2A/B*, *CCND1*, *CDK4/6*, and *MDM2*) [8,14,15,16,17,18], or genes implicated in chromatin remodeling and telomere maintenance (*ATRX*, *ARID1A/B,* and *TERT*) [8,11,13] have been described as contributing to the development and progression of NB. Large segmental chromosome changes are common in NB, while small chromosome or gene rearrangements are relatively rare, with recurrent somatic mutations mainly found in *ALK*, *ATRX*, *PTPN11,* and *TIAM1* genes [7,8,12,13]. Unfortunately, SCA have not been fully investigated and their prognostic role remains largely unknown. Although many unbalanced chromosomal aberrations in NB have been well characterized, including focal amplification of oncogenes [13,14,15] and deletions of tumor suppressor genes [16,17,18], broad regions of chromosomal gains and losses are poorly understood. It is conceivable that the loss of large chromosome regions leads to the simultaneous inactivation of multiple tumor suppressor genes, greatly decreasing the dosage and the expression of resident genes. Even if chromosome 10q loss is not a frequent occurrence, it has already been reported in both familial and sporadic NB [21,22,23,24]. The prevalence of 10q allelic loss was 22.2% [24] while that of loss of heterozygosity (LOH) was 4.3% [23]. We intended to evaluate the potential impact of chromosome 10q loss on the survival rate of patients affected by NB. To reach this goal, we analyzed the influence of 10q loss on patients’ survival in a dataset of Italian NB cases enrolled in the Italian Neuroblastoma Registry.

## 2. Materials and Methods

### 2.1. Primary Tumors and Cell Lines

NB primary samples were obtained during surgery or biopsy. Tumor tissue samples were collected after resection and stored in the BIT-Gaslini Biobank, Tissue Section, IRCCS Istituto Giannina Gaslini, Genova, Italy. Tumor content was confirmed by a review of hematoxylin and eosin-stained tumor sections by the local pathologists. The stage of the NB patients was classified according to the International Staging System (INSS) [25], and the assessment of the risk of death was defined by the International Neuroblastoma Risk Group (INRG) classification system [26]. The clinical data is derived from the Italian Neuroblastoma Registry (RINB) of the AIEOP (the Italian Association of Pediatric Hematology and Oncology). The clinical characteristics of NB patients are collected in a pseudo-anonymized manner and stored in a secure system at Italian Inter-University Consortium CINECA. Our investigation was conducted following the Declaration of Helsinki, and it has obtained the approval of the Italian Institutional Ethics Committee (Measure n° 270/17 related to the clinical study protocol IGG-NCA.AP-2016, validated on 15 December 2016 and renewed on 24 May 2021). Written informed consent was obtained from the parents of all the NB patients at the time of the enrollment.

Human NB cell lines were obtained as follows: GICAN, IMR-32, and ACN from Interlab Cell Line Collection (ICLC, IRCCS Ospedale San Martino, Genova, Italy), SK-N-AS, SH-SY5Y, SH-EP, SK-N-SH, SK-N-BE(2)c, LA-N-1, and LA-N-5 from the Leibniz-Institute DMSZ, SMS-KCNR from Children’s Oncology Group Cell Culture, Tet-21/N kindly provided by Dr. M. Schwab (University of Heidelberg, Heidelberg, Germany). All NB cell lines were characterized by HLA class I typing by PCR-SSP sets, and by high-resolution oligonucleotide array comparative genomic hybridization (array-CGH) analysis. Cell lines were cultured in Dulbecco’s modified Eagle’s medium (DMEM) High Glucose (EuroClone, Milano, Italy) supplemented with 10% FBS (ThermoFisher Scientific, Waltham, MA, USA) and with 1% L-glutamine/penicillin-streptomycin. Cells were maintained at 37 °C in a humidified atmosphere containing 95% air and 5% CO_2_. Primary samples and cell lines. DNAs from NB primary tumors and cell lines were processed using the QIAamp DNA Extraction Kit (Qiagen, Hilden, Germany).

### 2.2. Genomic Profile Analysis

DNAs from 260 NB primary samples and 12 NB cell lines were tested by high-resolution oligonucleotide array-CGH and Single Nucleotide Polymorphism (SNP) array using a 4 × 180 K platform (Agilent Technologies, Santa Clara, CA, USA) with a mean resolution of approximately 25 kb. The copy number variants were defined as a displacement of the normal value of at least 8 consecutive probes and the mapping positions refer to the Genome Assembly GRCh19/hg19 (UCSC genome browser, http://genome.ucsc.edu, accessed on 20 October 2022). Images of the array were acquired by the Agilent C Scanner, and visualized by the Agilent Feature Extraction 10.5 Software. Data were examined by the genomic Workbench 7.0.40 software (Agilent), to detect chromosomal aberrations and breakpoints by ADM-1 (threshold 10), with 0.5 Mb window size in order to minimize potential false positives. The cutoffs to call aberrations were: 0.2 = gains, −0.3 = losses, 2 = amplifications, and −2 = homozygous deletions. The quality of the test was assessed by the strength of the QC metrics values. Polymorphisms (http://projects.trag.ca/variation/, accessed on 20 October 2022) were not included since they were considered normal variants.

### 2.3. Data Sets

#### 2.3.1. Italian Cohort of NB Patients

A retrospective series of 260 NB primary samples carrying SCA genomic profile of all stages from children and adolescents (age < 18 years) at diagnosis was collected at IRCCS Istituto Giannina Gaslini, Genova, Italy, between 1995 and 2018. The main patients’ characteristics are resumed in Supplementary Table S1. About 38% of patients were aged 0–17 months, 48% 18–59 months, and 15% 60 months or more at the onset of the disease. Males were 53%. The majority of patients had stage 4 disease (51%), and about one-third carried the amplification of the MYCN proto-oncogene. Follow-up was obtained by the database of the Italian Neuroblastoma Registry (RINB) [27]. RINB was activated in 1979 and includes all subjects with any peripheral neuroblastic tumor (i.e., neuroblastoma, ganglioneuroblastoma, and benign ganglioneuroma) diagnosed at the institutions participating in the Italian Neuroblastoma Group. It was estimated that RINB collects more than 90% of the expected peripheral neuroblastic tumor incident cases in Italy [27]. The structure and protocol of RINB were approved by the Ethics Committees of each participating center. Parents or legal guardians of the enrolled patients were asked to sign an informed consent form. For this reason, no specific further consent was needed for this study. The RINB database is located at the Italian Inter-University Consortium CINECA headquarters in Italy and can be accessed only by authorized users. It received the ISO 9001:2015 Quality Management System certification, and the ISO/IEC 27,001:2013 Information Security Management System certification.

#### 2.3.2. In Silico Data Sets

Genes contained in the region of deletion on chromosome 10q associated with poor survival in the RINB data set were traced in the large in silico data set summarized by Cangelosi et al. [28], and their association with both event-free (EFS) and overall survival (OS) was assessed. This dataset describes the gene expression profile of 786 NB tumors at onset and the clinical and molecular information of the patients. It combines the gene expression results from three different platforms. This large dataset is therefore the most complete available to date, concerning NB gene expression profiles and patients’ clinical data [28].

Genes not found in the Cangelosi et al. data set were searched in other two public large in silico data sets, one generated by Agilent customized 4 × 44 K oligonucleotide microarray, including the gene expression profile of 709 and 498 NB tumor samples [29] and one generated by the Illumina HiSeq 2000 platform [30]. All clinical data included in these two public datasets are obtainable via ArrayExpress (http://www.ebi.ac.uk/arrayexpress, accessed on 23 November 2022; login: E-MTAB-1781) and Gene Expression Omnibus (http://www.ncbi.nlm.nih.gov7geo, accessed on 23 November 2022; access: gse62564). Finally, results from in silico analysis were validated on in silico data from an independent cohort of 283 NB patients recently available on the AMC R2 platform [31].

### 2.4. Statistical Analysis

Descriptive statistics were reported as absolute frequencies and percentages for qualitative data, while median and interquartile range (IQR) were used for continuous variables, due to the non-normal distribution of most of them. Comparison between categorical variables was assessed by Pearson’s chi-squared test or Fisher’s exact test when appropriate. The Mann-Whitney U test was used for two-group comparisons of continuous variables, while in the presence of more than two groups, the Kruskal-Wallis test was applied [32].

The Kaplan-Meier method was used to assess the association between the expression of each gene examined and the survival of the patients, who were divided into two and three groups of identical sample size considering median and tertile values, respectively. By the Kalbfleisch and Prentice method, a 95% confidence interval (CI) of survival estimates was calculated [33].

Overall Survival (OS) time was determined starting from the date of diagnosis until the date of death or the last known information. Event Free survival (EFS) was determined starting from the date of diagnosis until the first event of death or disease recurrence. In the NRC-283 database, it was possible to find information on Progression Free survival (PFS) too.

Kaplan-Meier curves were compared by the log-rank test and the log-rank test for trend. A Multivariable Cox regression model was applied to adjust for the confounding effect of the prognostic factors available at diagnosis (*MYCN* status, age, and stage) [33]. NB patients were stratified into subgroups by *MYCN* status and stage at diagnosis (stages 1, 2, 3, 4, and 4s) [34].

All tests were two-tailed and a *p*-value < 0.05 was considered statistically significant. Association between deletion of each gene in the 10q region and patients’ survival was performed by an ad hoc program script implemented in R language, using the library “survival” developed by Herry Therneau. All the other analyses were performed by STATA statistical software (release 13.1, Stata Corporation, College Station, TX, USA).

## 3. Results

### 3.1. Analysis of 10q Loss in NB Samples and Cell Lines

**Figure 1:**
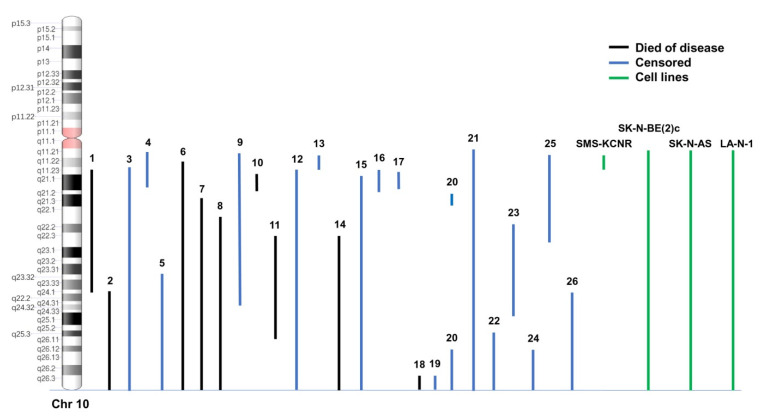
Detailed location of the 10q losses and/or CN-LOH found in 26 NB patients and 4 NB cell lines. Patients who died of disease are indicated in black, censored patients in blue, and cell lines in green.

The genomic profiles obtained by high-resolution array-CGH and SNP-array of 260 NB samples were reevaluated for the presence of 10q loss. In general, we observed recurrent loss of 1p, 3p, 4p, 6q, 11q, 19p and gain of 1q, 2p, 7q, 17q, and amplification of 12q and *MYCN* at expected frequency levels. In 23 NB samples, chromosome 10q was a loss in hemizygosis, in one sample it was a loss in homozygosis in the region 10q21.1, and two samples were subjected to copy-neutral loss of heterozygosity (CN-LOH) of chromosome 10q without changes in copy number. CN-LOH is a mechanism leading to the inactivation of tumor suppressor genes in tumors [35]. The precise loss boundaries are displayed in Figure 1 and Table 1. The other remaining 234 cases had other SCA both recurring and non-recurring, but no loss of chromosome 10q (Supplementary Table S1). We observed that only 38.4% (10/26) of samples with 10q loss also bear *MYCN* amplification (Table 1).

Genomic profile analysis of NB cell lines revealed that chromosome 10q was lost in SK-N-AS, SK-N-BE(2)c, SMS-KCNR, and LA-N-1, it was gained in SH-EP, and it was normal in other cell lines analyzed (Table 2).

### 3.2. Association between NB Patients’ Characteristics and 10q Loss

Table 3 shows the distribution of main patients’ characteristics at diagnosis by the status of chromosome 10q in the analyzed cohort of 260 NB patients. No association was observed with age, gender, stage, and *MYCN* status.

The association between the occurrence of 10q loss and losses in other chromosomes is reported in Supplementary Table S2. A higher proportion of other segmental deletions in patients with 10q loss was observed for chromosome 4q (11.5% vs. 2.6%, *p* = 0.05), 8p (15.4% vs. 2.1%, *p* = 0.007), 19q (19.2% vs. 4.3%, *p* = 0.010), and 21q (11.5% vs. 2.1%, *p* = 0.036). Furthermore, 10q loss was associated with gain in chromosome 1q (34.6% vs. 17.5%, *p* = 0.036), 3q (23.1 vs. 3.0), 5p (11.5% vs. 1.7%, *p* = 0.024), 6p (23.1 vs. 5.6, *p* = 0.006), 7q (23.1% vs. 9.0%, *p* = 0.038), and 17p (7.7 vs. 0.9, *p* = 0.051) (Supplementary Table S3).

**Figure 2:**
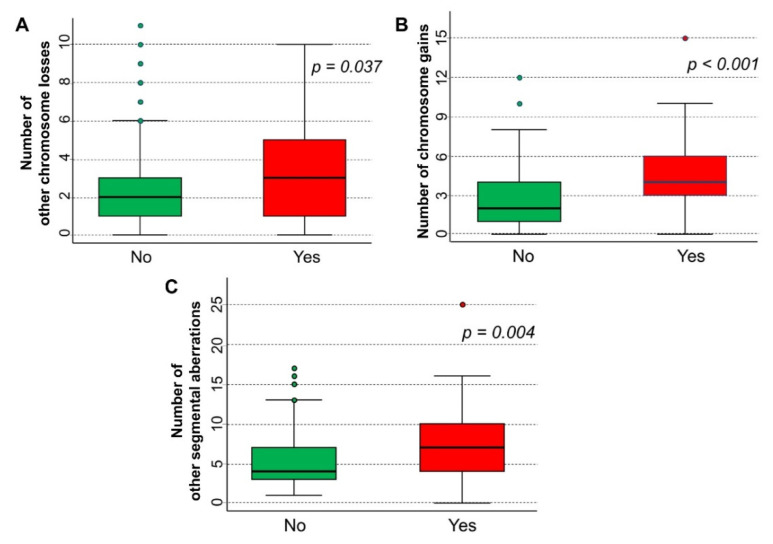
Association between 10q loss and other segmental chromosomal aberrations in a cohort of 260 neuroblastoma patients. (**A**) Other segmental deletions; (**B**) segmental gains; (**C**) any chromosomal aberrations but 10q loss.

The occurrence of loss in chromosome 10q was also associated with the number of other chromosome losses (median value: 2, IQR: 1–3, in patients with no loss of 10q, vs. 3, IQR: 1–5, in patients with loss of 10q, Figure 2A), with the number of chromosome gains (median: 2, IQR: 1–4, vs. 4, IQR: 3–6, Figure 2B), and with any other SCA (median: 4, IQR: 3–7, vs. 7, IQR: 4–10, Figure 2C).

### 3.3. Survival Analysis of NB Patients by the Occurrence of Chromosome 10q Loss

The follow-up time of the entire cohort of 260 NB patients ranged between 0 days and 20 years with a median value of 3 years (IQR: 1.4–5.1). Sixty-nine deaths were observed, which included 4 deaths from toxicity and 1 for causes other than NB. Ninety-seven patients experienced disease relapse or progression, including 59 patients who died. One patient had a secondary malignancy after 1.9 years from diagnosis (acute myeloid leukemia, therapy-related), he was alive and in complete remission for NB at the end of 3 years follow-up.

**Figure 3:**
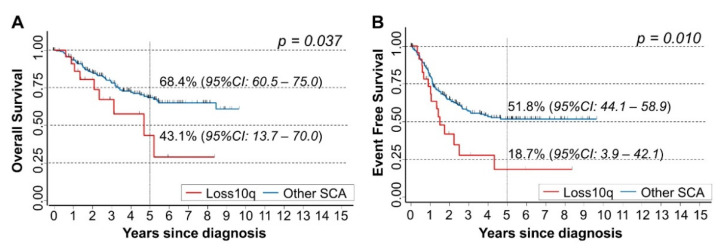
Survival analyses of 260 NB patients in relation to the chromosome 10q loss. (**A**) Overall Survival; (**B**) Event-Free Survival. Five-year survival estimates and their related 95% CI are shown.

Kaplan-Meier survival curves showed that patients carrying chromosome 10q loss had poorer survival compared with patients with other SCA (Figure 3A), and a similar pattern was observed for EFS (Figure 3B). The corresponding HRs adjusted for the main patients’ characteristics at diagnosis (age, *MYCN* status, and stage) were 1.7 for OS (95% CI: 0.84–3.5, *p* = 0.137), and 1.8 for EFS (95% CI: 1.0–3.1, *p* = 0.037).

### 3.4. Deletion Map of Chromosome 10q Loss Associated with NB Patients’ Survival

**Figure 4:**
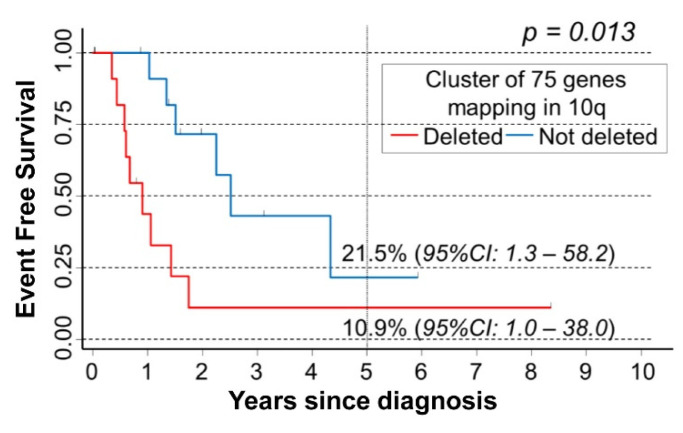
Event Free Survival of 26 NB patients bearing 10q loss in their primary tumor in association with the occurrence of the deletion of the cluster of 75 genes mapping in 10q. Five-year survival estimates and their related 95% CI are shown.

A minimum deleted region in 10q was not identifiable in the cohort of 26 NB patients here examined. In order to identify a set of genes with a potential tumor suppressor activity, OS and EFS analysis was carried out for each gene mapping in 10q splitting the sub-cohort of 26 patients bearing 10q loss (Table 1) into two groups based on the presence of deletion of each considered gene. With regard to OS, no statistically significant association was found with the deletion of any gene mapping in 10q. Conversely, a cluster of 75 genes was detected which were found deleted in 12 patients with very poor EFS survival (Figure 4).

The cluster inside the 10q loss region associated with reduced EFS included the following genes: *ACTA2, AGAP11, ANKRD1, ANKRD22, ANXA11, ATAD1, BMPR1A, C10orf116, C10orf57, C10orf58, C10orf99, CFLP1, CH25H, DYDC1, DYDC2, EIF5AL1, FAM190B, FAM22A, FAM22D, FAM25A, FAM35A, FAS, FLJ37201, GHITM, GLUD1, GRID1, HTR7, IFIT1, IFIT1L, IFIT2, IFIT3, IFIT5, KIF20B, KILLIN, LDB3, LIPA, LIPF, LIPJ, LIPK, LIPM, LIPN, LOC219347, LOC439994, LOC642361, LOC650623, LOC728190, LRIT1, LRIT2, MAT1A, MBL1P, MINPP1, MIR107, MIR346, MMRN2, NRG3, NUDT9P1, OPN4, PANK1, PAPSS2, PCDH21, PCGF5, PLAC9, PTEN, RGR, RNLS, RPP30, SFTPA1, SFTPA2, SFTPD, SH2D4B, SLC16A12, SNCG, STAMBPL1, TSPAN14,* and *WAPAL*.

### 3.5. In Silico Analysis of the Association between the Expression of 75 Genes Identified in the Cluster Inside the 10q Loss Region and Survival of NB Patients

Among the 75 genes mapping in chromosome 10q and found associated with EFS in the cohort under study, 38 were analyzed in the large in silico data set summarized by Cangelosi et al. [28], namely: *ACTA2, ANKRD22, ANXA11, ATAD1, BMPR1A, C10orf58* (alias *FAM213A*), *CH25H, CCSER2* (alias, *Gcap14, FAM190B, KIAA1128, NPD012*), *FAM35A, FAS, FLJ37201, GHITM, GLUD1, GRID1, HTR7, IFIT1, IFIT2, IFIT3, IFIT5, KIF20B, LDB3, LIPA, MINPP1, MMRN2, NRG3, PANK1, PAPSS2, PCDH21* (alias *CDHR1)*, *PCGF5, PLAC9, PTEN, RNLS, RPP30, SFTPA1* (alias *PSDAP*), *SFTPA2* (alias *PSAP*), *SNCG, STAMBPL1,* and *TSPAN14*. Three other genes were traced in the *E-MTAB-1781* data set (*C10Orf57,* alias *FLJ3263, RGR,* and *SFTPD*), and the remaining 34 (*AGAP11, ANKRD1, C10ORF116, C10ORF99, CFL1P1, DYDC1, DYDC2, EIF5AL1, FAM22A, FAM22D, FAM25A, IFIT1B* (alias *IFIT1L*), *KLLN* (alias *KILLIN*), *LIPF, LIPJ, LIPK, LIPM, LIPN, LOC439994, LOC642361, LOC650623, LOC728190, LRIT1, LRIT2, MAT1A, MBL1P, MIR107, MIR346, NUDT9P1, OPN4, SH2D4B, SLC16A12, TMEM254AS1,* and *WAPAL*) were found in SEQC-498 database. A positive association between gene expression and patients’ survival, observed in univariable analysis and confirmed in a multivariable Cox regression model adjusting for the available confounders (*MYCN* status, age, and stage at diagnosis), was found for eight genes, namely: *AGAP11, CCSER2, FAM22A, FAM22D, IFIT2, PAPSS2, PCGF5,* and *NUDT9P1*. Validation on the independent cohort NRC-283 *in silico*, confirmed the observed association only for *CCSER2* both in univariable and in multivariable regression analysis, adjusting for *MYCN* status, age, and stage at diagnosis. Furthermore, the stratified analysis showed a potential protective effect of high *CCSER2* expression levels in patients with both amplified and normal *MYCN* status, and in both localized and stage 4 diseases. The expression level of *FAM22A* and *FAM22D* was not available in the NRC-283 database. For the other five genes, after adjustment for the available confounders, the association with patients’ survival was no longer observed. The following paragraphs report the details of the analyses for the *CCSER2* gene, while the results inherent to the genes *AGAP11, IFIT2, PAPSS2, PCGF5,* and *NUDT9P1* are reported in Supplementary Genes Analysis, in Supplementary Figures S1–S20 and Supplementary Tables S4–S13. The results concerning all the other analyzed genes are reported in Supplementary Figures S21–S84 and Supplementary Tables S14–S83, except for *C10orf99*, *FAM25A*, *MIR-107*, and *MIR-346* genes, which were not analyzed since they presented with ≥90% of missing values, probably related to the absence of expression in NB samples. The main features of the datasets here utilized are listed in Table 4.

#### CCSER2

**Figure 5:**
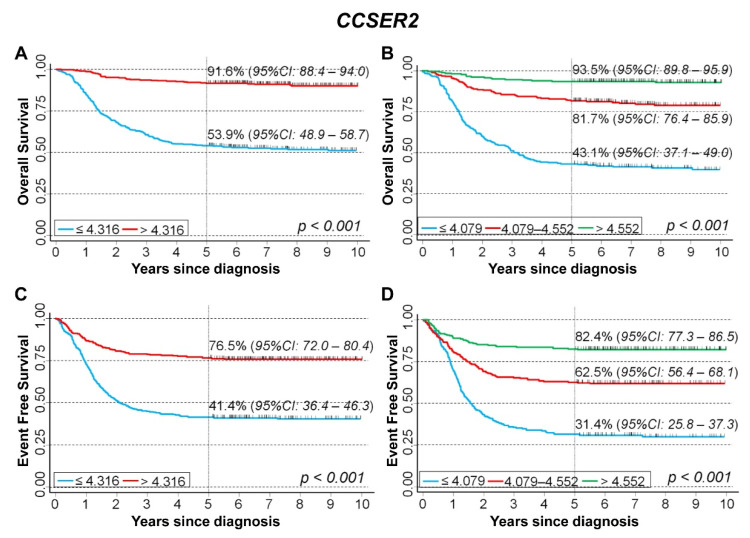
Survival analyses of 786 NB patients using the Cangelosi et al. dataset for *CCSER2* gene expression. Overall survival, cut-off on the median (**A**) or tertile (**B**) expression value; Event-free survival, cut-off on the median (**C**) or tertile (**D**) expression value. Five-year survival estimates and their related 95% CI are shown.

We examined the association between *CCSER2* gene expression and the survival rate of 786 NB patients in Cangelosi et al. data set, finding that the overall survival appears to be strongly associated with *CCSER2* gene expression (Figure 5A). Splitting the data on the basis of tertile values, a clear trend emerged (Figure 5B). Results from the analysis of EFS were consistent with those observed for OS. In particular, 5-year EFS was 41.4% for gene expression values below the median, and 76.5% for values above (Figure 5C). Splitting the data on the basis of the tertile values, 5-year EFS was 31.4% for low expression levels, 62.5% for intermediate levels, and 82.4% for high values (Figure 5D).

**Figure 6:**
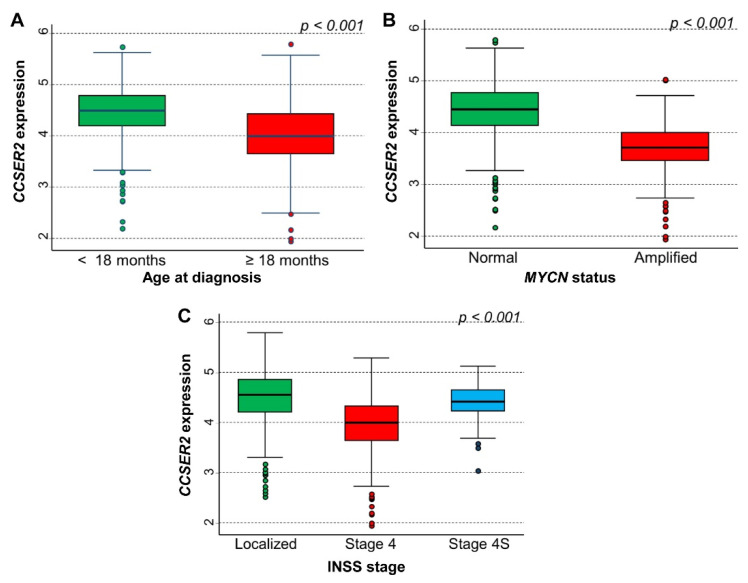
Association between *CCSER2* gene expression and the main patients’ characteristics at diagnosis evaluated on the cohort of 786 neuroblastoma patients in the data set of Cangelosi et al. (**A**) Age; (**B**) *MYCN* status; (**C**) INSS stage.

*CCSER2* gene expression was strongly associated with each favorable prognostic factor, which included lower age at the onset of disease (Figure 6A), normal *MYCN* status (Figure 6B), and both localized and 4S stages (Figure 6C).

Multivariable analysis via the Cox regression model (Table 5) confirmed the positive association between *CCSER2* expression and patients’ survival. Adjusted HRs obtained by splitting the data on the median value were 0.37 for OS, and 0.55 for EFS. Splitting the data on the tertile values, a clear trend in HRs emerged (*p* < 0.001 for both OS and EFS). HRs adjusted estimates for OS were 0.60 for intermediate levels, and 0.27 for higher levels. The corresponding figures for EFS were 0.71, and 0.35. The positive association between gene expression and patients’ survival was confirmed by the highly significant linear trend in both OS and EFS observed including in the Cox model the original continuous values of *CCSER2* expression.

**Figure 7:**
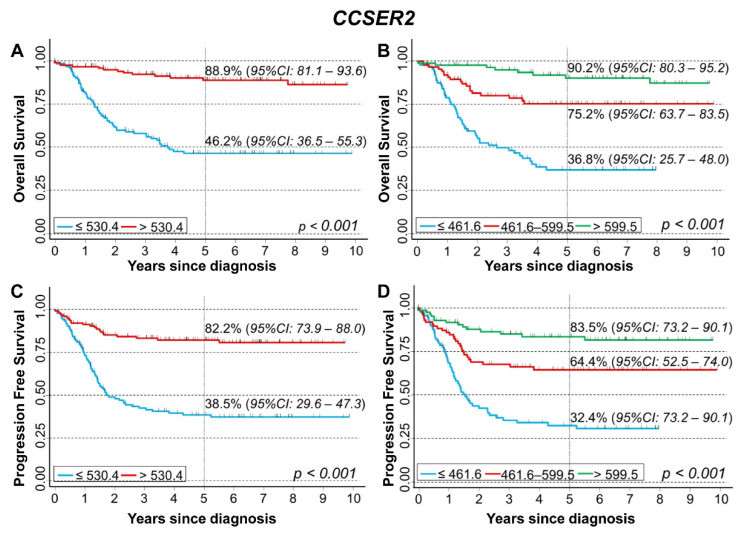
Survival analyses of 283 NB patients using the NRC-283 dataset for *CCSER2* gene expression. Overall survival, cut-off on the median (**A**) or tertile (**B**) expression value; Progression-free survival, cut-off on the median (**C**) or tertile (**D**) expression value. Five-year survival estimates and their related 95% CI are shown.

Results of the analysis of the in silico cohort of 283 NB patients in the NRC-283 database confirmed the positive association between *CCSER2* gene expression values and both overall and progression-free survival. Five-year OS was 46.2% for values below the median expression level and 88.9% for values above (Figure 7A). A clear trend was observed splitting the data on the tertiles (Figure 7B). With regard to PFS, a clear difference also emerged between estimates above and below the median expression level (Figure 7C). A trend in PFS estimates by increasing values of *CCSER2* expression was also observed splitting the data on the tertiles (Figure 7D).

**Figure 8:**
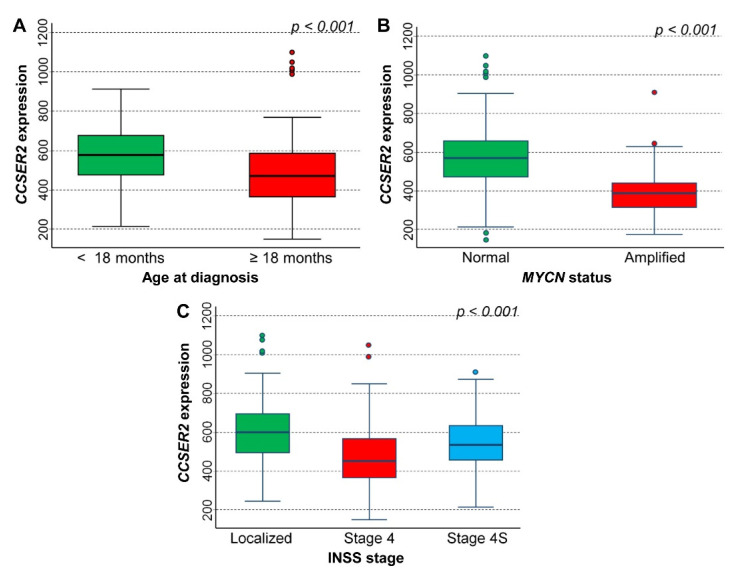
The association between *CCSER2* gene expression and the main patient characteristics at diagnosis was evaluated on the cohort of 283 neuroblastoma patients in the NRC-283 data set. (**A**) Age; (**B**) *MYCN* status; (**C**) INSS stage.

*CCSER2* gene expression values were positively associated with favorable prognostic factors, including lower age at diagnosis (Figure 8A), normal *MYCN* status (Figure 8B), and localized stage or stage 4S (Figure 8C).

Adjusting for these potential confounders, the possible protective effect of high values of gene expression was confirmed in multivariable Cox regression analysis, both for OS and EFS (Table 6). In detail, for OS, HR was 0.36 for values above the median expression vs. values below. Stratification by tertile values showed a clear trend. A similar pattern emerged for PFS.

**Figure 9:**
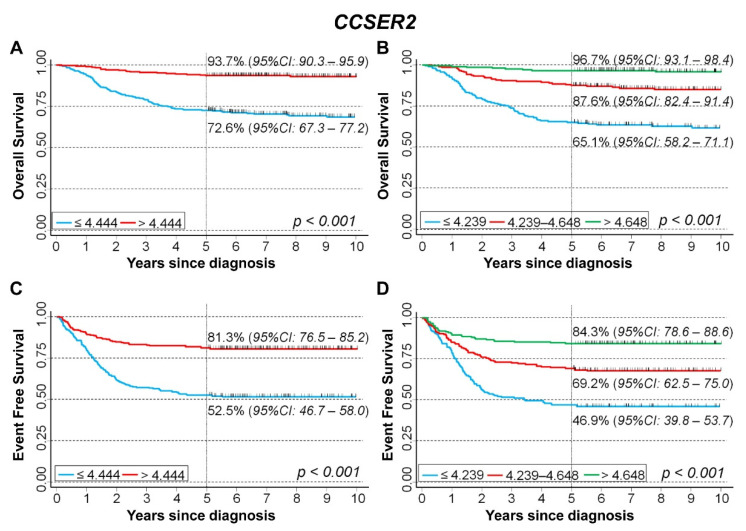
Survival analyses of 629 NB patients with not amplified *MYCN* using the Cangelosi et al. dataset for *CCSER2* gene expression. Overall survival, cut-off on the median (**A**) or tertile (**B**) expression value; Event-free survival, cut-off on the median (**C**) or tertile (**D**) expression value. Five-year survival estimates and their related 95% CI are shown.

Results of the analysis of the sub-cohort of the Cangelosi et al. dataset with normal *MYCN* status showed a positive association between gene expression levels and OS, both splitting the data based on the median expression value (Figure 9A) or stratifying on the tertiles (Figure 9B). A similar pattern emerged for EFS (Figure 9C,D).

Multivariable analysis via the Cox regression model confirmed the observed association between *CCSER2* expression and patients’ survival (Table 7). With regard to OS, HR was 0.37 for values above the median expression vs. values below. Stratifying on the tertiles, a clear trend was observed. A consistent pattern emerged for EFS.

**Figure 10:**
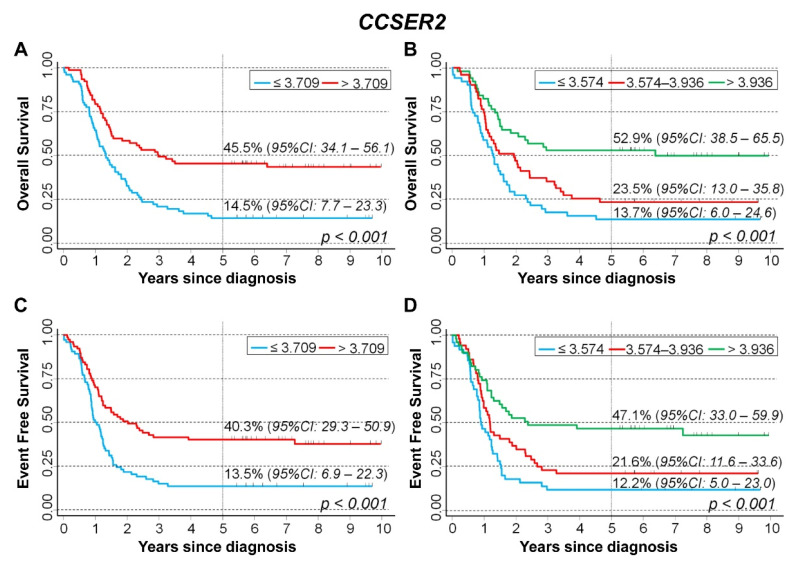
Survival analyses of 153 NB patients with amplified *MYCN* using the Cangelosi et al. dataset for *CCSER2* gene expression values. Overall survival, cut-off on the median (**A**) or tertile (**B**) expression value; Event-free survival, cut-off on the median (**C**) or tertile (**D**) expression value. Five-year survival estimates and their related 95% CI are shown.

The potential protective role of *CCSER2* high expression values was also observed among patients with amplified *MYCN* status. OS was 14.5% in patients with values below the median and 45.5% for patients with values above (Figure 10A). Stratifying the tertile values, OS was 13.7% for low values, 23.5% for intermediate values, and 52.9% for high values (Figure 10B). With regard to EFS, survival probability was 13.5% for values below the median, 40.3% for values above (Figure 10C), and was 12.2% for values below the first tertile, 21.6% for intermediate values, and 47.1% for values above the second tertile (Figure 10D).

Multivariable analysis confirmed the positive association between *CCSER2* expression and patients’ survival (Table 8). With regard to OS, HR was 0.49 splitting the data on the median expression value. HR was 0.38 for values below the first tertile and 0.69 for intermediate values vs. the lowest levels. A similar pattern was observed for EFS.

**Figure 11:**
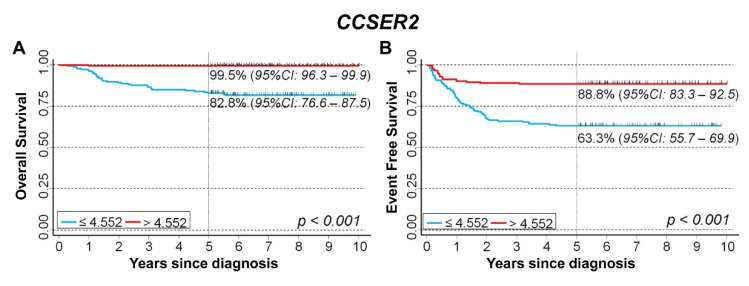
Survival analyses of 373 NB patients with a localized stage at diagnosis using the Cangelosi et al. dataset for *CCSER2* gene expression. Cut-off on the median expression value. (**A**) Overall survival; (**B**) Event-free survival. Five-year survival estimates and their related 95% CI are shown.

Analyzing patients with a localized stage at diagnosis, we found that the very small number of observed events in some subgroups did not allow us to both perform the analysis stratified on the tertile values and adjust for the potential confounding of clinical variables in a Cox regression model. OS was 82.8% for patients with expression values below the median, and 99.5% for values above (Figure 11A). With regard to EFS, the corresponding estimates were 63.3% and 88.8%, respectively (Figure 11B).

**Figure 12:**
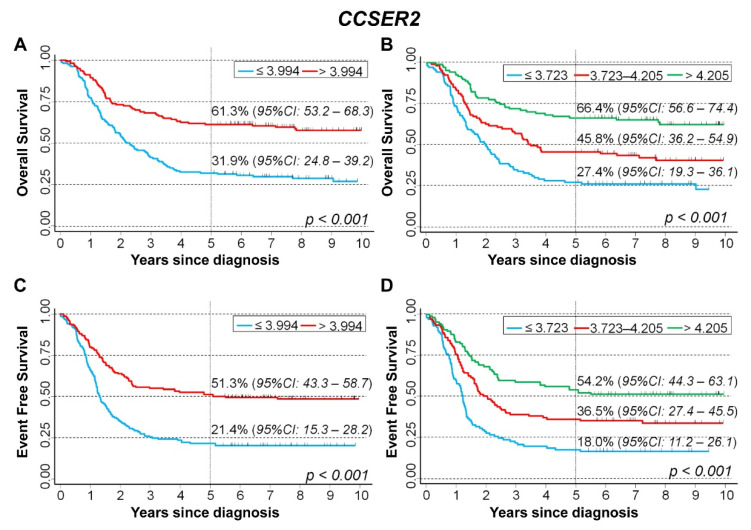
Survival analyses of 320 NB patients with stage 4 at diagnosis using the Cangelosi et al. dataset for *CCSER2* gene expression. Overall survival, cut-off on the median (**A**) or tertile (**B**) expression value; Event-free survival, cut-off on the median (**C**) or tertile (**D**) expression value. Five-year survival estimates and their related 95% CI are shown.

Among patients with stage 4 disease, a better prognosis was associated with high *CCSER2* expression levels. In detail, OS was 31.9% in correspondence to values below the median and 61.3% for values above (Figure 12A). Splitting the data on the tertile values, OS was 27.4% for patients with the lowest values, 45.8% for patients with intermediate values, and 66.4% for patients with the highest values, respectively (Figure 12B). A similar pattern was observed for EFS, where the corresponding estimates were 21.4% and 51.3%, splitting the data on the median expression level, (Figure 12C), and 18.0%, 36.5%, and 54.2% splitting the data on the tertile values (Figure 12D), respectively.

Multivariable Cox regression analysis, adjusting for the potential effect of the main available confounders, confirmed the positive association between *CCSER2* expression and patients’ survival (Table 9). For OS, HR was 0.65 for values above vs. below the median of gene expression and 0.51 or 0.71 for high or intermediate values vs. low values, obtained stratifying data on the tertiles. Consistent data were observed for EFS, where HR was 0.65 splitting the data on the median value, and 0.53 or 0.66, splitting the data on the tertiles, respectively.

## 4. Discussion

In recent years, recurrent losses and gains of large chromosomal regions related to NB prognosis have been identified. NB is mainly driven by structural chromosomal aberrations such as losses of chromosomes 1p, 3p, 4p, 6q, 11q, 19p, gains of 1q, 2p, 7q, 17q, amplifications of genes on 2p and 12q and of *MYCN* oncogene, and loss of whole chromosome X, which are all markers of high-risk disease [13,14,15,16,17,18,19,20]. This suggests that in the same chromosomal region tumor suppressor genes and oncogenes may coexist and cooperate in causing both tumor initiation and progression.

Here we provided evidence that the loss of the whole or part of the long arm of chromosome 10, although rare, correlates with poor prognosis in NB patients. Consistent with our findings, an NB study combining genomic and expression microarray data with survival data identified, along with other chromosomal abnormalities, 10q loss as a negative prognostic indicator [36]. These data highlight how the 10q loss can affect the transcriptional program of NB, and the need to define candidate tumor suppressor genes within this chromosome region. We hypothesized that the loss of chromosome 10q could induce the collective inactivation of many tumor suppressor genes by regulating the different tumor stages. To address this theory, we initially investigated a cluster of 75 genes mapped within the regions of 10q loss characterized by our 26 NB cases and associated with reduced EFS. A survival analysis of each gene in relation to the gene expression was performed using in silico data related to 786 NB patients included in the large data set summarized by Cangelosi et al. [28]. Six genes *CCSER2*, *AGAP11*, *IFIT2*, *PAPSS2*, *PCGF5*, and *NUDT9P1* were observed to have potential NB tumor suppressor activity since their low expression was associated with poor survival even after correction for confounding by other prognostic factors (*MYCN* status, age at diagnosis, stage of disease). Then we suggest that the reduced expression of these genes provoked by 10q loss could promote the progression of NB.

Among them, the *CCSER2* gene showed the greatest impact on the survival of NB patients. *CCSER2* gene (also known as *Gcap14*, *FAM190B*, *KIAA1128*, *NPD012*) encodes for the Coiled-Coil Serine Rich 2 protein, that has been previously linked, along with other proteins, to the DISC1 pathway [37] through binding DISC1 itself or one of its interacting partners. DISC1 pathway is involved in neurogenesis, synapse regulation, neurite outgrowth, neural differentiation, proliferation, and migration [38,39]. What is interesting is that the protein DISC1 is involved in human NB cells regulating the activation of PI3K [39,40]. Furthermore, *CCSER2* is a regulator of microtubule dynamics during neurodevelopment, and its deficiency results in defective neuronal migration [41]. *CCSER2* has not yet been described as a tumor suppressor gene, however, there is a good chance that it could be involved in the development or progression of NB, even if functional studies will be needed to define this role. Notably, missense loss-of-function mutations of the *CCSER2* gene have been reported in NB primary tumors [8].

*AGAP11* is a long non-coding ribonucleic acid (lncRNA) that belongs to the ankyrin repeat and GTPase domain Arf GTPase activating protein gene family [42]. The lncRNAs commonly play a salient role in the spatiotemporal regulation of gene expression during developmental processes [43]. *AGAP11* due to its inherent role in cell function as lncRNA mediates changes in the microenvironment and thereby participates in the development of cellular senescence [42]. Although there is no experimental data related to a particular function in the oncology field for *AGAP11,* there is evidence to suggest that genes closely related to tumors (such as *LPL*, *MMP9*, *SGK1*, *TLR4*, and *FOS*) interact with this lncRNA through different mechanism [44,45,46]. In light of our data, *AGAP11* could be an NB tumor suppressor lncRNA at the 10q23.2 region, which needs to be checked with cellular functional studies.

*IFIT2* (interferon-induced proteins with tetratricopeptide 2) gene is a crucial interferon-stimulated gene family protein, which has been confirmed to play an important role in the anti-tumor process inhibiting the proliferation and migration of cancer cells [47]. *IFIT2* also improves cell death through apoptosis via a mitochondrial pathway on Bcl2 [48]. Studies have shown that *IFIT2* depletion can lead to up-regulation of TNFα promoting angiogenesis, metastasis, epithelial-mesenchymal transition, cell migration, and invasion of oral squamous cell carcinoma [49]. Some studies have shown IFIT proteins as prognostic markers to determine the clinical outcome of many cancers [50,51,52,53,54]. Our analysis suggests that the down-regulation of *IFIT2* was a negative prognostic factor for NB patients. Finally, it has been suggested that IFIT proteins could be a novel therapeutic target for tumor therapy [55], so IFIT2 could become a possible target in NB too.

*PAPSS2* gene encodes for the bifunctional 3′-phosphoadenosine 5′-phosphosulfate synthetase 2, a rate-limiting enzyme in the sulfation pathway, and it was demonstrated that depletion of PAPSS2 led to premature cell senescence in xenograft tumor mouse model and various cancer cell [56]. To our knowledge, the role of *PAPSS2* in NB progression is poorly defined, and no study, apart from ours, has elucidated the association between reduced *PAPSS2* gene expression and decreased survival of NB patients.

*PCGF5* gene encodes for the polycomb group ring finger protein 5, an important regulator of gene expression, and chromatin structure during neuronal development, acting primarily by modifying histones and reducing DNA accessibility [57]. Indeed, it has been reported that PCGF5 is a crucial requirement in the differentiation of mouse embryonic stem cells toward a neuronal cell fate [58]. PCGF5 is not only engaged to repress genes but also binds to active genes during neuronal differentiation [58].

*NUDT9P1* (Nudix hydrolase 9 pseudogene 1) is a non-coding gene regulated by MYCN [59]. Interestingly, low expression of the *NUDT9P1* gene was associated with poor outcomes of *MYCN*-non-amplified NB patients [59]. We also showed that low *NUDT9P1* expression was associated with unfavorable patients’ characteristics at diagnosis, and higher values were observed for low-risk patients such as localized stage or stage 4S. Therefore, the *NUDT9P1* MYCN-driven gene might play critical a role in NB carcinogenesis.

In addition, eight genes with potential oncogenic action were identified whose expression was inversely related to survival by correcting for the effect of possible confounders: *ANXA11*, *EIF5AL1*, *GLUD1*, *KIF20B*, *LDB3*, *MINPP1*, *PANK1*, and *TSPAN14*.

Our study demonstrates that loss of chromosome 10q affects multiple genes and that these genes may act collectively or redundantly at more than one stage of NB progression. The pathways associated with these six genes may represent promising targets for future therapeutic interventions.

## 5. Conclusions

Here we suggest that six genes could be potentially implicated as a target of 10q loss in NB: *CCSER2*, *AGAP11*, *IFIT2*, *PAPSS2*, *PCGF5*, and *NUDT9P1*. In particular, the *CCSER2* gene might have potential relevance in NB as it is normally expressed during neurodevelopment, and its deficiency results in defective neuronal migration. Since NB is known to originate from neural crest cells, their defective migration could lead to the development of this peripheral neuroblastic tumor. Additional functional studies in vivo and in vitro must be carried out to demonstrate it. Nevertheless, it is likely that other genes resident on chromosome 10q may also be NB suppressors. In summary, our study points out that the loss of the long arm of chromosome 10 leads to the inactivation of multiple genes with potential tumor suppressive activity, acting collectively during the different steps of NB progression.

## Figures and Tables

**Table 1:** Chromosome 10q loss in 26 NB patients.

Case N°	Age at Onset (Months)	INSS Stage	INRG Stage	*MYCN* Status	Cytoband and Chromosomal Coordinates of 10q Loss	Relapse	Follow-Up	Disease State
1	47	4	M	gain	10q11.23–q21.3; chr10:51676176–69078545	yes	dead	
2	68	4	M	gain	10q24.1–q26.3; chr10: 98064330–135404523	yes	dead	
3	40	3	L2	amplified	10q11.23–q26.3; chr10: 50955699–135372492	no	alive	CR
4	46	4	M	single copy	10q11.21–21.1; chr10: 42969765–57723272	yes	alive	AD
5	55	4	M	amplified	10q23.32–q26.3; chr10: 93204607–135404523	no	alive	CR
6	5	1	L1	amplified	10q11.22–q26.3; chr10: 48334407–135404523	yes	dead	
7	10	3	L2	amplified	10q21.3–q26.3; chr10: 64979620–135404523	no	dead	
8	14	1	L1	amplified	10q22.1–q26.3; chr10: 73406500–135404523	yes	dead	
9	200	2A	L1	single copy	10q11.21–q24.32; chr10: 42969765–103988947	yes	alive	CR
10	45	4	M	gain	10q21.1; chr10: 55252216–56505255	yes	dead	
11	37	1	L1	single copy	10q22.3–q26.11; chr10: 78015106–121458431	yes	dead	
12	53	2B	L1	gain	CN-LOH 10q11.23–q26.3; chr10: 4541520–67615559	yes	alive	CR
13	52	4	M	gain	10q11.22; chr10: 46938469–48317747	yes	alive	AD
14	22	4	M	amplified	10q22.3–q26.3; chr10: 81234748–135404523	yes	dead	
15	33	3	L2	amplified	10q21.1–q26.3; chr10: 57654752–135411735	no	alive	AD
16	1	4S	Ms	gain	CN-LOH 10q21.1–21.2; chr10: 58949883–63882016	no	alive	CR
17	2	1	L1	gain	10q21.1; chr10: 56329864–58030413	na	na	
18	12	3	L2	gain	10q26.3; chr10:133245712–135405996	no	dead	
19	10	4	M	single copy	10q26.3; chr10:133079678–134996216	no	alive	CR
20	31	4	M	single copy	10q21.3; 10q26.13–q26.3; chr10:68757961–69106575 chr10:123773590–135421826	no	alive	AD
21	30	4	M	amplified	10q11.21–q26.3; chr10:45247685–135372492	yes	alive	AD
22	20	4	M	amplified	10q25.3–q26.3; chr10:116405945–135434178	no	alive	na
23	20	3	L2	single copy	10q22.2–q24.33; chr10:75923421–105458525	no	alive	AD
24	59	4	M	single copy	10q26.13–q26.3; chr10:123773590–135377532	na	na	
25	52	4	M	single copy	10q11.22–q22.3; chr10:49797866–77817731	no	alive	CR
26	46	4	M	amplified	10q24.1–q26.3; chr10: 98515392–135404523	yes	alive	AD

AD, active disease; CR, complete remission; CN-LOH, copy-neutral loss of heterozygosity; na, not available; INSS, International Neuroblastoma Staging System; INRG, International Neuroblastoma Risk Group.

**Table 2:** Chromosome 10q status in 12 NB cell lines.

NB Cell Line	Chromosome 10q Status	Chromosomal Coordinates of 10q Loss	*MYCN* (2p24.3)
SK-N-AS	10q11.21–q26.3 loss	Chr10: 43615122–135474787	single copy
SK-N-BE(2)c	10q11.21–q26.3 loss	Chr10: 42418957–135434178	amplification
LA-N-1	10q11.21–q26.3 loss	Chr10: 42976950–135234843	amplification
SMS-KCNR	10q11.22 loss	Chr10: 46938469–49262406	single copy
SH-EP	10q23.31–q24.32 gain	Chr10:90628315–103956178	gain
GICAN	normal	-	single copy
ACN	normal	-	single copy
SH-SY5Y	normal	-	gain
SK-N-SH	normal	-	amplification
LA-N-5	normal	-	amplification
IMR32	normal	-	amplification
Tet-21/N	normal	-	single copy

**Table 3:** Association between chromosome 10q status and patients’ characteristics in a cohort of 260 neuroblastoma patients with segmental chromosome alterations (SCA) at diagnosis.

	Other SCA *		Loss 10q		
Patients’ Characteristics	*N*	%	*N*	%	*p*
**Age (months)**					0.369
0–17	91	38.9	7	26.9	
18–59	108	46.2	16	61.5	
≥60	35	15.0	3	11.5	
**Gender**					0.619
Male	123	52.6	15	57.7	
Female	111	47.4	11	42.3	
**INSS Stage**					0.999
1–2	55	23.6	6	24.0	
3	42	18.0	5	20.0	
4	119	51.1	13	52.0	
4S	17	7.3	1	4.0	
***MYCN* status**					0.345
Non-amplified	165	70.5	16	61.5	
Amplified	69	29.5	10	38.5	

* Other than chromosome 10q loss.

**Table 4:** Main features of the four datasets utilized in the study.

	Cangelosi et al. (*N* = 786)		E-MTAB-1781 (*N* = 709)		SEQC-498 (*N* = 498)		NRC-283 (*N* = 283)	
Patient Characteristics	*N*	%	*N*	%	*N*	%	*N*	%
**Age at diagnosis**								
<18 months	449	57.1	431	60.8	305	61.2	145	51.2
≥18 months	337	42.9	278	39.2	198	38.8	133	47.0
Missing	0	0.0	0	0.0	0	0.0	5	1.8
**INSS Stage**								
1	143	18.2	159	22.4	121	24.3	50	17.7
2	125	15.9	118	16.6	78	15.7	36	12.7
3	105	13.4	93	13.1	63	12.7	43	15.2
4	320	40.7	259	36.5	183	36.8	124	43.8
4s	92	11.7	80	11.3	53	10.6	27	9.5
Missing	1	0.1	0	0.0	0	0.0	3	1.1
***MYCN* status**								
Not amplified	629	80.0	581	82.0	401	80.5	222	78.4
Amplified	153	19.5	122	17.2	92	18.5	55	19.4
Missing	4	0.5	6	0.8	5	1.0	6	2.2
Events *	320	40.7 ^1^	256 ^2^	36.1	183	36.9	97 ^3^	34.3
**Deaths**	229	29.1	161	22.7	105	21.1	75 ^4^	26.5

*, For NRC-283, events are referred to progression free survival; ^1^, 17 missing for the Event Free Survival; ^2^, 14 missing for the Event Free Survival; ^3^, 8 missing for the Progression Free Survival; ^4^, 7 missing for the Overall Survival.

**Table 5:** Survival of the cohort of 786 NB patients from the Cangelosi et al. data set, in relation to *CCSER2* gene expression values, evaluated by the Cox model.

		Univariable Analysis			Multivariable Analysis		
Gene Expression	N/O	HR	95% CI	*p*	HR	95% CI	*p*
**Overall Survival**							
Median				<0.001			<0.001
≤4.316 (reference)	393/192	1	-		1	-	
>4.316	393/37	0.14	0.10–0.21		0.37	0.25–0.55	
Tertiles				<0.001			<0.001
≤4.079 (reference)	262/157	1	-		1	-	
4.079–4.552	262/54	0.25	0.18–0.34		0.60	0.43–0.85	
>4.552	262/18	0.08	0.05–0.13		0.27	0.16–0.47	
Continuous variable	786/229	0.28	0.23–0.33	<0.001	0.56	0.45–0.69	<0.001
**Event Free Survival**							
Median				<0.001			<0.001
≤4.316 (reference)	377/225	1	-		1	-	
>4.316	392/95	0.32	0.25–0.40		0.55	0.41–0.72	
Tertiles				<0.001			<0.001
≤4.079 (reference)	248/173	1	-		1	-	
4.079–4.552	259/100	0.44	0.34–0.56		0.71	0.53–0.94	
>4.552	262/47	0.18	0.13–0.25		0.35	0.24–0.51	
Continuous variable	769/320	0.35	0.30–0.41	<0.001	0.55	0.45–0.68	<0.001

N/O, Number of patients/Outcome (deaths for the overall and events for the event-free survival); HR, Hazard Ratio. Multivariable analysis: HRs are adjusted by *MYCN* status, age, and stage at diagnosis.

**Table 6:** Survival of the cohort of 283 NB patients from the NRC-283 data set, in relation to *CCSER2* gene expression values, was evaluated by the Cox model.

		Univariable Analysis			Multivariable Analysis		
Gene Expression	N/O	HR	95% CI	*p*	HR	95% CI	*p*
**Overall Survival**							
Median				<0.001			0.003
≤530.4 (reference)	141/62	1	-		1	-	
>530.4	135/13	0.16	0.09–0.29		0.36	0.18–0.72	
Tertiles				<0.001			0.017
≤461.6 (reference)	94/48	1	-		1	-	
461.6–599.5	92/19	0.30	0.18–0.52		0.68	0.37–1.3	
>599.5	90/8	0.11	0.05–0.23		0.36	0.15–0.85	
Continuous variable	276/75	0.994	0.993–0.996	<0.001	0.997	0.995–0.999	0.005
**Progression Free Survival**							
Median				<0.001			<0.001
≤530.4 (reference)	140/75	1	-		1	-	
>530.4	135/22	0.22	0.14–0.37		0.38	0.22–0.66	
Tertiles				<0.001			0.008
≤461.6 (reference)	94/55	1	-		1	-	
461.6–599.5	91/28	0.41	0.26–0.64		0.71	0.41–1.2	
>599.5	90/14	0.18	0.10–0.32		0.39	0.19–0.79	
Continuous variable	265/97	0.996	0.995–0.997	<0.001	0.998	0.997–0.999	0.014

N/O, Number of patients/Outcome (deaths for the overall and events for the progression-free survival); HR, Hazard Ratio. Multivariable analysis: HRs are adjusted by *MYCN* status, age, and stage at diagnosis.

**Table 7:** Survival of the cohort of 629 NB patients with not amplified *MYCN* status from the Cangelosi et al. dataset, in relation to *CCSER2* gene expression values, evaluated by the Cox model.

		Univariable Analysis			Multivariable Analysis		
Gene Expression	N/O	HR	95% CI	*p*	HR	95% CI	*p*
**Overall Survival**							
Median				<0.001			<0.001
≤4.444 (reference)	314/98	1	-		1	-	
>4.444	315/21	0.18	0.11–0.29		0.37	0.23–0.60	
Tertiles				<0.001			<0.001
≤4.239 (reference)	209/80	1	-		1	-	
4.239–4.648	210/31	0.32	0.22–0.50		0.66	0.43–1.0	
>4.648	210/8	0.08	0.04–0.17		0.20	0.10–0.43	
Continuous variable	629/119	0.25	0.19–0.32	<0.001	0.52	0.38–0.70	<0.001
**Event Free Survival**							
Median				<0.001			<0.001
≤4.444 (reference)	299/146	1	-		1	-	
>4.4444	315/61	0.33	0.25–0.47		0.44	0.32–0.61	
Tertiles				<0.001			<0.001
≤4.239 (reference)	196/107	1	-		1	-	
4.239–4.648	208/67	0.51	0.37–0.69		0.68	0.50–0.94	
>4.648	210/33	0.23	0.15–0.34		0.34	0.23–0.52	
Continuous variable	614/207	0.28	0.22–0.37	<0.001	0.44	0.33–0.59	<0.001

N/O, Number of patients/Outcome (deaths for the overall and events for the progression-free survival); HR, Hazard Ratio. Multivariable analysis: HRs are adjusted by age, and stage at diagnosis.

**Table 8:** Survival of the cohort of 153 NB patients with amplified *MYCN* status from the Cangelosi et al. dataset, in relation to *CCSER2* gene expression values, evaluated by the Cox model.

		Univariable Analysis			Multivariable Analysis		
Gene Expression	N/O	HR	95% CI	*p*	HR	95% CI	*p*
**Overall Survival**							
Median				<0.001			<0.001
≤3.709 (reference)	76/65	1	-		1	-	
>3.709	77/43	0.45	0.31–0.67		0.49	0.33–0.73	
Tertiles				<0.001			<0.001
≤3.574 (reference)	51/44	1	-		1	-	
3.574–3.936	51/39	0.69	0.45–1.1		0.69	0.45–1.1	
>3.936	51/25	0.35	0.21–0.57		0.38	0.23–0.63	
Continuous variable	153/108	0.65	0.47–0.88	0.008	0.70	0.50–0.97	0.038
**Event Free Survival**							
Median				<0.001			0.001
≤3.709 (reference)	74/64	1	-		1	-	
>3.709	77/47	0.49	0.33–0.71		0.53	0.36–0.78	
Tertiles				<0.001			<0.001
≤3.574 (reference)	49/43	1	-		1	-	
3.574–3.936	51/40	0.69	0.45–1.1		0.69	0.45–1.1	
>3.936	51/28	0.39	0.24–0.63		0.42	0.26–0.68	
Continuous variable	151/111	0.69	0.51–0.95	0.023	0.75	0.54–1.04	0.096

N/O, Number of patients/Outcome (deaths for the overall and events for the progression-free survival); HR, Hazard Ratio. Multivariable analysis: HRs are adjusted by age, and stage at diagnosis.

**Table 9:** Survival of the cohort of 320 NB patients with stage 4 at diagnosis from the Cangelosi et al. dataset, in relation to *CCSER2* gene expression values, evaluated by the Cox model.

		Univariable Analysis			Multivariable Analysis		
Gene Expression	N/O	HR	95% CI	*p*	HR	95% CI	*p*
**Overall Survival**							
Median				<0.001			0.009
≤3.994 (reference)	160/116	1	-		1	-	
>3.994	160/67	0.43	0.32–0.58		0.65	0.47–0.90	
Tertiles				<0.001			<0.001
≤3.723 (reference)	106/80	1	-		1	-	
3.723–4.205	107/63	0.61	0.44–0.85		0.71	0.51–0.99	
>4.205	107/40	0.32	0.22–0.46		0.51	0.33–0.77	
Continuous variable	320/183	0.52	0.41–0.64	<0.001	0.68	0.53–0.87	0.003
**Event Free Survival**							
Median				<0.001			0.006
≤3.994 (reference)	154/123	1	-		1	-	
>3.994	160/83	0.45	0.34–0.60		0.65	0.48–0.89	
Tertiles				<0.001			0.001
≤3.723 (reference)	100/83	1	-		1	-	
3.723–4.205	107/70	0.56	0.41–0.78		0.66	0.48–0.91	
>4.205	107/53	0.36	0.25–0.50		0.53	0.36–0.79	
Continuous variable	314/206	0.51	0.41–0.63	<0.001	0.65	0.51–0.85	0.002

N/O, Number of patients/Outcome (deaths for the overall and events for the progression-free survival); HR, Hazard Ratio. Multivariable analysis: HRs are adjusted by *MYCN* status and age.

## Data Availability

All the data are contained within the article or in Supplementary Material and they are also available on the Internet Platform AMC R2 (https://r2.amc.nl).

## Supplementary Materials

The following supporting information can be downloaded at: https://www.mdpi.com/article/10.3390/cancers15072035/s1, Genes Analysis of *AGAP11*, *IFIT2*, *PAPSS2*, *PCGF5*, and *NUDT9P1*, Figures S1–S84: Association between gene expression and NB patients’ survival and clinical characteristics for the analyzed genes other than *CCSER2*, Table S1: Demographic and clinical characteristics at diagnosis of the cohort of 260 neuroblastoma patients included in the analysis, Table S2: Association between chromosome 10q loss and other chromosomes losses in a cohort of 260 neuroblastoma patients with segmental chromosome alterations at diagnosis, Table S3: Association between chromosome 10q loss and chromosomes gains in a cohort of 260 neuroblastoma patients with segmental chromosome alterations at diagnosis, Tables S4–S83: Association between gene expression and NB patients’ survival for the analyzed genes other than *CCSER2*.
