# Supplementary material for: Multiple Genes with Potential Tumor Suppressive Activity Are Present on Chromosome 10q Loss in Neuroblastoma and Are Associated with Poor Prognosis
Source: Cancers (Basel). 2023 Mar 29;15(7):2035. doi: 10.3390/cancers15072035 (PMC10093755; doi:10.3390/cancers15072035)
Supplement: Supplementary file 1 [file cancers-15-02035-s001.zip › cancers-2286488-supplementary.pdf]

## Supplementary Material

# Multiple genes with potential tumor suppressive activity are present on chromosome 10q loss in neuroblastoma and are associated with poor prognosis

**Ognibene M. et al.**

## Supplementary Genes Analysis

*AGAP11*

We analyzed the survival of the cohort of 498 NB patients in the SEQC-498 database in relation to the expression levels of *AGAP11* gene. Overall survival was positively associated with the gene expression (5-year OS: 71.7%, 95% CI: 65.4 – 77.0 for expression values below the median, and 85.8%, 95% CI: 80.4 – 89.8 for expression values above the median, $p < 0.001$, Supplementary Figure S1A). A clear trend was observed after splitting the data on the basis of the tertile values (5-year OS: 64.9%, 95% CI: 56.8 – 71.9 for low expression values; 83.9%, 95% CI: 76.9 – 89.0 for intermediate values; and 87.3%, 95% CI: 80.8 – 91.8 for high values, $p <$ 0.001, Supplementary Figure S1B). Results from the analysis of EFS confirmed the pattern observed for OS. For instance, 5-year EFS was 56.2% (95% CI: 49.7 – 62.3) for gene expression values below the median, and 69.0% (95% CI: 62.7 – 74.5) for values above ($p = 0.002$, Supplementary Figure S1C). *S*plitting the data on the basis of the tertile values, 5-year EFS was 51.6% (95% CI: 43.5 – 59.0) for low expression levels, 64.7% (95% CI: 56.7 – 71.6) for intermediate levels, and 71.4% (95% CI: 63.8 – 77.8) for high values ($p < 0.001$, Supplementary Figure S1D). A statistically significant association was observed between high gene expression levels and each favorable prognostic factors (Figure 6), which included lower age at onset of disease ($p$=0.030, Supplementary Figure S2A), normal *MYCN* status ($p < 0.001$, Supplementary Figure S2B), and both localized and 4S stage ($p <$ 0.001, Supplementary Figure S2C).

Multivariable regression analysis model (Supplementary Table S4) confirmed the positive association between *AGAP11* expression values and OS. Adjusted HR obtained splitting the data on the median value was 0.62 (95% CI: 0.41 – 0.95, $p = 0.025$). OS analysis performed using the tertile values as cut-offs confirmed the positive observed trend (HR = 0.49, 0.30 – 0.82 for high vs. low expression levels, and HR = 0.64, 95%CI: 0.39 – 1.0, for intermediate vs. low levels, $p = 0.003$). With regard to EFS, a quite similar pattern was observed, but differences by group were less evident and statistical significance no longer reached. Analysis of linear trend, using the original continuous gene expression value, indicated a week protective effect both for OS and for EFS ($p$ = 0.072 and 0.064, respectively).

Results of the analysis of the *in silico* cohort of 283 NB patients in the NRC-283 database partly confirmed the positive association between *AGAP11* gene expression values and OS, but not PFS (Supplementary Figure S3). For instance, 5-year OS was 72.5% (95%CI: 63.1 – 79.9) for values above the median of gene expression and 62.2% (95%CI: 52.4 – 70.6) for values below, with a borderline statistical significance ($p = 0.067$). Splitting the data on the basis of the tertiles, OS was 75.9% (95%CI: 64.3 – 84.2) for high expression values, 64.9% (95%CI: 52.9 – 74.6) for intermediate values and 61.2% (95%CI: 48.9 – 71.4) for low values (p = 0.029).

Association between *AGAP11* expression levels and the major potential confounders in the NRC-283 data set showed that similar expression values were observed by age (Supplementary Figure S4A) and stage (Supplementary Figure S4C), whereas a lower expression level was associated with *MYCN* amplification ($p <$ 0.001, Supplementary Figure S4B).

After correction for confounding by multivariable Cox regression analysis, the association observed between OS and *AGAP11* expression values was reduced and no longer statistically significant (HR = 0.70, splitting the data set on the median value of gene expression, 95%CI: 0.43 – 1.1, $p = 0.145$, and HR = 0.88, 95%CI: 0.52 – 1.5 and HR = 0.66, 95%CI: 0.36 – 1.2, $p = 0.199$, Supplementary Table S5). No association emerged for the PFS.

*IFIT2*

We analyzed the survival of the cohort of 786 NB patients included in the Cangelosi *et al.* data set in relation to the expression levels of *IFIT2* gene. Overall survival was 79.2% (95%CI: 74.8 – 82.9) for expression values above the median, and 66.3% (95%CI: 61.4 – 70.8) for values below the median ($p$< 0.001, Supplementary Figure S5A). The association between higher expression levels and better OS was confirmed by splitting the cohort into three groups on the tertile values of gene expression (OS = 79%, 95%CI: 73.6 – 83.5, OS = 77.5%, 95%CI: 71.9 – 82.1, and OS = 61.8%, 95%CI: 55.7 – 67.4, $p$ < 0.001, Supplementary Figure S5B). A similar pattern was observed for EFS. For instance, EFS was 62.9% (95%CI: 57.9 – 67.5) above the median and 55.6% below (95%CI: 50.4 – 60.4, $p$ = 0.010, Supplementary Figure S5C). Splitting the data on the tertile values the corresponding figures were: 64.5% (95%CI: 58.3 – 70.0), 62.3% (95%CI: 56.1 – 67.9), and 50.8% (95%CI: 44.4 – 56.8), $p$ < 0.001 (Supplementary Figure S5D).

The association between *IFIT2* expression values and the main available potential confounders (age, *MYCN* status and stage) showed that a slight not statistically significant association was observed with age at diagnosis (Supplementary Figure S6A), while higher expression values were associated with normal *MYCN* status ($p$ < 0.001, Supplementary Figure S6B) and localized and stage 4S disease ($p$ < 0.001, Supplementary Figure S6C).

The association between high *IFIT2* expression levels and better OS was confirmed in multivariable analysis splitting the data on the median value (HR = 0.70, 95%CI: 0.54 – 0.92, $p$ = 0.010, Supplementary Table S6). However, in the analysis stratified by tertile values, the trend was no longer evident. With regard to EFS, the association with *IFIT2* expression was reduced and no longer significant in multivariable analysis after splitting the cohort both on the median and on the tertile levels. However, a statistically significant linear trend was observed for both OS (HR = 0.87, 95%CI: 0.75 – 0.99, $p$ = 0.041) and EFS (HR = 0.89, 95%CI: 0.78 – 1.0, $p$ = 0.048) using the original continuous values of gene expression.

Validation analysis on the cohort of 283 NB patients in the NRC-283 database found a slightly better OS for patients with *IFIT2* expression values above the median (OS = 61.0%, 95%CI: 51.1 – 69.5, vs. 73.8%, 95%CI: 64.4 – 81.1, $p$ = 0.038, Supplementary Figure S7A). This association was confirmed by splitting the data set into three groups using the tertile expression values (low levels: OS = 50.9%, 95%CI: 38.2 – 62.2; intermediate levels: 70.8%, 95%CI: 58.6 – 80.0; high levels: 78.9%, 95%CI: 68.2 – 86.4, $p$ = 0.009, Supplementary Figure S7B). PFS analysis did not find a significant difference between high and low expression values by splitting the data on the median (Supplementary Figure S7C), but confirmed a positive association between *IFIT2* expression and survival stratifying the patients on the tertile values (PFS = 42.9%, 95%CI: 31.3 – 54.1 for low values, 61.6%, 95%CI: 49.5 – 71.6 for intermediate value, and 73.9%, 95%CI: 63.1 – 82.0, for high values, respectively, $p$ = 0.021, Supplementary Figure S7D).

The association between *IFIT2* expression and the available potential confounders showed that higher values were observed among patients with normal *MYCN* status ($p$ < 0.001, Supplementary Figure S8B), whereas expression levels were similar between different groups of age and stage at diagnosis.

Multivariable analysis via Cox regression model showed that, after adjusting for the potential effect of the available confounders, the apparent protective role of high levels of *IFIT2* gene expression was no longer observed (Supplementary Table S7).

*PAPSS2*

We analyzed the survival of the cohort of 786 NB patients included in the Cangelosi *et al.* data set in relation to the expression levels of *PAPSS2* gene. Overall survival was positively associated with the gene expression (OS = 81.4%, 95%CI: 77.2 – 84.9 for values above the median and OS = 64.1%, 95%CI: 59.2 – 68.6, for values below the median, $p$ < 0.001, Supplementary Figure S9A). Evidence of trend emerged splitting the data on the base of the tertile values (OS = 83.6%, 95%CI: 78.5 – 87.6, 77.5%, 95%CI: 71.9 – 82.1, and 57.3%, 95%CI: 51.0 – 63.0, respectively, $p$ < 0.001, Supplementary Figure S9B). A better EFS was also observed in relation with higher

gene expression levels (EFS = 64.7%, 95%CI: 59.7 – 69.2 for values above the median and 53.8%, 95%CI: 48.7 – 58.7, for values below the median, $p = 0.003$, Supplementary Figure S9C). This association was confirmed after splitting the data on the basis of the tertile of expression values (EFS = 64.5%, 95%CI: 58.3 – 70.0, 63.7%, 95%CI: 57.5 – 69.2, and 49.4%, 95%CI: 43.1 – 55.4, $p < 0.001$, Supplementary Figure S9D).

*PAPSS2* expression was associated with favorable prognostic features which included lower age at diagnosis ($p < 0.001$, Supplementary Figure S10A), normal *MYCN* status ($p < 0.001$, Supplementary Figure S10B) and localized/stage 4S disease ($p < 0.001$, Supplementary Figure S10C).

Multivariable Cox regression analysis confirmed the association between OS and *PAPSS2* expression values when the cohort was split into three groups using the tertiles values as cut-offs (HR = 0.79, 95%CI: 0.58 – 1.1, and HR = 0.67, 95%CI: 0.48 – 0.94, $p = 0.015$, Supplementary Table S8). This trend was confirmed using the original continuous variable as a predictor (HR = 0.86, 95%CI: 0.74 – 0.99, $p = 0.029$). In multivariable analysis the association between higher expression values and a better EFS was not confirmed.

A positive association between the expression of *PAPSS2* and patient survival was observed in the analysis of the NRC-283 NB cohort for both OS and PFS. For instance, OS above the median gene expression value was 74.9% (95%CI: 65.8 – 81.9) vs. 59.5% (95%CI: 49.3 – 68.2) below the median value ($p = 0.025$, Supplementary Figure S11A). A positive trend in OS was observed stratifying the data on the tertile of gene expression (55.5%, 95%CI: 42.9 – 66.3 for low values, 69.8%, 95%CI: 57.9 – 79.0 for intermediate values, and 76.6%, 95%CI: 65.4 – 84.6 for high values, respectively, $p = 0.014$, Supplementary Figure S11B). A similar pattern was observed for PFS, both stratifying on the median value (PFS = 52.3%, 95%CI: 42.8 – 61.0 and 67.8%, 95%CI: 58.4 – 75.5 for values above and below the median expression level, respectively, $p = 0.009$, Supplementary Figure S11C), and splitting the data on the tertile values (49.6%, 95%CI: 38.0 – 60.2, 63.1%, 95%CI: 51.5 – 72.7, and 67.7%, 95%CI: 56.0 – 77.0, respectively, $p = 0.015$, Supplementary Figure S11D).

The association between *PAPSS2* gene expression levels and the available potential confounders showed that higher expression values were observed in younger patients ($p = 0.001$, Supplementary Figure S12A), while no statistically significant difference was observed comparing patients with normal *MYCN* status with those with *MYCN* amplification ($p = 0.078$, Supplementary Figure S12B) and comparing different stage at diagnosis ($p = 0.078$, Supplementary Figure S12C).

The potential protective role of *PAPSS2* on patient survival, indicated by results of the univariable analysis, was not confirmed after adjustment for confounding in multivariable analysis via Cox regression model, for either OS or PFS (Supplementary Table S9).

*PCGF5*

We analyzed the survival of 786 NB patients included in the Cangelosi *et al.* data set in relation to the expression of *PCGF5* gene. A better OS was associated with higher expression values splitting the data both on the median (OS = 85.0%, 95%CI: 81.1 – 88.2, vs. 60.6%, 95%CI: 55.5 – 65.2, $p < 0.001$, Supplementary Figure S13A), and on the tertiles of gene expression values (OS = 87.0%, 95%CI: 82.3 – 90.6, 77.1%, 95%CI: 71.5 – 81.7, 54.2%, 95%CI: 48.0 – 60.0, $p < 0.001$, Supplementary Figure S13B). A similar pattern was observed for EFS (EFS = 70.0%, 95%CI: 65.2 – 74.3 vs. 48.3%, 95%CI: 43.2 – 53.2, splitting the data on the median value, $p < 0.001$, Supplementary Figure S13C; EFS = 70.5%, 95%CI: 64.6 – 75.6, 62.9%, 95%CI: 56.7 – 68.5, 43.8%, 95%CI: 37.6 – 49.8, splitting the data on the basis of the tertiles, $p < 0.001$, Supplementary Figure S13D).

The association between *PCGF5* expression levels and the available patients' prognostic characteristics showed that higher values were associated with a lower age at diagnosis ($p < 0.001$, Supplementary Figure S14A), normal *MYCN* status ($p < 0.001$, Supplementary Figure S14B) and localized or stage 4S disease ($p < 0.001$, Supplementary Figure S14C).

Multivariable regression analysis confirmed the positive association between survival and *PCGF5* expression (Supplementary Table S10). Splitting the data on the median value an HR = 0.56 was observed for OS (95%CI: 0.42 – 0.76, $p < 0.001$), and a clear trend emerged splitting the cohort into three groups using the tertiles (HR =

0.71, 95%CI: 0.52 – 0.97, and HR = 0.48, 95%CI: 0.33 – 0.69, $p < 0.001$). This trend was confirmed using the original expression values on a continuous scale (HR = 0.79, 95%CI: 0.66 – 0.93, $p = 0.006$). A similar pattern was observed for EFS, both splitting the data on the median value (HR= 0.67, 95%CI: 0.53 – 0.85, $p < 0.001$), and stratifying on the tertiles (HR = 0.82, 95%CI: 0.63 – 1.1, and HR = 0.66, 95%CI: 0.50 – 0.89, p = 0.006) and was also confirmed using the original values as a continuous predictor (HR = 0.85, 95%CI: 0.72 – 1.0, $p = 0.047$).

The association between *PCGF5* expression levels and patient survival in the validation cohort NRC-283 showed that higher values of gene expression were associated with a better survival both for OS and for EFS. In details, splitting the data on the median expression, OS was 57.2% (95%CI: 47.2 – 65.9) for low values and 77.5 (95%CI: 68.4 – 84.3) for high values ($p < 0.001$, Supplementary Figure S15A). Stratifying the cohort by the tertile values, OS was 46.7% (95%CI: 34.7 – 57.8) for low levels, 75.9% (95%CI: 64.2 – 84.2) for intermediate levels, and 79.7% (95%CI: 68.5 – 87.3) for high levels, respectively ($p < 0.001$, Supplementary Figure S15B). A quite similar pattern was observed for PFS. The corresponding figures were 53.3% (95%CI: 43.6 – 62.0) below the median, and 66.5% (95%CI: 57.2 – 74.3) above the median, respectively ($p = 0.048$, Supplementary Figure S15C). In patients with *PCGF5* expression values in the third tertile PFS was 43.6% (95%CI: 32.2 – 54.4), in the second tertile was 64.8% (95%CI: 53.0 – 74.3), and in the first tertile 71.4% (95%CI: 59.8 – 80.2, $p = 0.005$ , Supplementary Figure S15D).

*PCGF5* expression levels were positively associated with favorable prognostic features at diagnosis (Figure 28), including lower age ($p = 0.003$, Supplementary Figure S16A), normal *MYCN* status ($p < 0.001$, Supplementary Figure S16B) and localized or stage 4S stage ($p = 0.023$, Supplementary Figure S16C).

Results of the analysis by multivariable Cox regression model indicated that the potential protective effect observed in univariable analysis was mainly due to confounding by the main prognostic patient characteristics both for OS and for PFS (Supplementary Table S11).

*NUDT9P1*

We analyzed the survival of the cohort of 498 NB patients from the SEQC-498 database in relation to the expression levels of *NUDT9P1* gene. OS was positively associated with the gene expression both splitting the data on the median (OS = 90.3%, 95%CI: 85.7 – 93.5, vs. 66.7%, 95%CI: 60.1 – 72.5, $p < 0.001$, Supplementary Figure S17A), and using the tertile values as cut-offs (OS = 92.8%, 95%CI: 87.3 – 95.9, 85.8%, 95%CI: 79.2 – 90.4, 56.0%, 95%CI: 47.4 – 63.7, $p < 0.001$, Supplementary Figure S17B). A similar pattern was observed for EFS (74.9%, 95%CI: 68.9 – 79.9 vs. 49.9%, 95%CI: 43.3 – 56.2, splitting the data on the median expression value, $p < 0.001$, Supplementary Figure S17C; EFS = 77.1%, 95%CI: 69.9 – 82.7, 66.5%, 95%CI: 58.5 – 73.3, 43.3%, 95%CI: 35.4 – 51.0, splitting the data on the tertiles, $p < 0.001$, Supplementary Figure S17D).

The association between *NUDT9P1* expression levels and the available patients' prognostic characteristics showed that higher values were associated with a lower age at diagnosis ($p = 0.001$, Supplementary Figure S18A), normal *MYCN* status ($p < 0.001$, Supplementary Figure S18B) and localized or stage 4S disease ($p < 0.001$, Supplementary Figure S18C).

Multivariable regression analysis confirmed the positive association between patients' survival and *NUTD9P1* expression values (Supplementary Table S12). Splitting the data on the median, an HR = 0.36 was observed for OS (95%CI: 0.23 – 0.58, $p < 0.001$). A clear trend emerged splitting the cohort into three groups using the tertiles (HR = 0.56, 95%CI: 0.34 – 0.92, and HR = 0.24, 95%CI: 0.13 – 0.44, respectively, $p < 0.001$). This trend was confirmed analyzing the original expression values on a continuous scale (HR = 0.06, 95%CI: 0.02 – 0.20, $p < 0.001$). A similar pattern was observed for EFS. Splitting the data on the median value, HR was 0.52 (95%CI: 0.38 – 0.72, $p < 0.001$). A clear trend was observed splitting the cohort on the tertile values (HR = 0.71, 95%CI: 0.49 – 1.0, and HR = 0.41, 95%CI: 0.28 – 0.62, $p < 0.001$) and was confirmed using the original values as a continuous predictor (HR = 0.19, 95%CI: 0.08 – 0.43, $p < 0.001$).

We analyzed the association between *NUDT9P1* expression levels and patient survival in the validation cohort NRC-283. For OS, higher values of gene expression were associated with a better survival both splitting the

data on the median value (56.3%, 95%CI: 46.5 – 65.1, vs. 78.4%, 95%CI: 69.4 – 85.1, $p < 0.001$, Supplementary Figure S19A) and on the tertiles (49.9% 95%CI: 37.8 – 60.7 for low values, 75.6%, 95%CI: 63.9 – 83.9, for intermediate values, and 77.0%, 95%CI: 65.4 – 85.1, $p < 0.001$, Supplementary Figure S19B). With regard to PFS, results were consistent with those obtained for OS. PFS for patients with *NUDT9P1* expression levels above the median was 48.6% (95%CI: 39.2 – 57.4) vs. 71.4% (95%CI: 62.2 – 78.7) for patients with values above ($p <$ 0.001, Supplementary Figure S19C). Stratifying the cohort on the tertile values PFS was 42.5%, 95%CI: 31.2 – 53.4) for low values, 65.3% (95%CI: 53.5 – 74.8) for intermediate values, and 72.3% (95%CI: 60.9 – 80.9) for high values, respectively ($p < 0.001$, Supplementary Figure S19D).

*NUDT9P1* expression was associated with favorable patient characteristics at diagnosis. Higher values were observed for younger patients ($p = 0.036$, Supplementary Figure S20A), with normal *MYCN* status ($p < 0.001$, Supplementary Figure S20B), and localized stage or stage 4S ($p = 0.001$, Supplementary Figure S20C).

Correcting for confounding in multivariable Cox regression analysis, the association between high levels of *NUDT9P1* expression values observed in univariable analysis was reduced and no longer statistically significant (Supplementary Table S13) for both OS and PFS.

# Supplementary Figures

## Supplementary Figure S1

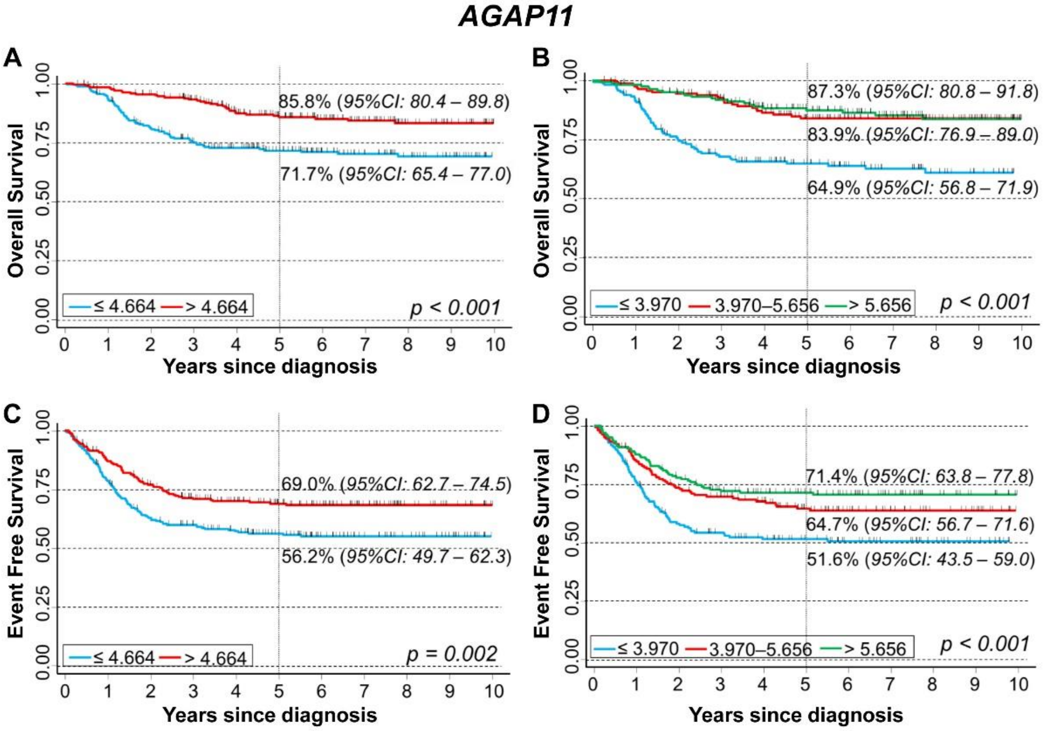


Survival analyses of 498 NB patients using the SEQC-498 dataset for *AGAP11* gene expression. Overall survival, cut-off on the median (**A**) or tertile (**B**) expression value; Event free survival, cut-off on the median (**C**) or tertile (**D**) expression value. Five-year survival estimates and the related 95% CI are shown

## Supplementary Figure S2

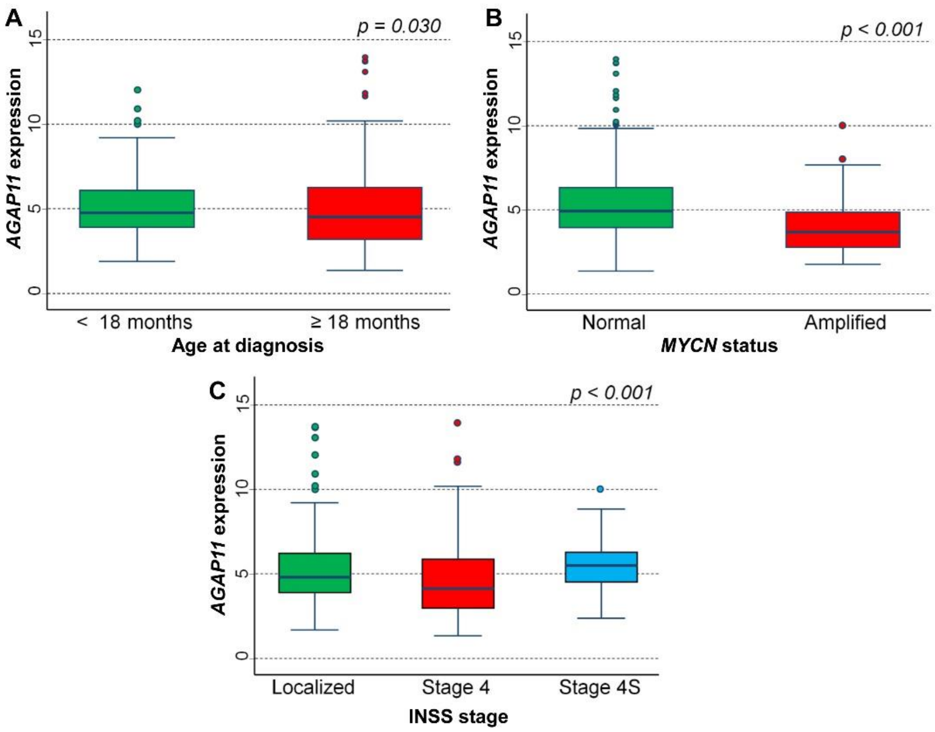


Association between *AGAP11* gene expression and the main patient characteristics at diagnosis evaluated on the cohort of 498 neuroblastoma patients in the SEQC-498 data set. **(A)** Age; **(B)** *MYCN* status; **(C)** INSS stage.

**Supplementary Figure S3**

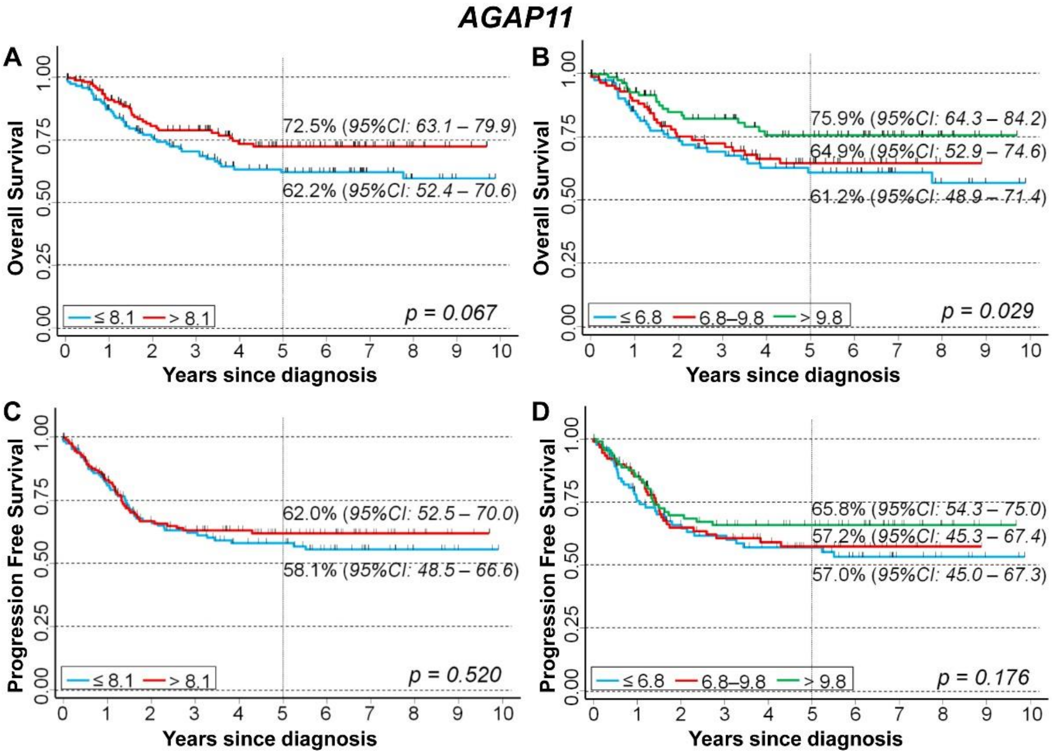


Survival analysis of 283 NB patients using the NRC-283 dataset for *AGAP11* gene expression. Overall survival, cut-off on the median (**A**) or tertile (**B**) expression value; Progression free survival, cut-off based on the median (**C**) or tertile (**D**) expression value. Five-year survival estimates and their related 95% CI are shown.

**Supplementary Figure S4**

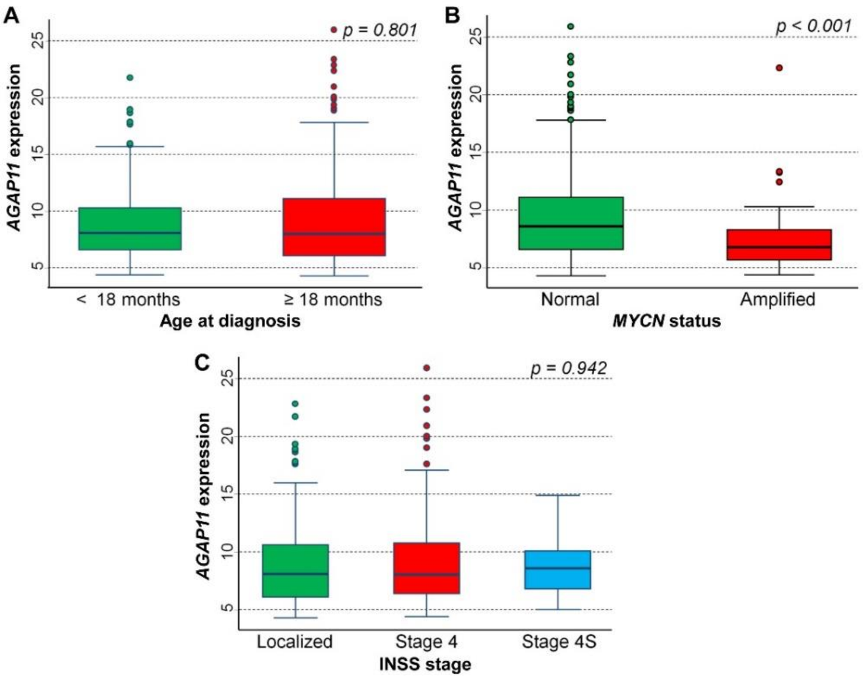


Association between *AGAP11* gene expression and the main patient characteristics at diagnosis evaluated on the cohort of 283 neuroblastoma patients in the NRC-283 data set. (**A**) Age; (**B**) *MYCN* status; (**C**) INSS stage.

**Supplementary Figure S5**

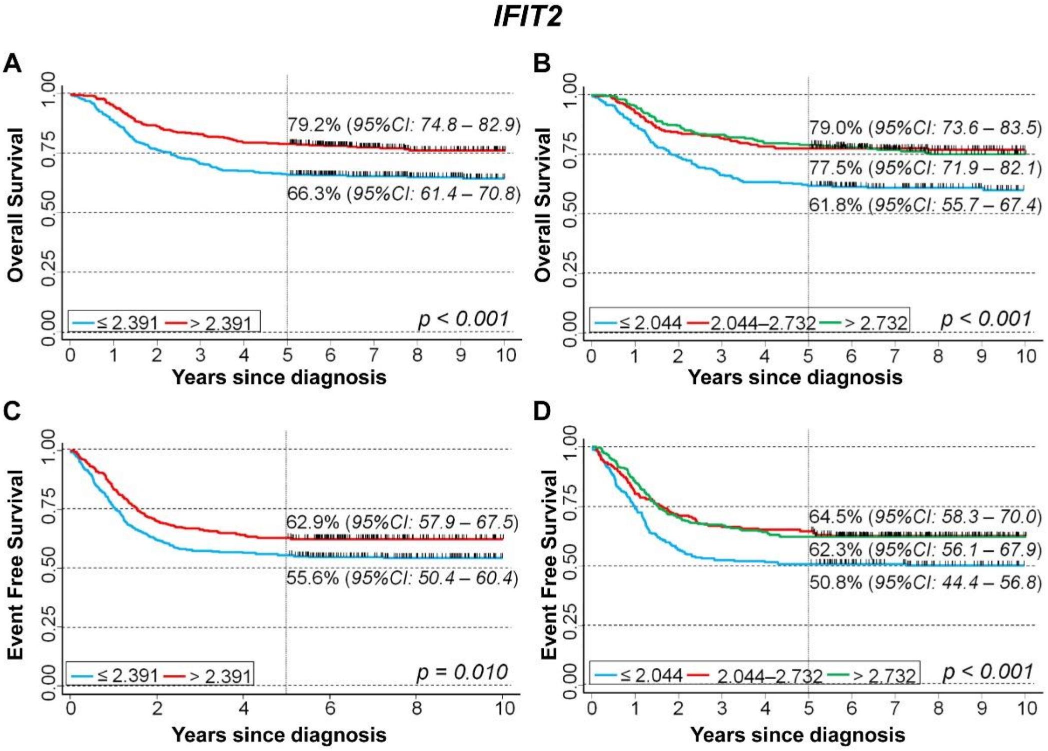


Survival analyses of 786 NB patients using the Cangelosi et al. dataset for *IFIT2* gene expression. Overall survival, cut-off on the median (**A**) or tertile (**B**) expression value; Event free survival, cut-off on the median (**C**) or tertile (**D**) expression value. Five-year survival estimates and their related 95% CI are shown.

**Supplementary Figure S6**

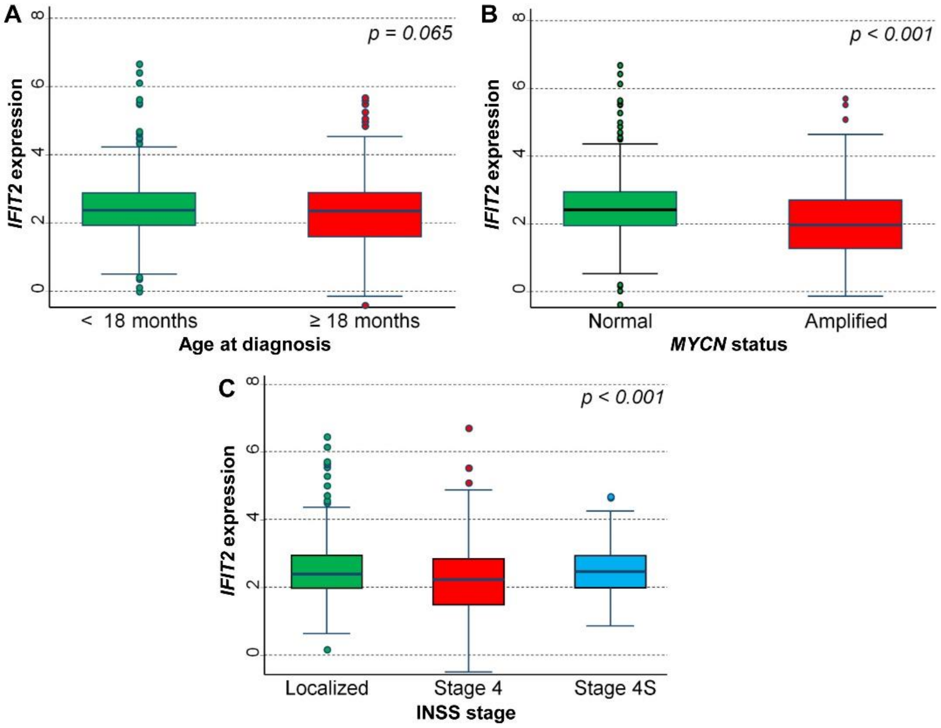


Association between *IFIT2* gene expression and the main patient characteristics at diagnosis evaluated on the cohort of 786 neuroblastoma patients in the data set of Cangelosi *et al.* (**A**) Age; (**B**) *MYCN* status; (**C**) INSS stage.

**Supplementary Figure S7**

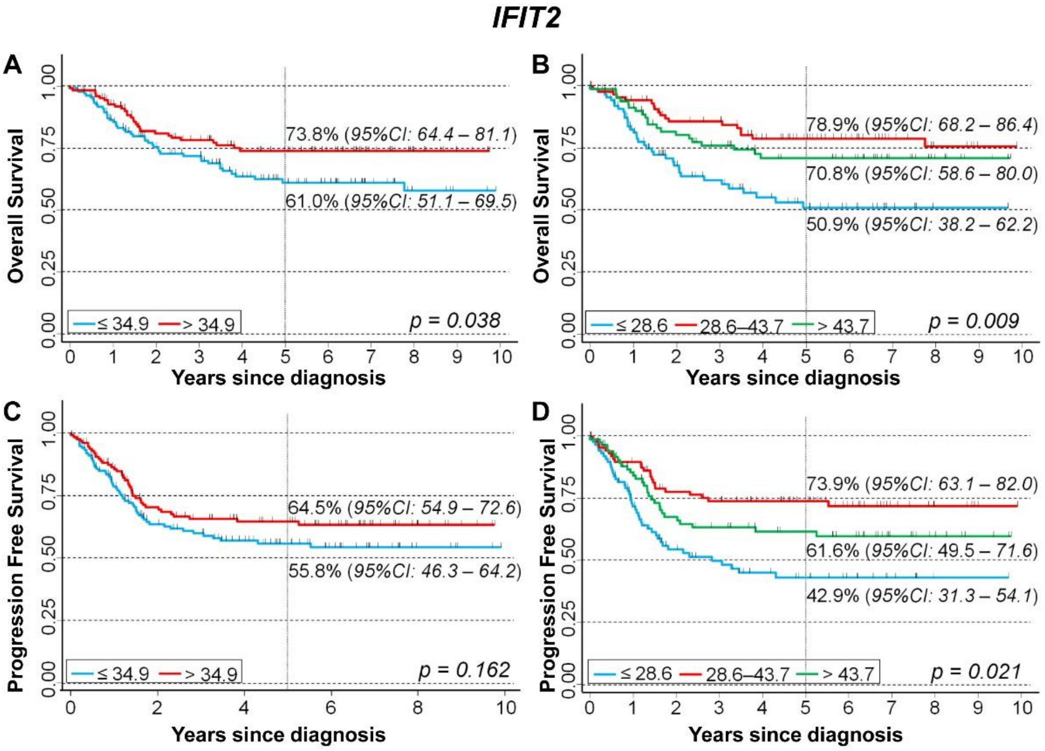


Survival analyses of 283 NB patients using the NRC-283 dataset for *IFIT21* gene expression. Overall survival, cut-off on the median (**A**) or tertile (**B**) expression value; Progression free survival, cut-off on the median (**C**) or tertile (**D**) expression value. Five-year survival estimates and their related 95% CI are shown.

**Supplementary Figure S8**

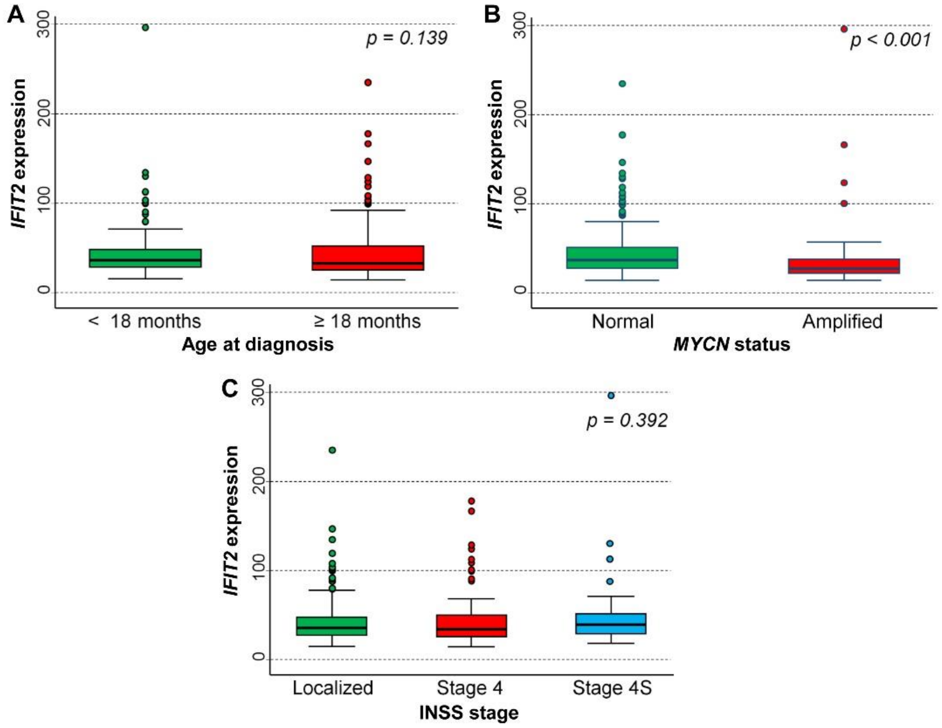


Association between *IFIT2* gene expression and the main patient characteristics at diagnosis evaluated on the cohort of 283 NB patients in the NRC-283 data set. (**A**) Age; (**B**) *MYCN* status; (**C**) INSS stage.

**Supplementary Figure S9**

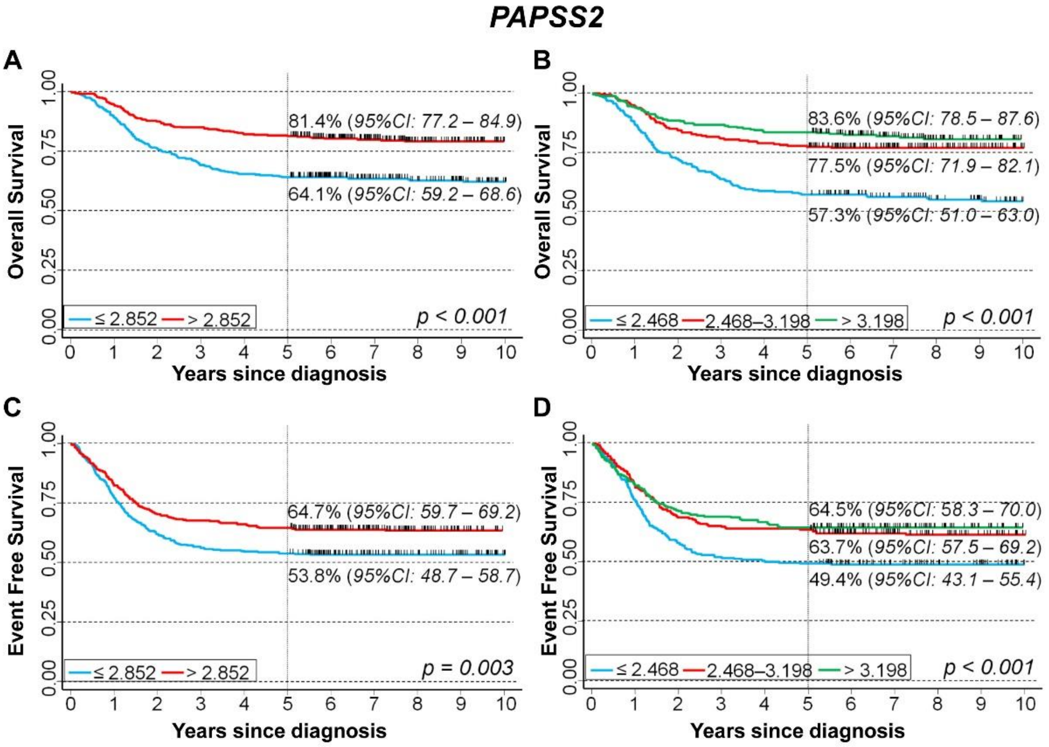


Survival analyses of 786 NB patients using the Cangelosi et al. dataset for *PAPSS2* gene expression. Overall survival, cut-off on the median (**A**) or tertile (**B**) expression value; Event free survival, cut-off on the median (**C**) or tertile (**D**) expression value. Five-year survival estimates and their related 95% CI are shown.

**Supplementary Figure S10**

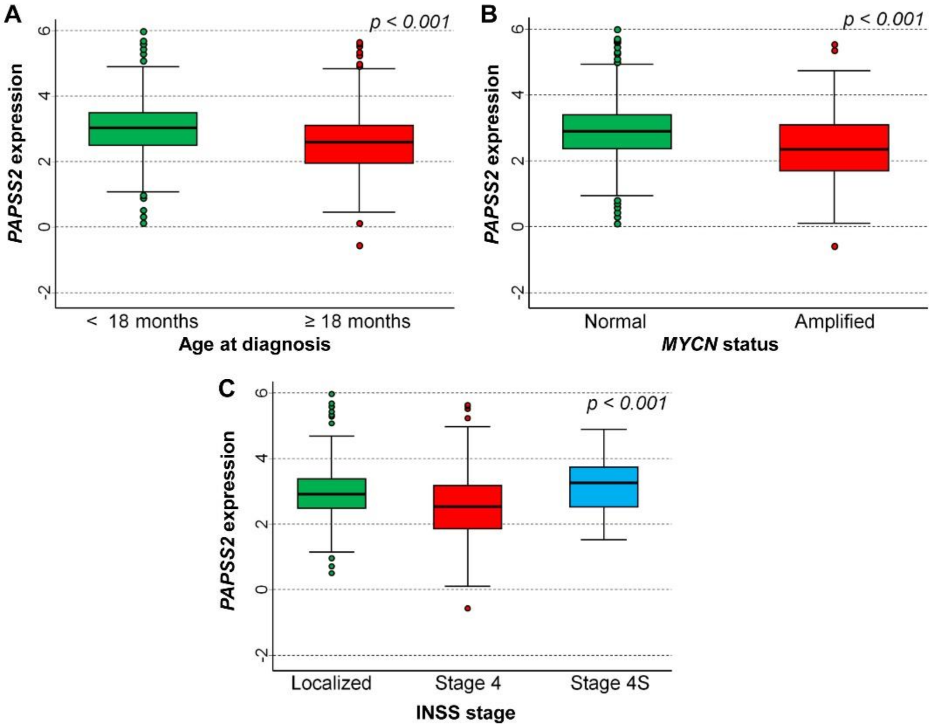


Association between *PAPSS2* gene expression and the main patient characteristics at diagnosis evaluated on the cohort of 786 neuroblastoma patients in the data set of Cangelosi *et al.* (**A**) Age; (**B**) *MYCN* status; (**C**) INSS stage.

**Supplementary Figure S11**

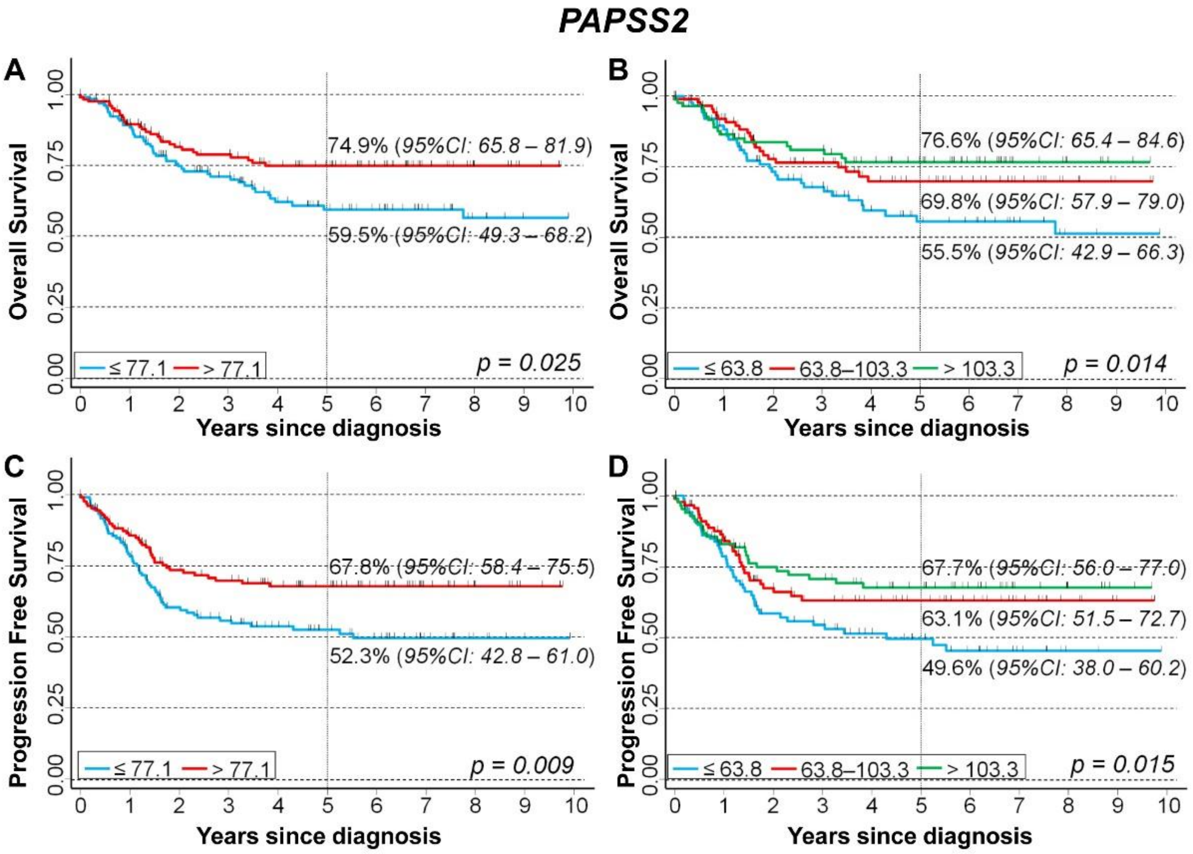


Survival analyses of 283 NB patients using the NRC-283 dataset for *PAPSS2* gene expression. Overall survival, cut-off on the median (**A**) or tertile (**B**) expression value; Progression free survival, cut-off on the median (**C**) or tertile (**D**) expression value. Five-year survival estimates and their related 95% CI are shown.

**Supplementary Figure S12**

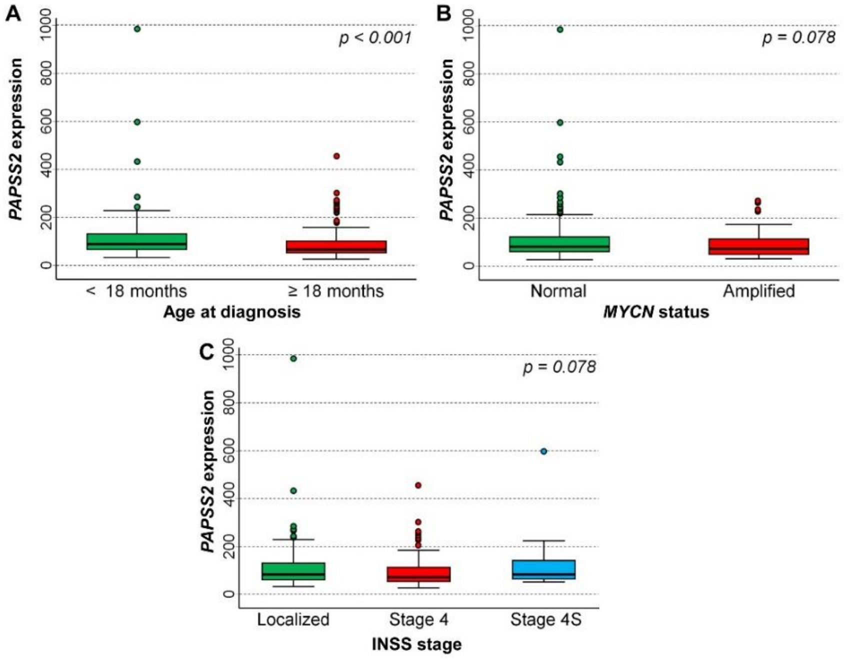


Association between *PAPSS2* gene expression and the main patient characteristics at diagnosis evaluated on the cohort of 498 neuroblastoma patients in the SEQC-498 data set. (**A**) Age; (**B**) *MYCN* status; (**C**) INSS stage.

**Supplementary Figure S13**

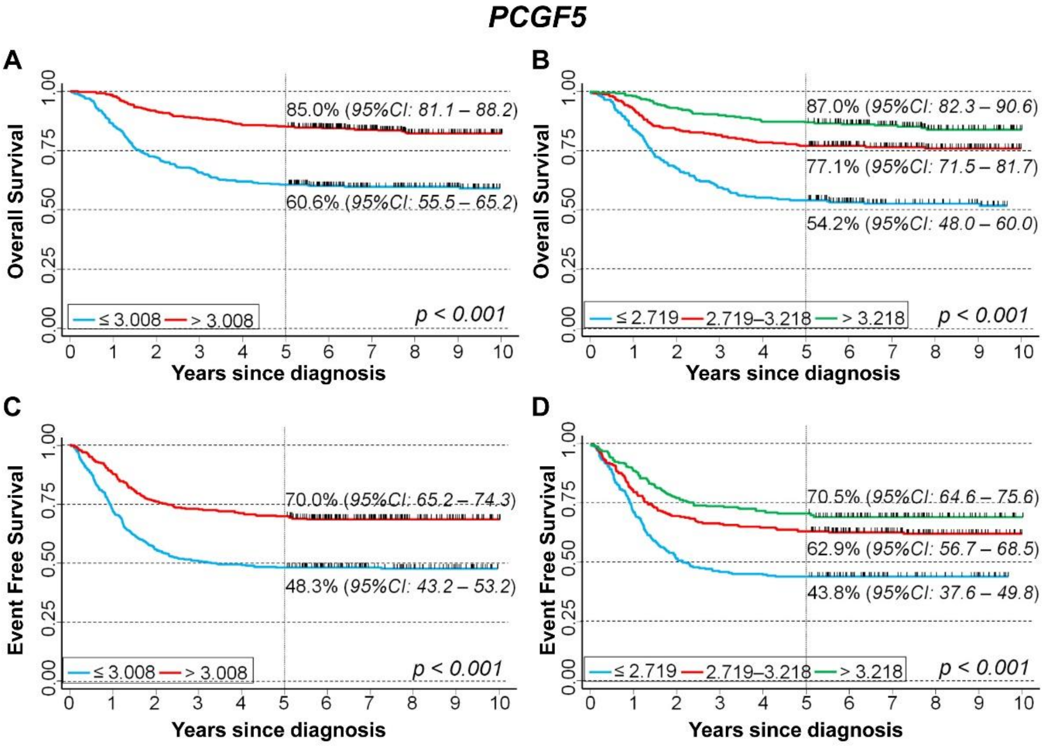


Survival analyses of 786 NB patients using the Cangelosi et al. dataset for *PCGF5* gene expression. Overall survival, cut-off on the median (**A**) or tertile (**B**) expression value; Event free survival, cut-off on the median (**C**) or tertile (**D**) expression value. Five-year survival estimates and their related 95% CI are shown.

**Supplementary Figure S14**

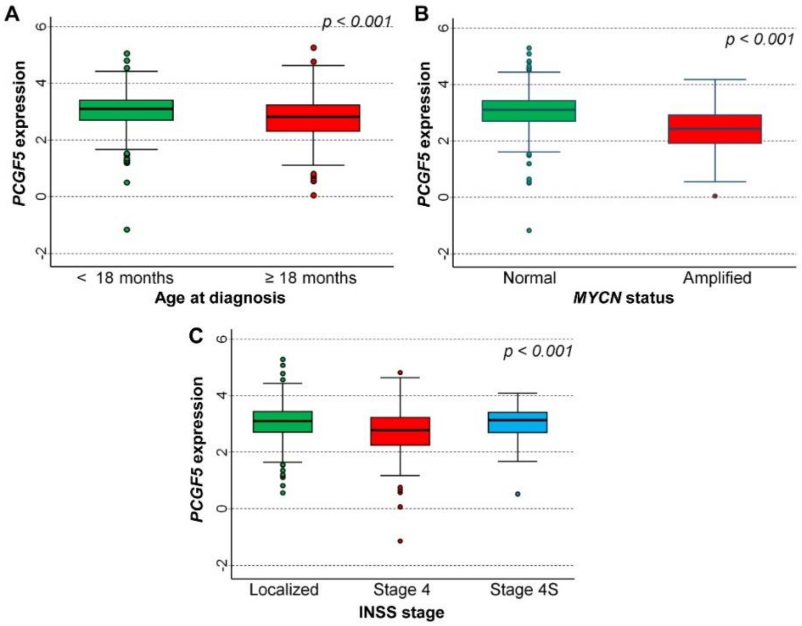


Association between *PCGF5* gene expression and the main patient characteristics at diagnosis evaluated on the cohort of 786 neuroblastoma patients in the data set of Cangelosi *et al.* (**A**) Age; (**B**) *MYCN* status; (**C**) INSS stage.

**Supplementary Figure S15**

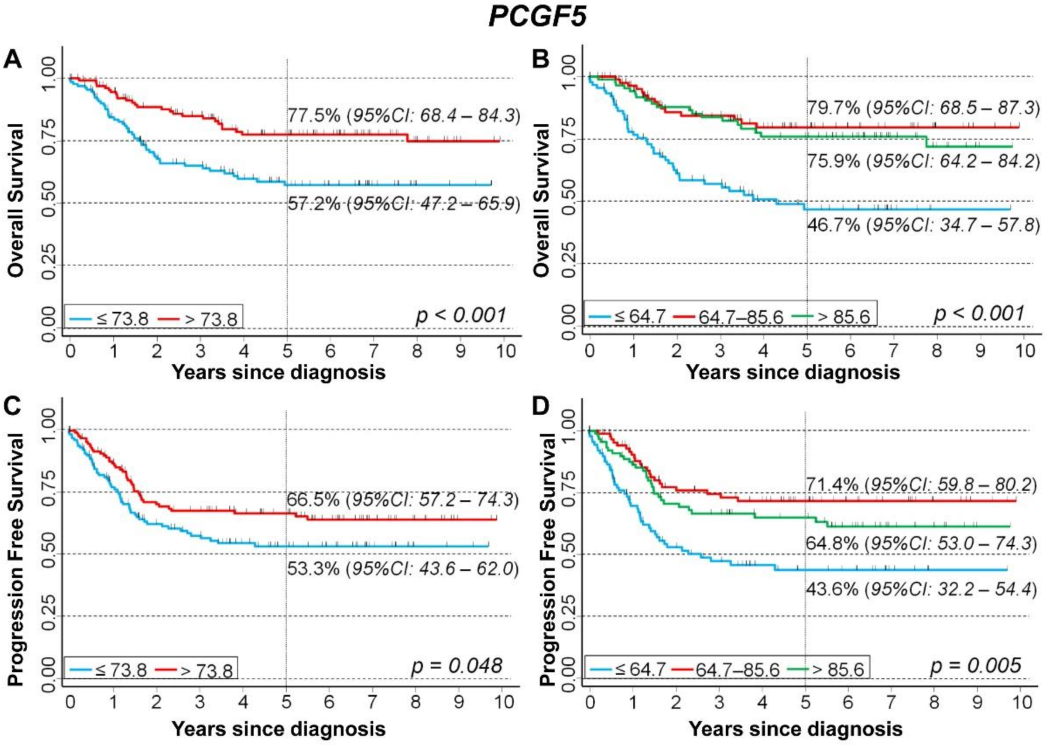


Survival analyses of 283 NB patients using the NRC-283 dataset for *PCGF5* gene expression. Overall survival, cut-off on the median (**A**) or tertile (**B**) expression value; Progression free survival, cut-off on the median (**C**) or tertile (**D**) expression value. Five-year survival estimates and their related 95% CI are shown.

**Supplementary Figure S16**

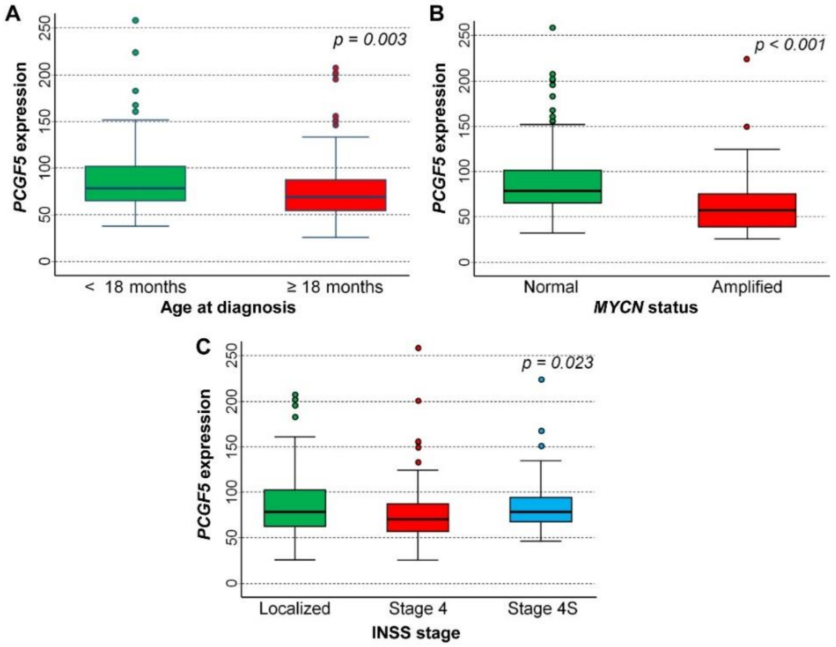


Association between *PCGF5* gene expression and the main patient characteristics at diagnosis evaluated on the cohort of 498 neuroblastoma patients in the SEQC-498 data set. (**A**) Age; (**B**) *MYCN* status; (**C**) INSS stage.

**Supplementary Figure S17**

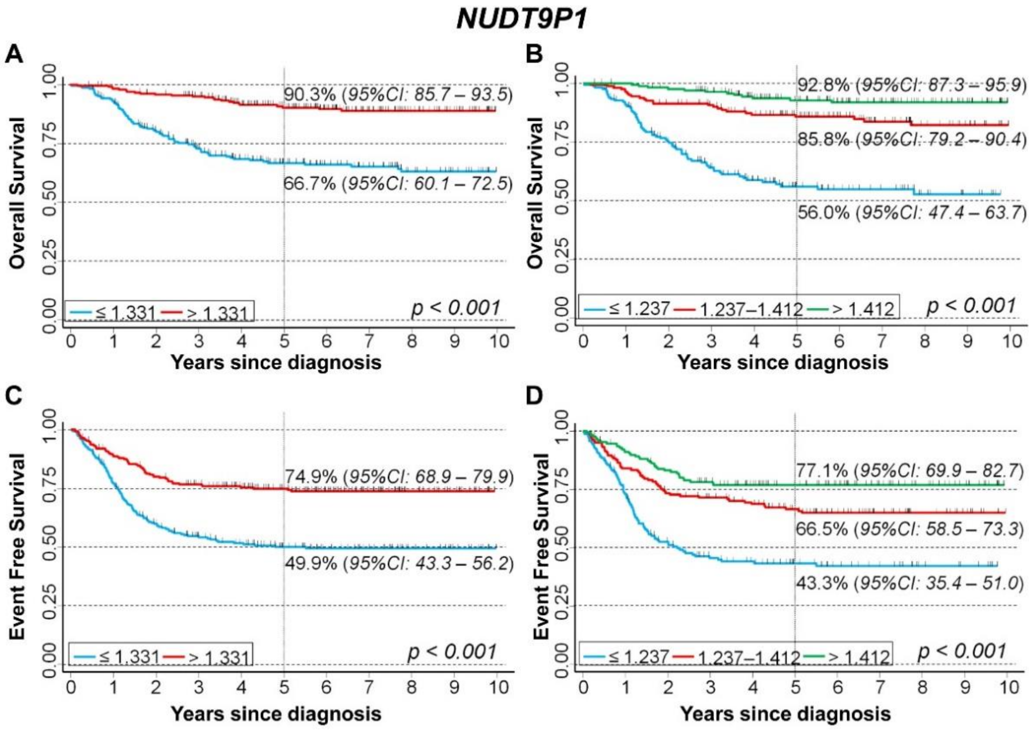


Survival analyses of 498 NB patients using the SEQC-498 data set for *NUDT9P1* gene expression Overall survival, cut-off on the median (**A**) or tertile (**B**) expression value; Event free survival, cut-off on the median (**C**) or tertile (**D**) expression value. Five-year survival estimates and their related 95% CI are shown.

**Supplementary Figure S18**

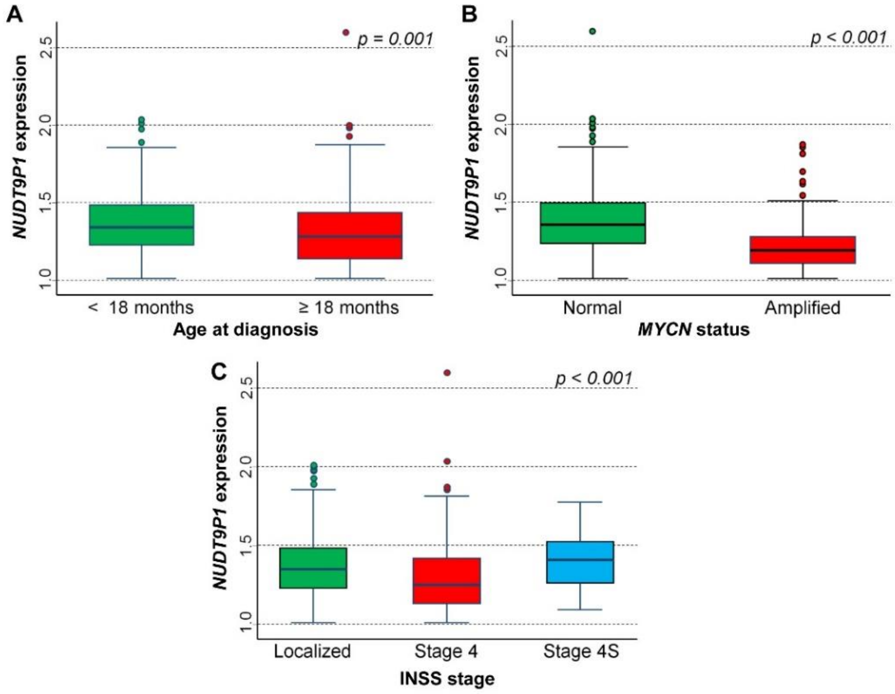


Association between *NUDT9P1* gene expression and the main patient characteristics at diagnosis evaluated on the cohort of 498 neuroblastoma patients in the SEQC-498 data set. (**A**) Age; (**B**) *MYCN* status; (**C**) INSS stage.

**Supplementary Figure S19**

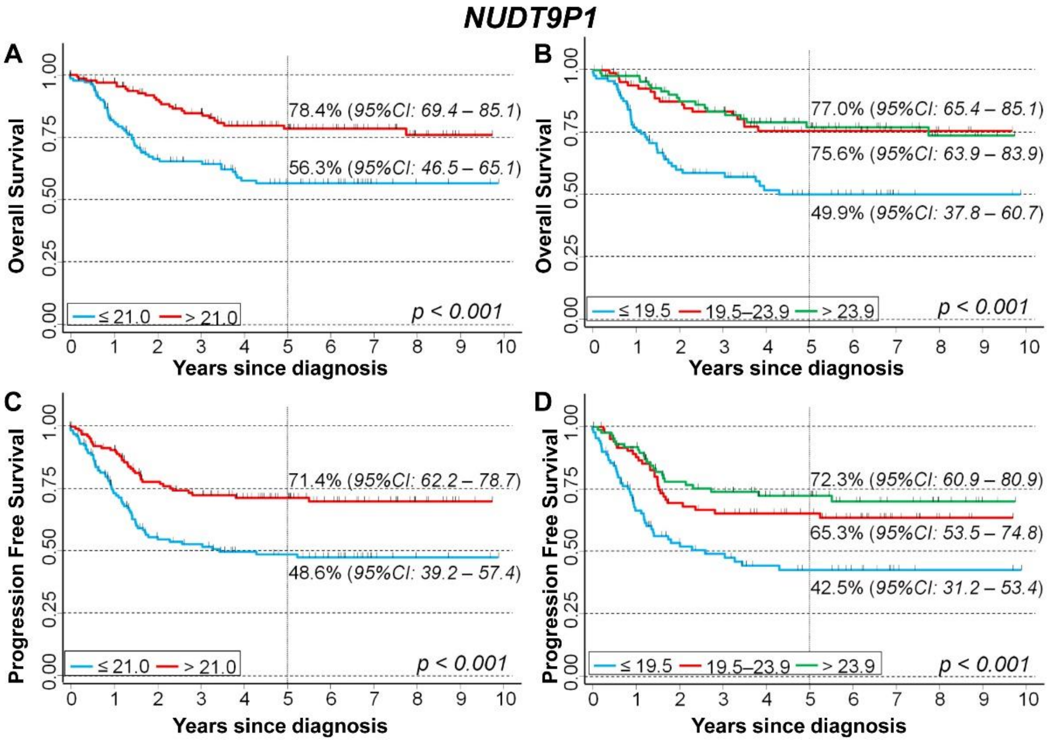


Survival analyses of 283 NB patients using the NRC-283 dataset for *NUDT9P1* gene expression. Overall survival, cut-off on the median(**A**) or tertile (**B**) expression value; Progression free survival, cut-off on the median (**C**) or tertile (**D**) expression value. Five-year survival estimates and their related 95% CI are shown.

**Supplementary Figure S20**

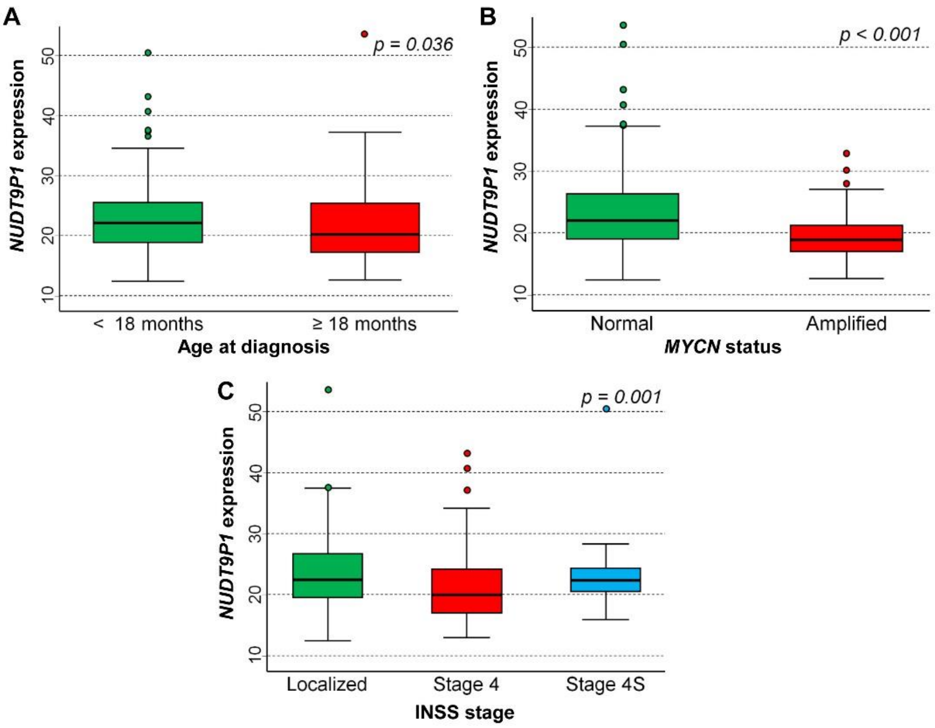


Association between *NUDT9P1* gene expression and the main patient characteristics at diagnosis evaluated on the cohort of 283 neuroblastoma patients in the NRC-283 data set. (**A**) Age; (**B**) *MYCN* status; (**C**) INSS stage.

## Supplementary Figure S21

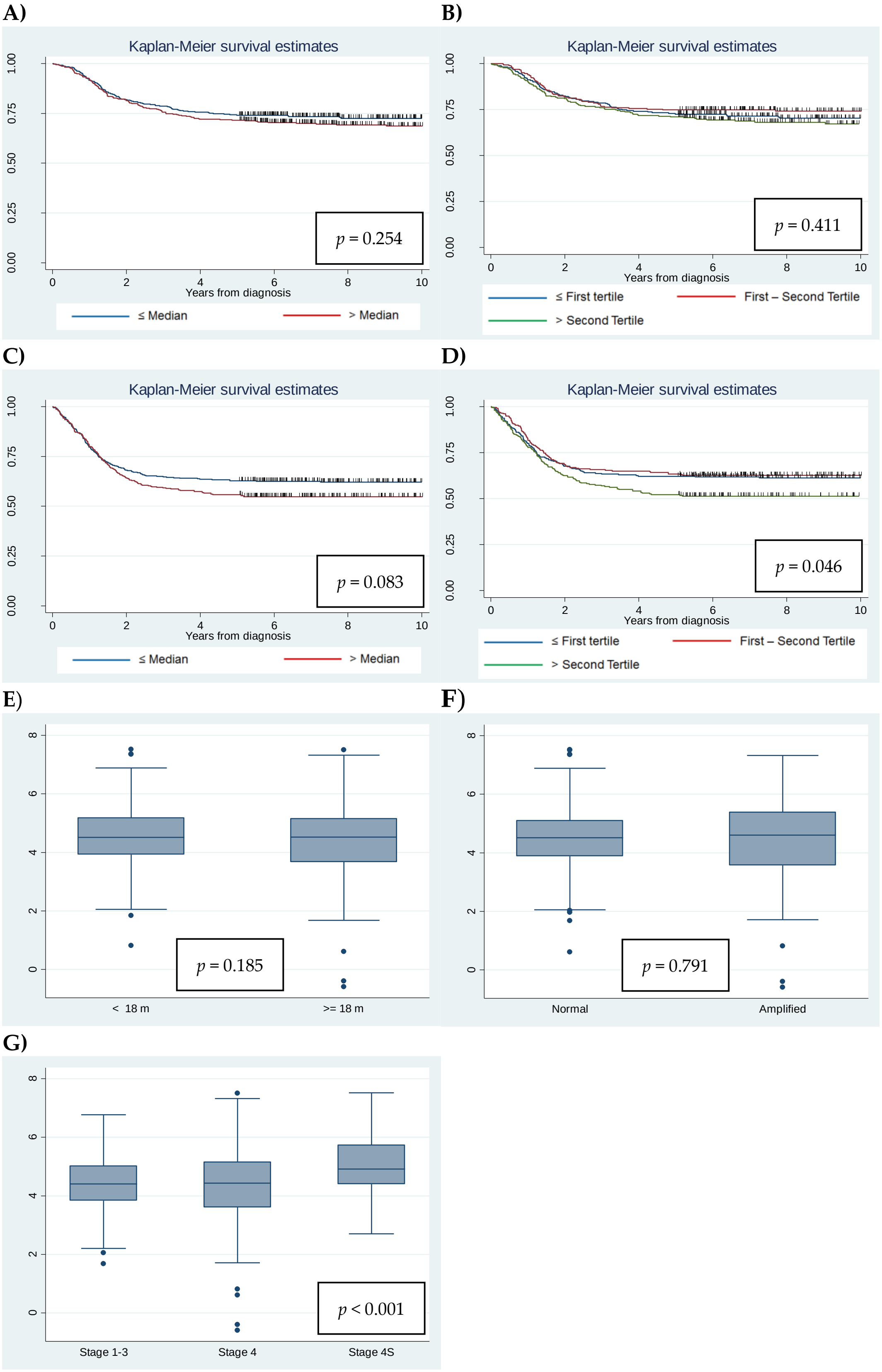


*ACTA2* gene expression and its association with survival and clinical characteristics in a cohort of 786 NB patients in the Cangelosi et al. *in silico* dataset. (A) Overall survival, cut-off based on the median expression value; (B) Overall survival, cut-off based on tertile expression values; (C) Event free survival, cut-off based on the median expression value; (D) Event free survival, cut-off based on tertile expression values; E) Age at diagnosis; (F) *MYCN* status; (G) INSS stage.

**Supplementary Figure S22**

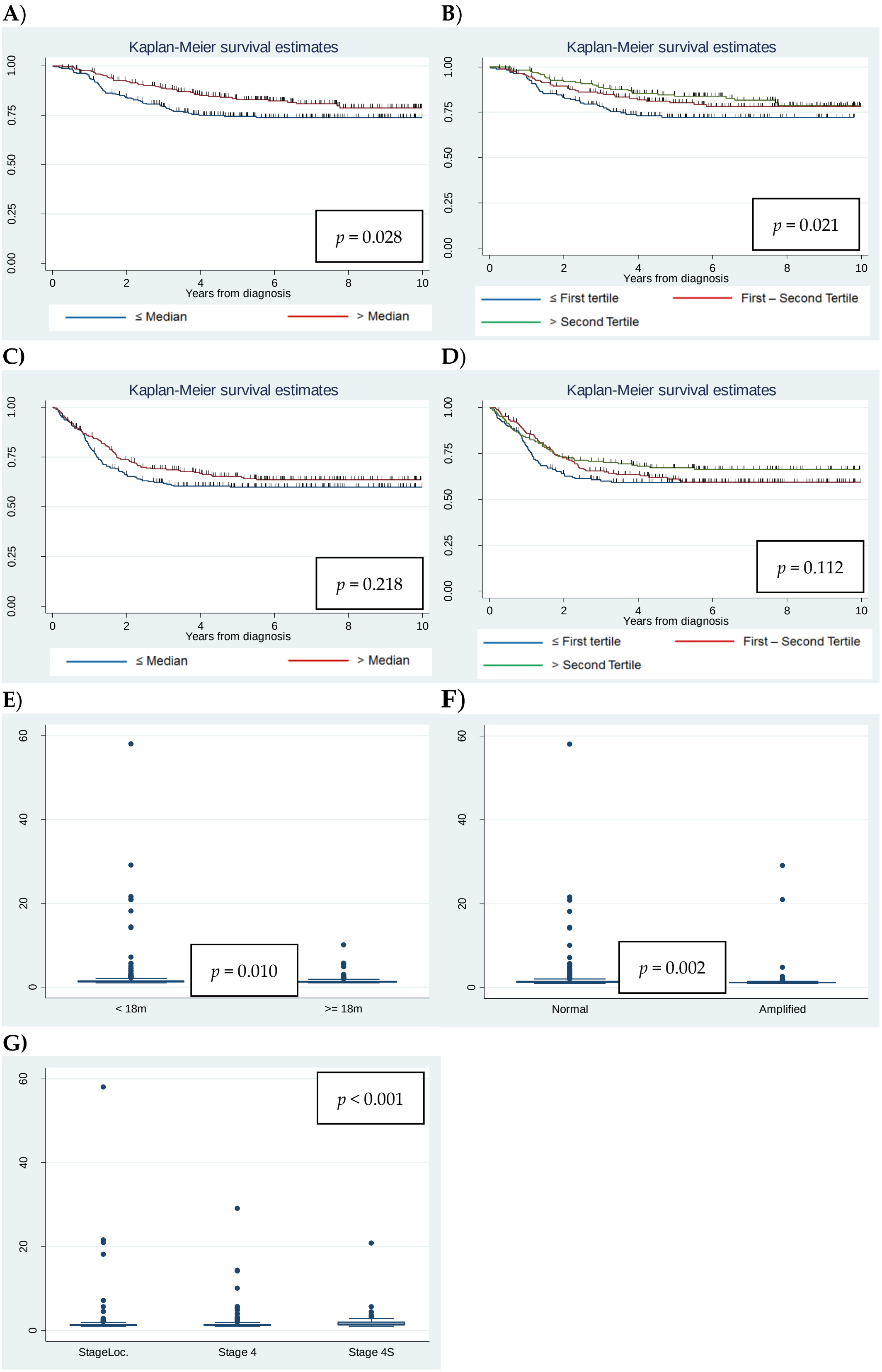


*ANKRD1* gene expression and its association with survival and clinical characteristics in a cohort of 498 NB patients in the SEQC-498 *in silico* dataset. (A) Overall survival, cut-off based on the median expression value; (B) Overall survival, cut-off based on tertile expression values; (C) Event free survival, cut-off based on the median expression value; (D) Event free survival, cut-off based on tertile expression values; E) Age at diagnosis; (F) *MYCN* status; (G) INSS stage.

**Supplementary Figure S23**

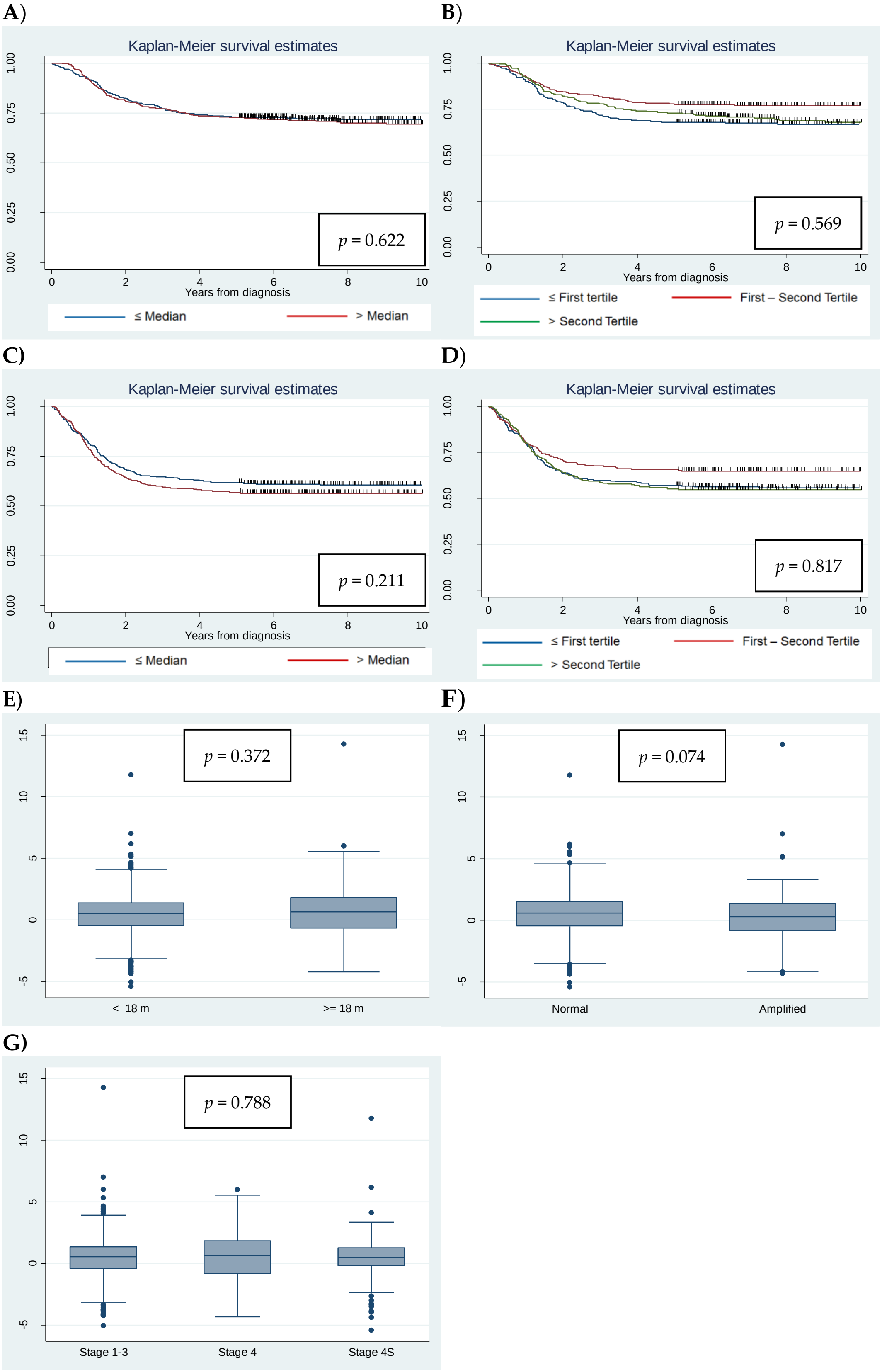


*ANRKD22* gene expression and its association with survival and clinical characteristics in a cohort of 786 NB patients in the Cangelosi et al. *in silico* dataset. (A) Overall survival, cut-off based on the median expression value; (B) Overall survival, cut-off based on tertile expression values; (C) Event free survival, cut-off based on the median expression value; (D) Event free survival, cut-off based on tertile expression values; E) Age at diagnosis; (F) *MYCN* status; (G) INSS stage.

.

**Supplementary Figure S24**

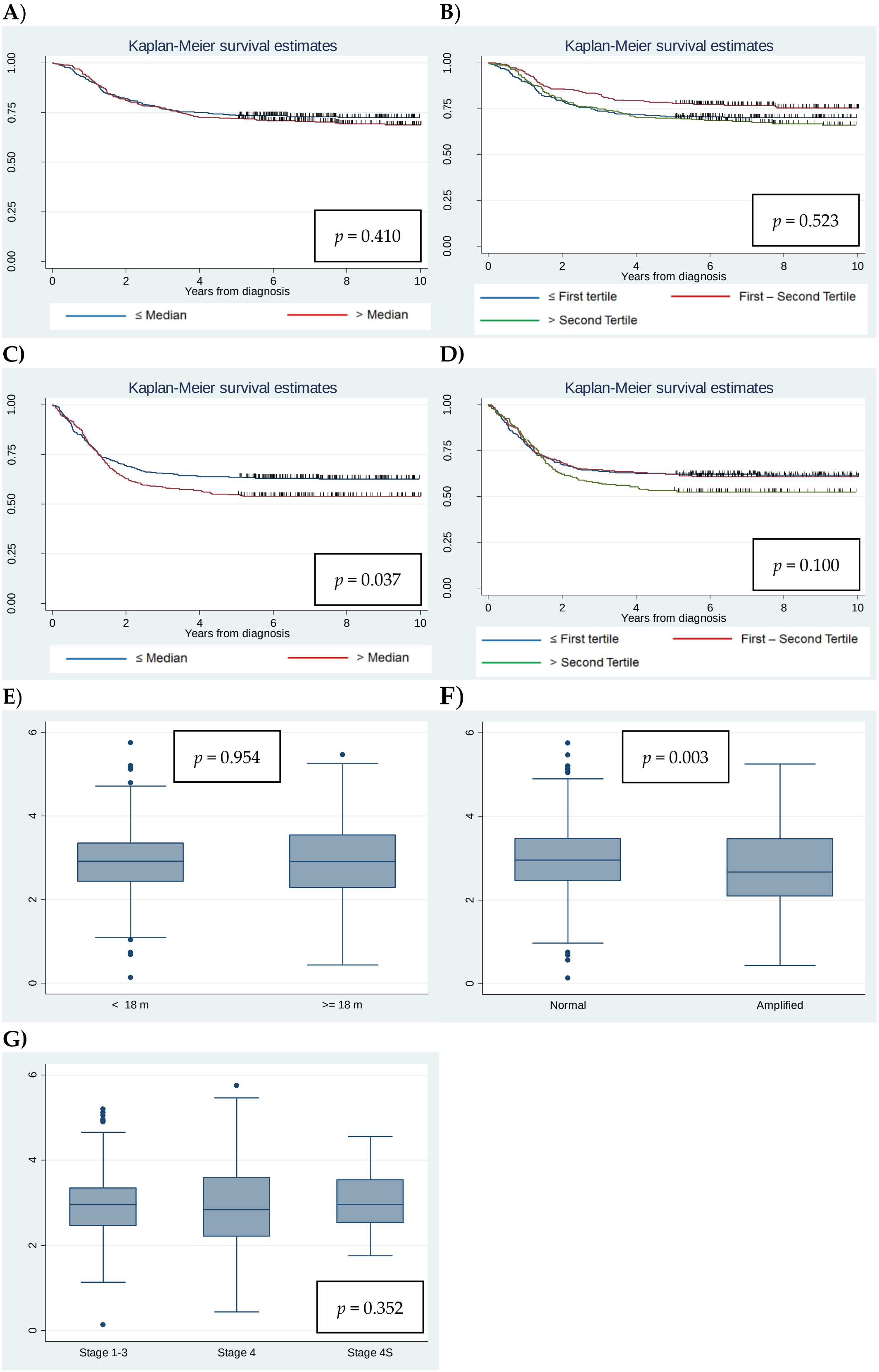


*ANXA11* gene expression and its association with survival and clinical characteristics in a cohort of 786 NB patients in the Cangelosi et al. *in silico* dataset. (A) Overall survival, cut-off based on the median expression value; (B) Overall survival, cut-off based on tertile expression values; (C) Event free survival, cut-off based on the median expression value; (D) Event free survival, cut-off based on tertile expression values; E) Age at diagnosis; (F) *MYCN* status; (G) INSS stage.

## Supplementary Figure S25

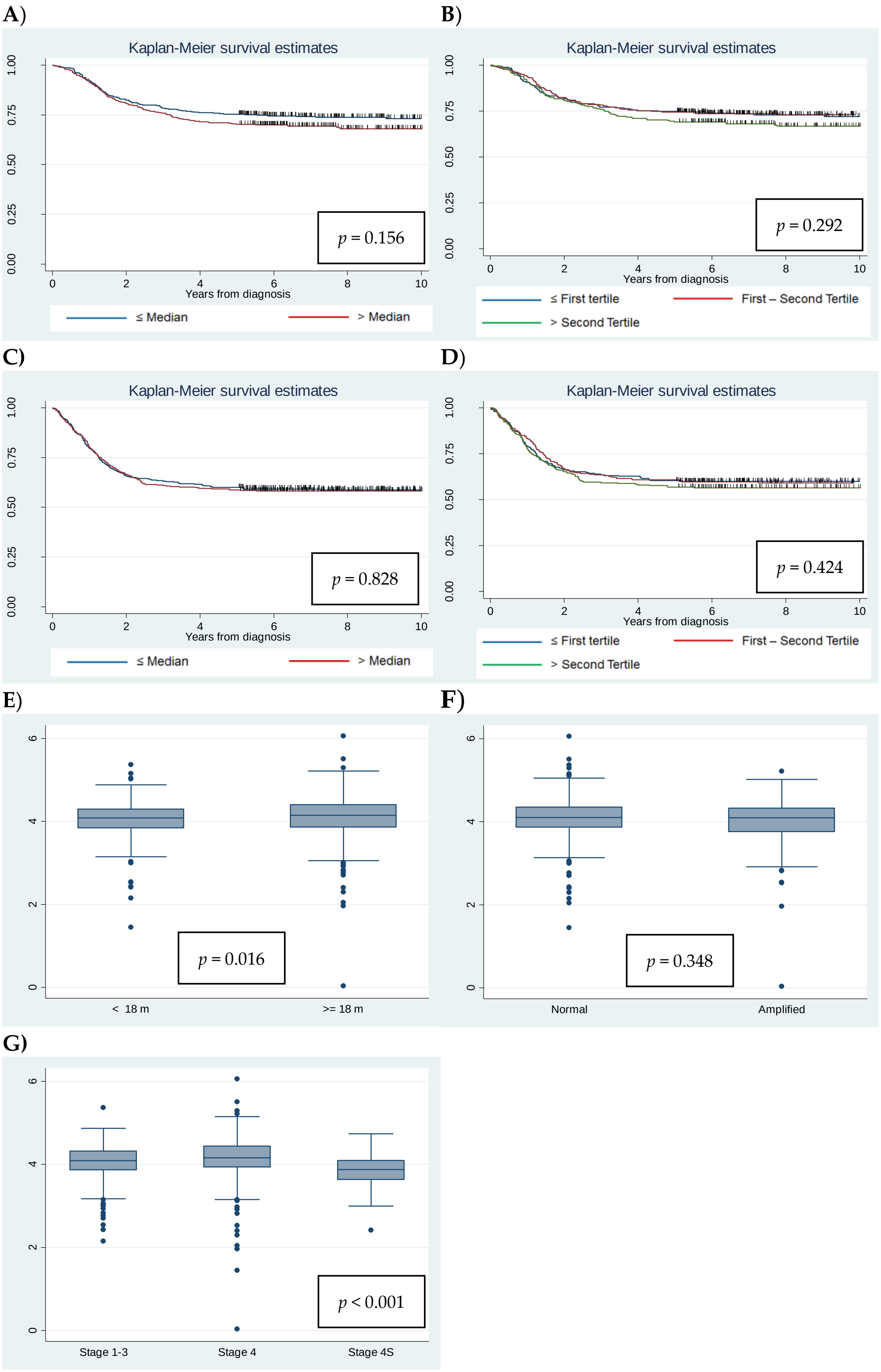


*ATAD1* gene expression and its association with survival and clinical characteristics in a cohort of 786 NB patients in the Cangelosi et al. *in silico* dataset. (A) Overall survival, cut-off based on the median expression value; (B) Overall survival, cut-off based on tertile expression values; (C) Event free survival, cut-off based on the median expression value; (D) Event free survival, cut-off based on tertile expression values; E) Age at diagnosis; (F) *MYCN* status; (G) INSS stage.

**Supplementary Figure S26**

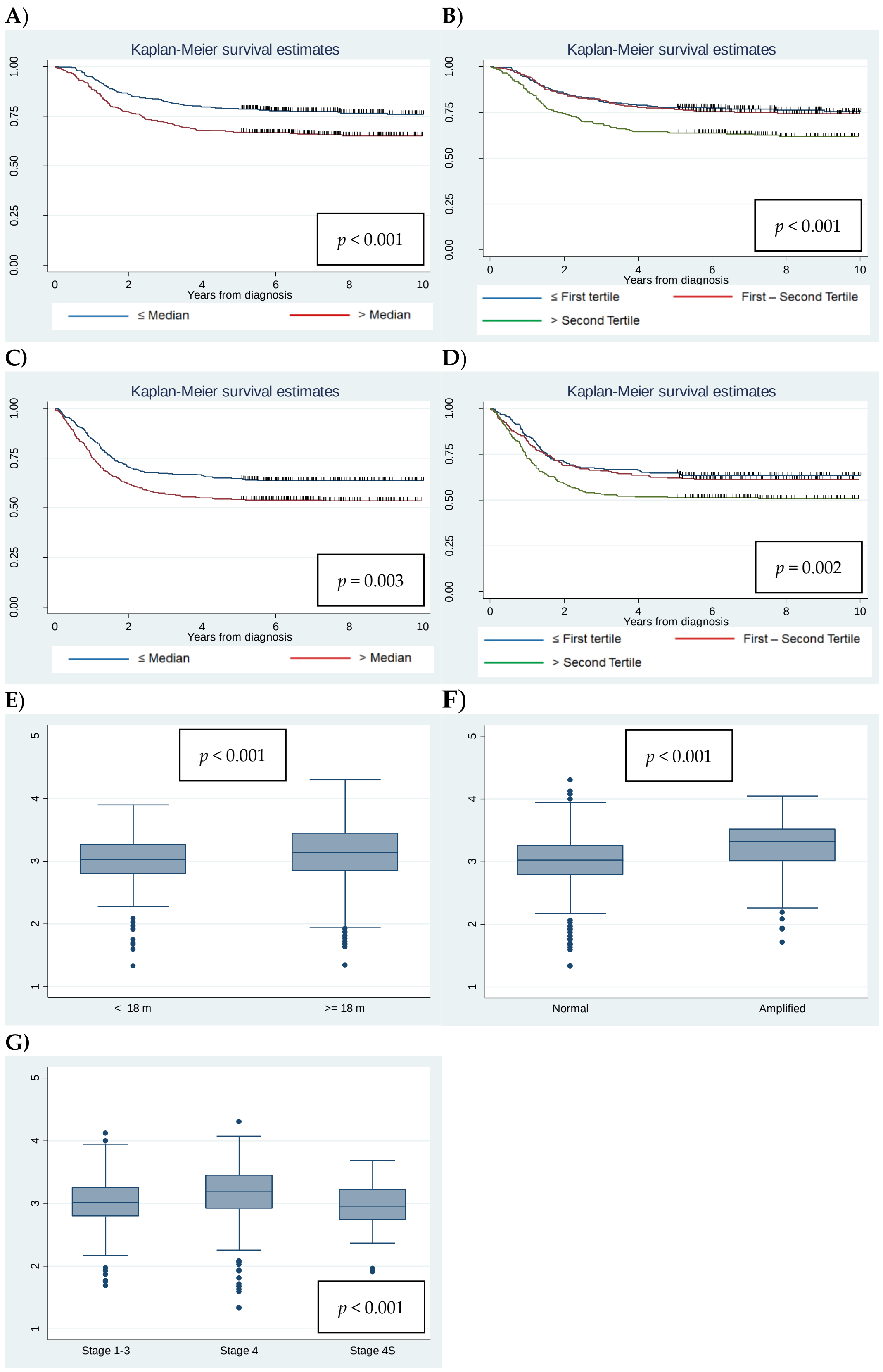


*BMPR1A* gene expression and its association with survival and clinical characteristics in a cohort of 786 NB patients in the Cangelosi et al. *in silico* dataset. (A) Overall survival, cut-off based on the median expression value; (B) Overall survival, cut-off based on tertile expression values; (C) Event free survival, cut-off based on the median expression value; (D) Event free survival, cut-off based on tertile expression values; E) Age at diagnosis; (F) *MYCN* status; (G) INSS stage.

## Supplementary Figure S27

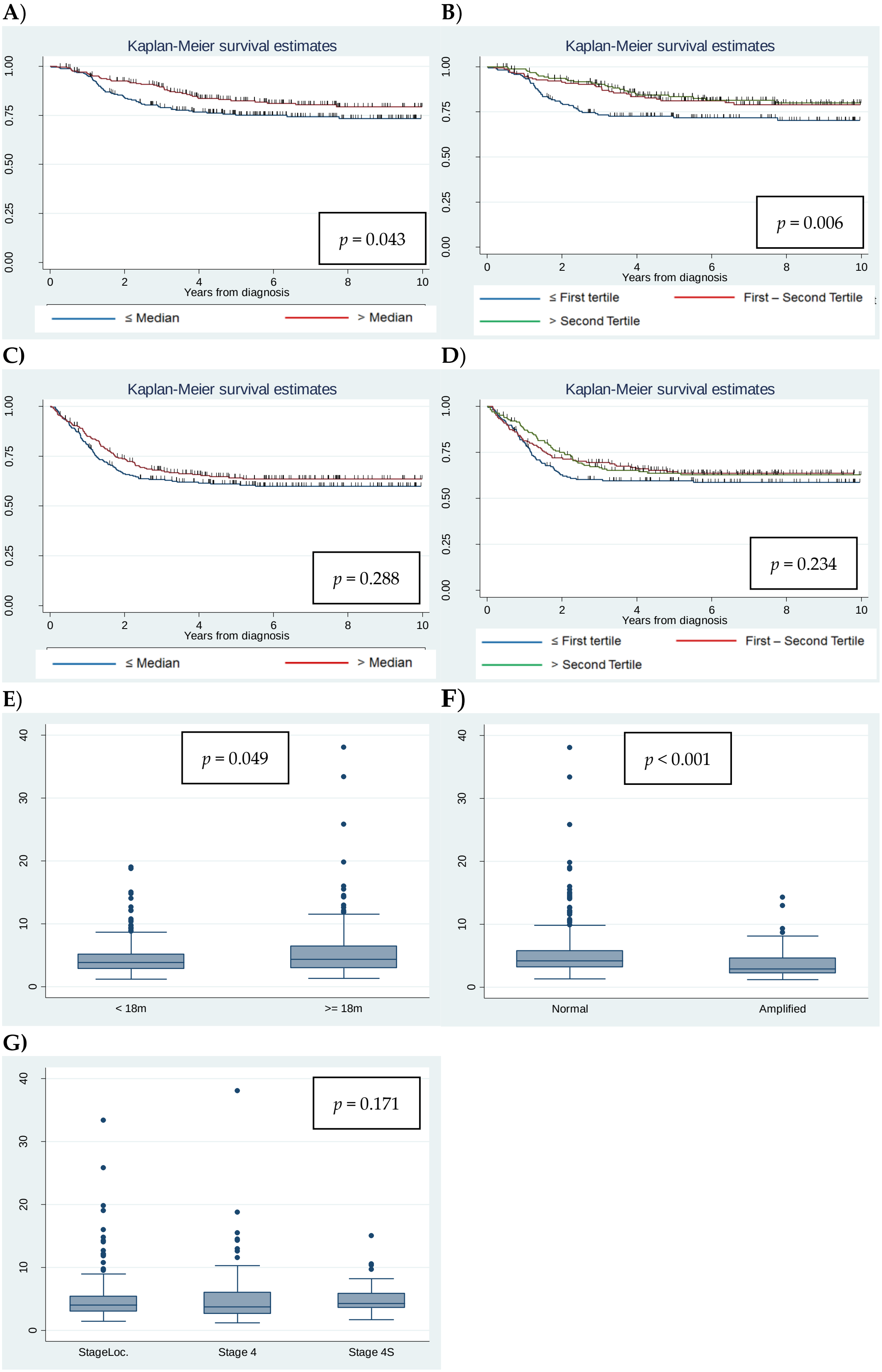


*C10orf116* gene expression and its association with survival and clinical characteristics in a cohort of 498 NB patients in the SEQC-498 *in silico* dataset. (A) Overall survival, cut-off based on the median expression value; (B) Overall survival, cut-off based on tertile expression values; (C) Event free survival, cut-off based on the median expression value; (D) Event free survival, cut-off based on tertile expression values; E) Age at diagnosis; (F) *MYCN* status; (G) INSS stage.

**Supplementary Figure S28**

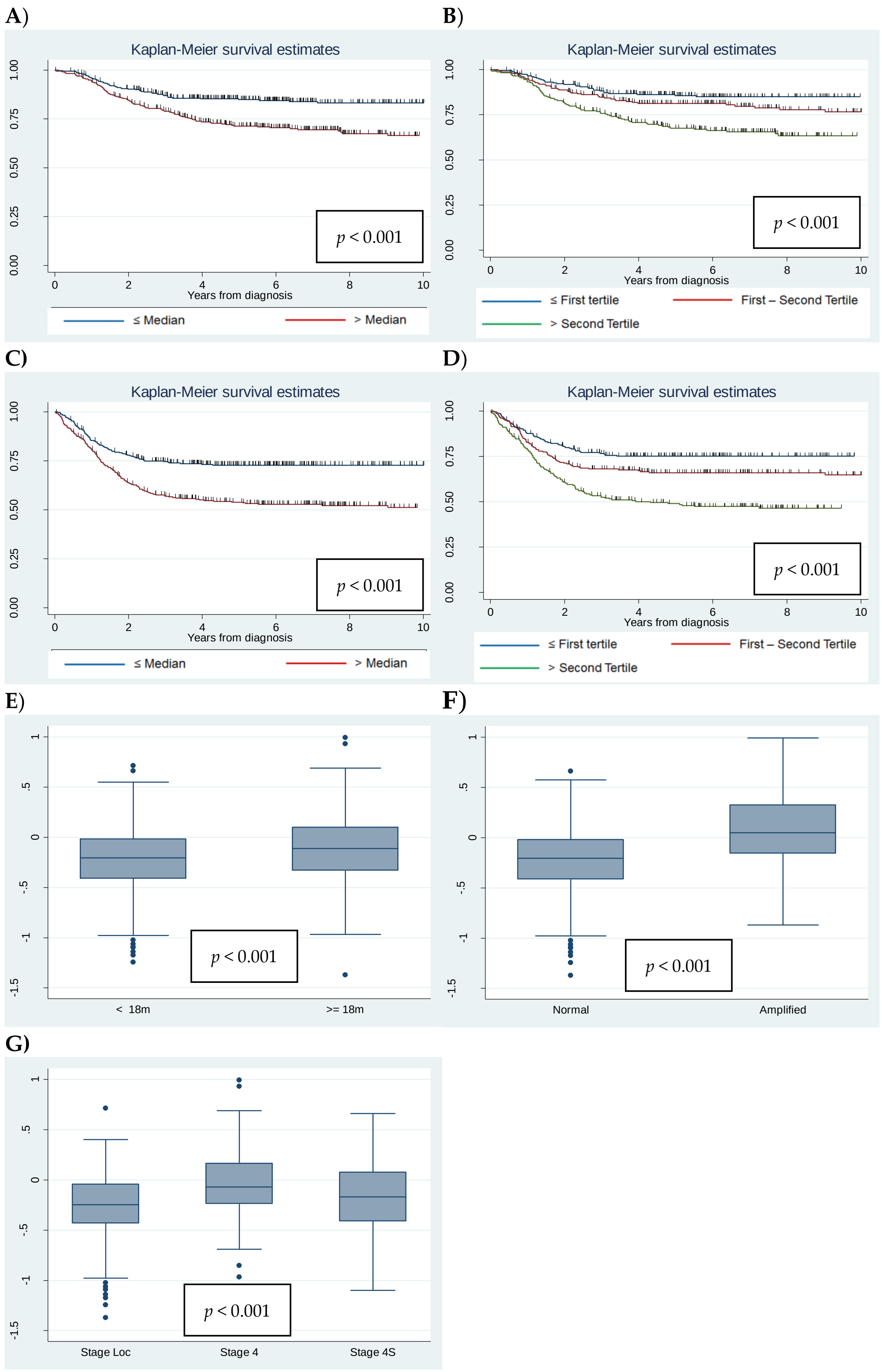


*C10orf57* (alias *FLJ3263*, Agilent probe: A_23_P97853) gene expression and its association with survival and clinical characteristics in a cohort of 709 NB patients in the E-MTAB-1781 *in silico* dataset. (A) Overall survival, cut-off based on the median expression value; (B) Overall survival, cut-off based on tertile expression values; (C) Event free survival, cut-off based on the median expression value; (D) Event free survival, cut-off based on tertile expression values; E) Age at diagnosis; (F) *MYCN* status; (G) INSS stage.

**Supplementary Figure S29**

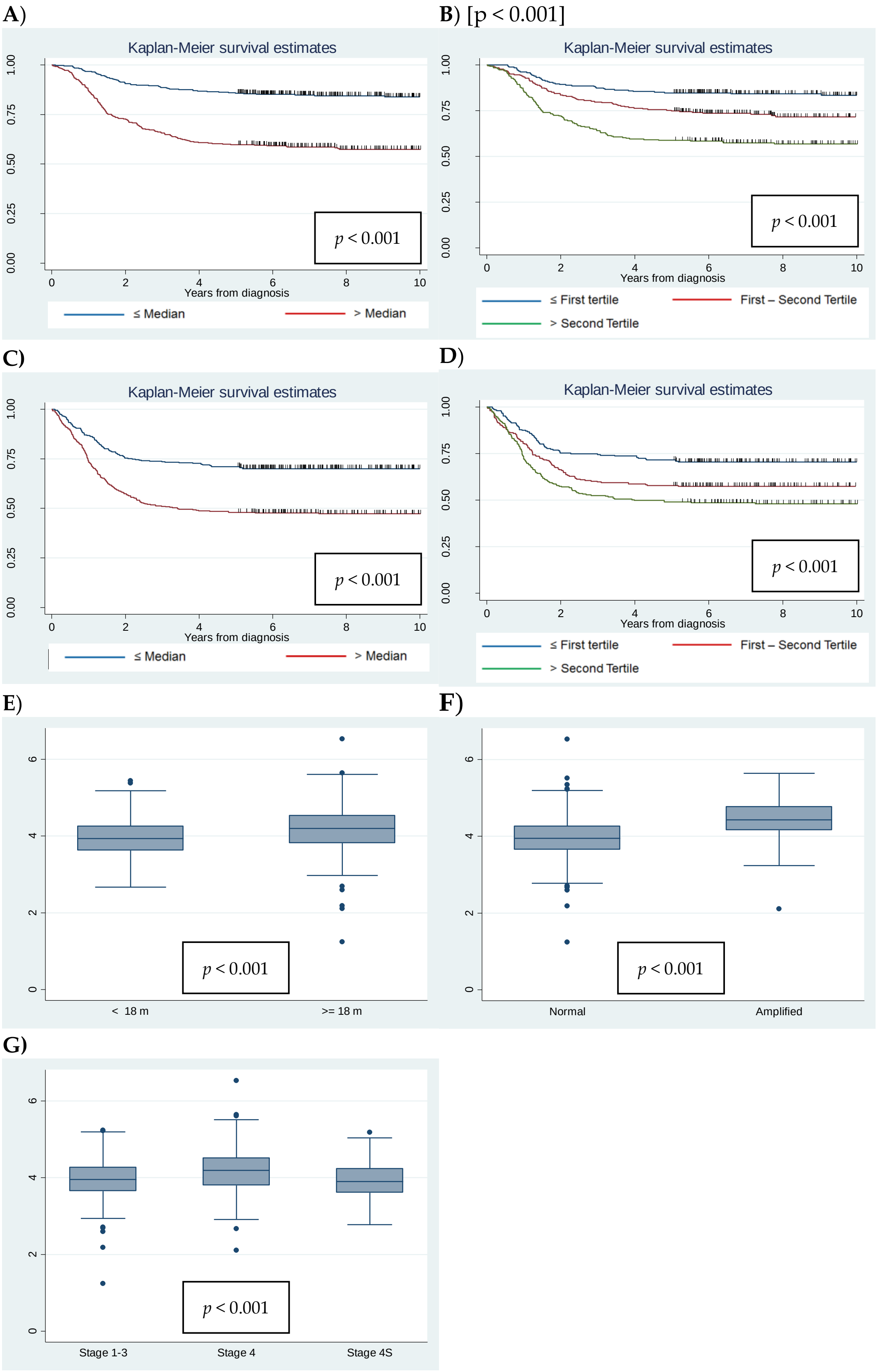


*C10prf58* (alias *FAM213A*) gene expression and its association with survival and clinical characteristics in a cohort of 786 NB patients in the Cangelosi et al. *in silico* dataset. (A) Overall survival, cut-off based on the median expression value; (B) Overall survival, cut-off based on tertile expression values; (C) Event free survival, cut-off based on the median expression value; (D) Event free survival, cut-off based on tertile expression values; E) Age at diagnosis; (F) *MYCN* status; (G) INSS stage.

**Supplementary Figure S30**

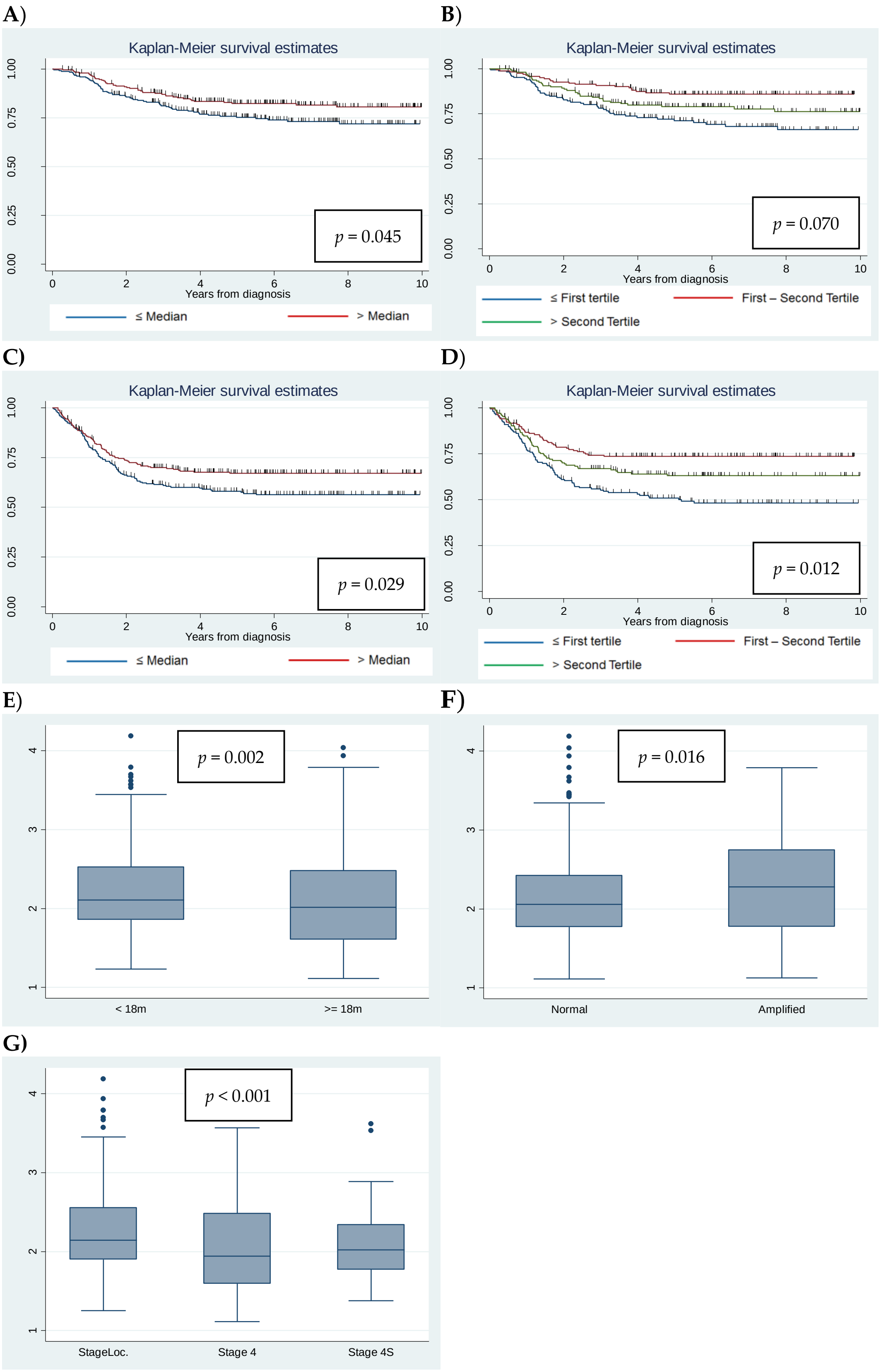


*CFL1P1* gene expression and its association with survival and clinical characteristics in a cohort of 498 NB patients in the SEQC-498. *in silico* dataset. (A) Overall survival, cut-off based on the median expression value; (B) Overall survival, cut-off based on tertile expression values; (C) Event free survival, cut-off based on the median expression value; (D) Event free survival, cut-off based on tertile expression values; E) Age at diagnosis; (F) *MYCN* status; (G) INSS stage.

## Supplementary Figure S31

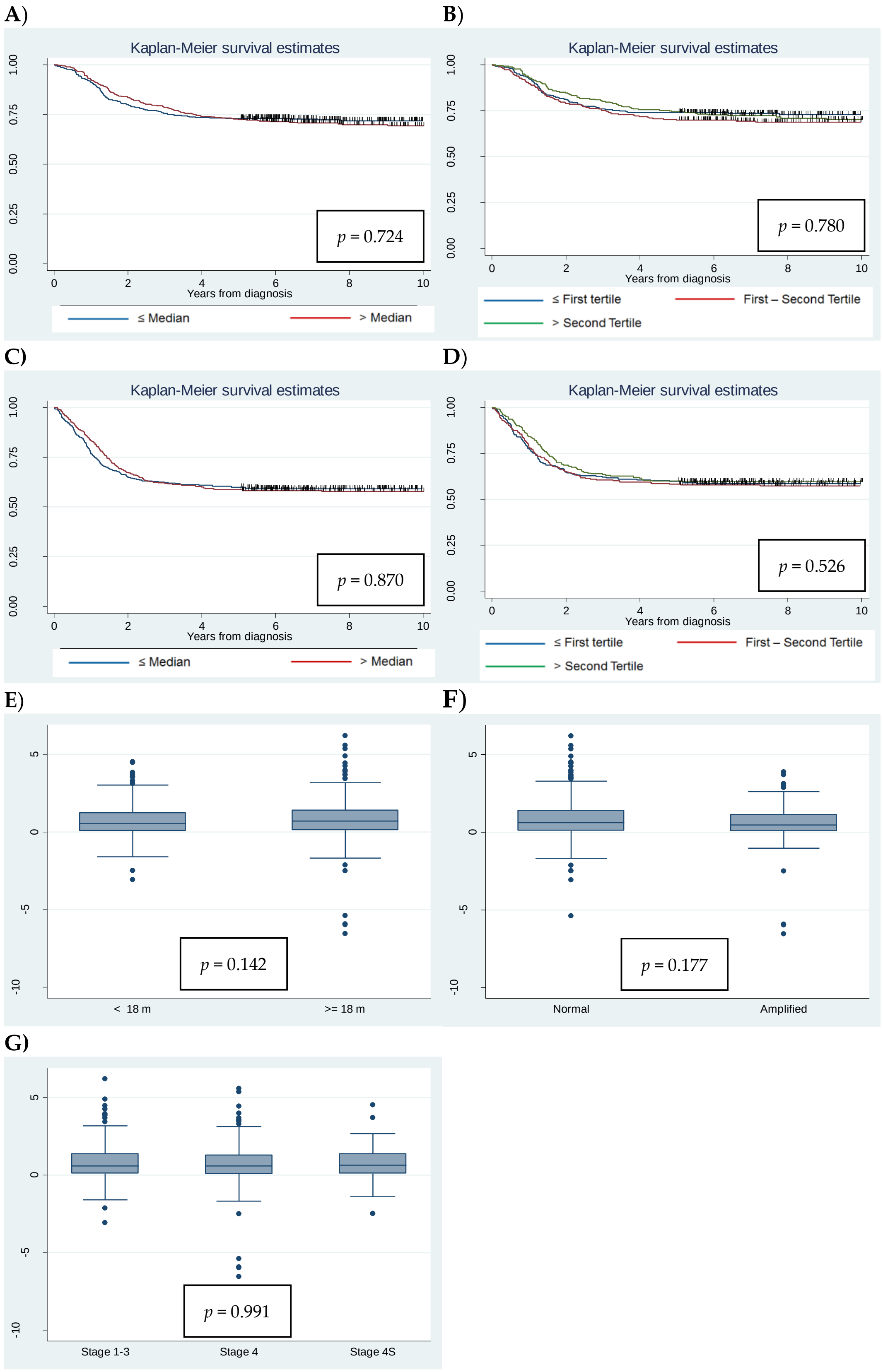


*CH25H* gene expression and its association with survival and clinical characteristics in a cohort of 786 NB patients in the Cangelosi et al. *in silico* dataset. (A) Overall survival, cut-off based on the median expression value; (B) Overall survival, cut-off based on tertile expression values; (C) Event free survival, cut-off based on the median expression value; (D) Event free survival, cut-off based on tertile expression values; E) Age at diagnosis; (F) *MYCN* status; (G) INSS stage.

**Supplementary Figure S32**

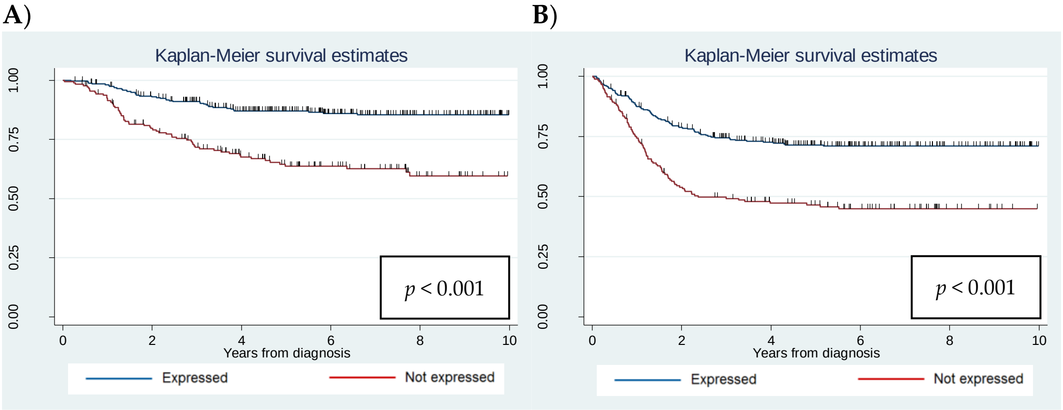


*DYDC1* gene expression and its association with survival and clinical characteristics in a cohort of 498 NB patients in the SEQC-498 *in silico* dataset. (A) Overall survival, gene expression yes/not; (B) Event Free Survival, gene expression yes/not.

## Supplementary Figure S33

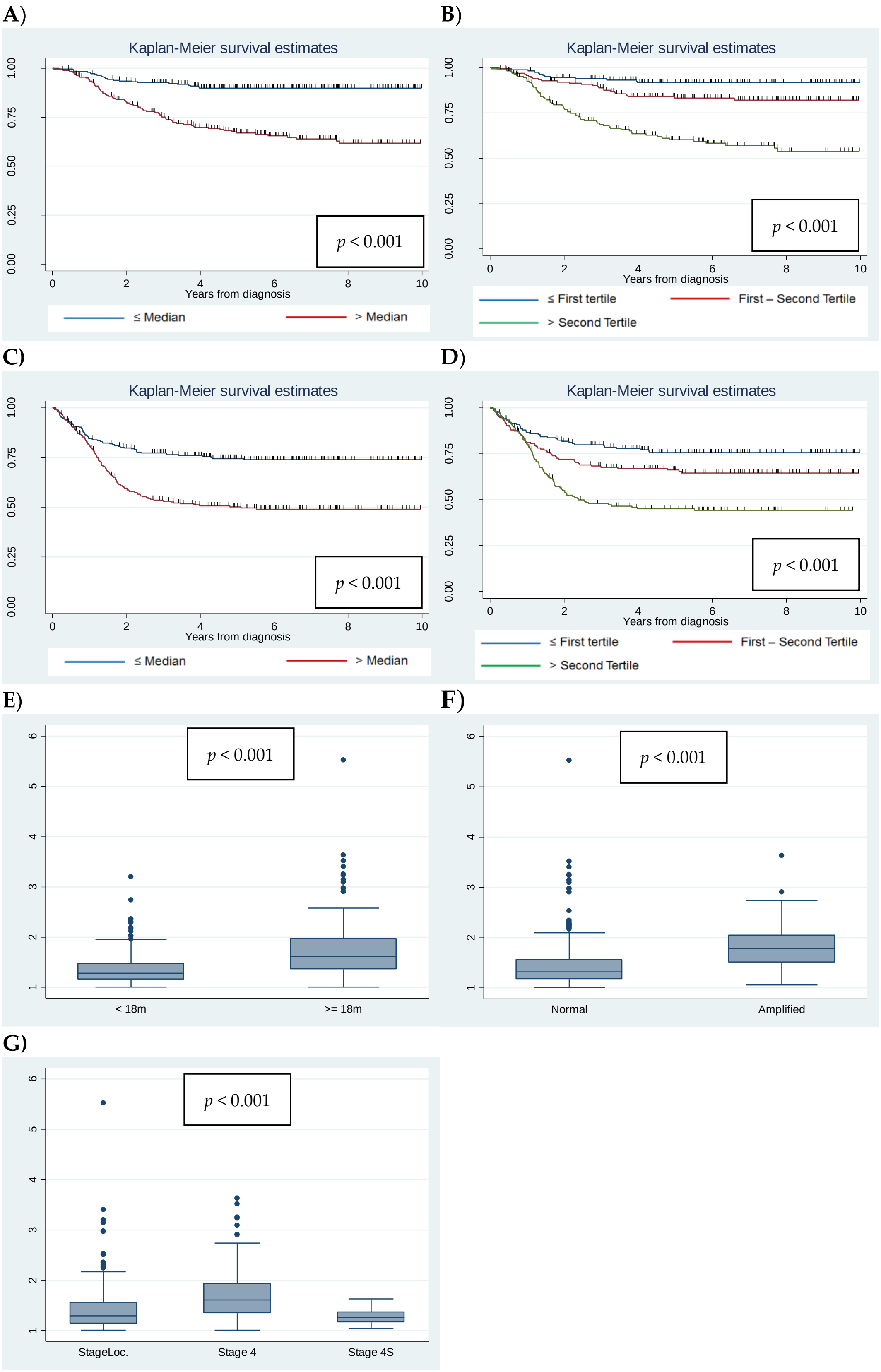


*DYDC2* gene expression and its association with  survival and clinical characteristics in a cohort of 498 NB patients in the SEQC-498. *in silico* dataset. (A) Overall survival, cut-off based on the median expression value; (B) Overall survival, cut-off based on tertile expression values; (C) Event free survival, cut-off based on the median expression value; (D) Event free survival, cut-off based on tertile expression values; E) Age at diagnosis; (F) *MYCN* status; (G) INSS  stage.

**Supplementary Figure S34**

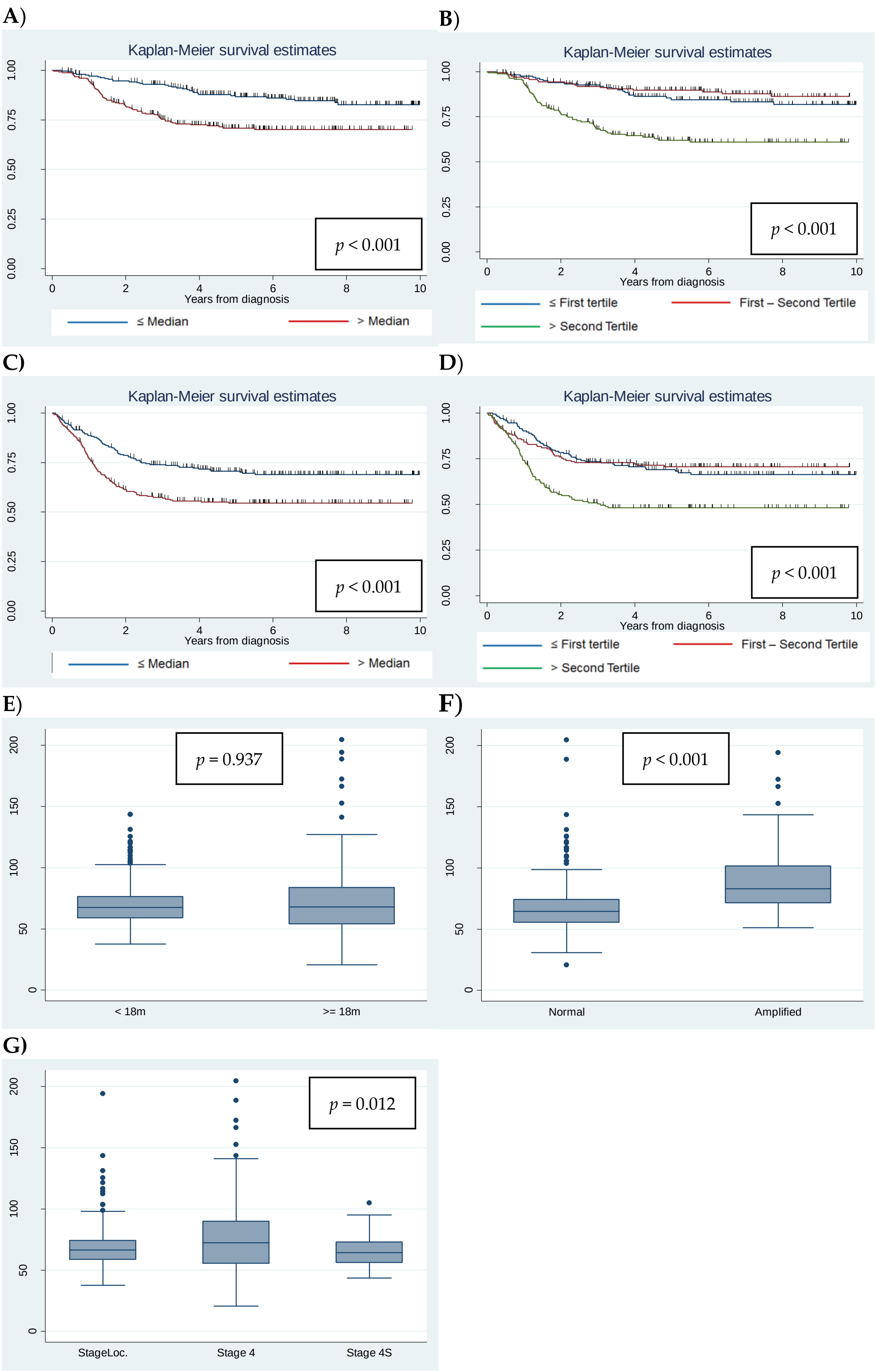


*EIF5AL1* gene expression and its association with survival and clinical characteristics in a cohort of 498 NB patients in the SEQC-498 *in silico* dataset. (A) Overall survival, cut-off based on the median expression value; (B) Overall survival, cut-off based on tertile expression values; (C) Event free survival, cut-off based on the median expression value; (D) Event free survival, cut-off based on tertile expression values; E) Age at diagnosis; (F) *MYCN* status; (G) INSS stage.

**Supplementary Figure S35**

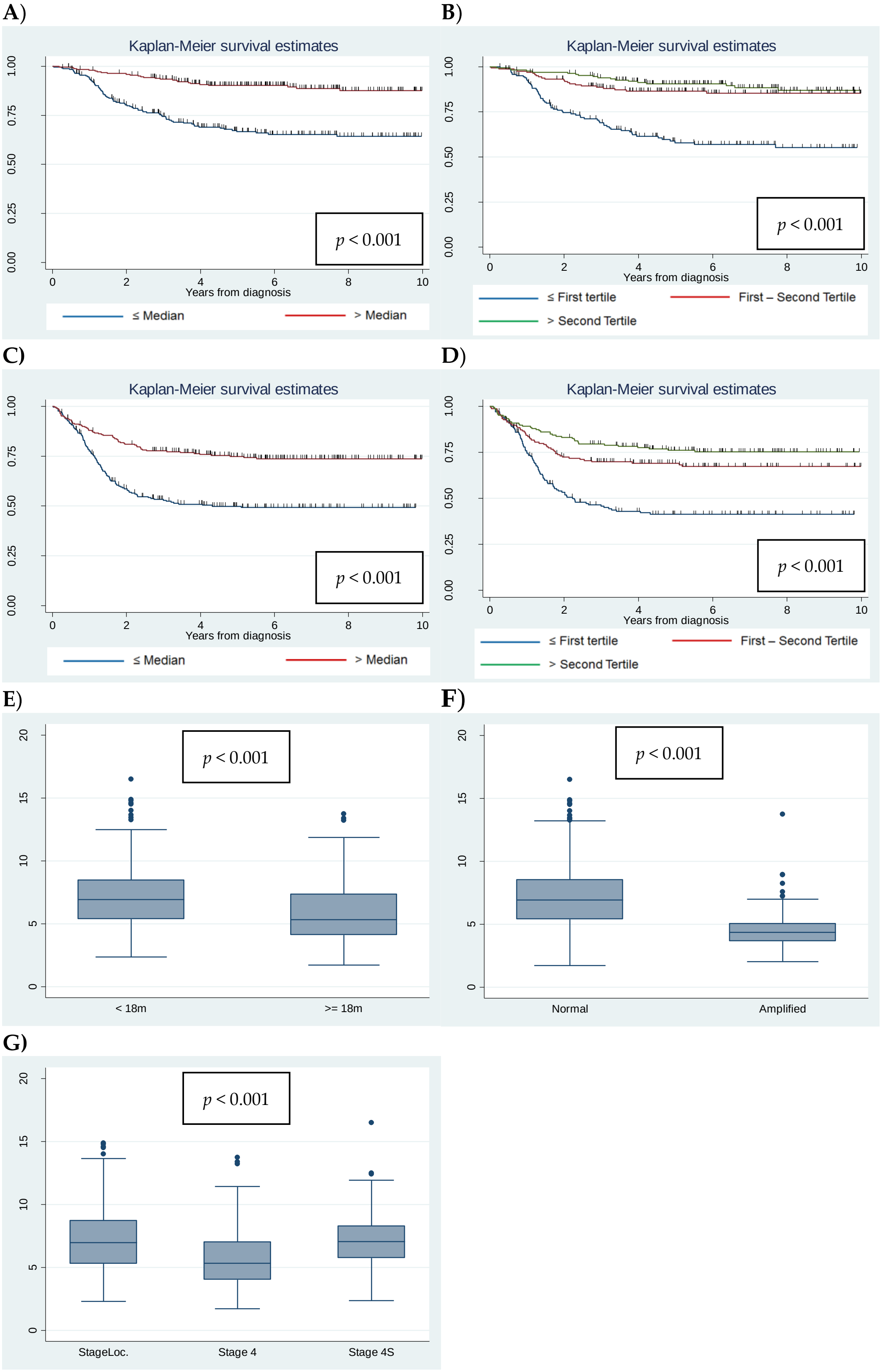


*FAM22A* gene expression and its association with survival and clinical characteristics in a cohort of 498 NB patients in the SEQC-498. *in silico* dataset. (A) Overall survival, cut-off based on the median expression value; (B) Overall survival, cut-off based on tertile expression values; (C) Event free survival, cut-off based on the median expression value; (D) Event free survival, cut-off based on tertile expression values; E) Age at diagnosis; (F) *MYCN* status; (G) INSS stage.

## Supplementary Figure S36

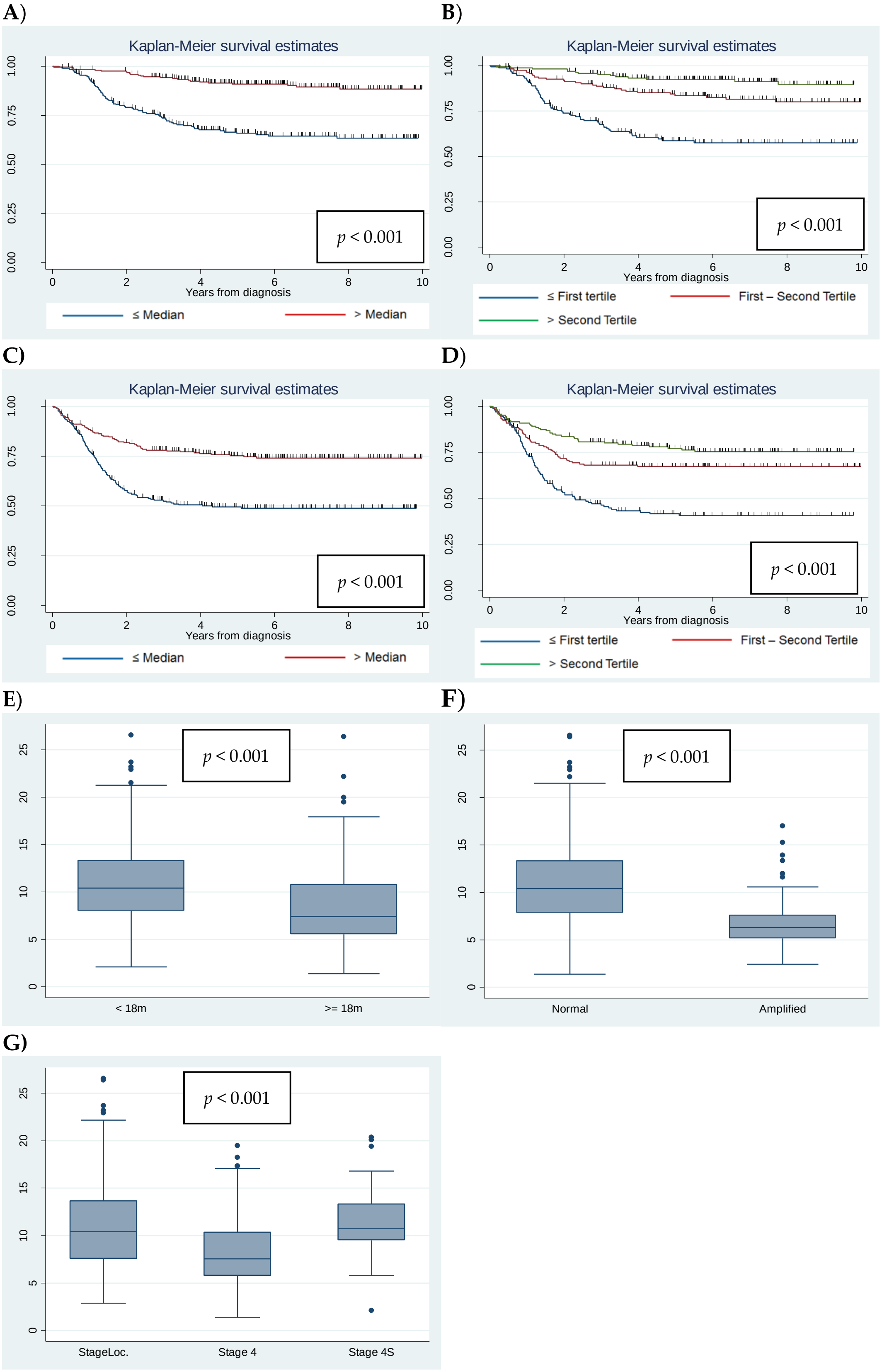


*FAM22D* gene expression and its association with survival and clinical characteristics in a cohort of 498 NB patients in the SEQC-498. *in silico* dataset. (A) Overall survival, cut-off based on the median expression value; (B) Overall survival, cut-off based on tertile expression values; (C) Event free survival, cut-off based on the median expression value; (D) Event free survival, cut-off based on tertile expression values; E) Age at diagnosis; (F) *MYCN* status; (G) INSS stage.

## Supplementary Figure S37

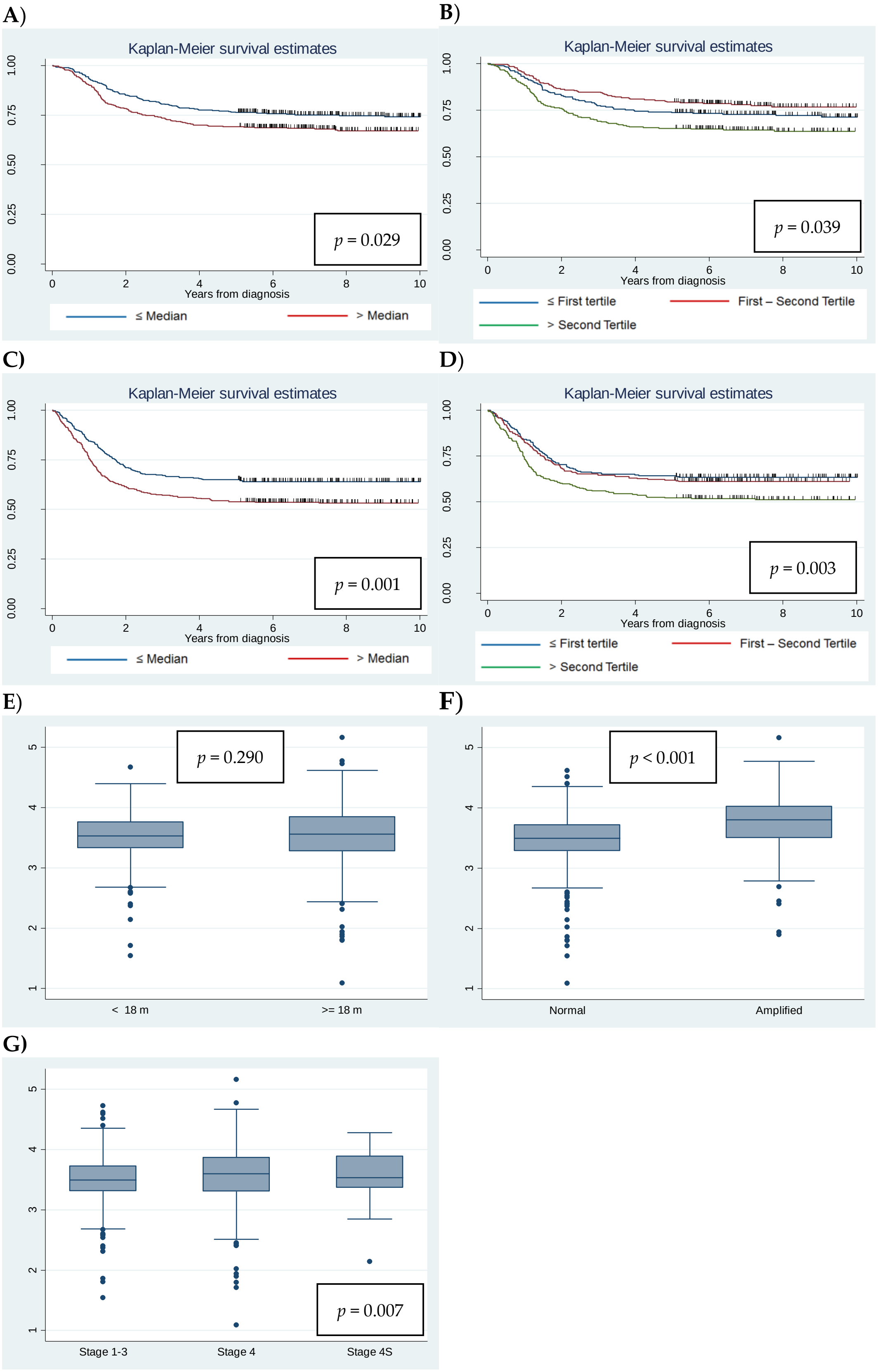


*FAM35A* gene expression and its association with survival and clinical characteristics in a cohort of 786 NB patients in the Cangelosi et al. *in silico* dataset. (A) Overall survival, cut-off based on the median expression value; (B) Overall survival, cut-off based on tertile expression values; (C) Event free survival, cut-off based on the median expression value; (D) Event free survival, cut-off based on tertile expression values; E) Age at diagnosis; (F) *MYCN* status; (G) INSS stage.

**Supplementary Figure S38**

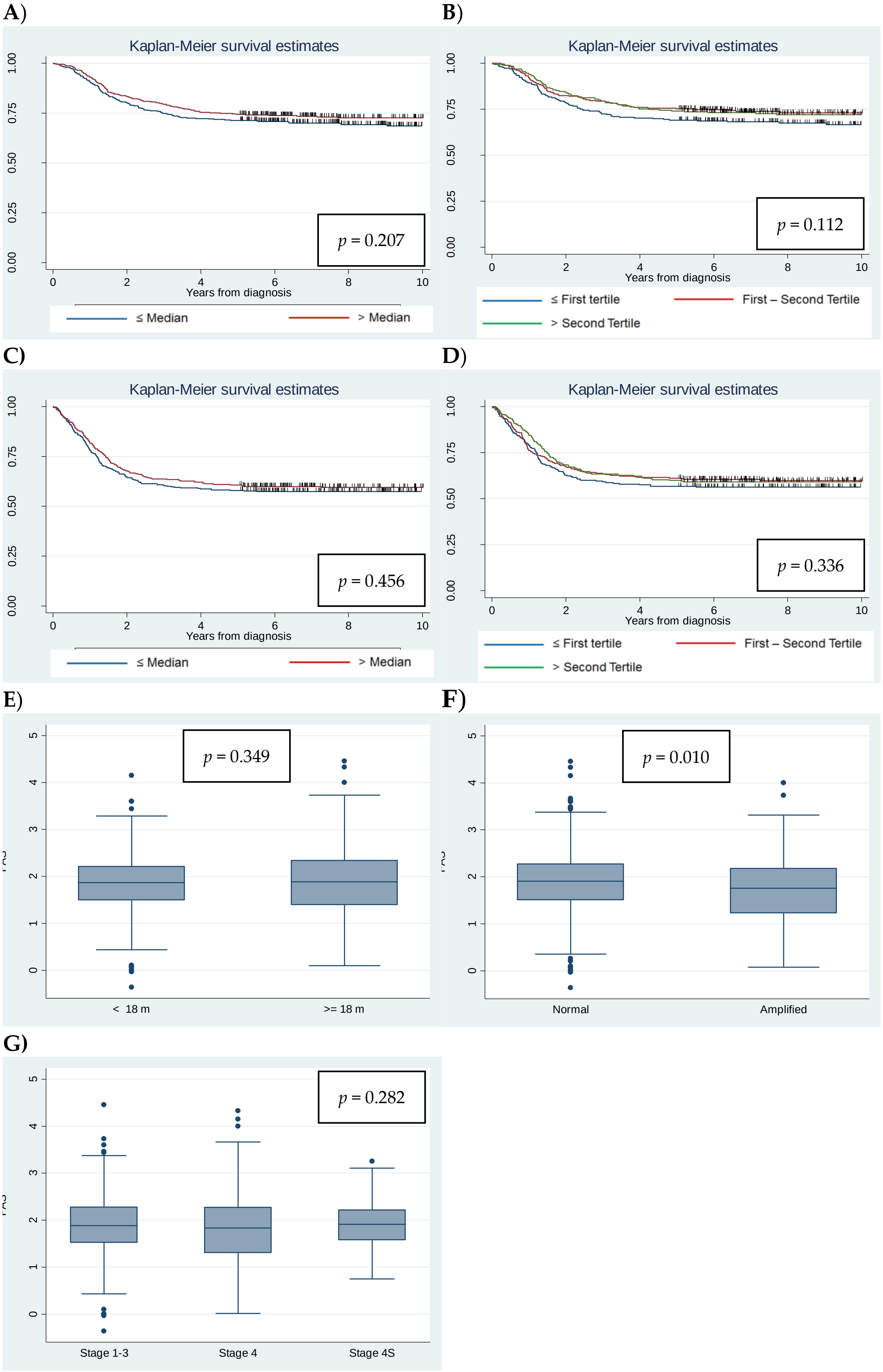


*FAS* gene expression and its association with survival and clinical characteristics in a cohort of 786 NB patients in the Cangelosi et al. *in silico* dataset. (A) Overall survival, cut-off based on the median expression value; (B) Overall survival, cut-off based on tertile expression values; (C) Event free survival, cut-off based on the median expression value; (D) Event free survival, cut-off based on tertile expression values; E) Age at diagnosis; (F) *MYCN* status; (G) INSS stage.

**Supplementary Figure S39**

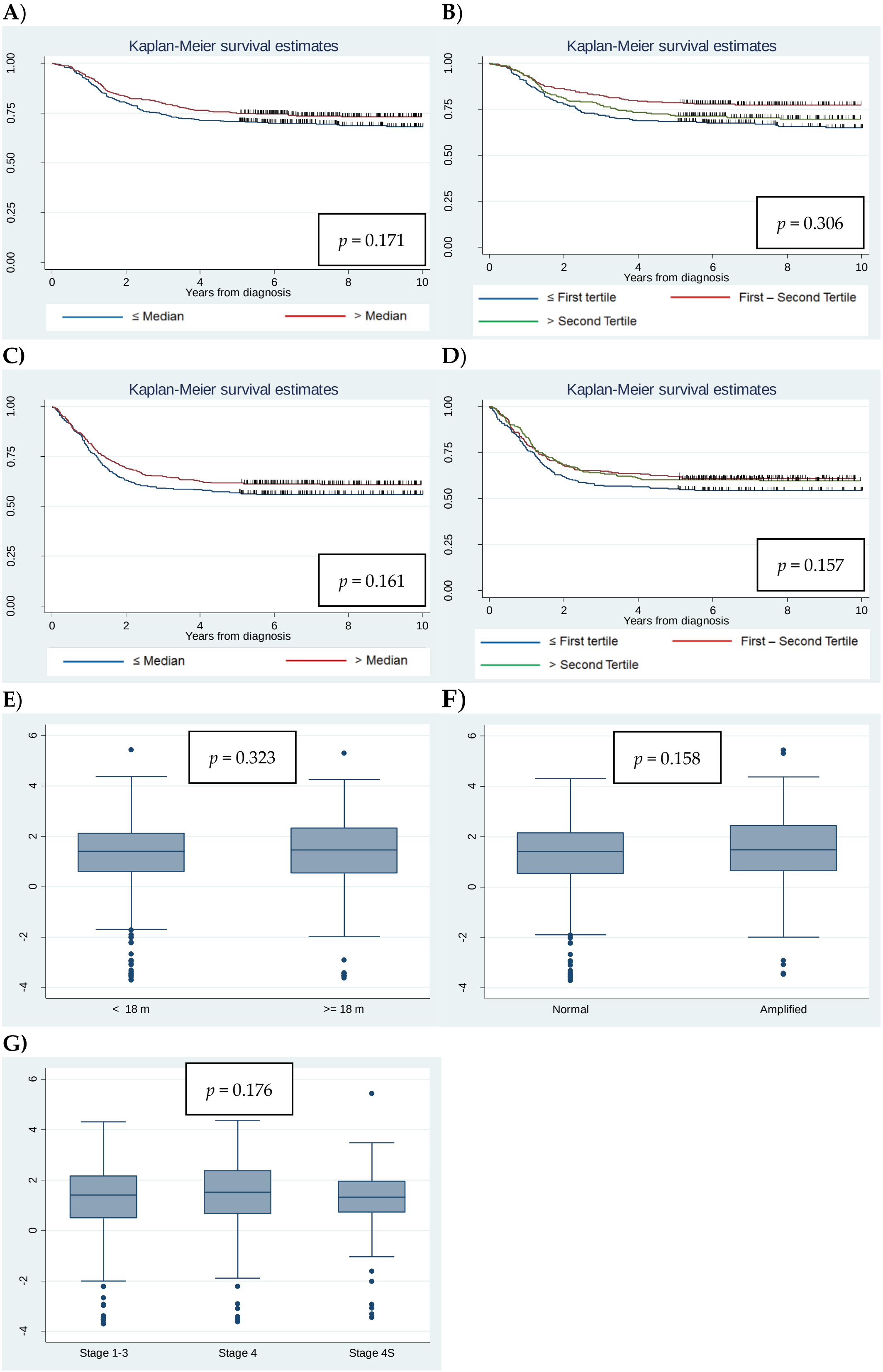


*FLJ37201* gene expression and its association with survival and clinical characteristics in a cohort of 786 NB patients in the Cangelosi et al. *in silico* dataset. (A) Overall survival, cut-off based on the median expression value; (B) Overall survival, cut-off based on tertile expression values; (C) Event free survival, cut-off based on the median expression value; (D) Event free survival, cut-off based on tertile expression values; E) Age at diagnosis; (F) *MYCN* status; (G) INSS stage.

**Supplementary Figure S40**

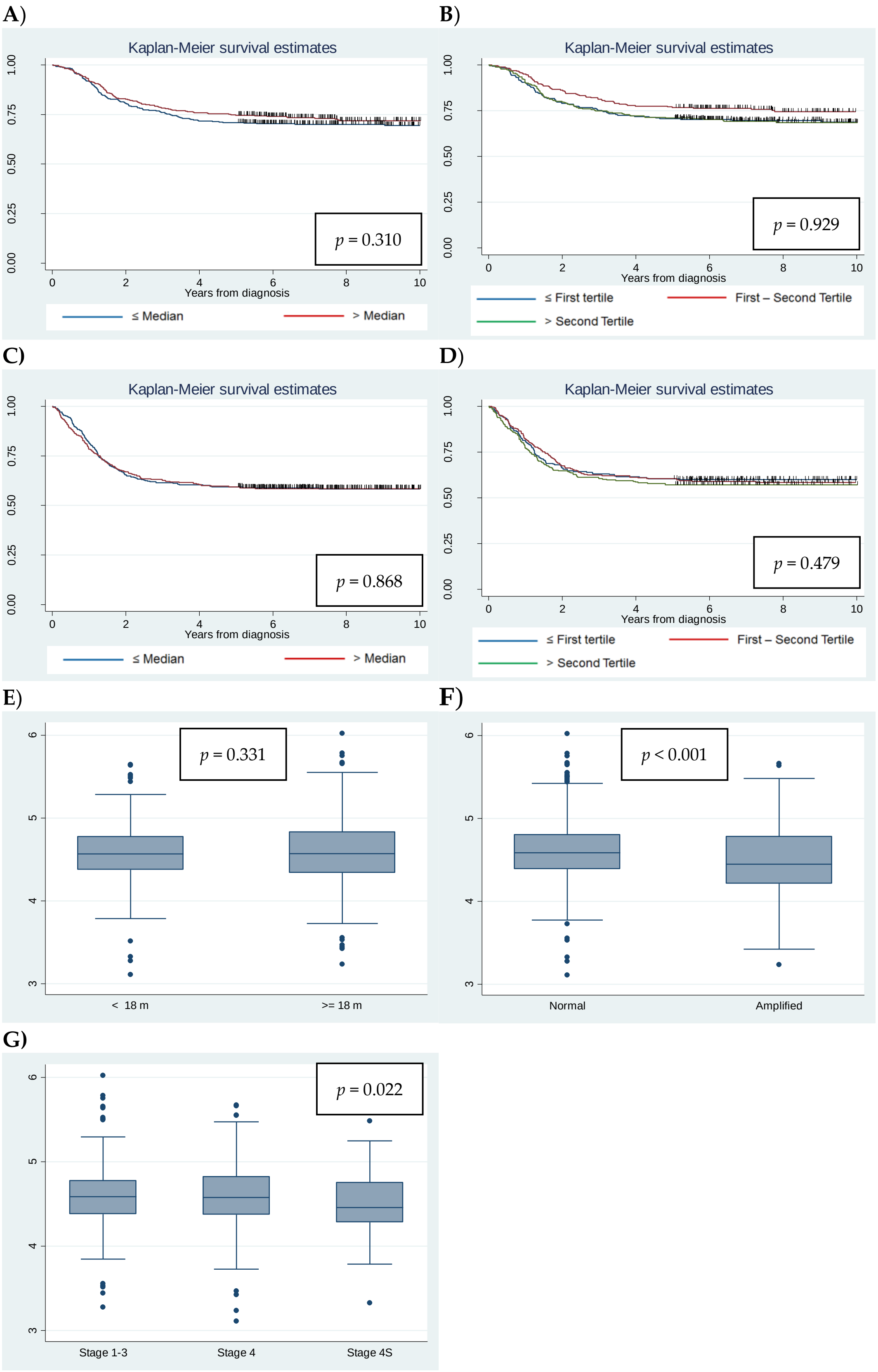


*GHITM* gene expression and its association with survival and clinical characteristics in a cohort of 786 NB patients in the Cangelosi et al. *in silico* dataset. (A) Overall survival, cut-off based on the median expression value; (B) Overall survival, cut-off based on tertile expression values; (C) Event free survival, cut-off based on the median expression value; (D) Event free survival, cut-off based on tertile expression values; E) Age at diagnosis; (F) *MYCN* status; (G) INSS stage.

**Supplementary Figure S41**

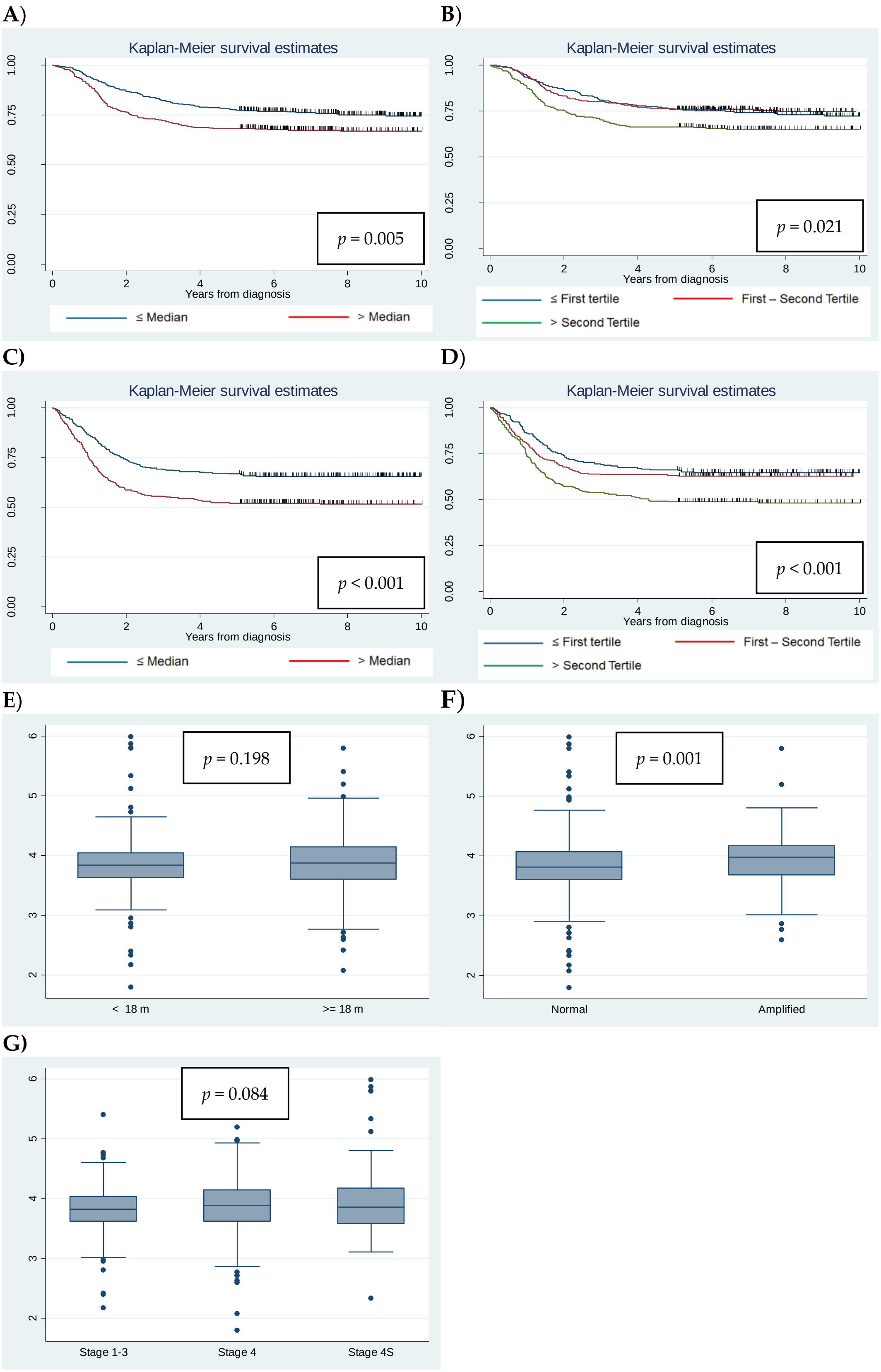


*GLUD1* gene expression and its association with survival and clinical characteristics in a cohort of 786 NB patients in the Cangelosi et al. *in silico* dataset. (A) Overall survival, cut-off based on the median expression value; (B) Overall survival, cut-off based on tertile expression values; (C) Event free survival, cut-off based on the median expression value; (D) Event free survival, cut-off based on tertile expression values; E) Age at diagnosis; (F) *MYCN* status; (G) INSS stage.

## Supplementary Figure S42

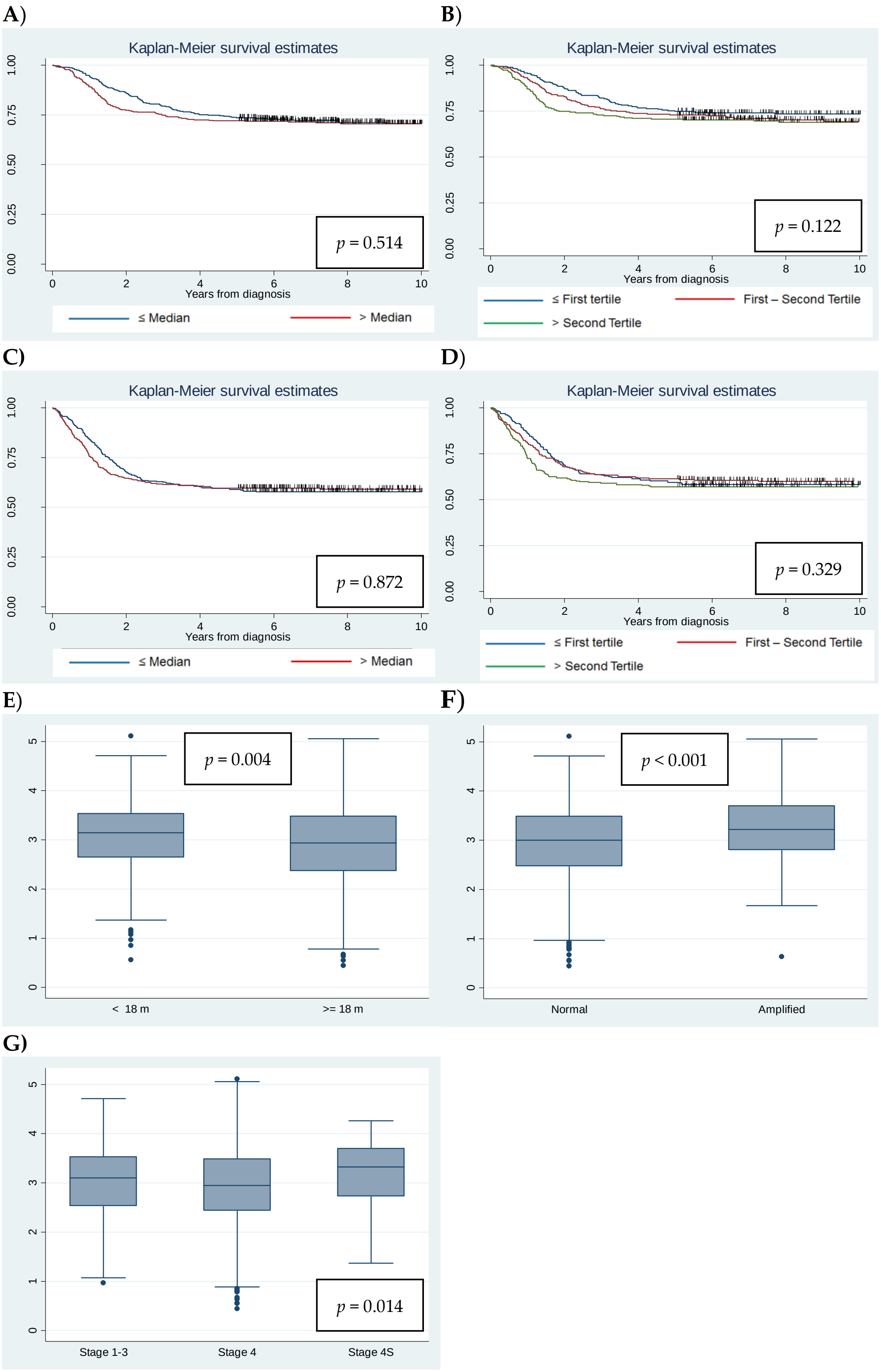


*GRID1* gene expression and its association with survival and clinical characteristics in a cohort of 786 NB patients in the Cangelosi et al. *in silico* dataset. (A) Overall survival, cut-off based on the median expression value; (B) Overall survival, cut-off based on tertile expression values; (C) Event free survival, cut-off based on the median expression value; (D) Event free survival, cut-off based on tertile expression values; E) Age at diagnosis; (F) *MYCN* status; (G) INSS stage.

## Supplementary Figure S43

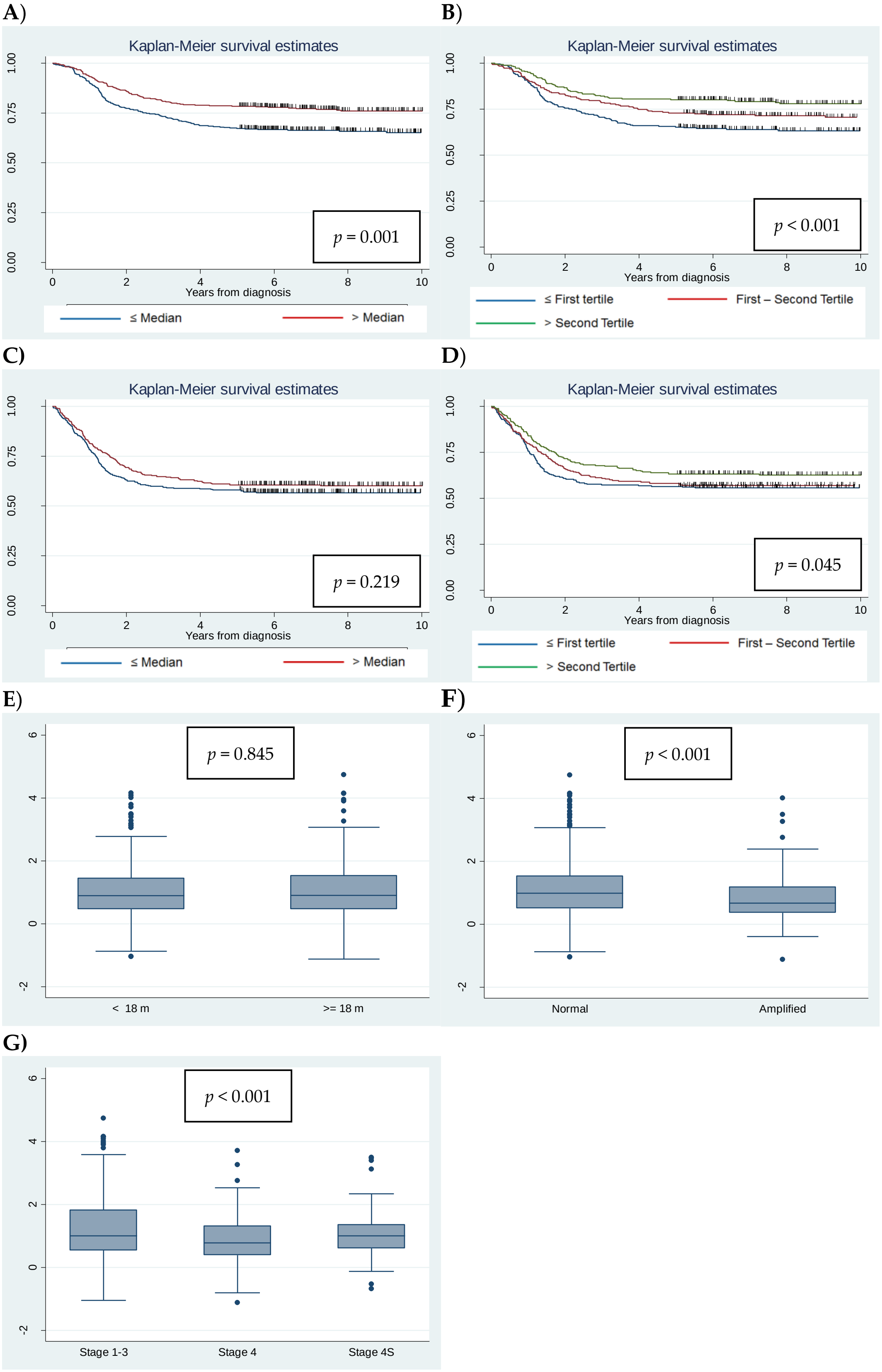


*HTR7* gene expression and its association with survival and clinical characteristics in a cohort of 786 NB patients in the Cangelosi et al. *in silico* dataset. (A) Overall survival, cut-off based on the median expression value; (B) Overall survival, cut-off based on tertile expression values; (C) Event free survival, cut-off based on the median expression value; (D) Event free survival, cut-off based on tertile expression values; E) Age at diagnosis; (F) *MYCN* status; (G) INSS stage.

**Supplementary Figure S44**

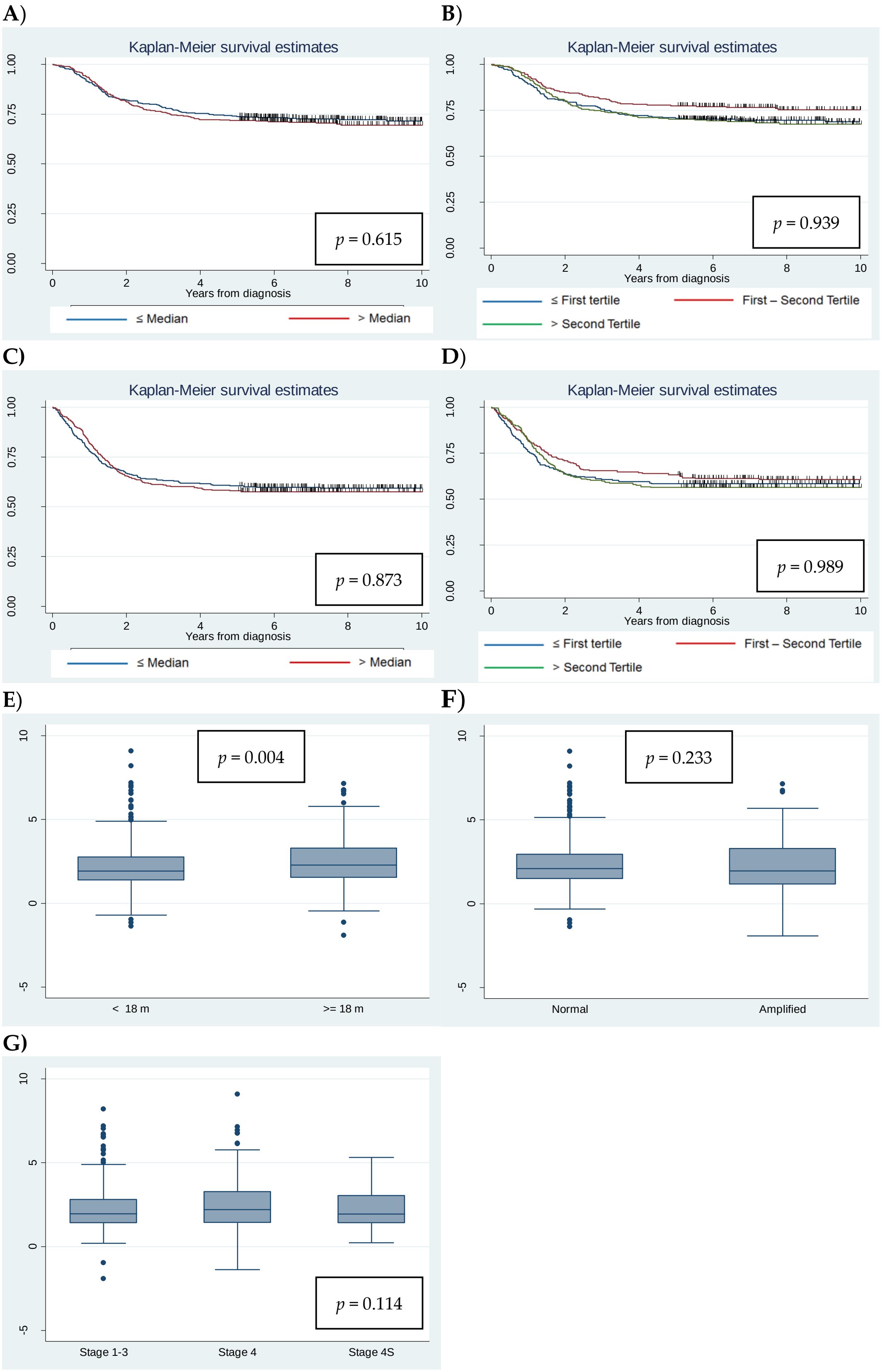


*IFIT1* gene expression and its association with survival and clinical characteristics in a cohort of 786 NB patients in the Cangelosi et al. *in silico* dataset. (A) Overall survival, cut-off based on the median expression value; (B) Overall survival, cut-off based on tertile expression values; (C) Event free survival, cut-off based on the median expression value; (D) Event free survival, cut-off based on tertile expression values; E) Age at diagnosis; (F) *MYCN* status; (G) INSS stage.

**Supplementary Figure S45**

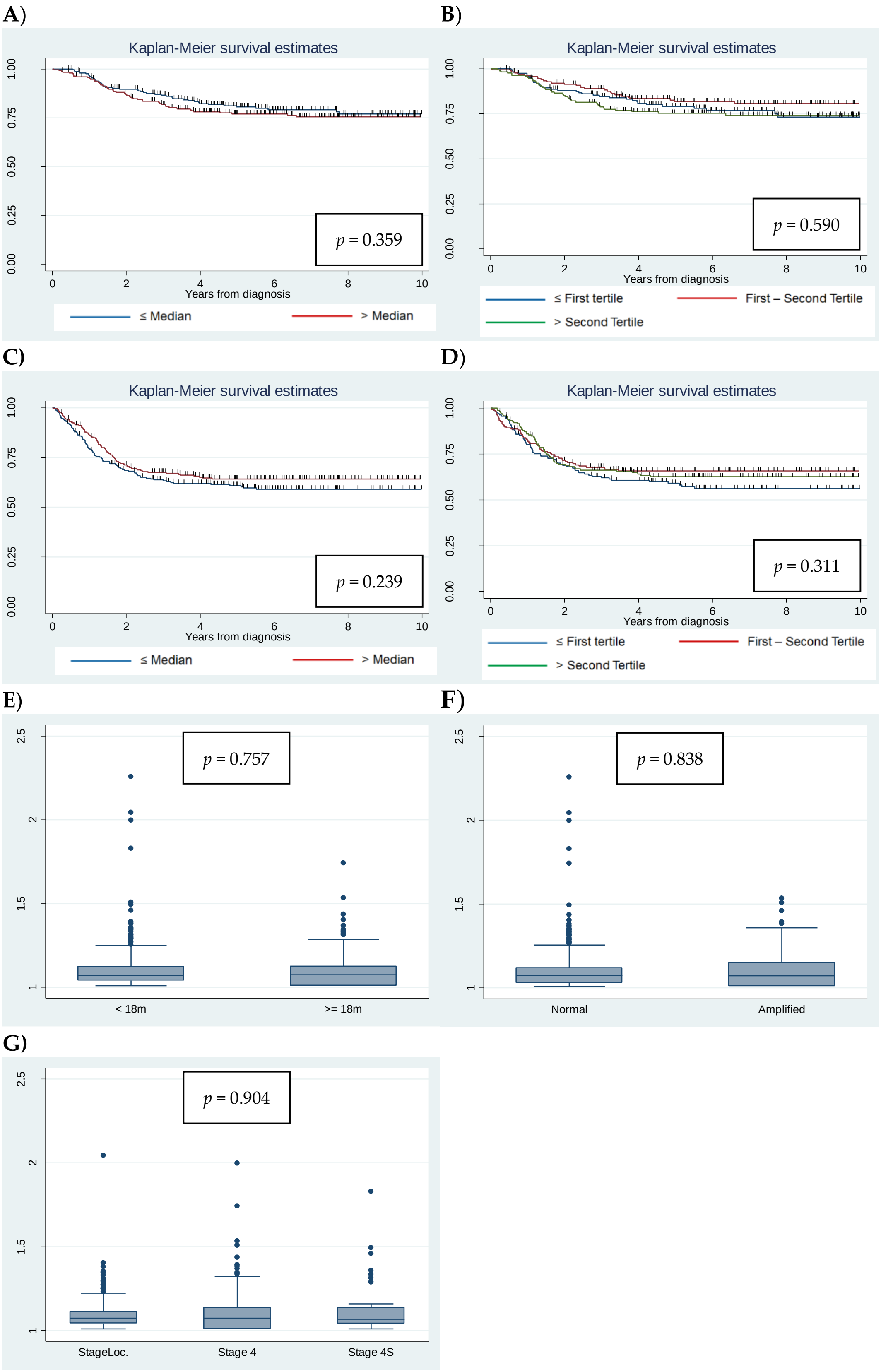


*IFIT1L* (alias *IFIT1B*) gene expression and its association with survival and clinical characteristics in a cohort of 498 NB patients in the SEQC-498. *in silico* dataset. (A) Overall survival, cut-off based on the median expression value; (B) Overall survival, cut-off based on tertile expression values; (C) Event free survival, cut-off based on the median expression value; (D) Event free survival, cut-off based on tertile expression values; E) Age at diagnosis; (F) *MYCN* status; (G) INSS stage.

## Supplementary Figure S46

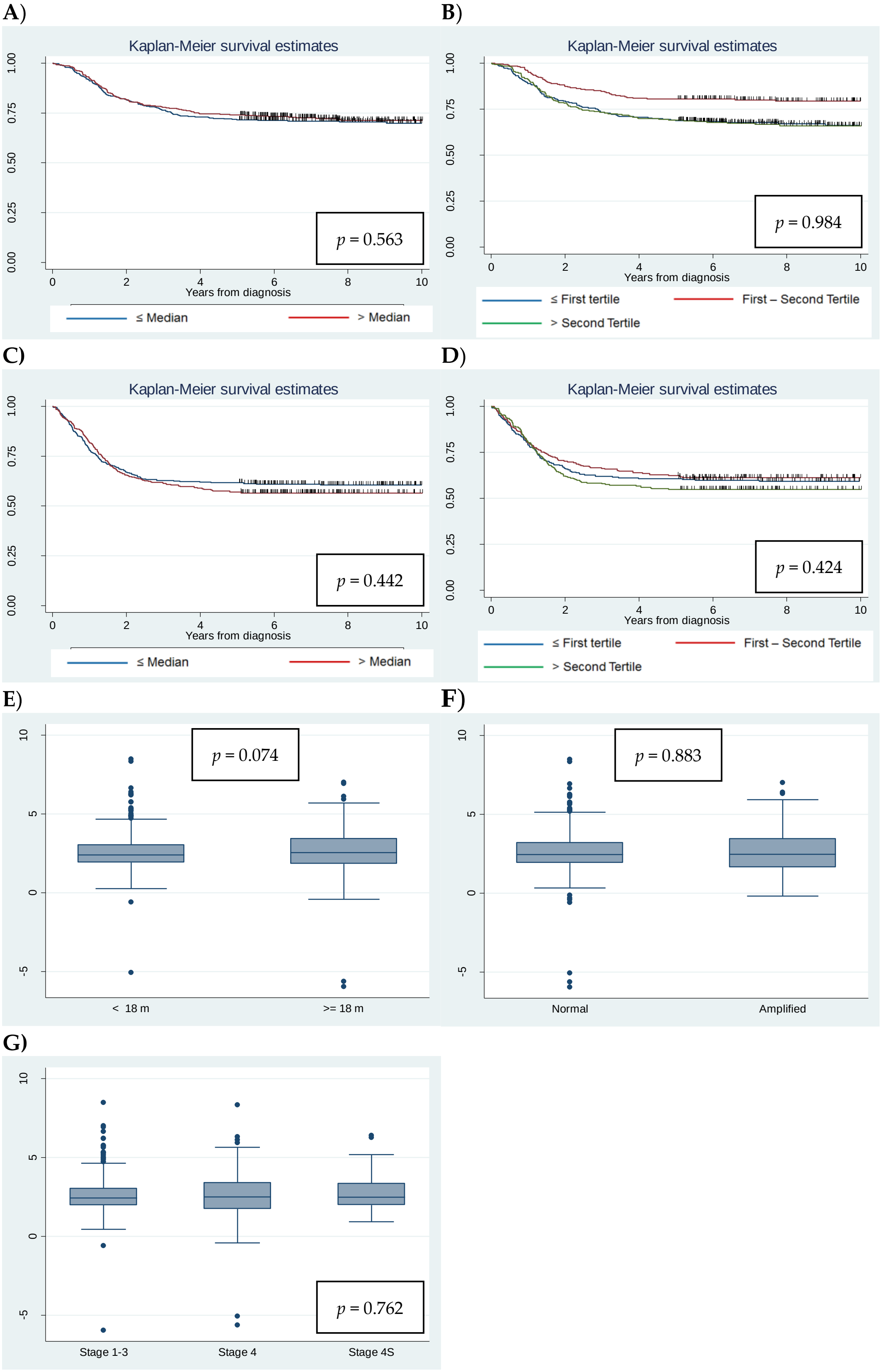


*IFIT3* gene expression and its association with survival and clinical characteristics in a cohort of 786 NB patients in the Cangelosi et al. *in silico* dataset. (A) Overall survival, cut-off based on the median expression value; (B) Overall survival, cut-off based on tertile expression values; (C) Event free survival, cut-off based on the median expression value; (D) Event free survival, cut-off based on tertile expression values; E) Age at diagnosis; (F) *MYCN* status; (G) INSS stage.

**Supplementary Figure S47**

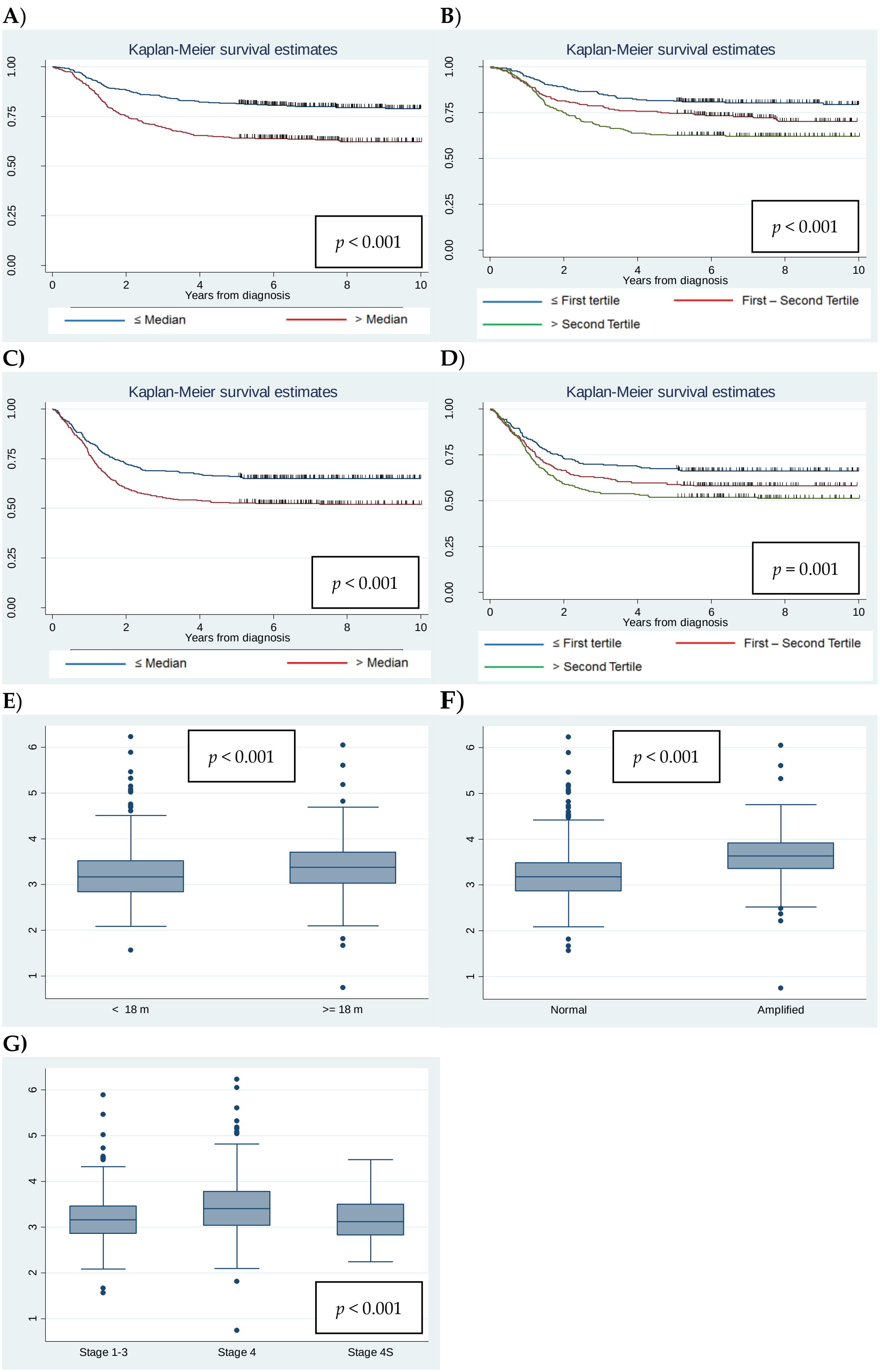


*IFIT5* gene expression and its association with survival and clinical characteristics in a cohort of 786 NB patients in the Cangelosi et al. *in silico* dataset. (A) Overall survival, cut-off based on the median expression value; (B) Overall survival, cut-off based on tertile expression values; (C) Event free survival, cut-off based on the median expression value; (D) Event free survival, cut-off based on tertile expression values; E) Age at diagnosis; (F) *MYCN* status; (G) INSS stage.

**Supplementary Figure S48**

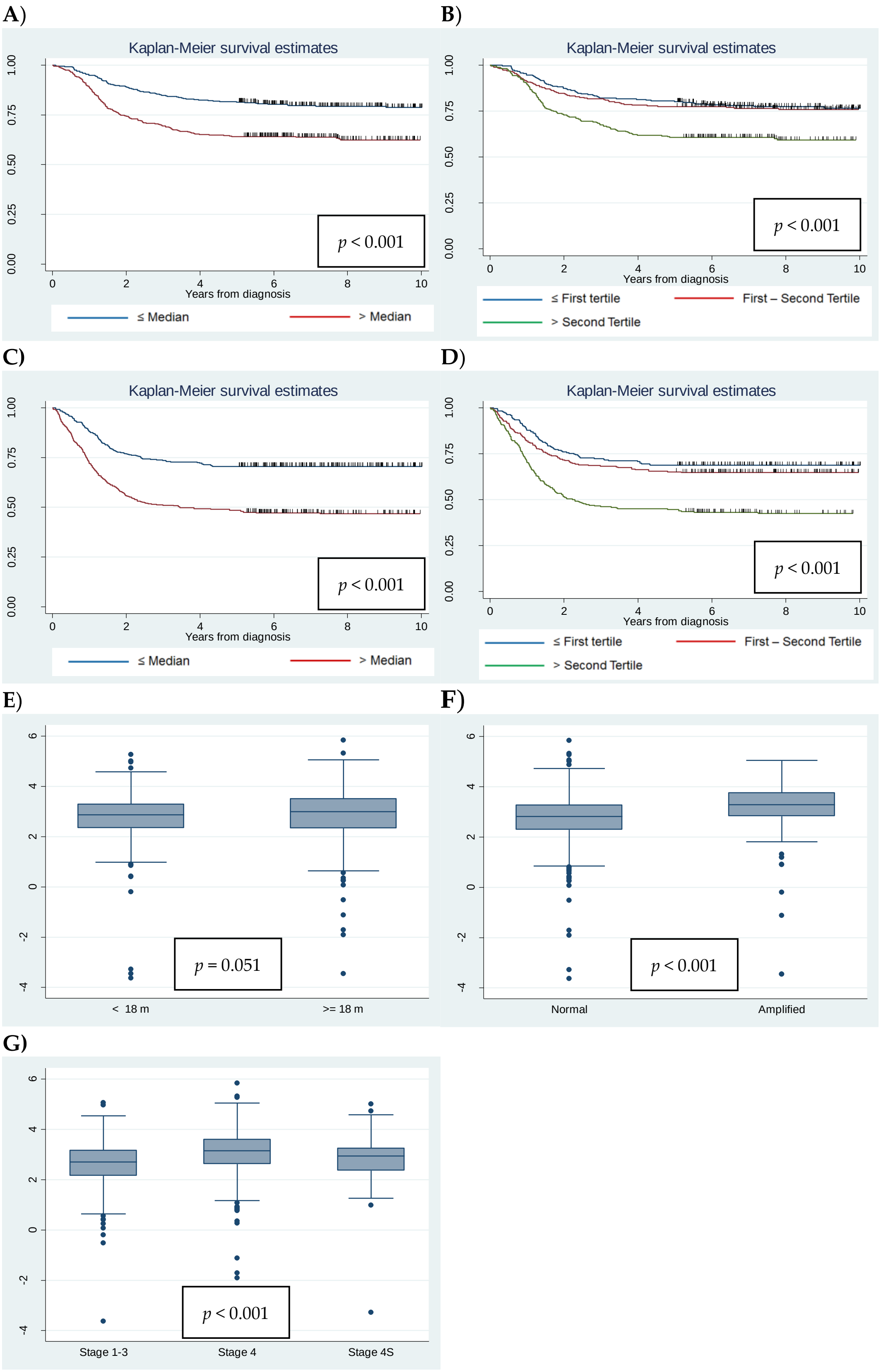


*KIF20B* gene expression and its association with survival and clinical characteristics in a cohort of 786 NB patients in the Cangelosi et al. *in silico* dataset. (A) Overall survival, cut-off based on the median expression value; (B) Overall survival, cut-off based on tertile expression values; (C) Event free survival, cut-off based on the median expression value; (D) Event free survival, cut-off based on tertile expression values; E) Age at diagnosis; (F) *MYCN* status; (G) INSS stage.

## Supplementary Figure S49

A)

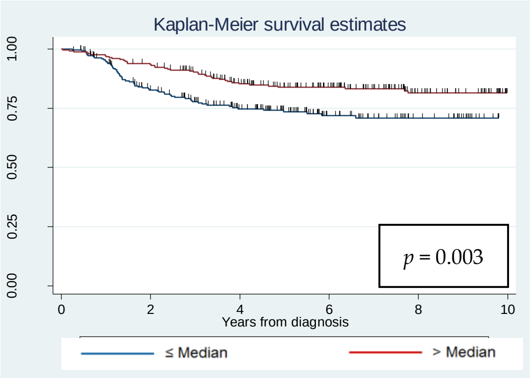


B)

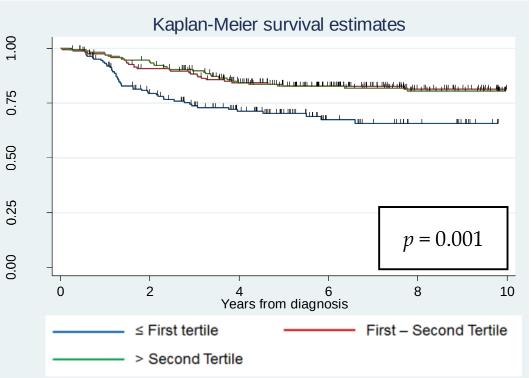


C)

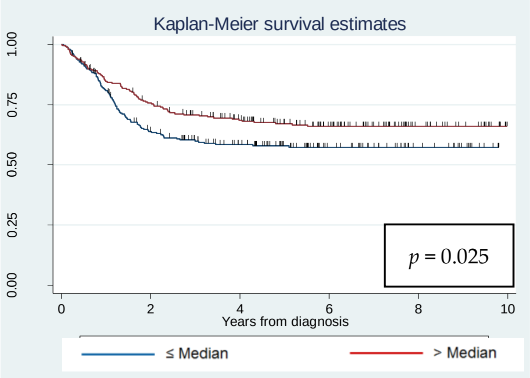


D) [p = 0.007]

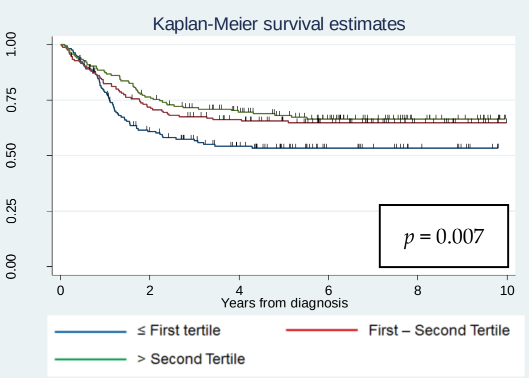


E)

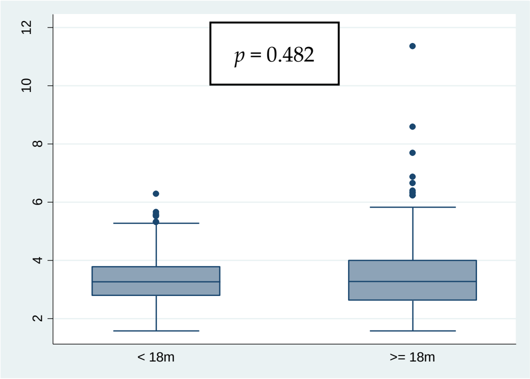


F)

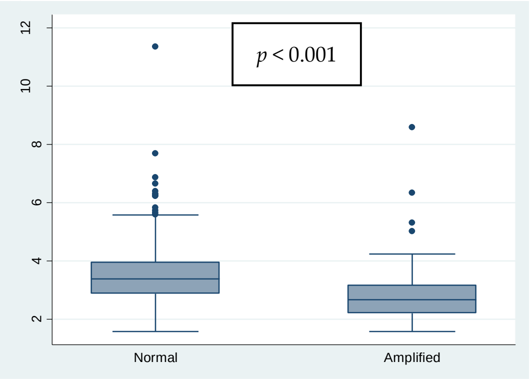


G)

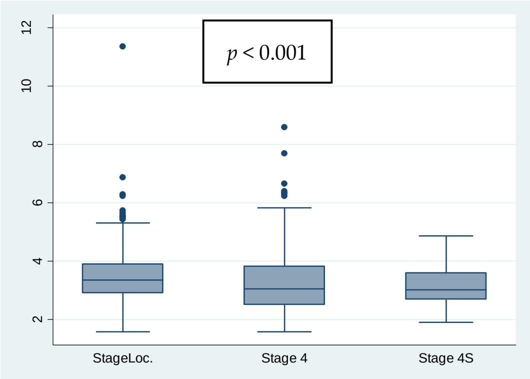


*KILLIN* (alia *KLLN*) gene expression and its association with survival and clinical characteristics in a cohort of 498 NB patients in the SEQC-498 *in silico* dataset. (A) Overall survival, cut-off based on the median expression value; (B) Overall survival, cut-off based on tertile expression values; (C) Event free survival, cut-off based on the median expression value; (D) Event free survival, cut-off based on tertile expression values; E) Age at diagnosis; (F) *MYCN* status; (G) INSS stage.

## Supplementary Figure S50

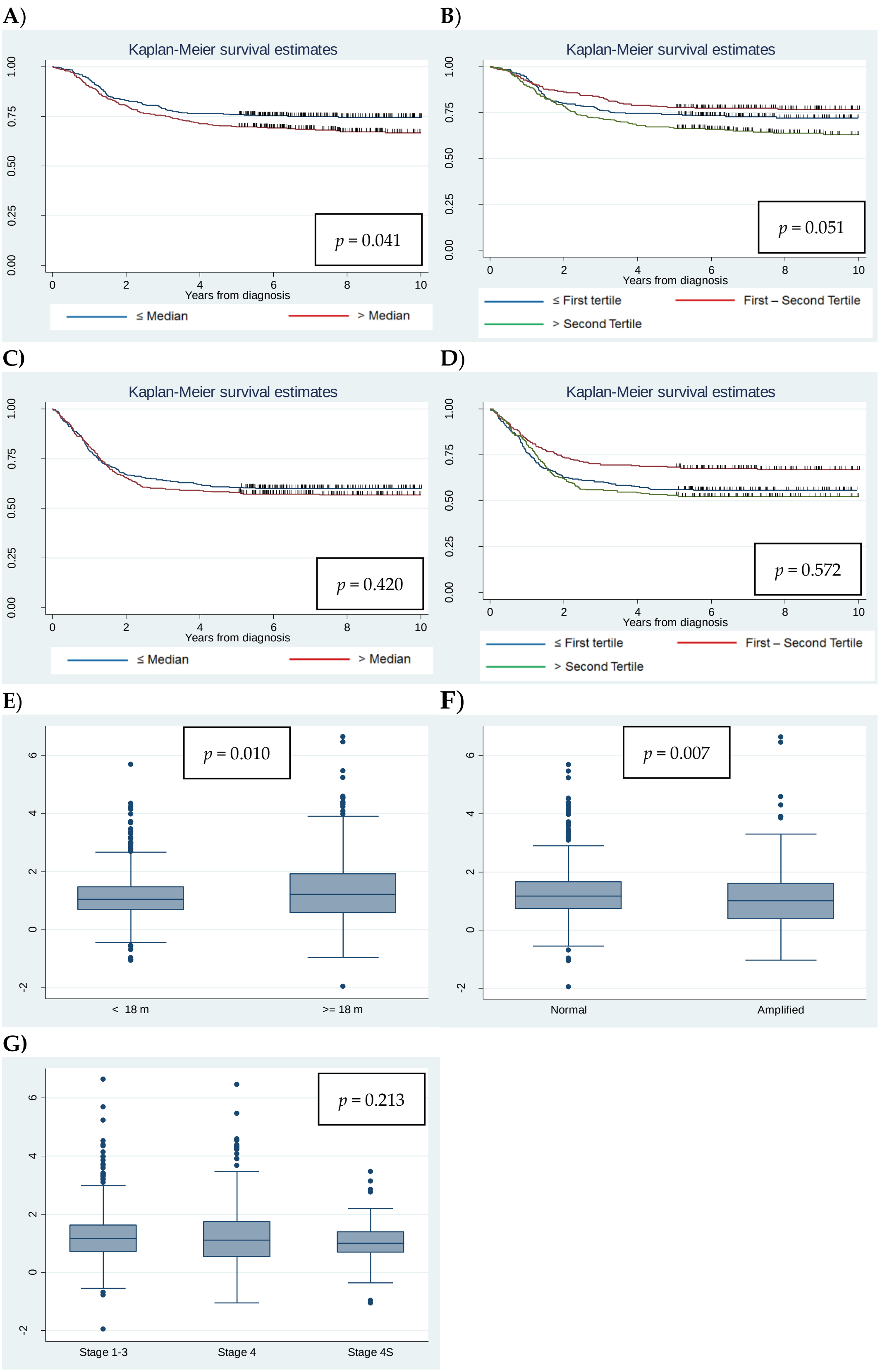


*LDB3* gene expression and its association with survival and clinical characteristics in a cohort of 786 NB patients in the Cangelosi et al. *in silico* dataset. (A) Overall survival, cut-off based on the median expression value; (B) Overall survival, cut-off based on tertile expression values; (C) Event free survival, cut-off based on the median expression value; (D) Event free survival, cut-off based on tertile expression values; E) Age at diagnosis; (F) *MYCN* status; (G) INSS stage.

## Supplementary Figure S51

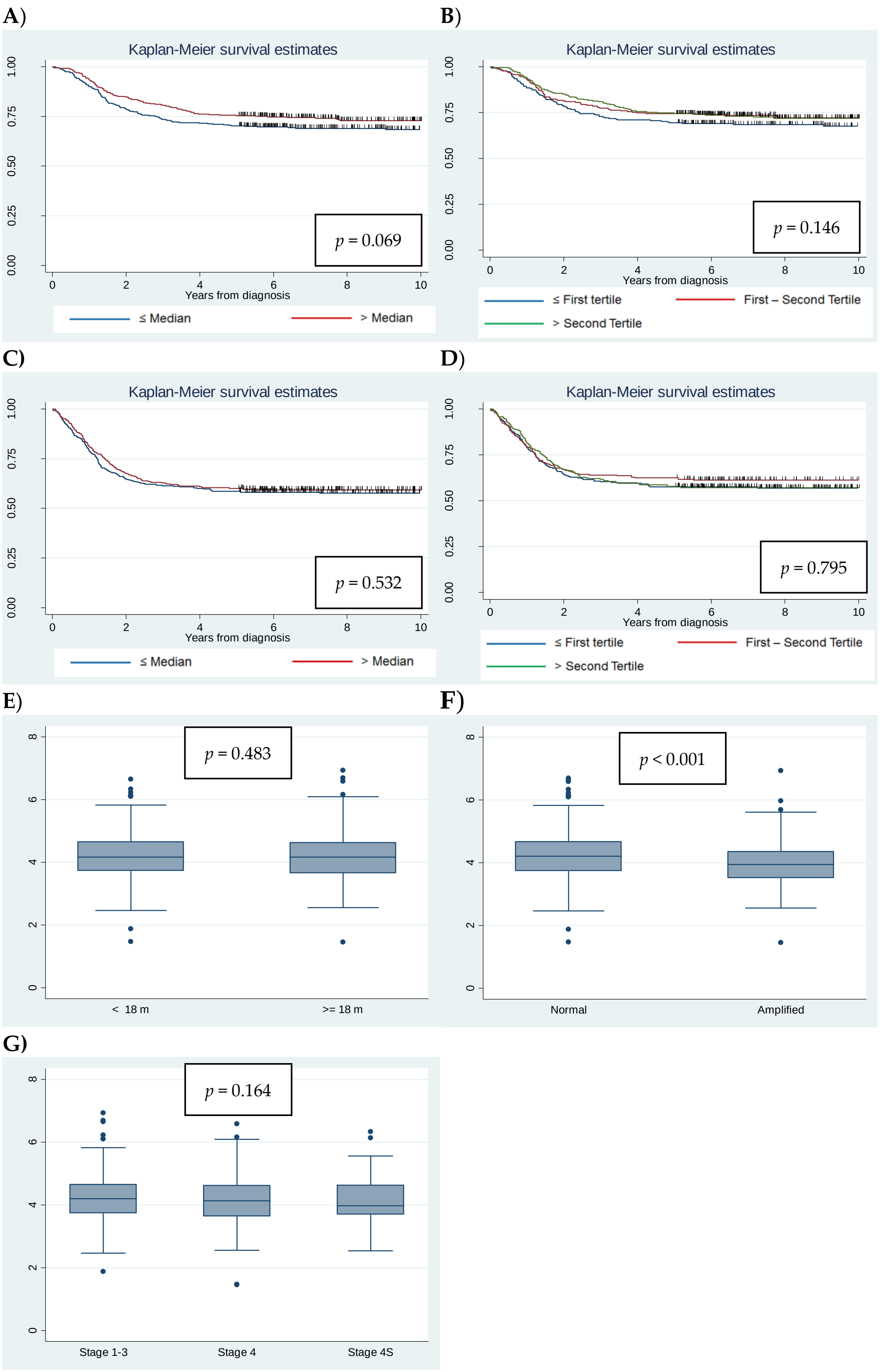


*LIPA* gene expression and its association with survival and clinical characteristics in a cohort of 786 NB patients in the Cangelosi et al. *in silico* dataset. (A) Overall survival, cut-off based on the median expression value; (B) Overall survival, cut-off based on tertile expression values; (C) Event free survival, cut-off based on the median expression value; (D) Event free survival, cut-off based on tertile expression values; E) Age at diagnosis; (F) *MYCN* status; (G) INSS stage.

**Supplementary Figure S52**

**A)**

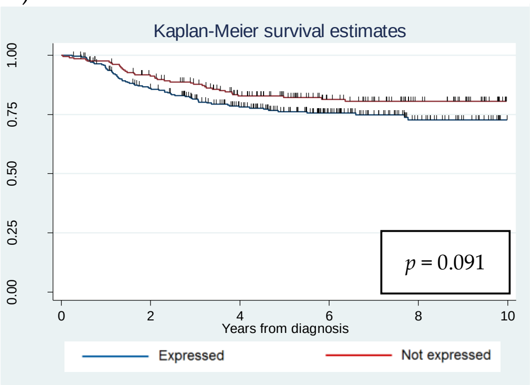


**B)**

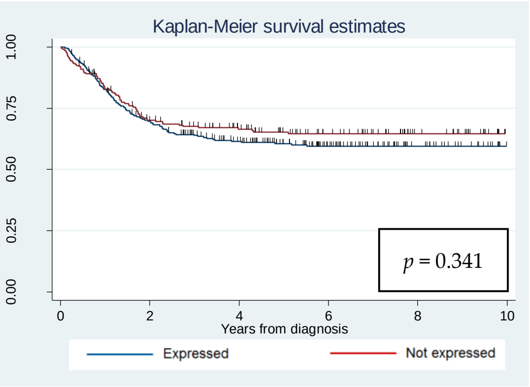


*LIPF* gene expression and its association with survival and clinical characteristics in a cohort of 498 NB patients in the SEQC-498 *in silico* dataset. (A) Overall survival, gene expression yes/not; (B) Event Free Survival, gene expression yes/not.

## Supplementary Figure S53

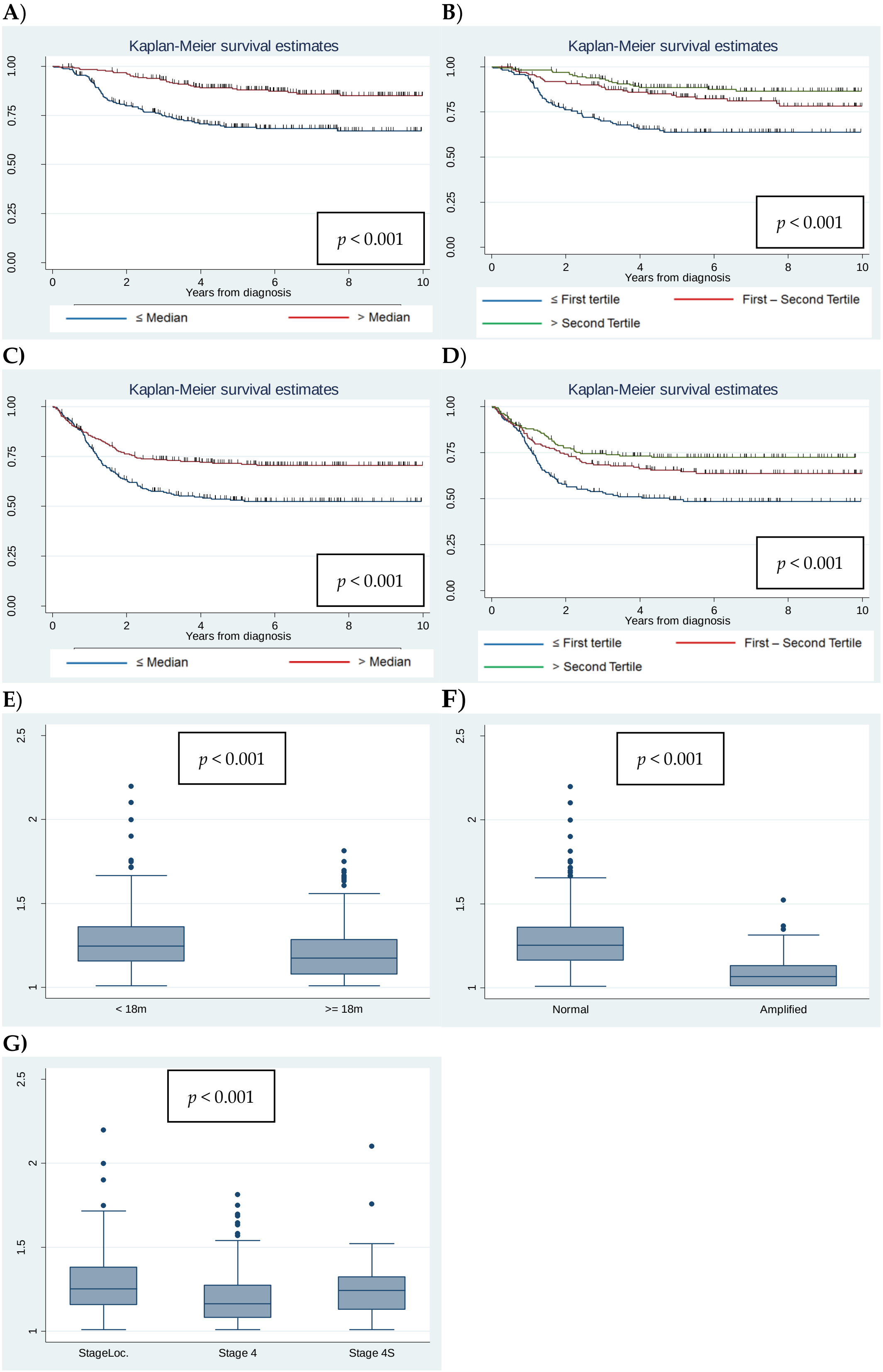


*LIPJ* gene expression and its association with survival and clinical characteristics in a cohort of 498 NB patients in the SEQC-498 *in silico* dataset. (A) Overall survival, cut-off based on the median expression value; (B) Overall survival, cut-off based on tertile expression values; (C) Event free survival, cut-off based on the median expression value; (D) Event free survival, cut-off based on tertile expression values; E) Age at diagnosis; (F) *MYCN* status; (G) INSS stage.

**Supplementary Figure S54**

A) B)

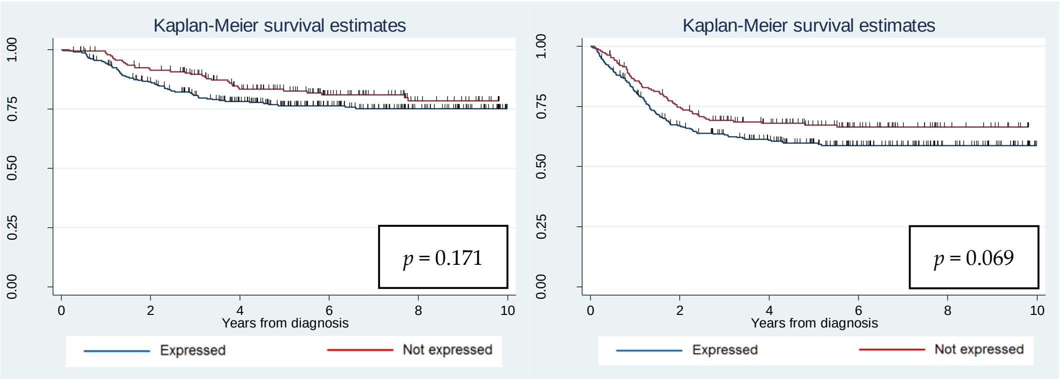


*LIPM* gene expression and its association with survival and clinical characteristics in a cohort of 498 NB patients in the SEQC-498 *in silico* dataset. (A) Overall survival, gene expression yes/not; (B) Event Free Survival, gene expression yes/not.

**Supplementary Figure S55**

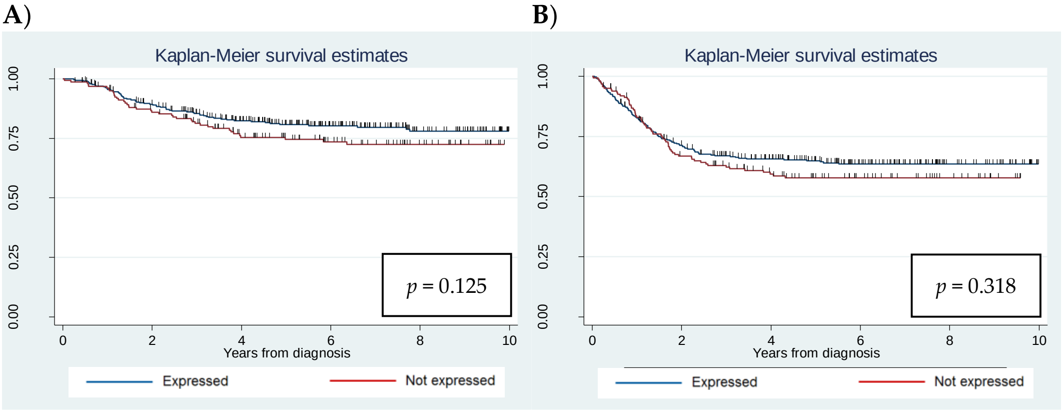


*LIPN* gene expression and its association with survival and clinical characteristics in a cohort of 498 NB patients in the SEQC-498 *in silico* dataset. (A) Overall survival, gene expression yes/not; (B) Event Free Survival, gene expression yes/not.

**Supplementary Figure S56**

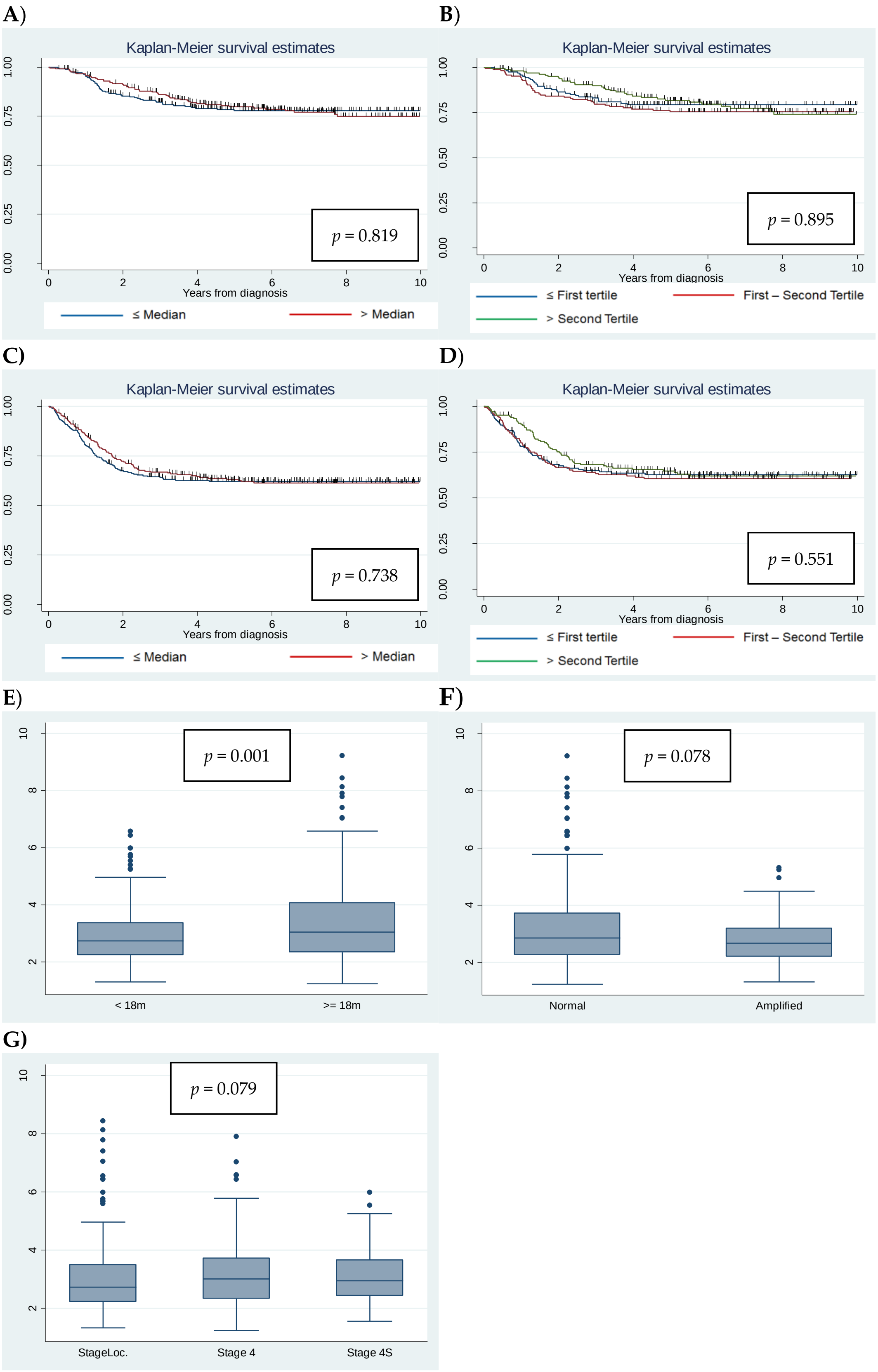


*LOC219347(alias: TMEM254AS1)* gene expression and its association with survival and clinical characteristics in a cohort of 498 NB patients in the SEQC-498. *in silico* dataset. (A) Overall survival, cut-off based on the median expression value; (B) Overall survival, cut-off based on tertile expression values; (C) Event free survival, cut-off based on the median expression value; (D) Event free survival, cut-off based on tertile expression values; E) Age at diagnosis; (F) *MYCN* status; (G) INSS stage.

## Supplementary Figure S57

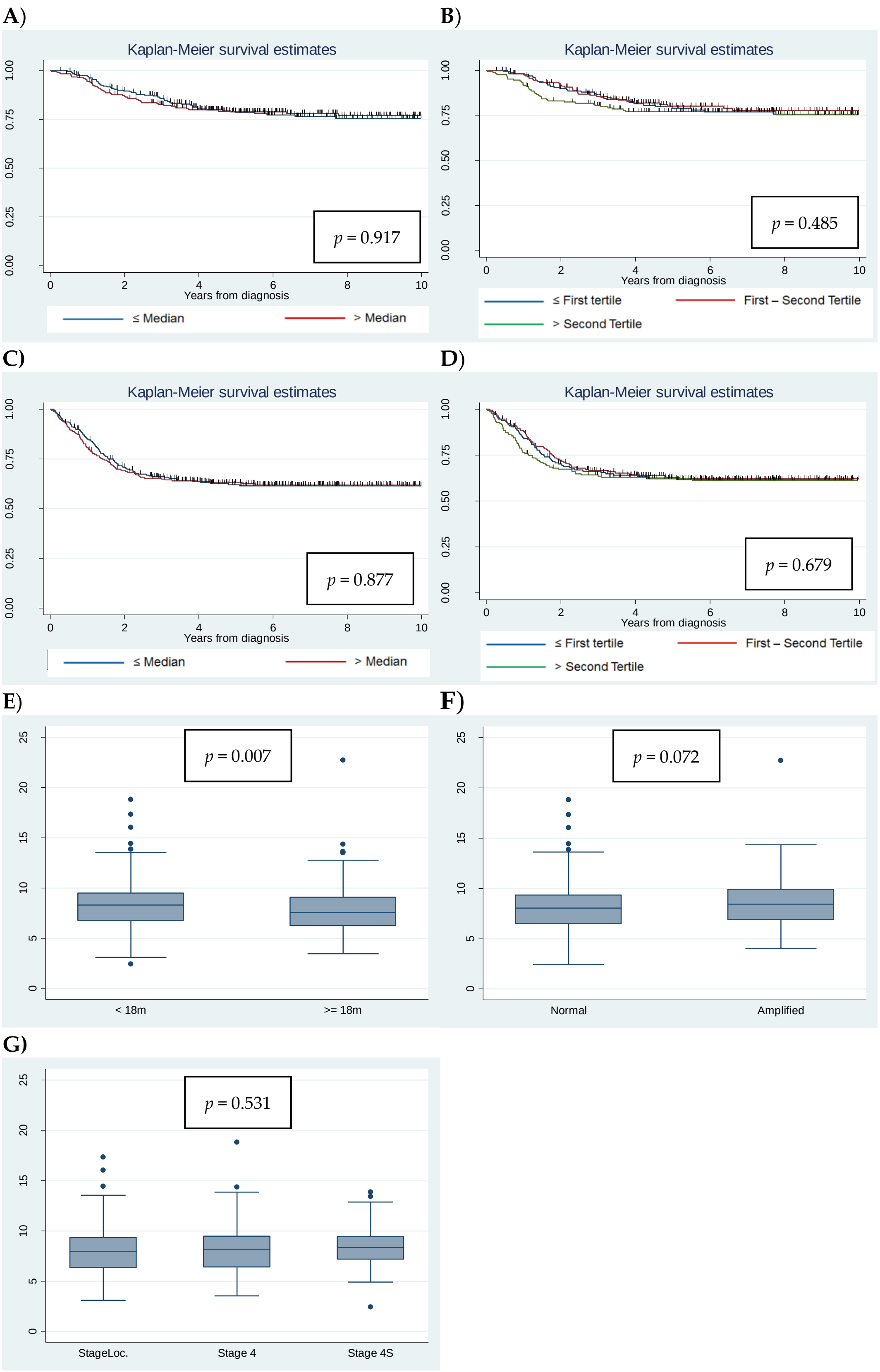


*LOC439994* gene expression and its association with survival and clinical characteristics in a cohort of 498 NB patients in the SEQC-498 *in silico* dataset. (A) Overall survival, cut-off based on the median expression value; (B) Overall survival, cut-off based on tertile expression values; (C) Event free survival, cut-off based on the median expression value; (D) Event free survival, cut-off based on tertile expression values; E) Age at diagnosis; (F) *MYCN* status; (G) INSS stage.

## Supplementary Figure S58

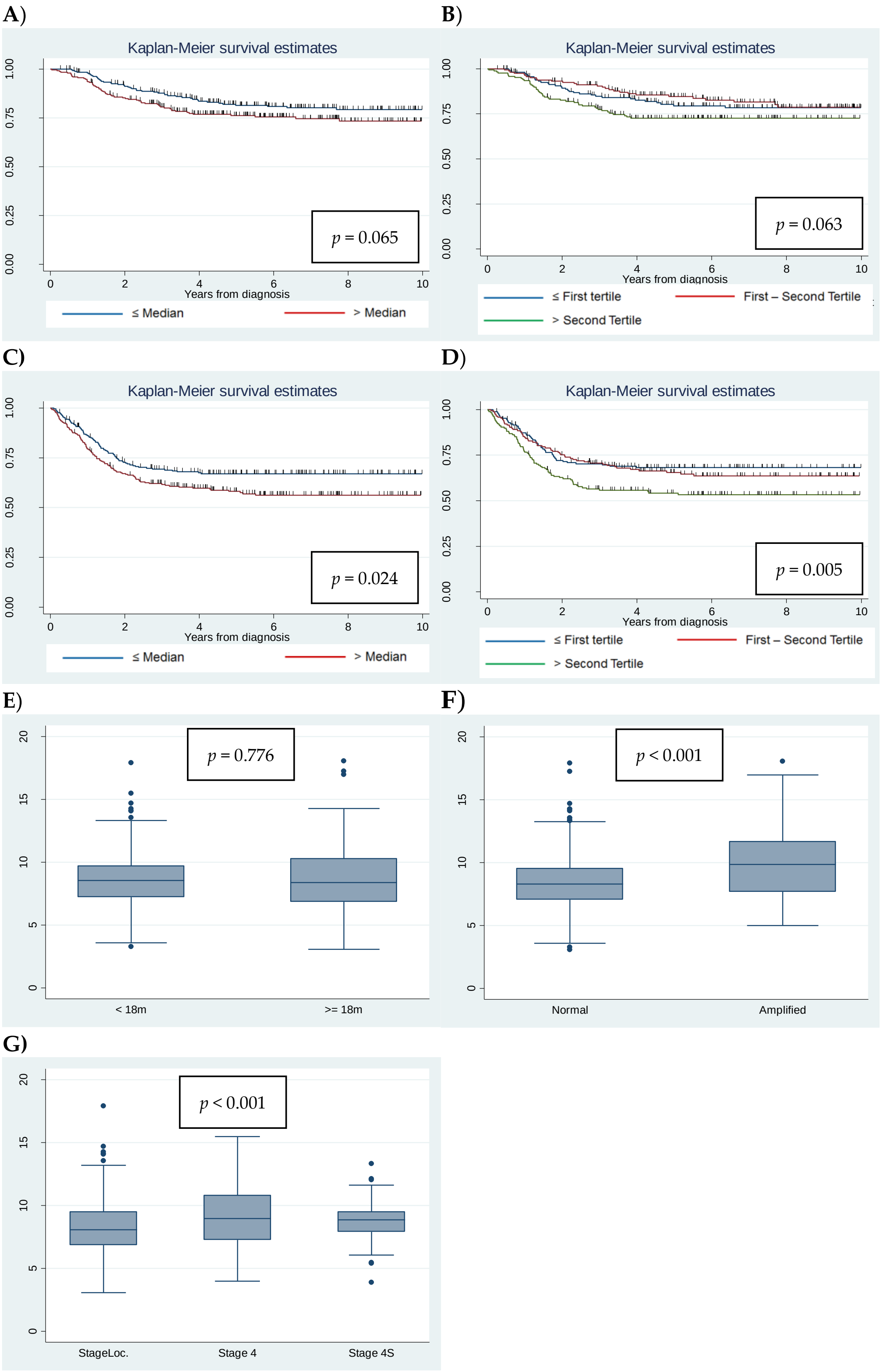


*LOC642361* gene expression and its association with survival and clinical characteristics in a cohort of 498 NB patients in the SEQC-498 *in silico* dataset. (A) Overall survival, cut-off based on the median expression value; (B) Overall survival, cut-off based on tertile expression values; (C) Event free survival, cut-off based on the median expression value; (D) Event free survival, cut-off based on tertile expression values; E) Age at diagnosis; (F) *MYCN* status; (G) INSS stage.

**Supplementary Figure S59**

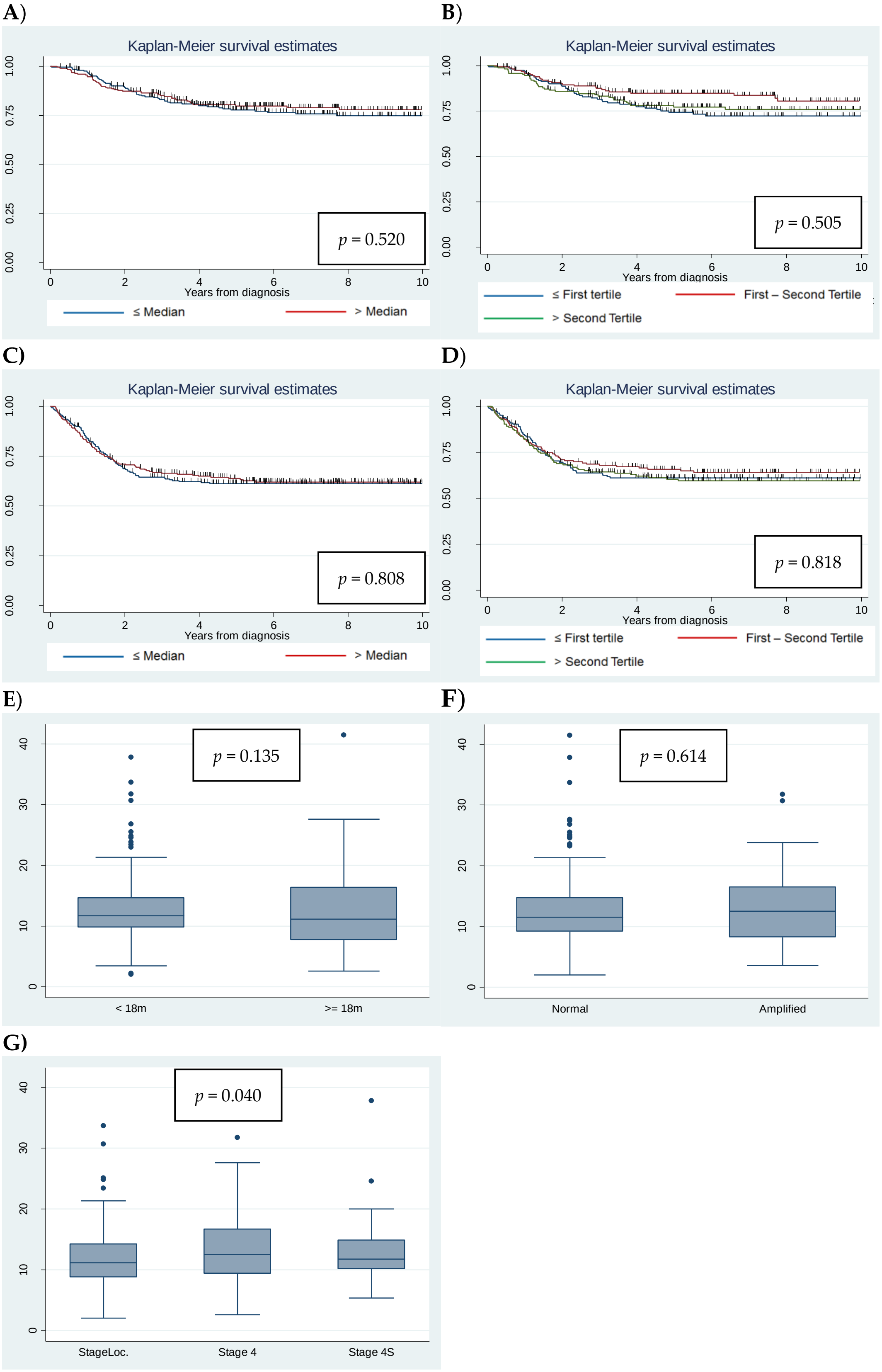


*LOC650623* gene expression and its association with survival and clinical characteristics in a cohort of 498 NB patients in the SEQC-498 *in silico* dataset. (A) Overall survival, cut-off based on the median expression value; (B) Overall survival, cut-off based on tertile expression values; (C) Event free survival, cut-off based on the median expression value; (D) Event free survival, cut-off based on tertile expression values; E) Age at diagnosis; (F) *MYCN* status; (G) INSS stage.

**Supplementary Figure S60**

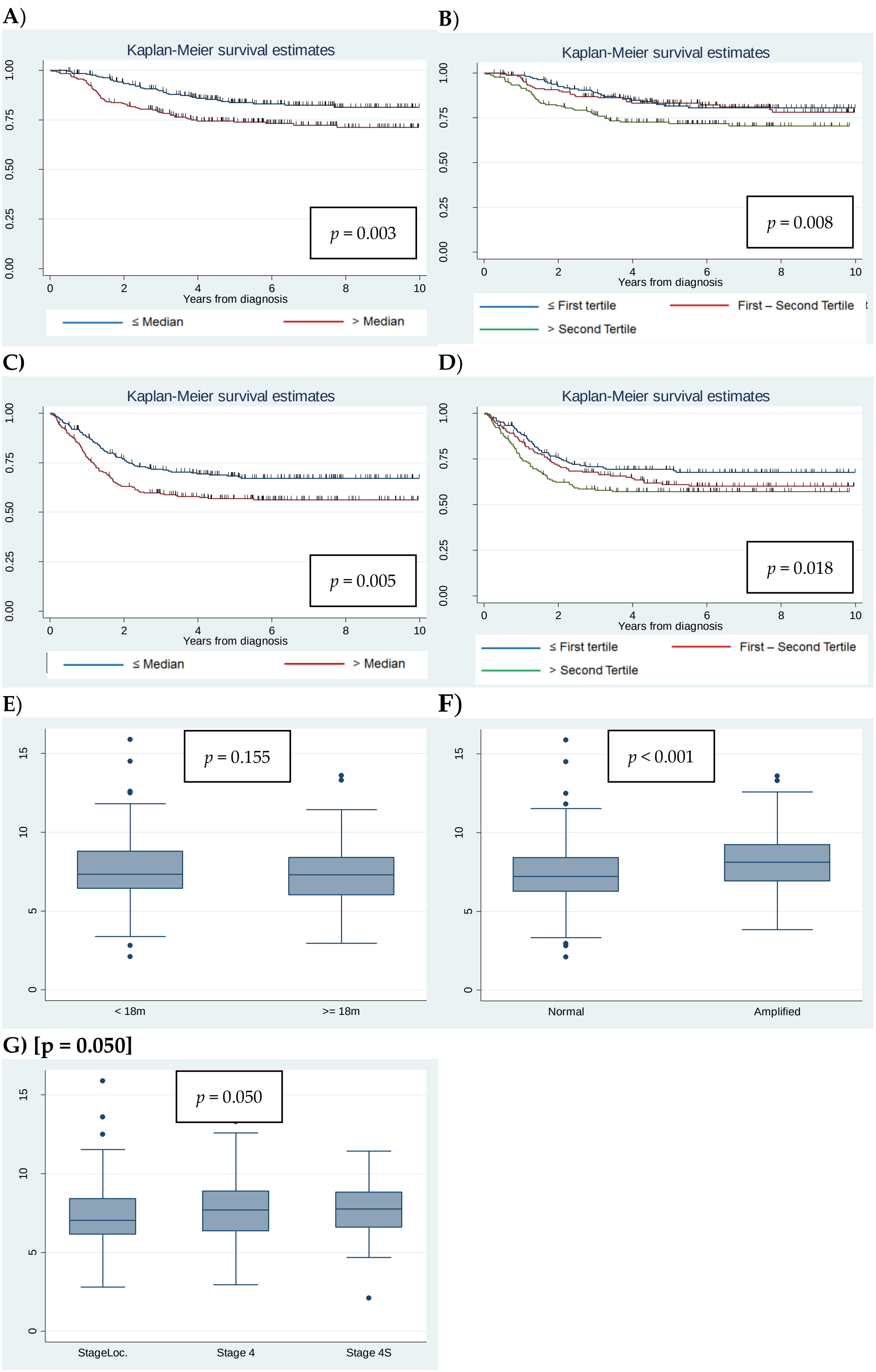


*LOC728190* gene expression and its association with survival and clinical characteristics in a cohort of 498 NB patients in the SEQC-498 *in silico* dataset. (A) Overall survival, cut-off based on the median expression value; (B) Overall survival, cut-off based on tertile expression values; (C) Event free survival, cut-off based on the median expression value; (D) Event free survival, cut-off based on tertile expression values; E) Age at diagnosis; (F) *MYCN* status; (G) INSS stage.

**Supplementary Figure S61**

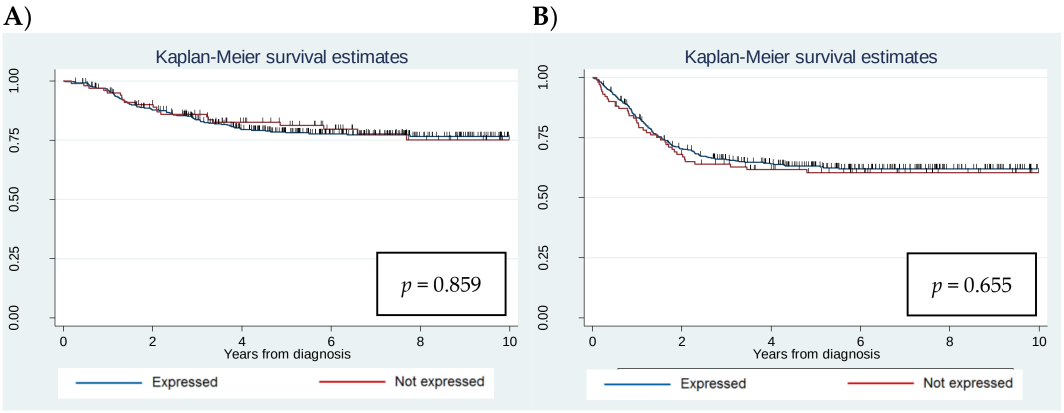


*LRIT1* gene expression and its association with survival and clinical characteristics in a cohort of 498 NB patients in the SEQC-498 *in silico* dataset. (A) Overall survival, gene expression yes/not; (B) Event Free Survival, gene expression yes/not.

**Supplementary Figure S62**

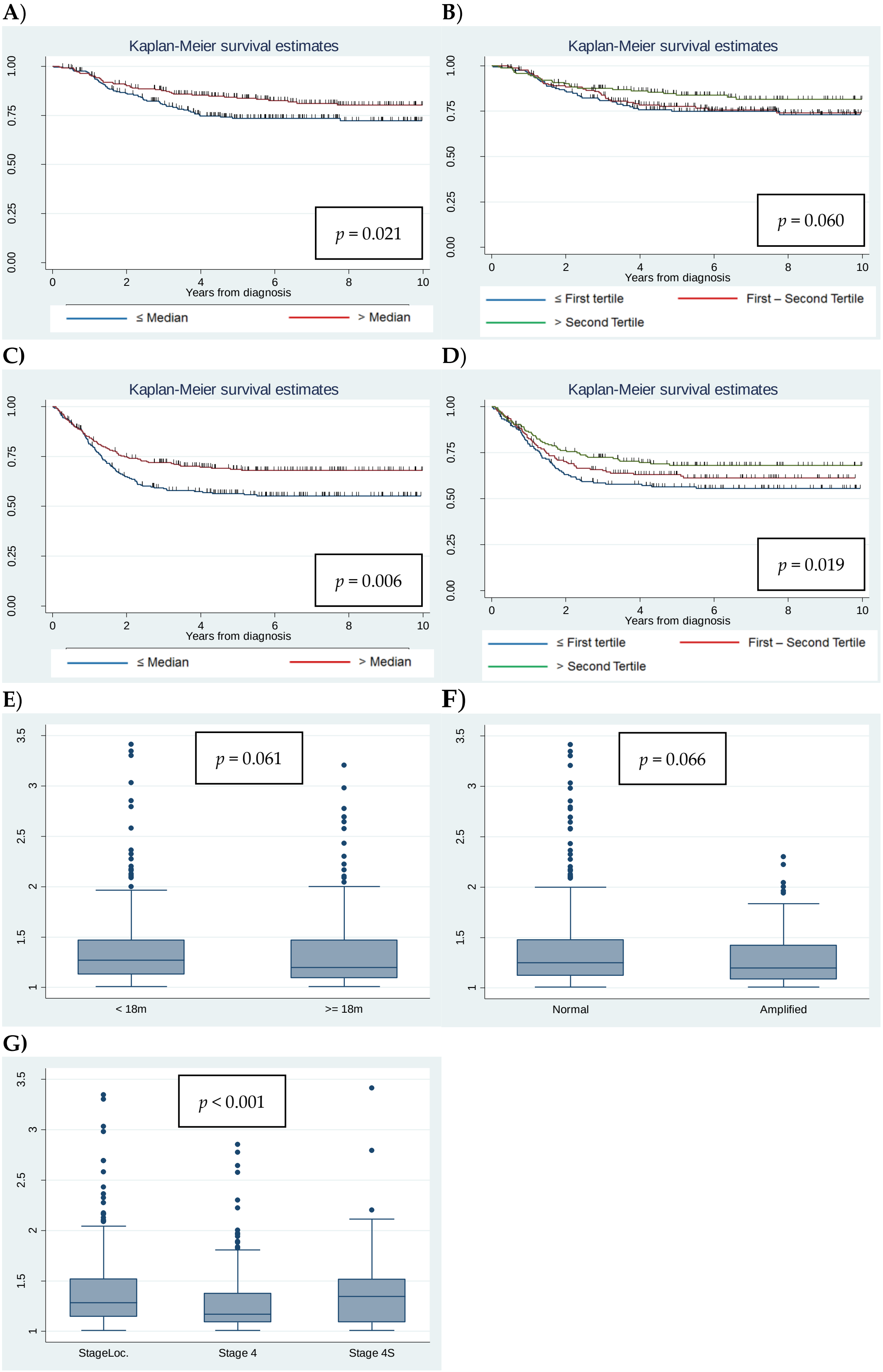


*LRIT2* gene expression and its association with survival and clinical characteristics in a cohort of 498 NB patients in the SEQC-498. *in silico* dataset. (A) Overall survival, cut-off based on the median expression value; (B) Overall survival, cut-off based on tertile expression values; (C) Event free survival, cut-off based on the median expression value; (D) Event free survival, cut-off based on tertile expression values; E) Age at diagnosis; (F) *MYCN* status; (G) INSS stage.

**Supplementary Figure S63**

**A)**

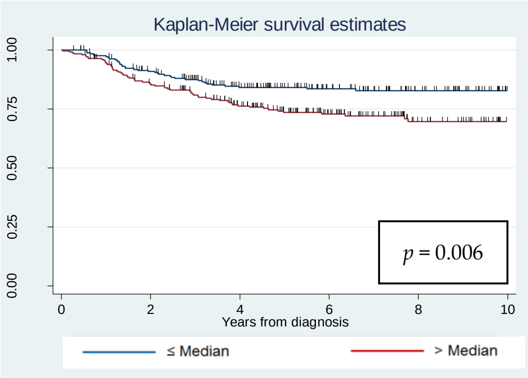


**B)**

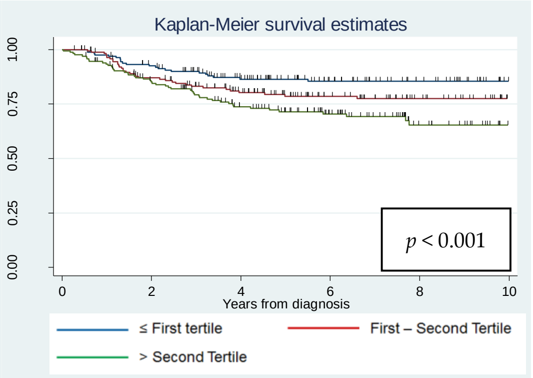


**C)**

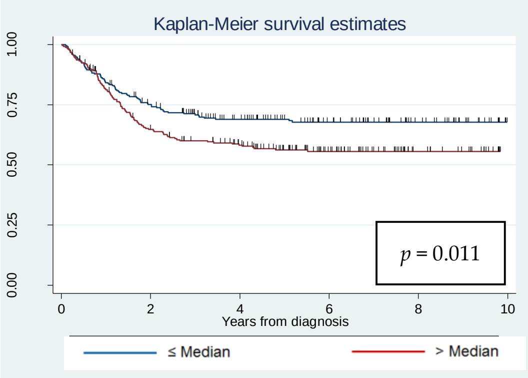


**D)**

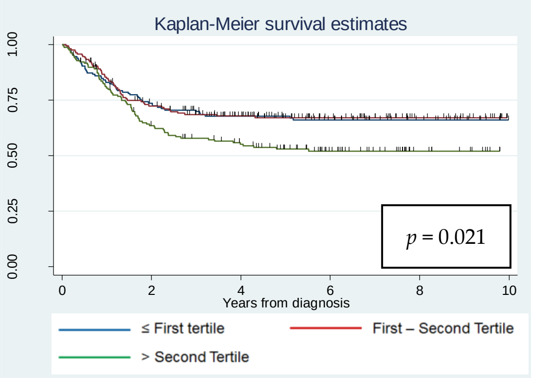


**E)**

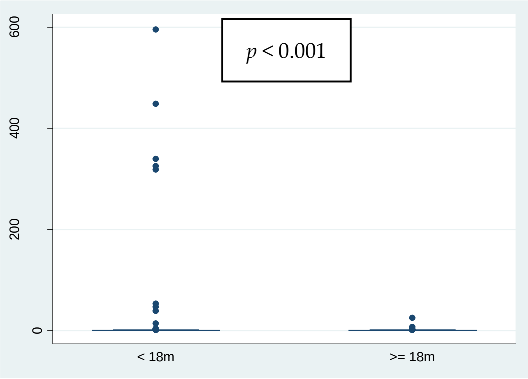


**F)** [p = 0.005]

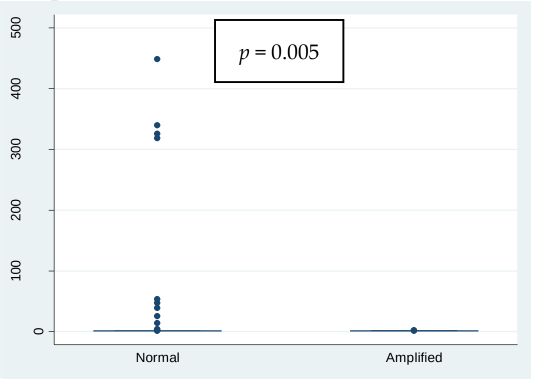


**G)**

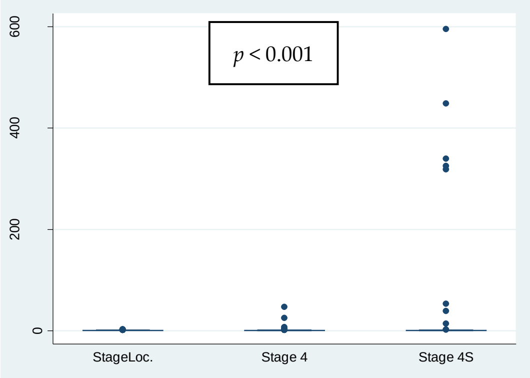


*MAT1A* gene expression and its association with survival and clinical characteristics in a cohort of 498 NB patients in the SEQC-498. *in silico* dataset. (A) Overall survival, cut-off based on the median expression value; (B) Overall survival, cut-off based on tertile expression values; (C) Event free survival, cut-off based on the median expression value; (D) Event free survival, cut-off based on tertile expression values; E) Age at diagnosis; (F) *MYCN* status; (G) INSS stage.

**Supplementary Figure S64**

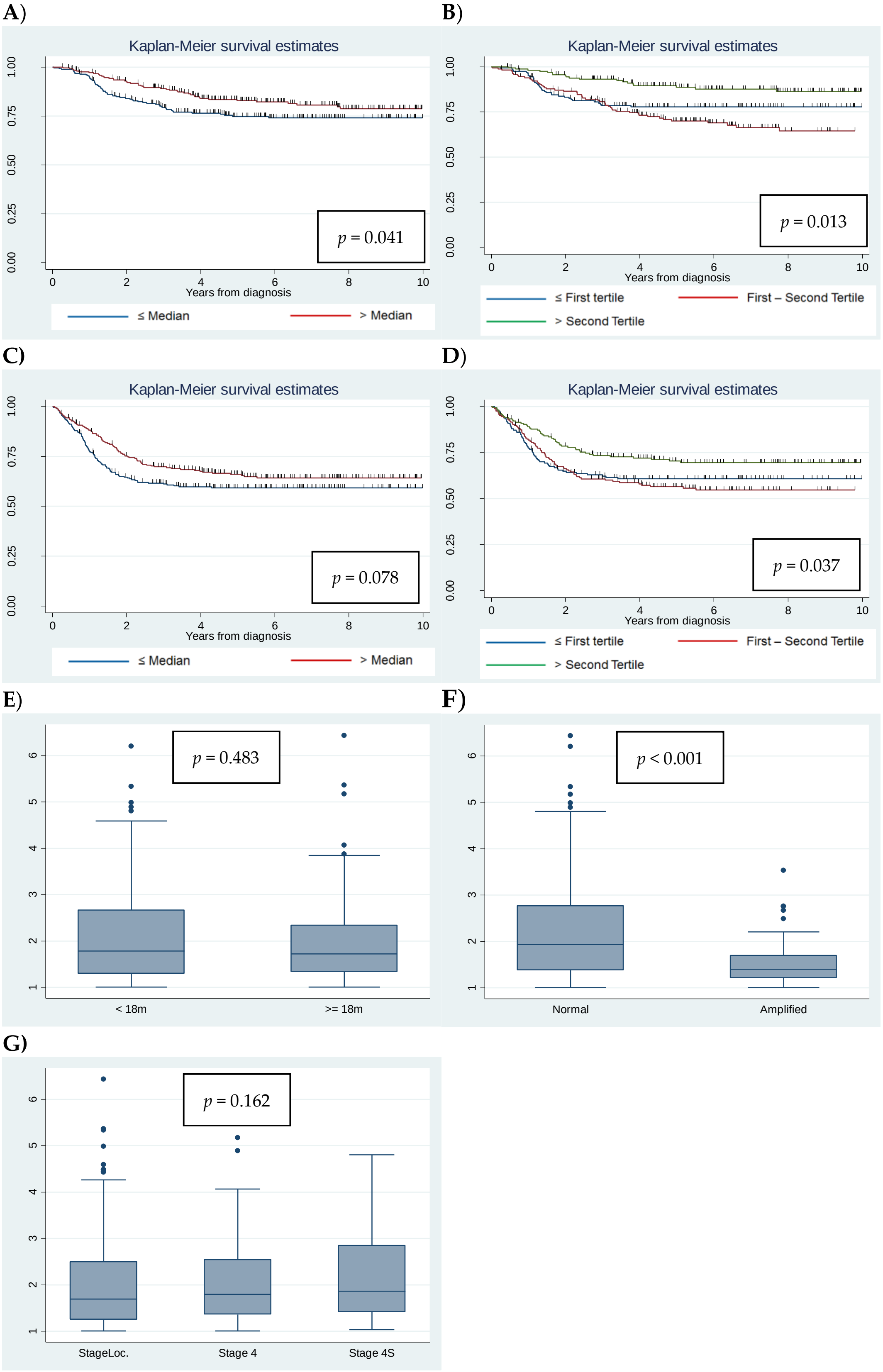


*MBL1P* gene expression and its association with survival and clinical characteristics in a cohort of 498 NB patients in the SEQC-498. *in silico* dataset. (A) Overall survival, cut-off based on the median expression value; (B) Overall survival, cut-off based on tertile expression values; (C) Event free survival, cut-off based on the median expression value; (D) Event free survival, cut-off based on tertile expression values; E) Age at diagnosis; (F) *MYCN* status; (G) INSS stage.

## Supplementary Figure S65

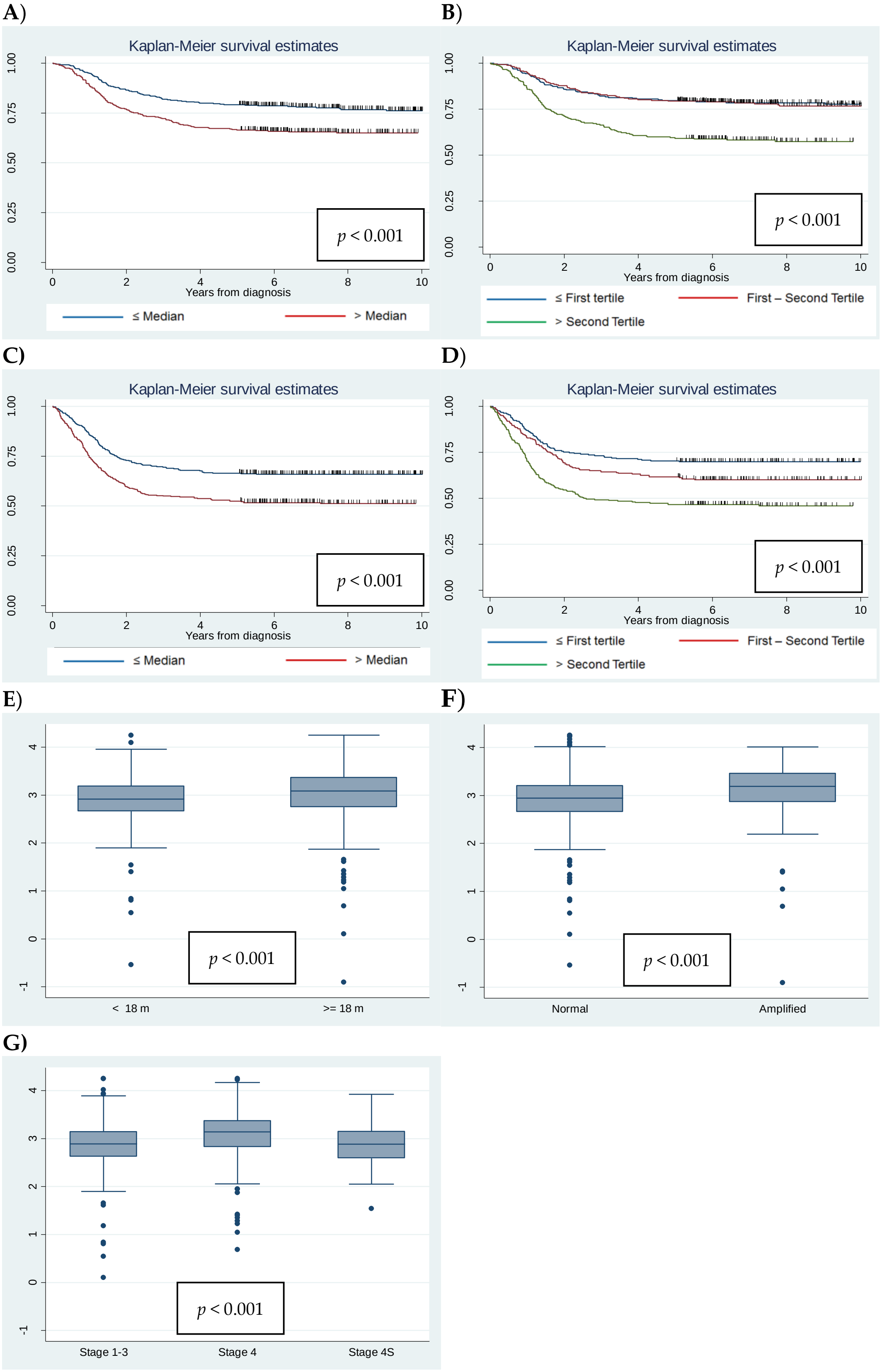


*MINPP1* gene expression and its association with survival and clinical characteristics in a cohort of 786 NB patients in the Cangelosi et al. *in silico* dataset. (A) Overall survival, cut-off based on the median expression value; (B) Overall survival, cut-off based on tertile expression values; (C) Event free survival, cut-off based on the median expression value; (D) Event free survival, cut-off based on tertile expression values; E) Age at diagnosis; (F) *MYCN* status; (G) INSS stage.

## Supplementary Figure S66

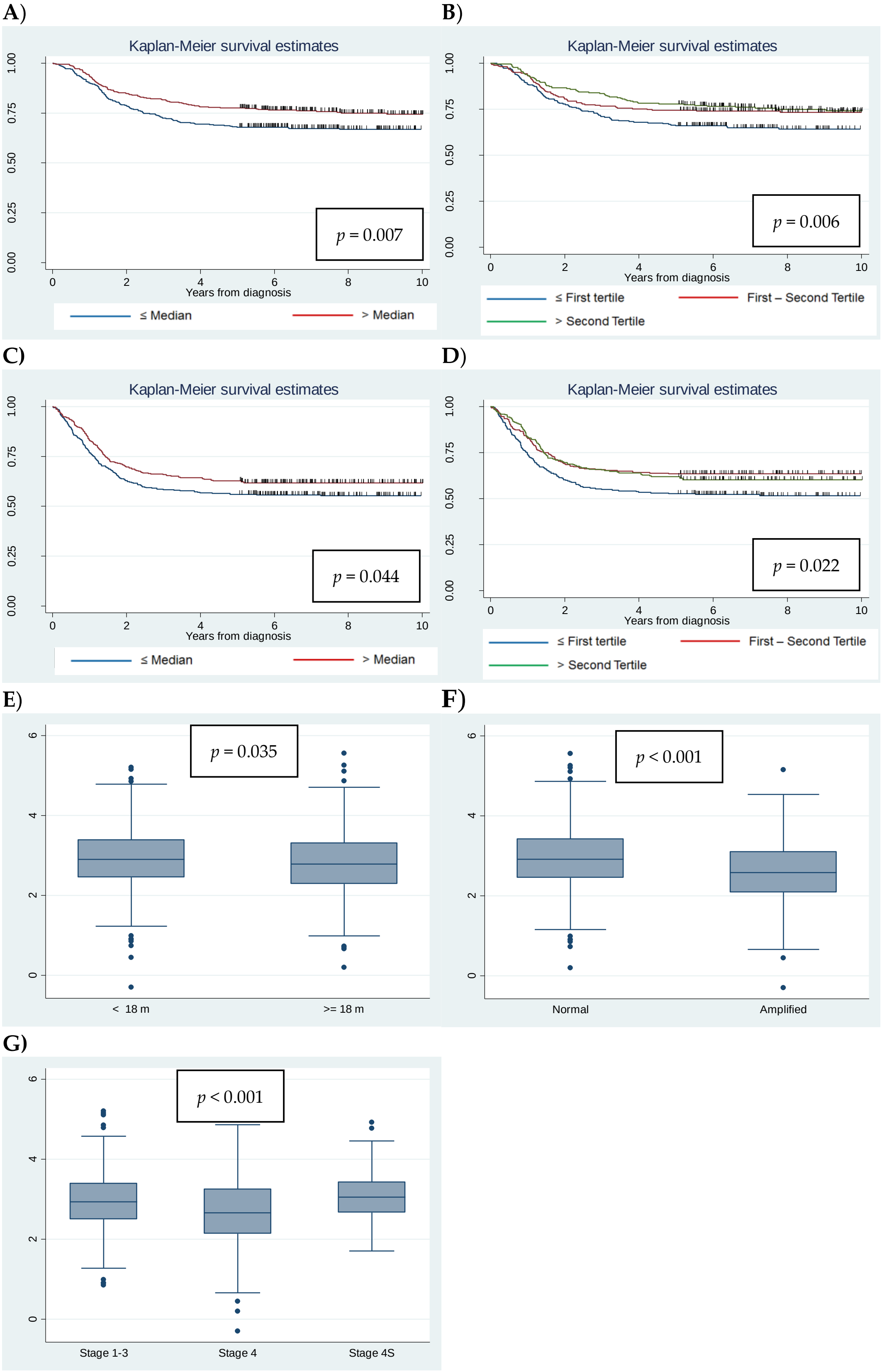


*MMRN2* gene expression and its association with survival and clinical characteristics in a cohort of 786 NB patients in the Cangelosi et al. *in silico* dataset. (A) Overall survival, cut-off based on the median expression value; (B) Overall survival, cut-off based on tertile expression values; (C) Event free survival, cut-off based on the median expression value; (D) Event free survival, cut-off based on tertile expression values; E) Age at diagnosis; (F) *MYCN* status; (G) INSS stage.

**Supplementary Figure S67**

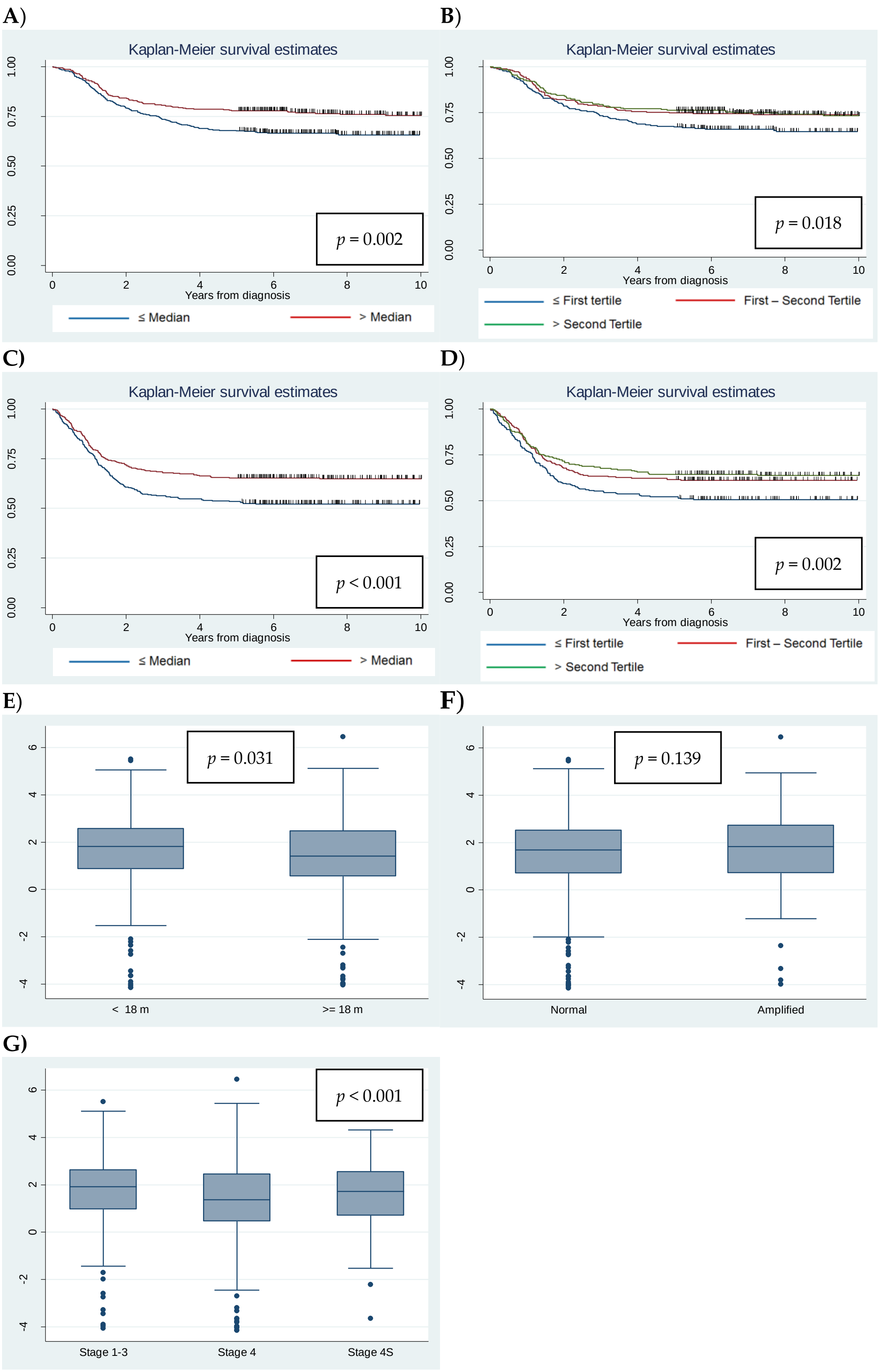


*NRG3* gene expression and its association with survival and clinical characteristics in a cohort of 786 NB patients in the Cangelosi et al. *in silico* dataset. (A) Overall survival, cut-off based on the median expression value; (B) Overall survival, cut-off based on tertile expression values; (C) Event free survival, cut-off based on the median expression value; (D) Event free survival, cut-off based on tertile expression values; E) Age at diagnosis; (F) *MYCN* status; (G) INSS stage.

**Supplementary Figure S68**

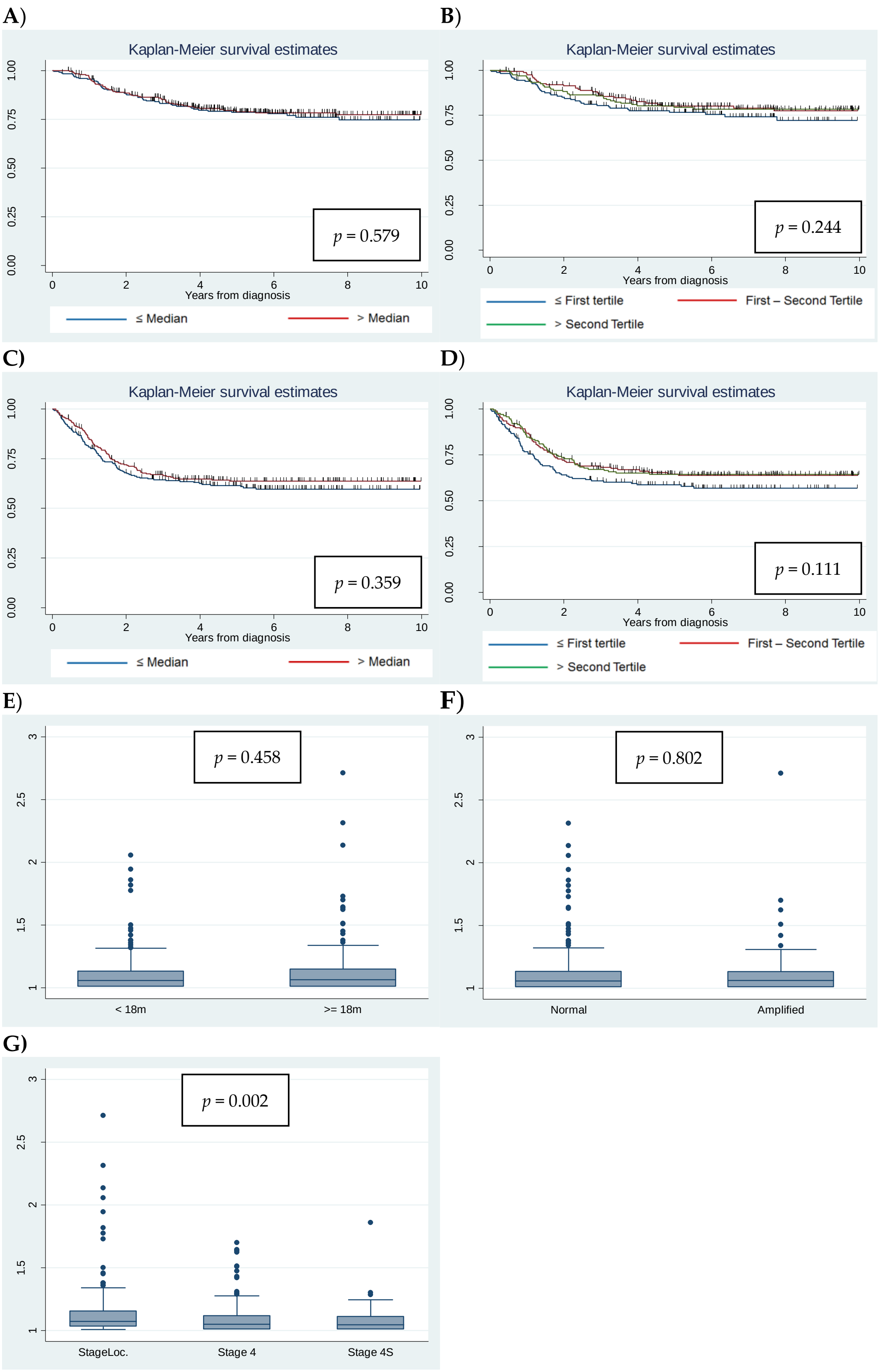


*OPN4* gene expression and its association with survival and clinical characteristics in a cohort of 498 NB patients in the SEQC-498 *in silico* dataset. (A) Overall survival, cut-off based on the median expression value; (B) Overall survival, cut-off based on tertile expression values; (C) Event free survival, cut-off based on the median expression value; (D) Event free survival, cut-off based on tertile expression values; E) Age at diagnosis; (F) *MYCN* status; (G) INSS stage.

## Supplementary Figure S69

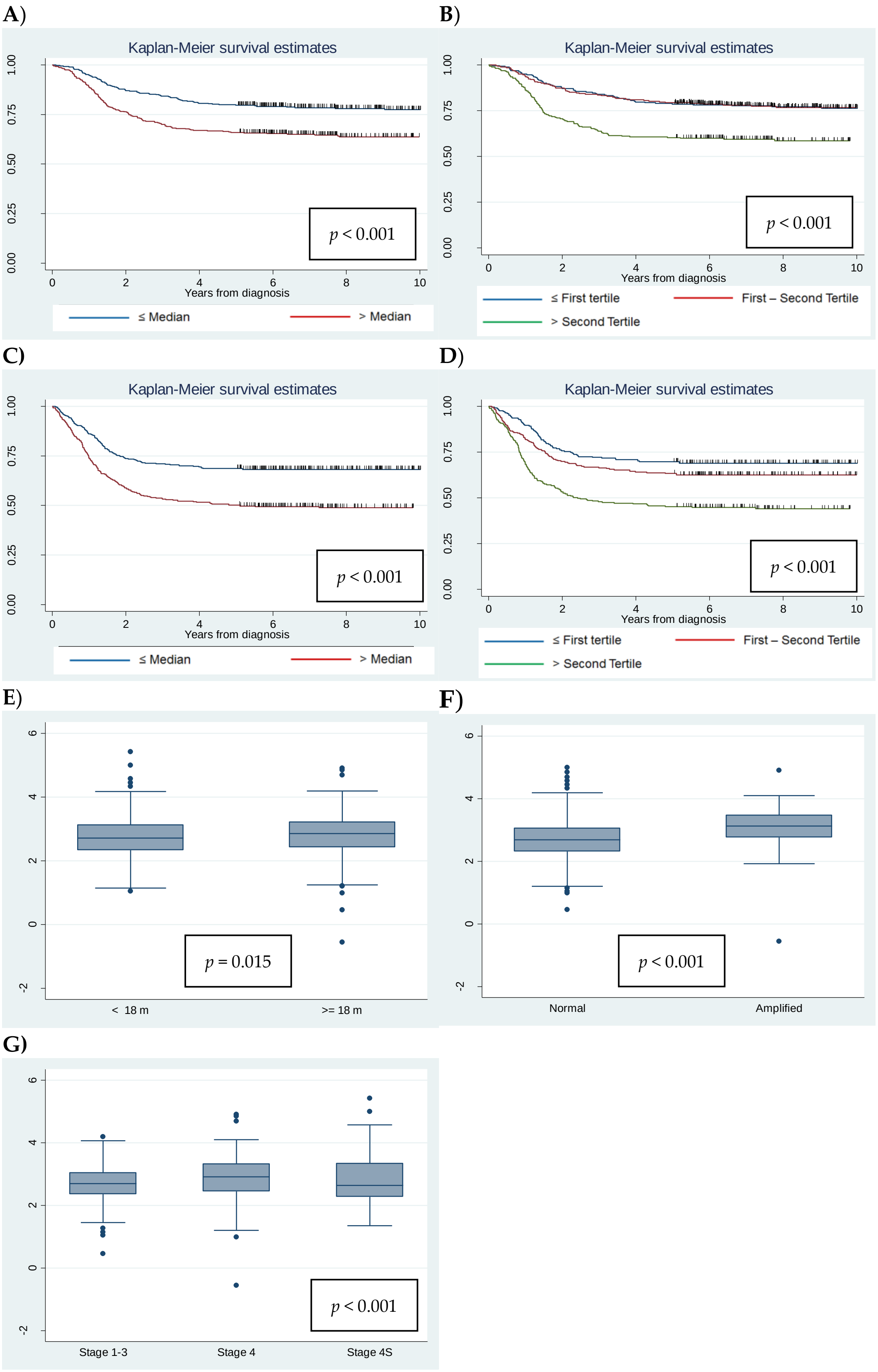


*PANK1* gene expression and its association with survival and clinical characteristics in a cohort of 786 NB patients in the Cangelosi et al. *in silico* dataset. (A) Overall survival, cut-off based on the median expression value; (B) Overall survival, cut-off based on tertile expression values; (C) Event free survival, cut-off based on the median expression value; (D) Event free survival, cut-off based on tertile expression values; E) Age at diagnosis; (F) *MYCN* status; (G) INSS stage.

**Supplementary Figure S70**

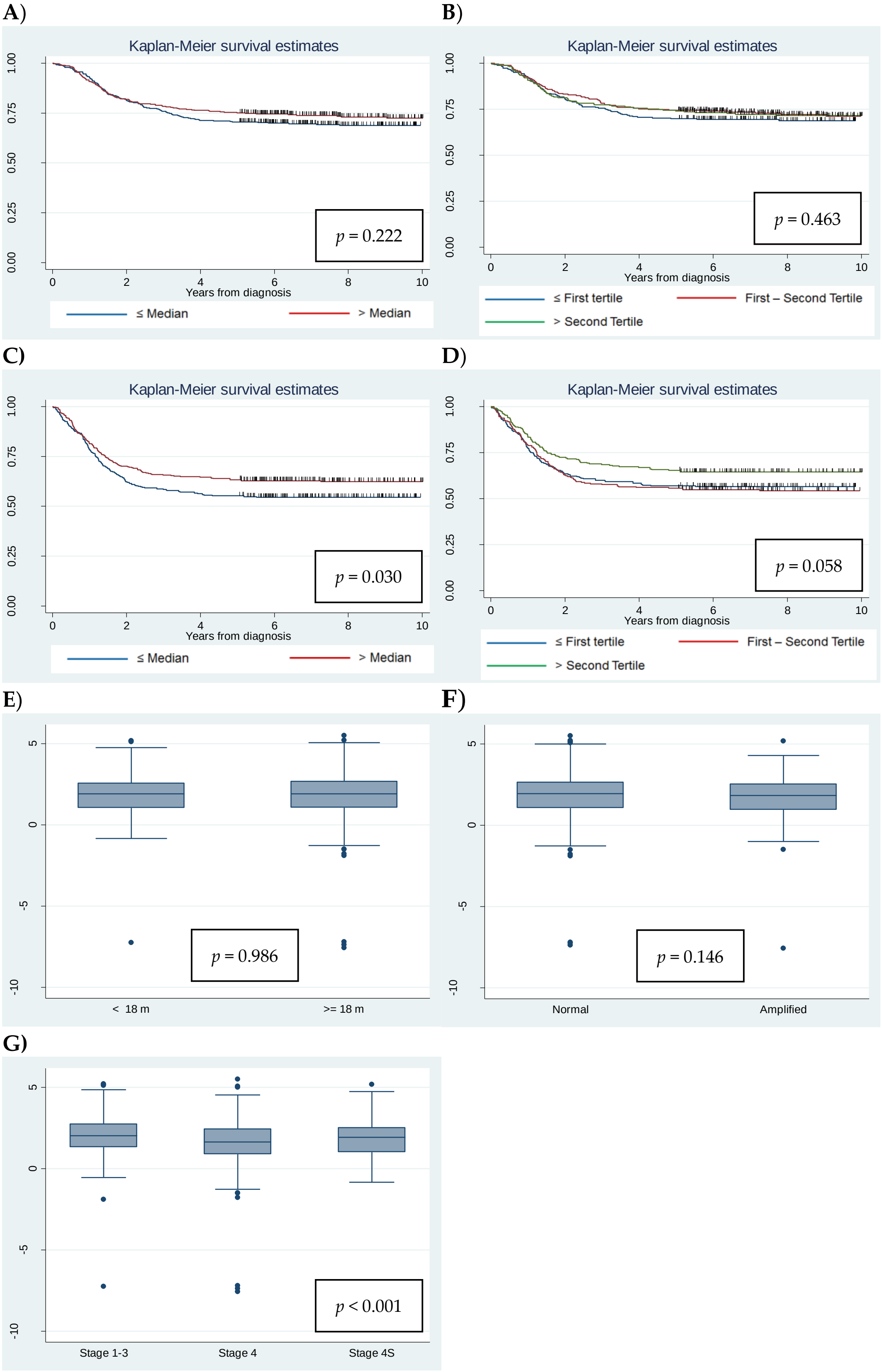


*PCDH21* (alias *CDHR1*) gene expression and its association with survival and clinical characteristics in a cohort of 786 NB patients in the Cangelosi et al. *in silico* dataset. (A) Overall survival, cut-off based on the median expression value; (B) Overall survival, cut-off based on tertile expression values; (C) Event free survival, cut-off based on the median expression value; (D) Event free survival, cut-off based on tertile expression values; E) Age at diagnosis; (F) *MYCN* status; (G) INSS stage.

## Supplementary Figure S71

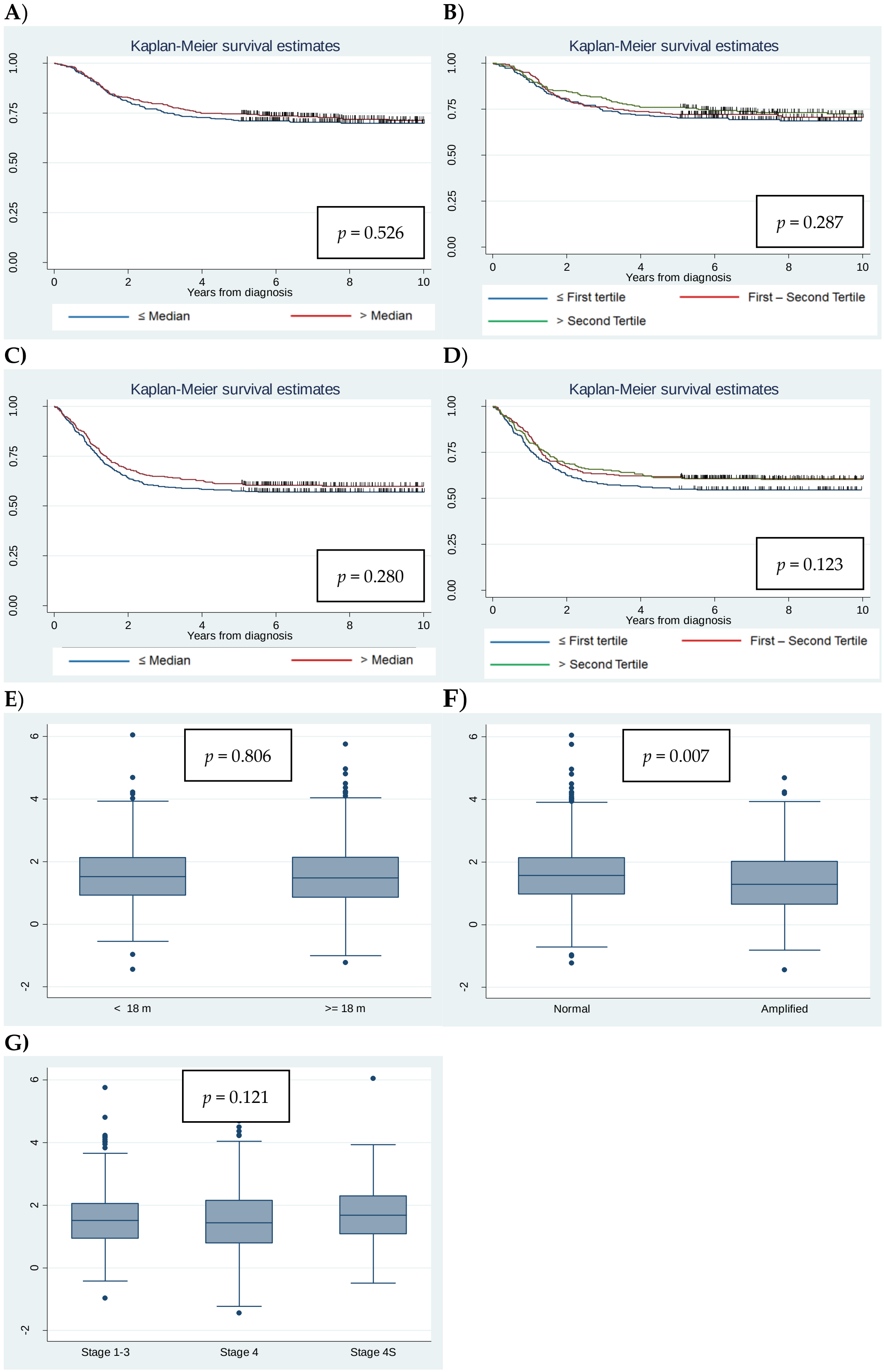


*PLAC9* gene expression and its association with survival and clinical characteristics in a cohort of 786 NB patients in the Cangelosi et al. *in silico* dataset. (A) Overall survival, cut-off based on the median expression value; (B) Overall survival, cut-off based on tertile expression values; (C) Event free survival, cut-off based on the median expression value; (D) Event free survival, cut-off based on tertile expression values; E) Age at diagnosis; (F) *MYCN* status; (G) INSS stage.

## Supplementary Figure S72

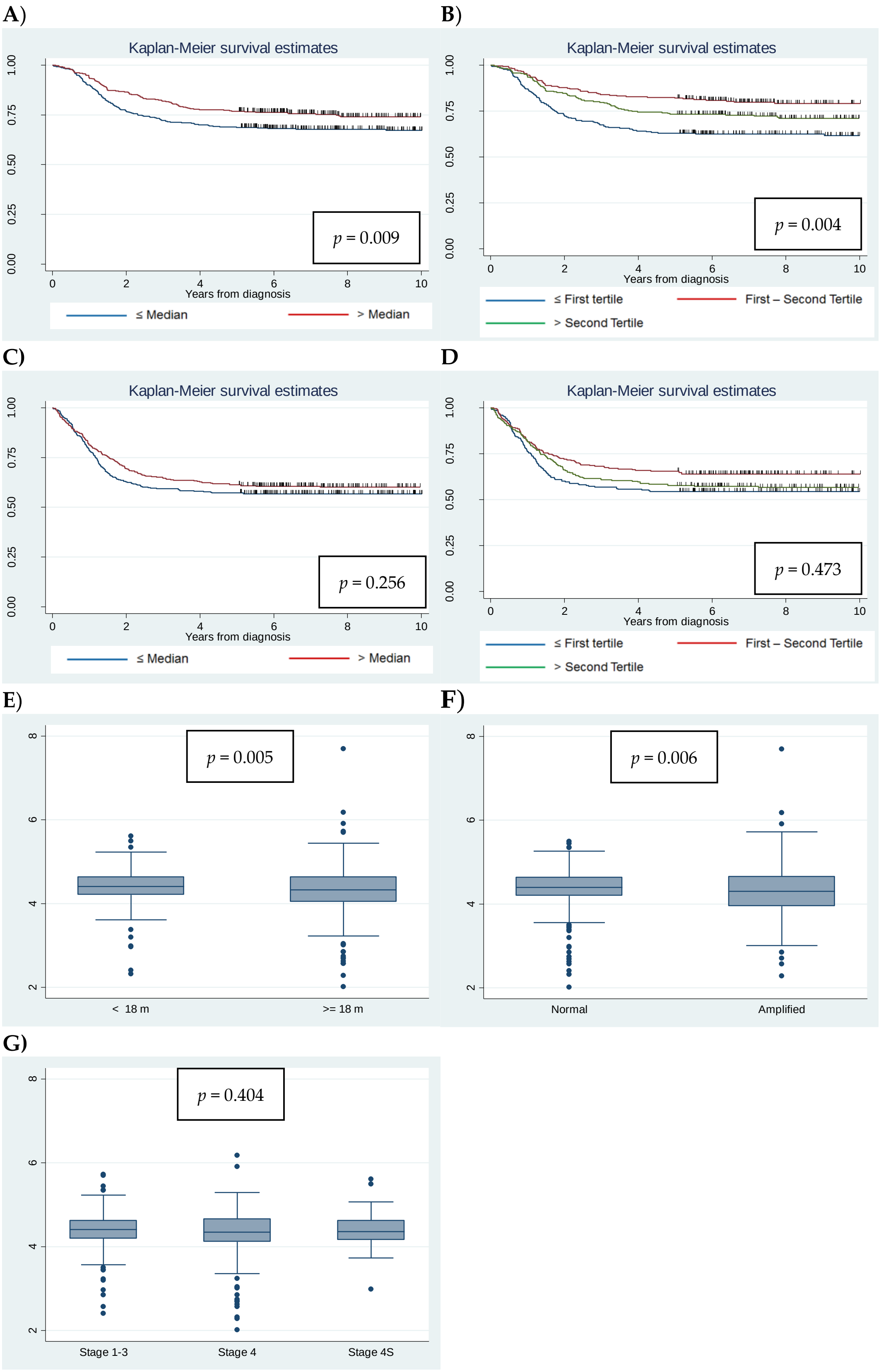


*PTEN* gene expression and its association with survival and clinical characteristics in a cohort of 786 NB patients in the Cangelosi et al. *in silico* dataset. (A) Overall survival, cut-off based on the median expression value; (B) Overall survival, cut-off based on tertile expression values; (C) Event free survival, cut-off based on the median expression value; (D) Event free survival, cut-off based on tertile expression values; E) Age at diagnosis; (F) *MYCN* status; (G) INSS stage.

## Supplementary Figure S73

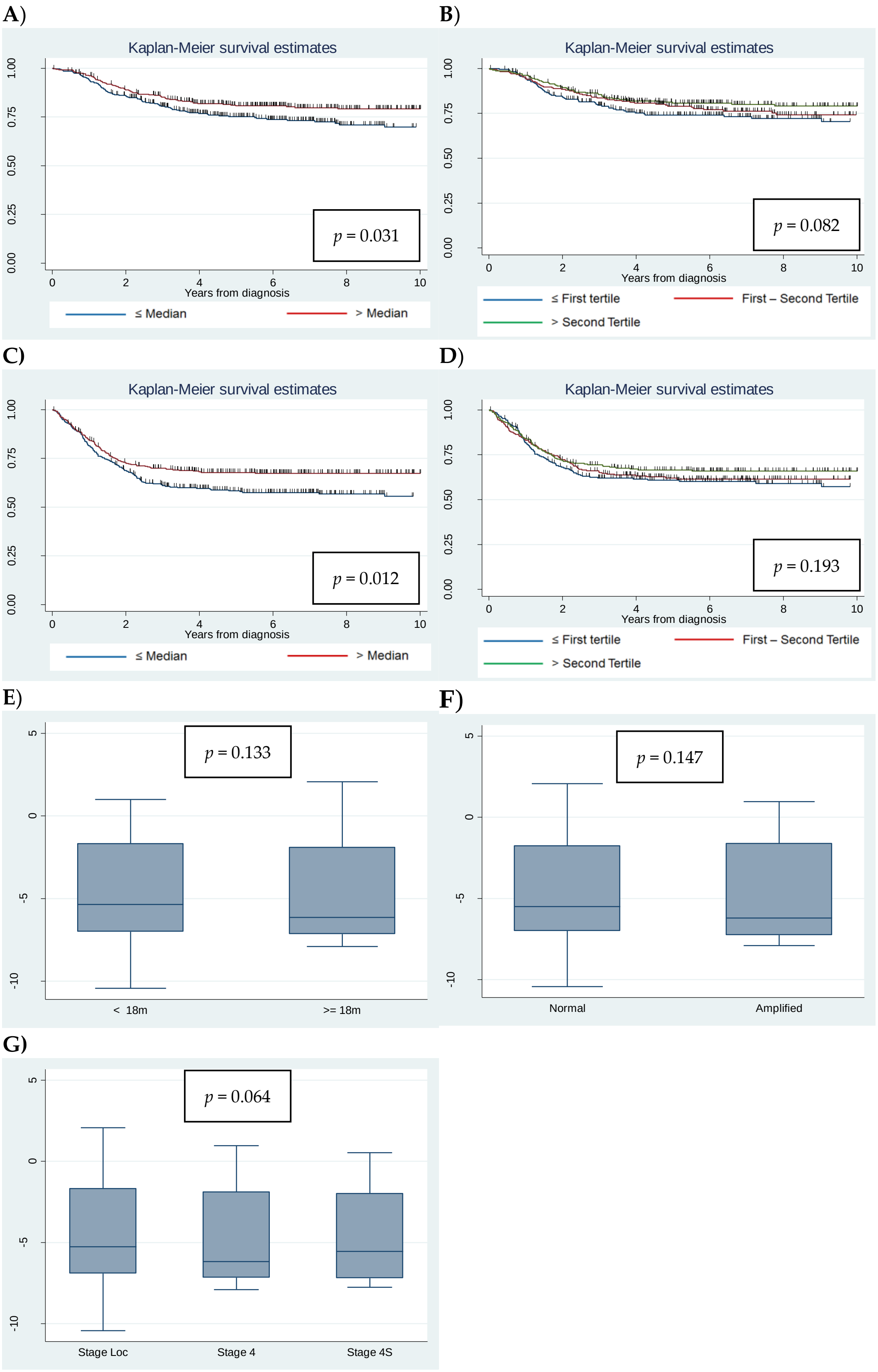


*RGR* gene expression and its association with survival and clinical characteristics in a cohort of 709 NB patients in the E-MTAB-1781 *in silico* dataset. (A) Overall survival, cut-off based on the median expression value; (B) Overall survival, cut-off based on tertile expression values; (C) Event free survival, cut-off based on the median expression value; (D) Event free survival, cut-off based on tertile expression values; E) Age at diagnosis; (F) *MYCN* status; (G) INSS stage.

**Supplementary Figure S74**

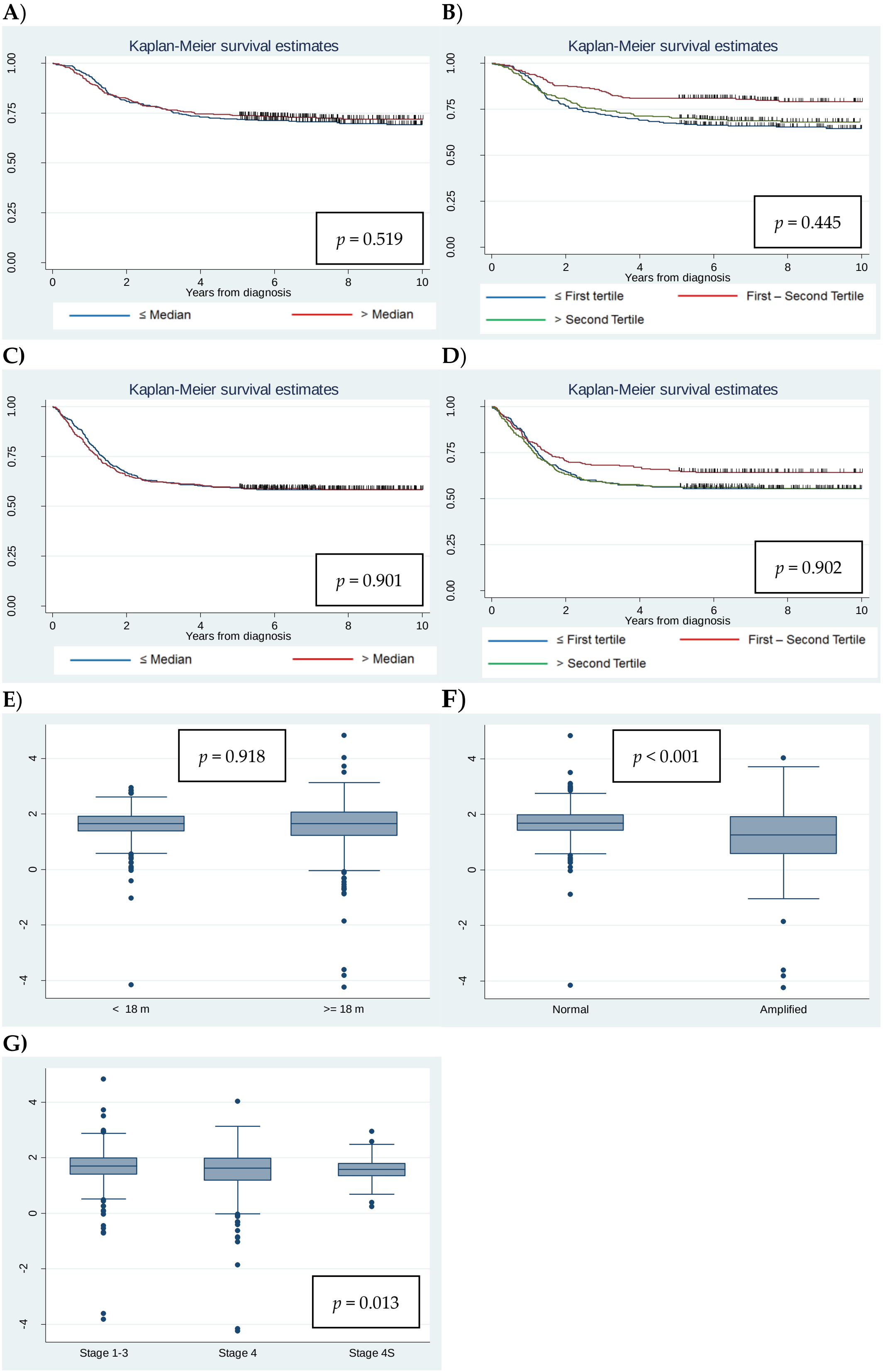


*RNLS* gene expression and its association with survival and clinical characteristics in a cohort of 786 NB patients in the Cangelosi et al. *in silico* dataset. (A) Overall survival, cut-off based on the median expression value; (B) Overall survival, cut-off based on tertile expression values; (C) Event free survival, cut-off based on the median expression value; (D) Event free survival, cut-off based on tertile expression values; E) Age at diagnosis; (F) *MYCN* status; (G) INSS stage.

**Supplementary Figure S75**

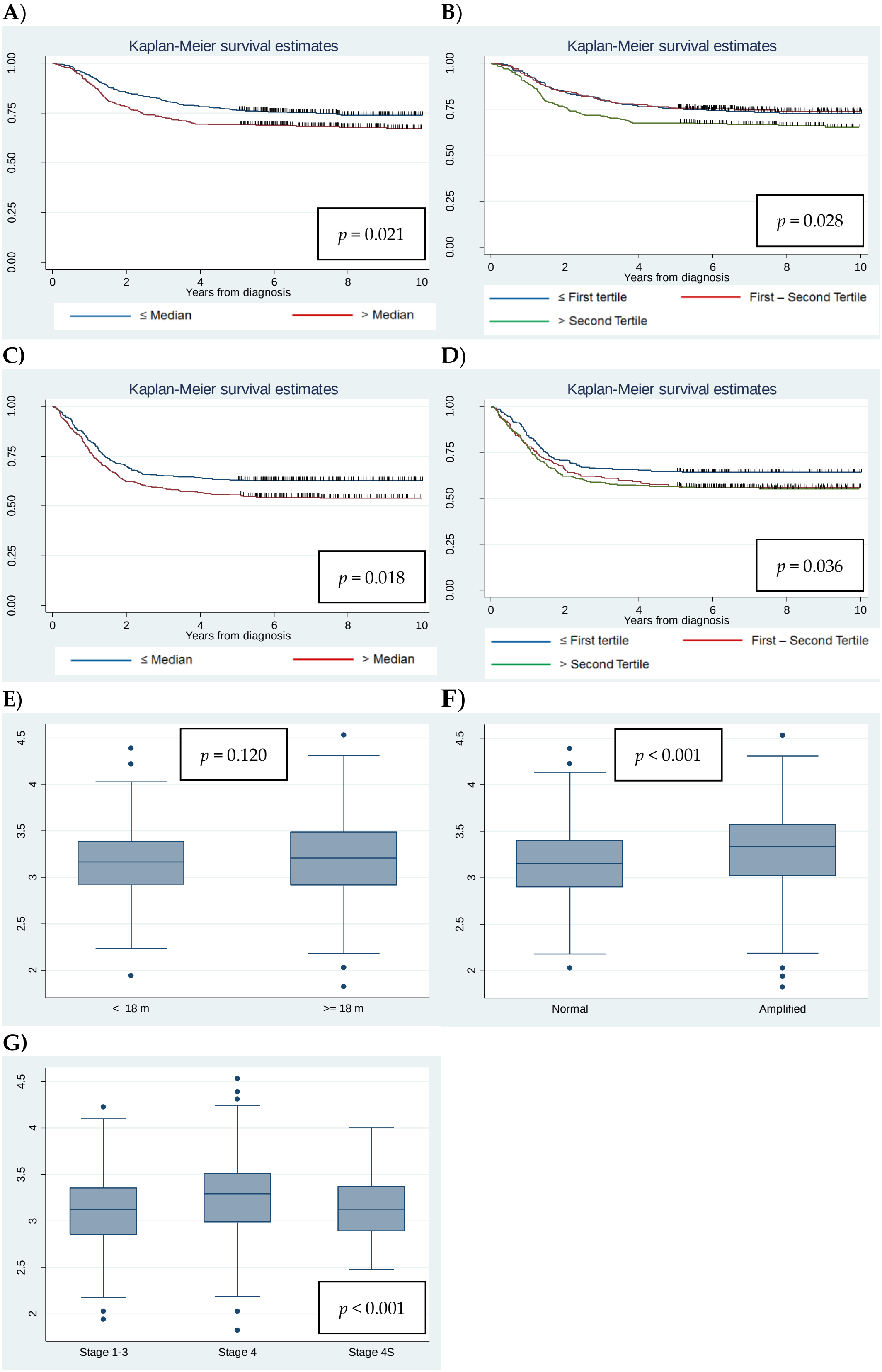


*RPP30* gene expression and its association with survival and clinical characteristics in a cohort of 786 NB patients in the Cangelosi et al. *in silico* dataset. (A) Overall survival, cut-off based on the median expression value; (B) Overall survival, cut-off based on tertile expression values; (C) Event free survival, cut-off based on the median expression value; (D) Event free survival, cut-off based on tertile expression values; E) Age at diagnosis; (F) *MYCN* status; (G) INSS stage.

**Supplementary Figure S76**

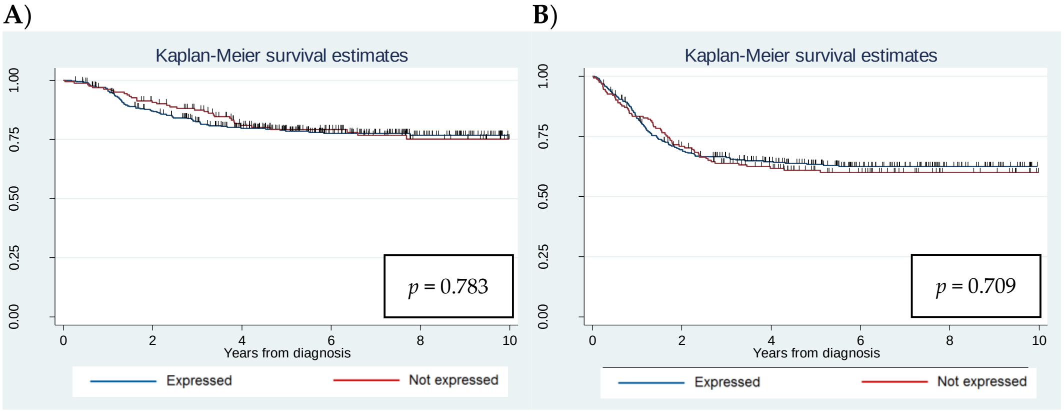


*SFTPA1* gene expression and its association with survival and clinical characteristics in a cohort of 498 NB patients in the SEQC-498 *in silico* dataset. (A) Overall survival, gene expression yes/not; (B) Event Free Survival, gene expression yes/not.

**Supplementary Figure S77**

A)

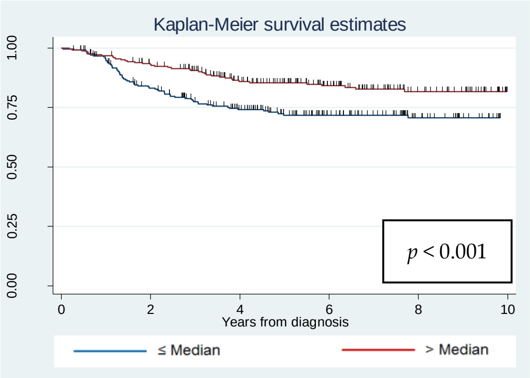


B)

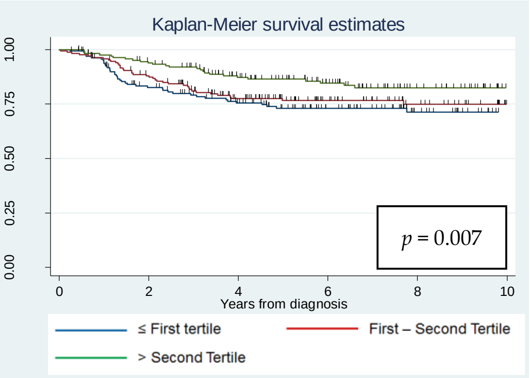


C)

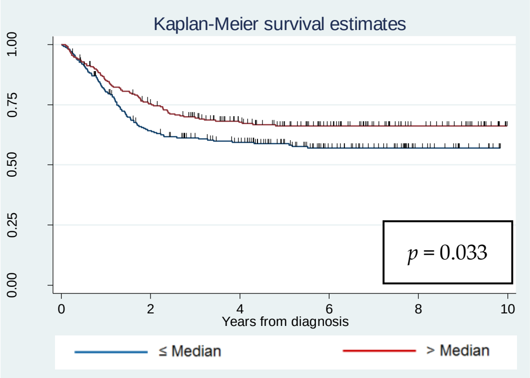


D)

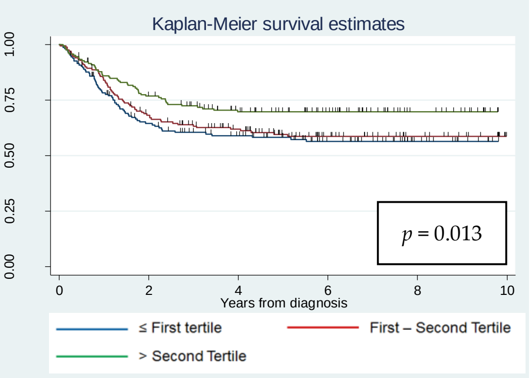


E)

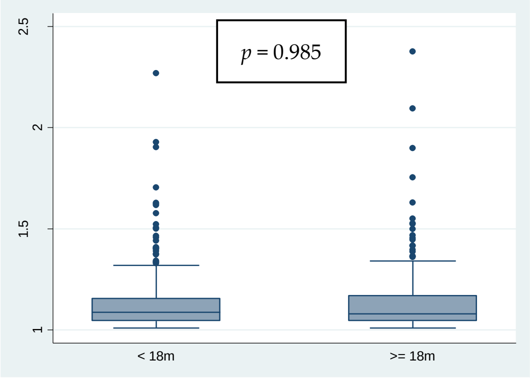


F)

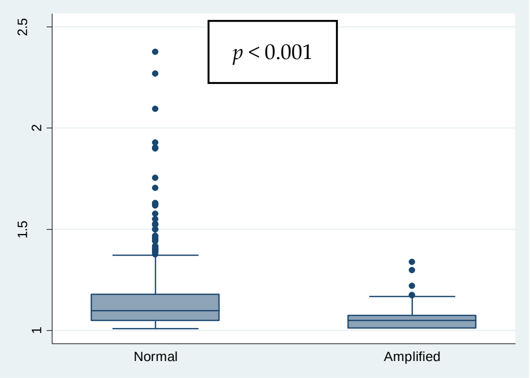


G)

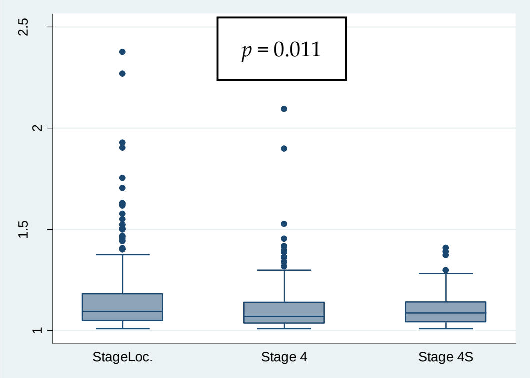


*SFTPA2* gene expression and its association with survival and clinical characteristics in a cohort of 498 NB patients in the SEQC-498 *in silico* dataset. (A) Overall survival, cut-off based on the median expression value; (B) Overall survival, cut-off based on tertile expression values; (C) Event free survival, cut-off based on the median expression value; (D) Event free survival, cut-off based on tertile expression values; E) Age at diagnosis; (F) *MYCN* status; (G) INSS stage.

**Supplementary Figure S78**

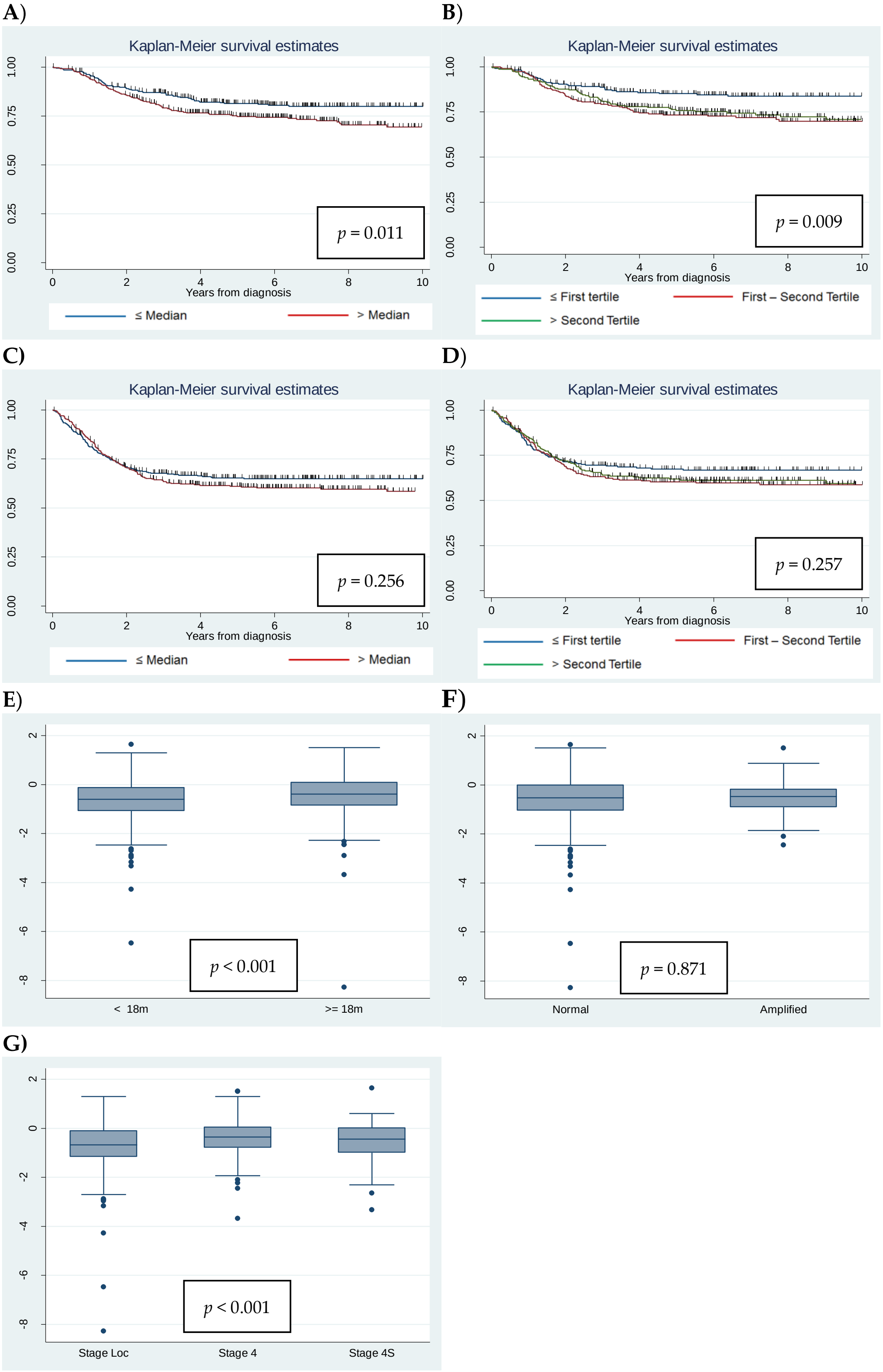


*SFTPD* gene expression and its association with survival and clinical characteristics in a cohort of 709 NB patients in the E-MTAB-1781 *in silico* dataset. (A) Overall survival, cut-off based on the median expression value; (B) Overall survival, cut-off based on tertile expression values; (C) Event free survival, cut-off based on the median expression value; (D) Event free survival, cut-off based on tertile expression values; E) Age at diagnosis; (F) *MYCN* status; (G) INSS stage.

**Supplementary Figure S79**

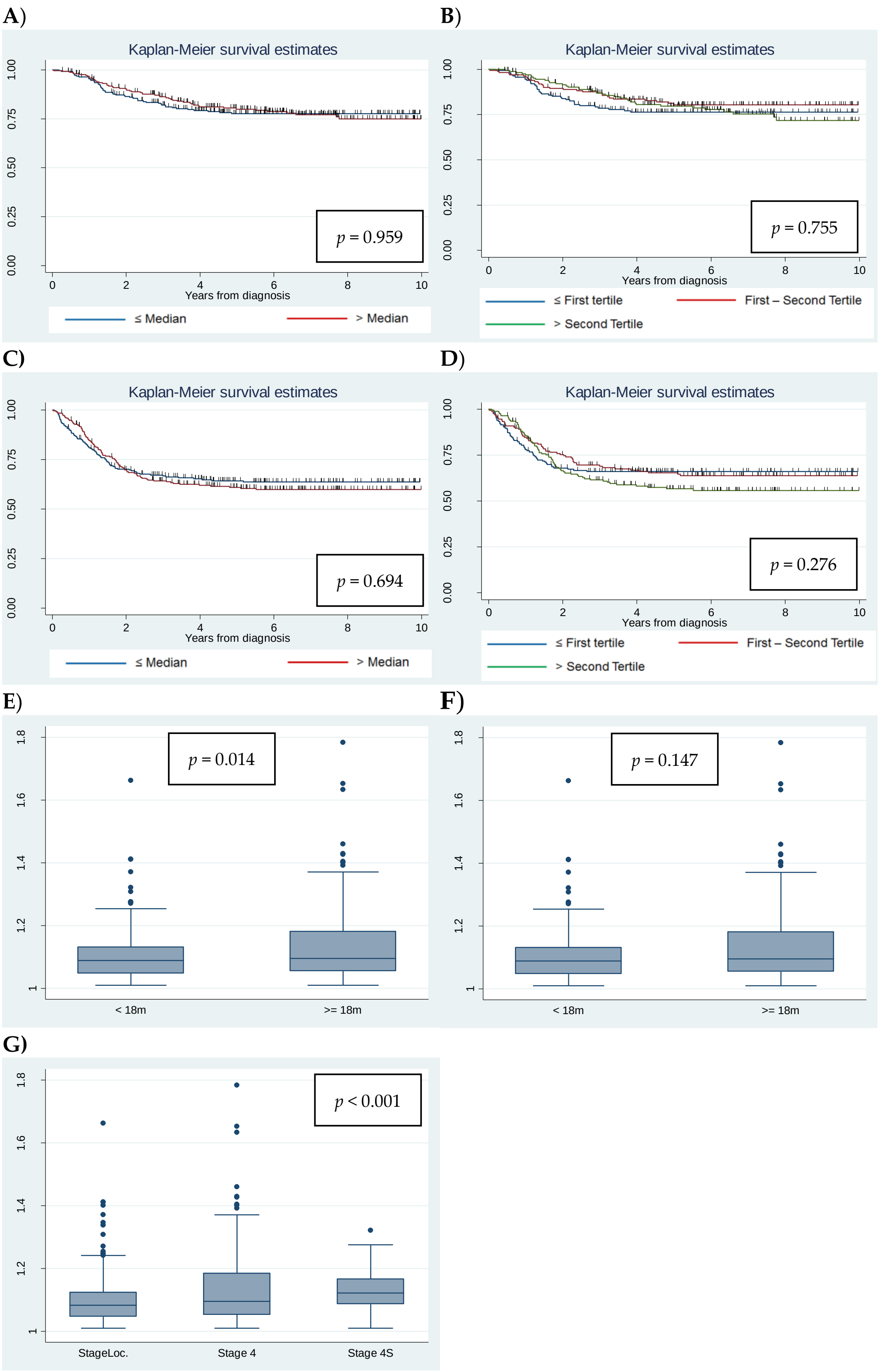


*SH2D4B* gene expression and its association with survival and clinical characteristics in a cohort of 498 NB patients in the SEQC-498 *in silico* dataset. (A) Overall survival, cut-off based on the median expression value; (B) Overall survival, cut-off based on tertile expression values; (C) Event free survival, cut-off based on the median expression value; (D) Event free survival, cut-off based on tertile expression values; E) Age at diagnosis; (F) *MYCN* status; (G) INSS stage.

**Supplementary Figure S80**

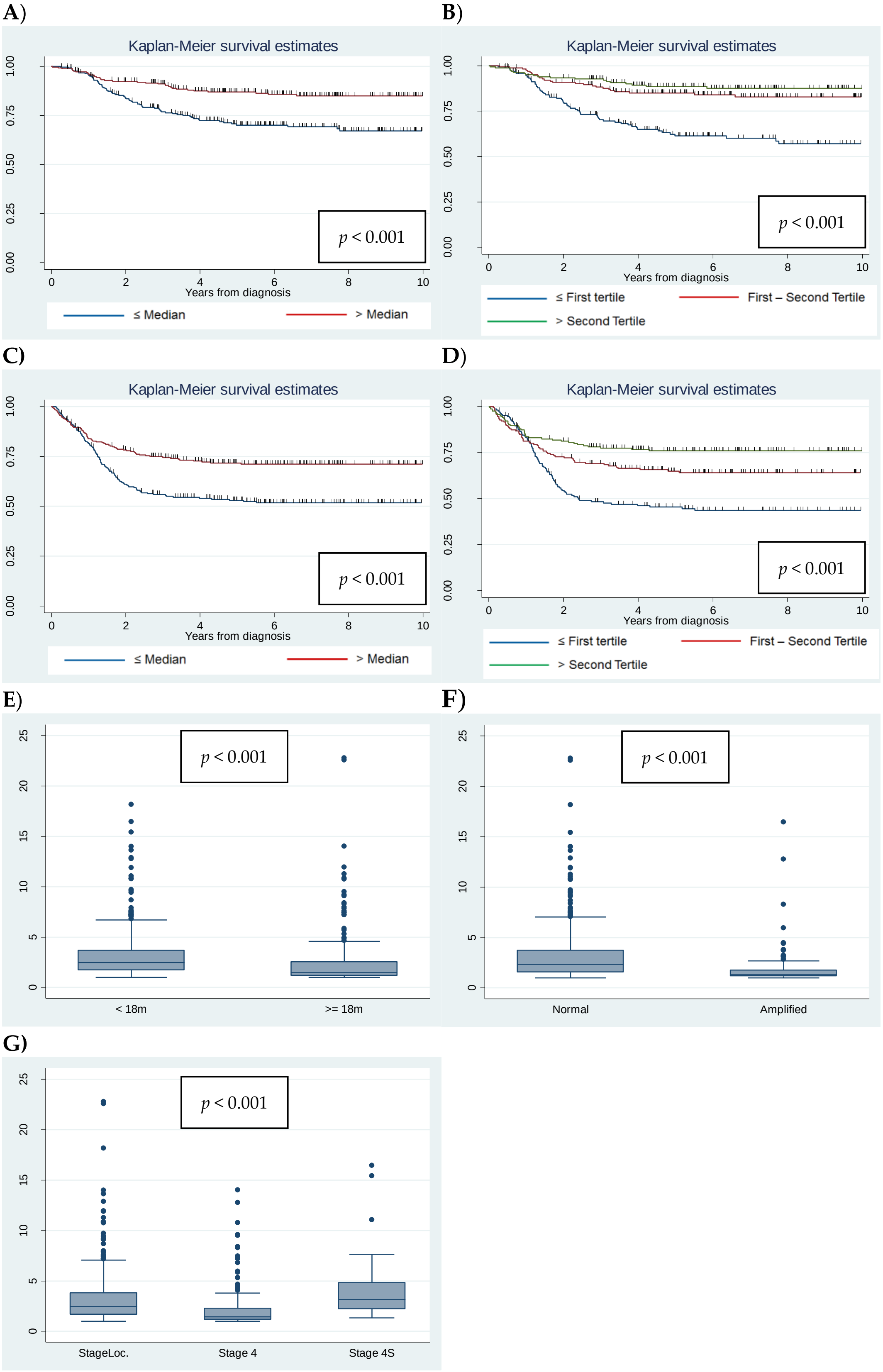


*SLC16A12* gene expression and its association with survival and clinical characteristics in a cohort of 498 NB patients in the SEQC-498 *in silico* dataset. (A) Overall survival, cut-off based on the median expression value; (B) Overall survival, cut-off based on tertile expression values; (C) Event free survival, cut-off based on the median expression value; (D) Event free survival, cut-off based on tertile expression values; E) Age at diagnosis; (F) *MYCN* status; (G) INSS stage.

**Supplementary Figure S81**

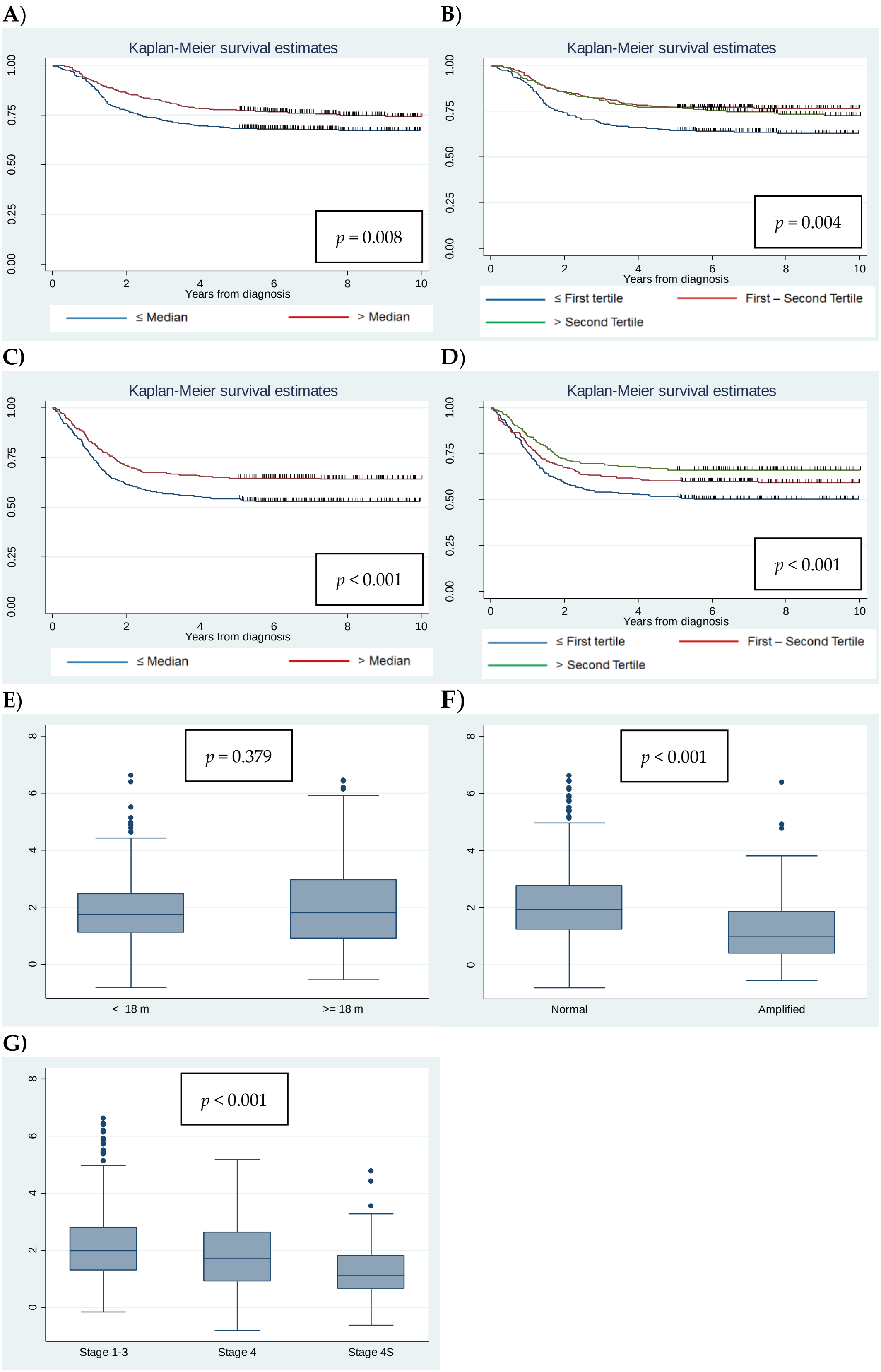


*SNCG* gene expression and its association with survival and clinical characteristics in a cohort of 786 NB patients in the Cangelosi et al. *in silico* dataset. (A) Overall survival, cut-off based on the median expression value; (B) Overall survival, cut-off based on tertile expression values; (C) Event free survival, cut-off based on the median expression value; (D) Event free survival, cut-off based on tertile expression values; E) Age at diagnosis; (F) *MYCN* status; (G) INSS stage.

**Supplementary Figure S82**

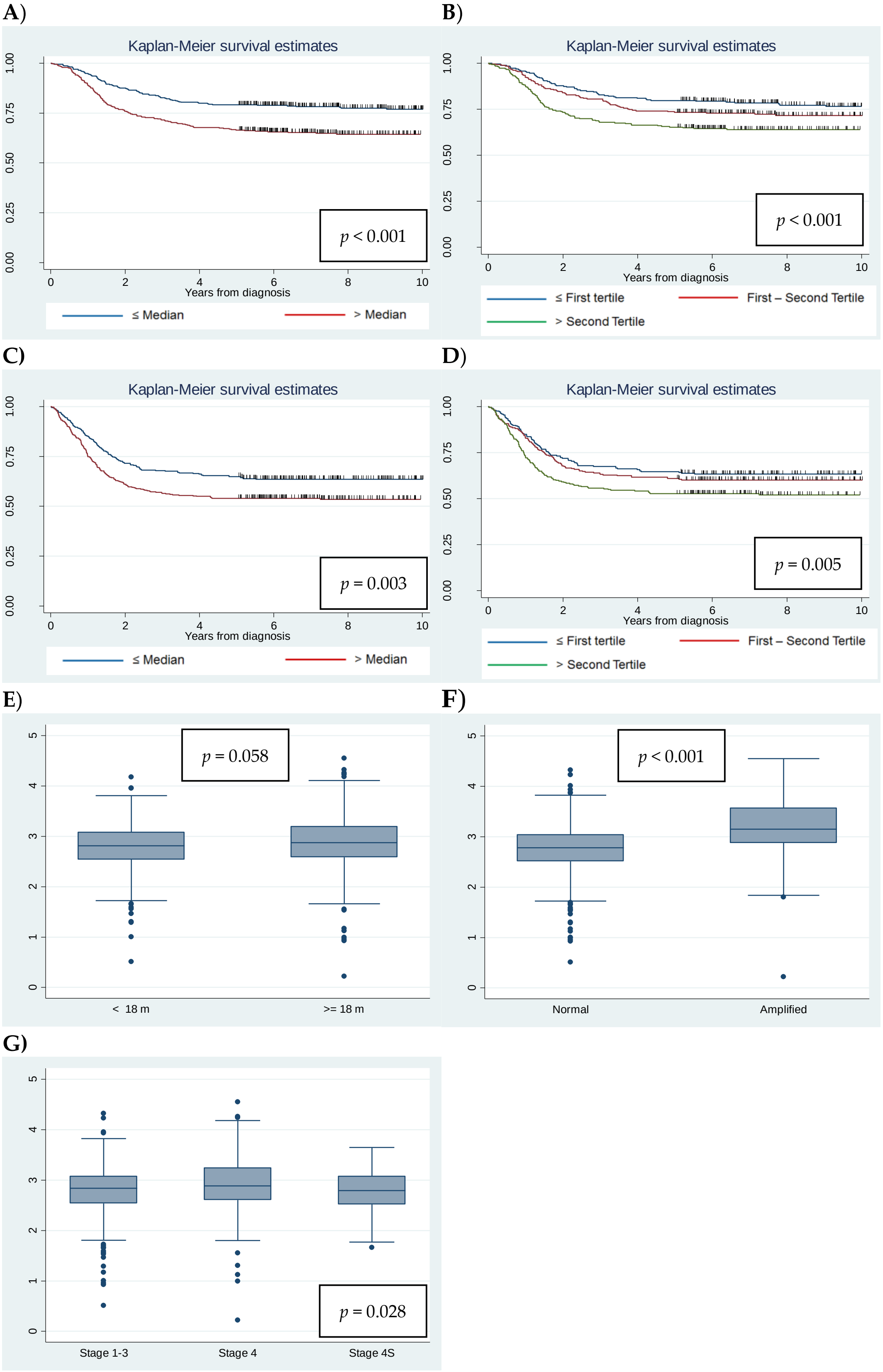


*STAMBPL1* gene expression and its association with survival and clinical characteristics in a cohort of 786 NB patients in the Cangelosi et al. *in silico* dataset. (A) Overall survival, cut-off based on the median expression value; (B) Overall survival, cut-off based on tertile expression values; (C) Event free survival, cut-off based on the median expression value; (D) Event free survival, cut-off based on tertile expression values; E) Age at diagnosis; (F) *MYCN* status; (G) INSS stage.

## Supplementary Figure S83

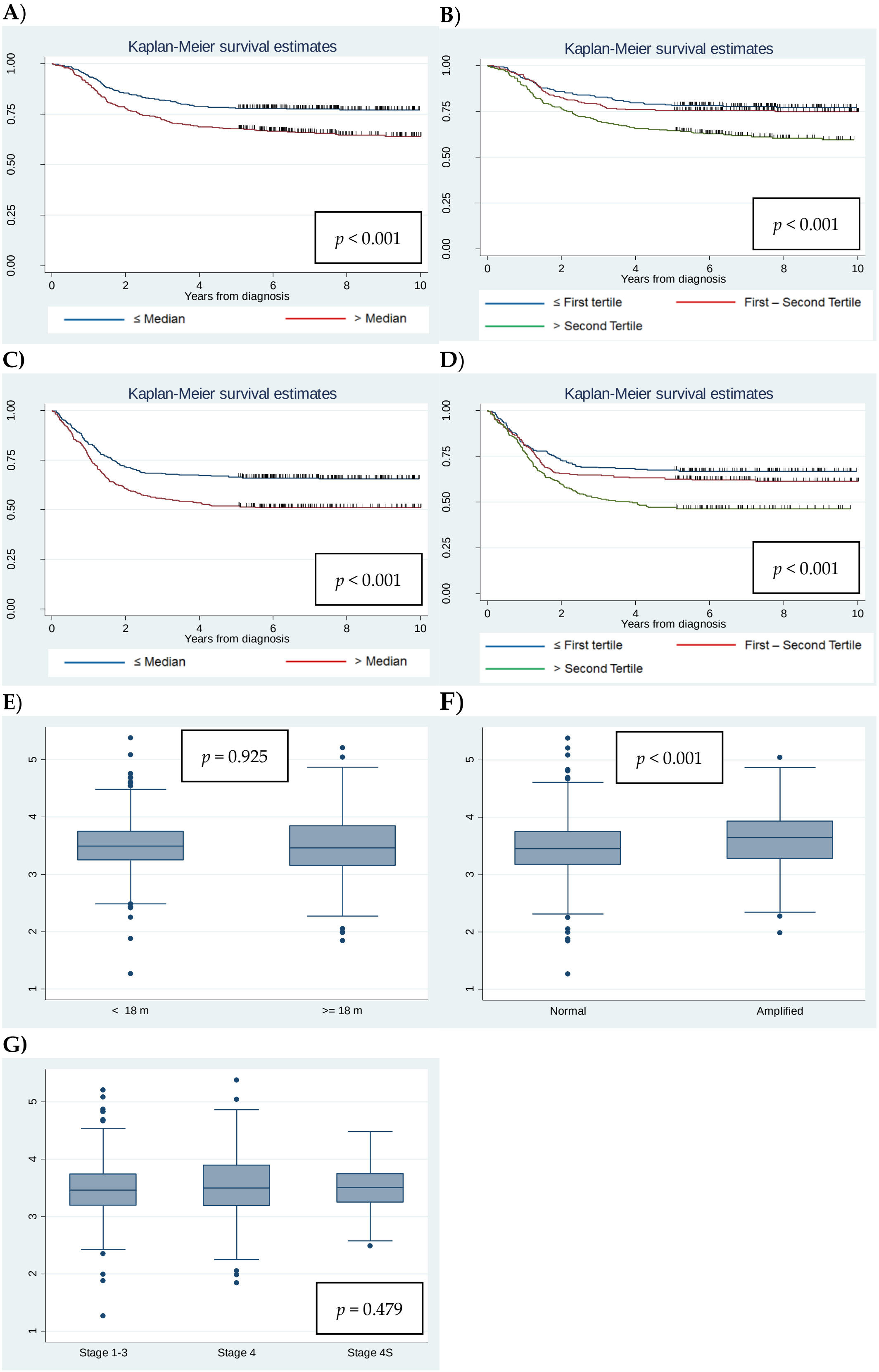


*TSPAN14* gene expression and its association with survival and clinical characteristics in a cohort of 786 NB patients in the Cangelosi et al. *in silico* dataset. (A) Overall survival, cut-off based on the median expression value; (B) Overall survival, cut-off based on tertile expression values; (C) Event free survival, cut-off based on the median expression value; (D) Event free survival, cut-off based on tertile expression values; E) Age at diagnosis; (F) *MYCN* status; (G) INSS stage.

**Supplementary Figure S84**

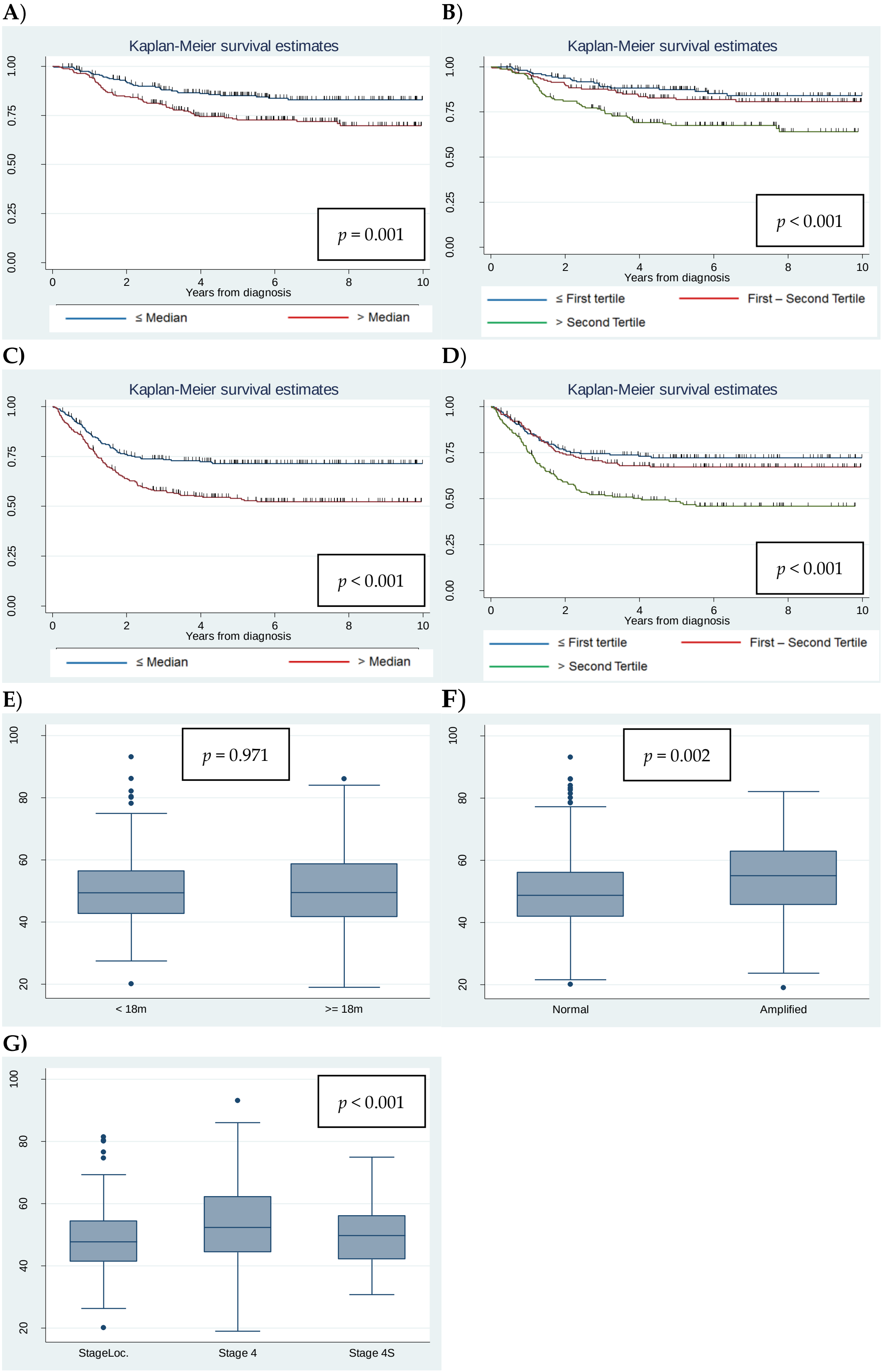


*WAPAL* gene expression and its association with survival and clinical characteristics in a cohort of 498 NB patients in the SEQC-498 *in silico* dataset. (A) Overall survival, cut-off based on the median expression value; (B) Overall survival, cut-off based on tertile expression values; (C) Event free survival, cut-off based on the median expression value; (D) Event free survival, cut-off based on tertile expression values; E) Age at diagnosis; (F) *MYCN* status; (G) INSS stage.

## Supplementary Tables

**Supplementary Table S1.** Demographic and clinical characteristics at diagnosis of the cohort of 260 neuroblastoma patients included in the analysis.

| Patient characteristics | N | % |
|---|---|---|
| Age (months) | | |
| 0-17 | 98 | 37.7 |
| 18-59 | 124 | 47.7 |
| ≥ 60 | 38 | 14.6 |
| Gender | | |
| Male | 138 | 53.1 |
| Female | 122 | 46.9 |
| INSS Stage* | | |
| Stage 1-2 | 61 | 23.6 |
| Stage 3 | 47 | 18.2 |
| Stage 4 | 132 | 51.2 |
| Stage 4S | 18 | 7.0 |
| *MYCN* status | | |
| Non-amplified | 181 | 69.6 |
| Amplified | 79 | 30.4 |
| Chromosome 10q loss | | |
| No | 234 | 90.0 |
| Yes | 26 | 10.0 |

* Two missing values

**Supplementary Table S2**. Association between chromosome 10q loss and other chromosome losses in a cohort of 260 neuroblastoma patients with segmental chromosome alterations at diagnosis.

| | 10q loss | | | | |
|---|---|---|---|---|---|
| | No (n = 234) | | Yes (n = 26) | | |
| Chromosome aberration | N | % | N | % | *p* |
| 1p loss | 103 | 44.0 | 12 | 46.2 | 0.838 |
| 1q loss | 9 | 3.9 | 1 | 3.9 | 0.999 |
| 2p loss | 7 | 3.0 | 0 | 0.0 | 0.999 |
| 2q loss | 10 | 4.3 | 1 | 3.9 | 0.999 |
| 3p loss | 45 | 19.2 | 7 | 26.9 | 0.436 |
| 3q loss | 2 | 0.9 | 1 | 3.9 | 0.272 |
| 4p loss | 34 | 14.5 | 6 | 23.1 | 0.255 |
| 4q loss | 6 | 2.6 | 3 | 11.5 | 0.050 |
| 5p loss | 13 | 5.6 | 2 | 7.7 | 0.652 |
| 5q loss | 9 | 3.9 | 1 | 3.9 | 0.999 |
| 6p loss | 3 | 1.3 | 1 | 3.9 | 0.346 |
| 6q loss | 21 | 9.0 | 3 | 11.5 | 0.718 |
| 7p loss | 1 | 0.4 | 0 | 0.0 | 0.999 |
| 7q loss | 2 | 0.9 | 1 | 3.9 | 0.272 |
| 8p loss | 5 | 2.1 | 4 | 15.4 | 0.007 |
| 8q loss | 7 | 3.0 | 1 | 3.9 | 0.575 |
| 9p loss | 16 | 6.8 | 3 | 11.5 | 0.417 |
| 9q loss | 13 | 5.6 | 3 | 11.5 | 0.206 |
| 10p loss | 6 | 2.6 | 2 | 7.7 | 0.185 |
| 10q loss | - | - | - | - | - |
| 11p loss | 6 | 2.6 | 0 | 0.0 | 0.999 |
| 11q loss | 86 | 36.8 | 11 | 42.3 | 0.670 |
| 12p loss | 1 | 0.43 | 0 | 0.0 | 0.999 |
| 12q loss | 3 | 1.3 | 0 | 0.0 | 0.999 |
| 13p loss | 0 | 0.0 | 0 | 0.0 | - |
| 13q loss | 2 | 0.9 | 1 | 3.9 | 0.272 |
| 14p loss | 0 | 0.0 | 0 | 0.0 | - |
| 14q loss | 21 | 9.0 | 4 | 15.4 | 0.292 |
| 15p loss | 0 | 0.0 | 0 | 0.0 | - |
| 15q loss | 14 | 6.0 | 1 | 3.9 | 0.999 |
| 16p loss | 8 | 3.4 | 0 | 0.0 | 0.999 |
| 16q loss | 10 | 4.3 | 2 | 7.7 | 0.342 |
| 17p loss | 19 | 8.1 | 1 | 3.9 | 0.703 |
| 17q loss | 4 | 1.7 | 0 | 0.0 | 0.999 |
| 18p loss | 2 | 0.9 | 0 | 0.0 | 0.999 |
| 18q loss | 2 | 0.9 | 0 | 0.0 | 0.999 |
| 19p loss | 36 | 15.4 | 4 | 15.4 | 0.999 |
| 19q loss | 10 | 4.3 | 5 | 19.2 | **0.010** |
| 20p loss | 3 | 1.3 | 1 | 3.9 | 0.346 |
| 20q loss | 0 | 0.0 | 0 | 0.0 | - |
| 21p loss | 0 | 0.0 | 0 | 0.0 | - |
| 21q loss | 5 | 2.1 | 3 | 11.5 | 0.036 |
| 22p loss | 0 | 0.0 | 0 | 0.0 | - |
| 22q loss | 13 | 5.6 | 2 | 7.7 | 0.652 |
| Yp loss | 9 | 3.9 | 1 | 3.9 | 0.999 |
| Yq loss | 7 | 3.0 | 1 | 3.9 | 0.575 |
| Xp loss | 2 | 0.9 | 0 | 0.0 | 0.999 |
| Xq loss | 1 | 0.43 | 0 | 0.0 | 0.999 |

**Supplementary Table S3.** Association between chromosome 10q loss and chromosomes gains in a cohort of 260 neuroblastoma patients with segmental chromosome alterations at diagnosis.

| | 10q loss | | | | |
|---|---|---|---|---|---|
| | No (n=234) | | Yes (n=26) | | |
| Chromosome aberration | N | % | N | % | *p* |
| 1p gain | 12 | 5.1 | 2 | 7.7 | 0.638 |
| 1q gain | 41 | 17.5 | 9 | 34.6 | 0.036 |
| 2p gain | 108 | 46.2 | 12 | 46.2 | 0.999 |
| 2q gain | 10 | 4.3 | 3 | 11.5 | 0.129 |
| 3p gain | 3 | 1.3 | 1 | 3.9 | 0.346 |
| 3q gain | 7 | 3.0 | 6 | 23.1 | 0.001 |
| 4p gain | 2 | 0.9 | 1 | 3.9 | 0.272 |
| 4q gain | 12 | 5.1 | 3 | 11.5 | 0.179 |
| 5p gain | 4 | 1.7 | 3 | 11.5 | 0.024 |
| 5q gain | 7 | 3.0 | 1 | 3.9 | 0.575 |
| 6p gain | 13 | 5.6 | 6 | 23.1 | 0.006 |
| 6q gain | 11 | 4.7 | 1 | 3.9 | 0.999 |
| 7p gain | 6 | 2.6 | 1 | 3.9 | 0.526 |
| 7q gain | 21 | 9.0 | 6 | 23.1 | 0.038 |
| 8p gain | 2 | 0.9 | 0 | 0.0 | 0.999 |
| 8q gain | 6 | 2.6 | 5 | 19.2 | 0.002 |
| 9p gain | 5 | 2.1 | 1 | 3.9 | 0.472 |
| 9q gain | 11 | 4.7 | 2 | 7.7 | 0.626 |
| 10p gain | 2 | 0.9 | 0 | 0.0 | 0.999 |
| 10q gain | 1 | 0.4 | 1 | 3.9 | 0.190 |
| 11p gain | 29 | 12.4 | 5 | 19.2 | 0.355 |
| 11q gain | 42 | 18.0 | 5 | 19.2 | 0.793 |
| 12p gain | 4 | 1.7 | 1 | 3.9 | 0.412 |
| 12q gain | 25 | 10.7 | 5 | 19.2 | 0.198 |
| 13p gain | 0 | 0.0 | 0 | 0.0 | - |
| 13q gain | 5 | 2.1 | 1 | 3.9 | 0.472 |
| 14p gain | 0 | 0.0 | 0 | 0.0 | - |
| 14q gain | 6 | 2.6 | 2 | 7.7 | 0.185 |
| 15p gain | 0 | 0.0 | 0 | 0.0 | - |
| 15q gain | 5 | 2.1 | 1 | 3.9 | 0.472 |
| 16p gain | 0 | 0.0 | 0 | 0.0 | - |
| 16q gain | 8 | 3.4 | 0 | 0.0 | 0.999 |
| 17p gain | 2 | 0.9 | 2 | 7.7 | 0.051 |
| 17q gain | 181 | 77.4 | 23 | 88.5 | 0.312 |
| 18p gain | 3 | 1.3 | 1 | 3.9 | 0.346 |
| 18q gain | 11 | 4.7 | 2 | 7.7 | 0.626 |
| 19p gain | 5 | 2.1 | 0 | 0.0 | 0.999 |
| 19q gain | 7 | 3.0 | 0 | 0.0 | 0.999 |
| 20p gain | 3 | 1.3 | 0 | 0.0 | 0.999 |
| 20q gain | 2 | 0.9 | 1 | 3.9 | 0.272 |
| 21p gain | 0 | 0.0 | 0 | 0.0 | - |
| 21q gain | 2 | 0.9 | 1 | 3.9 | 0.272 |
| 22p gain | 0 | 0.0 | 0 | 0.0 | - |
| 22q gain | 12 | 5.1 | 2 | 7.7 | 0.638 |
| Yp gain | 2 | 0.9 | 0 | 0.0 | 0.999 |
| Yq gain | 2 | 0.9 | 1 | 3.9 | 0.272 |
| Xp gain | 2 | 0.9 | 0 | 0.0 | 0.999 |
| Xq gain | 0 | 0.0 | 0 | 0.0 | - |

**Supplementary Table S4** Survival of the cohort of 498 NB patients from the SEQC-498 data set, in relation to *AGAP11* gene expression values, evaluated by the Cox model.

| | | Univariable analysis | | | Multivariable analysis | | |
|---|---|---|---|---|---|---|---|
| **Gene expression** | **N/O** | **HR** | **95%CI** | ***p*** | **HR** | **95%CI** | ***p*** |
| *Overall Survival* | | | | | | | |
| Median | | | | *< 0.001* | | | *0.025* |
| ≤ 4.664 (*reference*) | 249/71 | 1 | - | | 1 | - | |
| > 4.664 | 249/34 | 0.43 | *0.28 – 0.64* | | 0.62 | *0.41 – 0.95* | |
| Tertiles | | | | *< 0.001* | | | *0.003* |
| ≤ 3.970 (*reference*) | 166/58 | 1 | - | | 1 | - | |
| 3.970 – 5.656 | 166/25 | 0.35 | *0.22 – 0.56* | | 0.64 | *0.39 – 1.0* | |
| > 5.656 | 166/22 | 0.31 | *0.19 – 0.51* | | 0.49 | *0.30 – 0.82* | |
| Continuous variable | 498/105 | 0.79 | *0.71 – 0.89* | *< 0.001* | 0.92 | *0.83 – 1.01* | *0.072* |
| *Event Free Survival* | | | | | | | |
| Median | | | | *0.002* | | | *0.146* |
| ≤ 4.664 (*reference*) | 249/58 | 1 | - | | 1 | - | |
| > 4.664 | 249/75 | 0.63 | *0.47 – 0.84* | | 0.80 | *0.59 – 1.1* | |
| Tertiles | | | | *< 0.001* | | | *0.052* |
| ≤ 3.970 (*reference*) | 166/79 | 1 | - | | 1 | - | |
| 3.970 – 5.656 | 166/57 | 0.63 | *0.45 – 0.89* | | 0.92 | *0.64 – 1.3* | |
| > 5.656 | 166/47 | 0.50 | *0.35 – 0.72* | | 0.69 | *0.47 – 1.0* | |
| Continuous variable | 498/183 | 0.87 | *0.80 – 0.94* | *0.001* | 0.93 | *0.87 – 1.0* | *0.064* |

N/O, Number of patients/Outcome (deaths for the overall and events for the event free survival); HR, Hazard Ratio. Multivariable analysis: HRs are adjusted by *MYCN* status, age, and stage at diagnosis.

**Supplementary Table S5.** Survival of the cohort of 283 NB patients from the NRC-283 data set, in relation to *AGAP11* gene expression values, evaluated by the Cox model.

| | | Univariable analysis | | | Multivariable analysis | | |
|---|---|---|---|---|---|---|---|
| **Gene expression** | **N/O** | **HR** | **95%CI** | ***p*** | **HR** | **95%CI** | ***p*** |
| *Overall Survival* | | | | | | | |
| Median | | | | *0.067* | | | *0.145* |
| ≤ 8.10 (*reference*) | 137/44 | 1 | - | | 1 | - | |
| > 8.1 | 139/31 | 0.65 | *0.41 – 1.0* | | 0.70 | *0.43 – 1.1* | |
| Tertiles | | | | *0.029* | | | *0.199* |
| ≤ 6.8 (*reference*) | 88/30 | 1 | - | | 1 | - | |
| 6.8 – 9.8 | 94/27 | 0.84 | *0.50 – 1.4* | | 0.88 | *0.52 – 1.5* | |
| > 9.8 | 94/18 | 0.52 | *0.29 – 0.94* | | 0.66 | *0.36 – 1.2* | |
| Continuous variable | 276/75 | 0.97 | *0.90 – 1.03* | *0.264* | 0.98 | *0.91 – 1.04* | *0.451* |
| *Progression Free Survival* | | | | | | | |
| Median | | | | *0.520* | | | *0.949* |
| ≤ 8.1 (*reference*) | 136/51 | 1 | - | | 1 | - | |
| > 8.1 | 139/46 | 0.88 | *0.59 – 1.3* | | 0.99 | *0.65 – 1.5* | |
| Tertiles | | | | *0.176* | | | *0.764* |
| ≤ 6.8 (*reference*) | 87/35 | 1 | - | | 1 | - | |
| 6.8 – 9.8 | 94/34 | 0.89 | *0.56 – 1.4* | | 0.96 | *0.59 – 1.6* | |
| > 9.8 | 94/28 | 0.71 | *0.43 – 1.2* | | 0.92 | *0.54 – 1.6* | |
| Continuous variable | 275/973 | 0.98 | *0.93 – 1.04* | *0.504* | 0.99 | *0.94 – 1.05* | *0.875* |

N/O, Number of patients/Outcome (deaths for the overall and events for the progression free survival); HR, Hazard Ratio. Multivariable analysis: HRs are adjusted by *MYCN* status, age, and stage at diagnosis.

**Supplementary Table S6.** Survival of the cohort of 786 NB patients from the Cangelosi *et al.* data set, in relation to *IFIT2* gene expression values, evaluated by the Cox model.

| | | Univariable analysis | | | Multivariable analysis | | |
|---|---|---|---|---|---|---|---|
| **Gene expression** | **N/O** | **HR** | **95%CI** | ***p*** | **HR** | **95%CI** | ***p*** |
| *Overall Survival* | | | | | | | |
| Median | | | | *< 0.001* | | | *0.010* |
| ≤ 2.391 (*reference*) | 392/138 | 1 | - | | 1 | - | |
| > 2.391 | 394/91 | 0.59 | *0.45 – 0.77* | | 0.70 | *0.54 – 0.92* | |
| Tertiles | | | | *< 0.001* | | | *0.080* |
| ≤ 2.044 (*reference*) | 262/105 | 1 | - | | 1 | - | |
| 2.044 – 2.732 | 262/50 | 0.51 | *0.37 – 0.70* | | 0.69 | *0.50 – 0.96* | |
| > 2.732 | 262/64 | 0.53 | *0.39 – 0.72* | | 0.77 | *0.56 – 1.1* | |
| Continuous variable | 786/229 | 0.65 | *0.56 – 0.76* | *< 0.001* | 0.87 | *0.75 – 0.99* | *0.041* |
| *Event Free Survival* | | | | | | | |
| Median | | | | *0.010* | | | *0.062* |
| ≤ 2.391 (*reference*) | 378/173 | 1 | - | | 1 | - | |
| > 2.391 | 391/147 | 0.75 | *0.60 – 0.93* | | 0.81 | *0.65 – 1.0* | |
| Tertiles | | | | *0.002* | | | *0.129* |
| ≤ 2.044 (*reference*) | 250/125 | 1 | - | | 1 | - | |
| 2.044 – 2.732 | 259/97 | 0.66 | *0.51 – 0.86* | | 0.79 | *0.61 – 1.0* | |
| > 2.732 | 260/98 | 0.65 | *0.50 – 0.85* | | 0.81 | *0.62 – 1.1* | |
| Continuous variable | 769/320 | 0.76 | *0.67 – 0.87* | *< 0.001* | 0.89 | *0.78 – 1.0* | *0.048* |

N/O, Number of patients/Outcome (deaths for the overall and events for the event free survival); HR, Hazard Ratio. Multivariable analysis: HRs are adjusted by *MYCN* status, age, and stage at diagnosis.

**Supplementary Table S7.** Survival of the cohort of 283 NB patients from the NRC-283 data set, in relation to *IFIT2* gene expression values, evaluated by the Cox model.

| | | Univariable analysis | | | Multivariable analysis | | |
|---|---|---|---|---|---|---|---|
| **Gene expression** | **N/O** | **HR** | **95%CI** | ***p*** | **HR** | **95%CI** | ***p*** |
| *Overall Survival* | | | | | | | |
| Median | | | | *0.040* | | | *0.938* |
| ≤ 34.9 (*reference*) | 139/46 | 1 | - | | 1 | - | |
| > 34.9 | 137/29 | 0.61 | *0.39 – 0.98* | | 0.98 | *0.60 – 1.6* | |
| Tertiles | | | | *0.009* | | | *0.644* |
| ≤ 28.6 (*reference*) | 92/36 | 1 | - | | 1 | - | |
| 28.6 – 43.7 | 92/18 | 0.37 | *0.21 – 0.65* | | 0.50 | *0.28 – 0.90* | |
| > 43.7 | 92/21 | 0.51 | *0.30 – 0.88* | | 0.99 | *0.54 – 1.8* | |
| Continuous variable | 176/75 | 0.997 | *0.989 – 1.01* | *0.464* | 1.00 | *0.995 – 1.01* | *0.663* |
| *Progression Free Survival* | | | | | | | |
| Median | | | | *0.161* | | | *0.947* |
| ≤ 34.9 (*reference*) | 138/55 | 1 | - | | 1 | - | |
| > 34.9 | 137/42 | 0.75 | *0.50 – 1.1* | | 1.0 | *0.67 – 1.5* | |
| Tertiles | | | | *0.020* | | | *0.634* |
| ≤ 28.6 (*reference*) | 91/44 | 1 | - | | 1 | - | |
| 28.6 – 43.7 | 92/23 | 0.39 | *0.23 – 0.64* | | 0.52 | *0.31 – 0.88* | |
| > 43.7 | 92/30 | 0.60 | *0.37 – 0.95* | | 0.94 | *0.57 – 1.6* | |
| Continuous variable | 275/970 | 1.00 | *0.99 – 1.01* | *0.947* | 1.00 | *0.997–1.008* | *0.415* |

N/O, Number of patients/Outcome (deaths for the overall and events for the progression free survival); HR, Hazard Ratio. Multivariable analysis: HRs are adjusted by *MYCN* status, age, and stage at diagnosis.

**Supplementary Table S8.** Survival of the cohort of 786 NB patients from the Cangelosi *et al.* data set, in relation to *PAPSS2* gene expression values, evaluated by the Cox model.

| | | **Univariable analysis** | | | **Multivariable analysis** | | |
|---|---|---|---|---|---|---|---|
| **Gene expression** | **N/O** | **HR** | **95%CI** | ***p*** | **HR** | **95%CI** | ***p*** |
| *Overall Survival* | | | | | | | |
| Median | | | | *< 0.001* | | | *0.177* |
| ≤ 2.852 (*reference*) | 393/148 | 1 | - | | 1 | - | |
| > 2.852 | 393/81 | 0.49 | *0.37 – 0.64* | | 0.83 | *0.63 – 1.1* | |
| Tertiles | | | | *< 0.001* | | | *0.015* |
| ≤ 2.468 (*reference*) | 262/119 | 1 | - | | 1 | - | |
| 2.468 – 3.198 | 262/60 | 0.44 | *0.32 – 0.60* | | 0.79 | *0.58 – 1.1* | |
| > 3.198 | 262/50 | 0.36 | *0.26 – 0.49* | | 0.67 | *0.48 – 0.94* | |
| Continuous variable | 786/229 | 0.57 | *0.49 – 0.66* | *< 0.001* | 0.86 | *0.74 – 0.99* | *0.029* |
| *Event Free Survival* | | | | | | | |
| Median | | | | *0.003* | | | *0.985* |
| ≤ 2.852 (*reference*) | 381/179 | 1 | - | | 1 | - | |
| > 2.852 | 388/141 | 0.72 | *0.57 – 0.89* | | 1.0 | *0.79 – 1.3* | |
| Tertiles | | | | *<0.001* | | | *0.708* |
| ≤ 2.468 (*reference*) | 251/129 | 1 | - | | 1 | - | |
| 2.468 – 3.198 | 259/99 | 0.67 | *0.52 – 0.87* | | 0.97 | *0.74 – 1.3* | |
| > 3.198 | 259/92 | 0.62 | *0.48 – 0.81* | | 0.95 | *0.72 – 1.3* | |
| Continuous variable | 769/320 | 0.74 | *0.65 – 0.84* | *< 0.001* | 0.94 | *0.83 – 1.1* | *0.345* |

N/O, Number of patients/Outcome (deaths for the overall and events for the event free survival); HR, Hazard Ratio. Multivariable analysis: HRs are adjusted by *MYCN* status, age, and stage at diagnosis.

**Supplementary Table S9.** Survival of the cohort of 283 NB patients from the NRC-283 data set, in relation to *PAPSS2* gene expression values, evaluated by the Cox model.

| | | **Univariable analysis** | | | **Multivariable analysis** | | |
|---|---|---|---|---|---|---|---|
| **Gene expression** | **N/O** | **HR** | **95%CI** | ***p*** | **HR** | **95%CI** | ***p*** |
| *Overall Survival* | | | | | | | |
| Median | | | | *0.025* | | | *0.821* |
| ≤ 77.1 (*reference*) | 137/46 | 1 | - | | 1 | - | |
| > 77.1 | 139/29 | 0.59 | *0.37 – 094* | | 0.95 | *0.58 – 1.5* | |
| Tertiles | | | | *0.013* | | | *0.713* |
| ≤ 63.8 (*reference*) | 90/34 | 1 | - | | 1 | - | |
| 63.8 – 103.3 | 94/23 | 0.63 | *0.37 – 1.1* | | 0.90 | *0.52 – 1.5* | |
| > 103.3 | 92/18 | 0.50 | *0.28 – 0.89* | | 0.90 | *0.49 – 1.7* | |
| Continuous variable | 276/75 | 0.997 | *0.993–1.00* | *0.075* | 1.00 | *0.997–1.003* | *0.981* |
| *Progression Free Survival* | | | | | | | |
| Median | | | | *0.009* | | | *0.373* |
| ≤ 77.1 (*reference*) | 136/59 | 1 | - | | 1 | - | |
| > 77.1 | 139/38 | 0.59 | *0.39 – 0.88* | | 0.83 | *0.54 – 1.3* | |
| Tertiles | | | | *0.015* | | | *0.635* |
| ≤ 63.8 (*reference*) | 89/42 | 1 | - | | 1 | - | |
| 63.8 – 103.3 | 94/30 | 0.65 | *0.41 – 1.04* | | 0.92 | *0.57 – 1.5* | |
| > 103.3 | 92/25 | 0.55 | *0.34 – 0.91* | | 0.88 | *0.52 – 1.5* | |
| Continuous variable | 275/97 | 0.997 | *0.994–1.00* | *0.063* | 1.000 | *0.997–1.003* | *0.771* |

N/O, Number of patients/Outcome (deaths for the overall and events for the progression free survival); HR, Hazard Ratio. Multivariable analysis: HRs are adjusted by *MYCN* status, age, and stage at diagnosis.

**Supplementary Table S10.** Survival of the cohort of 786 NB patients from the Cangelosi *et al.* data set, in relation to *PCGF5* gene expression values, evaluated by the Cox model.

| | | Univariable analysis | | | Multivariable analysis | | |
|---|---|---|---|---|---|---|---|
| **Gene expression** | **N/O** | **HR** | **95%CI** | ***p*** | **HR** | **95%CI** | ***p*** |
| *Overall Survival* | | | | | | | |
| Median | | | | *< 0.001* | | | *< 0.001* |
| ≤ 3.008 (*reference*) | 393/161 | 1 | - | | 1 | - | |
| > 3.008 | 393/61 | 0.34 | *0.26 – 0.46* | | 0.56 | *0.42 – 0.76* | |
| Tertiles | | | | *< 0.001* | | | *< 0.001* |
| ≤ 2.719 (*reference*) | 262/126 | 1 | - | | 1 | - | |
| 2.719 – 3.218 | 262/62 | 0.41 | *0.30 – 0.56* | | 0.71 | *0.52 – 0.97* | |
| > 3.218 | 262/41 | 0.25 | *0.17 – 0.35* | | 0.48 | *0.33 – 0.69* | |
| Continuous variable | 786/229 | 0.52 | *0.45 – 0.60* | *< 0.001* | 0.79 | *0.66 – 0.93* | *0.006* |
| *Event Free Survival* | | | | | | | |
| Median | | | | *< 0.001* | | | *< 0.001* |
| ≤ 3.008 (*reference*) | 379/198 | 1 | - | | 1 | - | |
| > 3.008 | 390/122 | 0.49 | *0.39 – 0.62* | | 0.67 | *0.53 – 0.85* | |
| Tertiles | | | | *< 0.001* | | | *0.006* |
| ≤ 2.719 (*reference*) | 249/141 | 1 | - | | 1 | - | |
| 2.719 – 3.218 | 259/98 | 0.57 | *0.44 – 0.74* | | 0.82 | *0.63 – 1.1* | |
| > 3.218 | 261/81 | 0.43 | *0.33 – 0.57* | | 0.67 | *0.50 – 0.89* | |
| Continuous variable | 769/320 | 0.60 | *0.52 – 0.70* | *< 0.001* | 0.85 | *0.72 – 1.0* | *0.047* |

N/O, Number of patients/Outcome (deaths for the overall and events for the event free survival); HR, Hazard Ratio. Multivariable analysis: HRs are adjusted by *MYCN* status, age, and stage at diagnosis.

**Supplementary Table S11.** Survival of the cohort of 283 NB patients from the NRC-283 data set, in relation to *PCGF5* gene expression values, evaluated by the Cox model.

| | | Univariable analysis | | | Multivariable analysis | | |
|---|---|---|---|---|---|---|---|
| **Gene expression** | **N/O** | **HR** | **95%CI** | ***p*** | **HR** | **95%CI** | ***p*** |
| *Overall Survival* | | | | | | | |
| Median | | | | *< 0.001* | | | *0.239* |
| ≤ 73.8 (*reference*) | 138/49 | 1 | - | | 1 | - | |
| > 73.8 | 138/26 | 0.45 | *0.28 – 0.73* | | 0.74 | *0.44 – 1.2* | |
| Tertiles | | | | *< 0.001* | | | *0.151* |
| ≤ 64.7 (*reference*) | 92/41 | 1 | - | | 1 | - | |
| 64.7 – 85.6 | 91/15 | 0.29 | *0.16 – 0.52* | | 0.55 | *0.30 – 1.0* | |
| > 85.6 | 93/19 | 0.36 | *0.21 – 0.62* | | 0.67 | *0.37 – 1.2* | |
| Continuous variable | 276/75 | 0.984 | *0.976–0.993* | *< 0.001* | 0.997 | *0.989–1.004* | *0.388* |
| *Progression Free Survival* | | | | | | | |
| Median | | | | *0.048* | | | *0.979* |
| ≤ 73.8 (*reference*) | 137/55 | 1 | - | | 1 | - | |
| > 73.8 | 138/42 | 0.67 | *0.45 – 1.0* | | 1.0 | *0.65 – 1.5* | |
| Tertiles | | | | *0.006* | | | *0.703* |
| ≤ 64.7 (*reference*) | 91/45 | 1 | - | | 1 | - | |
| 64.7 – 85.6 | 91/22 | 0.38 | *0.23 – 0.64* | | 0.68 | *0.39 – 1.2* | |
| > 85.6 | 93/30 | 0.53 | *0.34 – 0.85* | | 0.90 | *0.54 – 1.5* | |
| Continuous variable | 275/97 | 0.992 | *0.986–0.999* | *0.015* | 1.0 | *0.994–1.006* | *0.919* |

N/O, Number of patients/Outcome (deaths for the overall and events for the progression free survival); HR, Hazard Ratio. Multivariable analysis: HRs are adjusted by *MYCN* status, age, and stage at diagnosis.

**Supplementary Table S12.** Survival of the cohort of 498 NB patients from the SEQC-498 data set, in relation to *NUDT9P1* gene expression values, evaluated by the Cox model.

| | | Univariable analysis | | | Multivariable analysis | | |
|---|---|---|---|---|---|---|---|
| **Gene expression** | **N/O** | **HR** | **95%CI** | ***p*** | **HR** | **95%CI** | ***p*** |
| *Overall Survival* | | | | | | | |
| Median | | | | *< 0.001* | | | *< 0.001* |
| ≤ 1.330 (*reference*) | 249/80 | 1 | - | | 1 | - | |
| > 1.330 | 249/25 | 0.25 | *0.16 – 0.39* | | 0.36 | *0.23 – 0.58* | |
| Tertiles | | | | *< 0.001* | | | *< 0.001* |
| ≤ 1.237 (*reference*) | 166/67 | 1 | - | | 1 | - | |
| 1.237 – 1.412 | 162/25 | 0.28 | *0.17 – 0.44* | | 0.56 | *0.34 – 0.92* | |
| > 1.412 | 170/13 | 0.13 | *0.07 – 0.24* | | 0.24 | *0.13 – 0.44* | |
| Continuous variable | 498/105 | 0.01 | *0.003 – 0.04* | < 0.001 | 0.06 | *0.02 – 0.20* | < 0.001 |
| *Event Free Survival* | | | | | | | |
| Median | | | | *< 0.001* | | | *< 0.001* |
| ≤ 1.330 (*reference*) | 249/198 | 1 | - | | 1 | - | |
| > 1.330 | 249/63 | 0.43 | *0.32 – 0.59* | | 0.52 | *0.38 – 0.72* | |
| Tertiles | | | | *< 0.001* | | | *< 0.001* |
| ≤ 1.237 (*reference*) | 166/90 | 1 | - | | 1 | - | |
| 1.237 – 1.412 | 162/55 | 0.48 | *0.35 – 0.68* | | 0.71 | *0.49 – 1.0* | |
| > 1.412 | 170/38 | 0.30 | *0.21 – 0.44* | | 0.41 | *0.28 – 0.62* | |
| Continuous variable | 498/183 | 0.08 | *0.03 – 0.19* | *< 0.001* | 0.19 | *0.08 – 0.43* | *< 0.001* |

N/O, Number of patients/Outcome (deaths for the overall and events for the event free survival); HR, Hazard Ratio. Multivariable analysis: HRs are adjusted by *MYCN* status, age, and stage at diagnosis.

**Supplementary Table S13.** Survival of the cohort of 283 NB patients from the NRC-283 data set, in relation to *NUDT9P1* gene expression values, evaluated by the Cox model.

| | | Univariable analysis | | | Multivariable analysis | | |
|---|---|---|---|---|---|---|---|
| **Gene expression** | **N/O** | **HR** | **95%CI** | ***p*** | **HR** | **95%CI** | ***p*** |
| *Overall Survival* | | | | | | | |
| Median | | | | *< 0.001* | | | *0.444* |
| ≤ 21 (*reference*) | 138/50 | 1 | - | | 1 | - | |
| > 21 | 138/25 | 0.40 | *0.25 – 0.65* | | 0.82 | *0.48 – 1.42* | |
| Tertiles | | | | *< 0.001* | | | *0.413* |
| ≤ 19.5 (*reference*) | 91/39 | 1 | - | | 1 | - | |
| 19.5 – 23.9 | 92/18 | 0.37 | *0.21 – 0.65* | | 0.66 | *0.37 – 1.2* | |
| > 23.9 | 93/18 | 0.35 | *0.20 – 0.61* | | 0.83 | *0.45 – 1.5* | |
| Continuous variable | 276/75 | 0.91 | *0.87–0.96* | *< 0.001* | 0.97 | *0.93 – 1.02* | *0.237* |
| *Progression Free Survival* | | | | | | | |
| Median | | | | *< 0.001* | | | *0.225* |
| ≤ 21.0 (*reference*) | 137/62 | 1 | - | | 1 | - | |
| > 21.0 | 138/35 | 0.46 | *0.30 – 0.70* | | 0.76 | *0.49 – 1.2* | |
| Tertiles | | | | *< 0.001* | | | *0.175* |
| ≤ 19.5 (*reference*) | 90/46 | 1 | - | | 1 | - | |
| 19.5 – 23.9 | 92/28 | 0.49 | *0.31 – 0.79* | | 0.79 | *0.49 – 1.3* | |
| > 23.9 | 93/23 | 0.38 | *0.23 – 0.62* | | 0.70 | *0.41 – 1.2* | |
| Continuous variable | 275/97 | 0.93 | *0.89 – 0.97* | *< 0.001* | 0.97 | *0.93 – 1.01* | *0.104* |

N/O, Number of patients/Outcome (deaths for the overall and events for the progression free survival); HR, Hazard Ratio. Multivariable analysis: HRs are adjusted by *MYCN* status, age, and stage at diagnosis.

**Supplementary Table S14.** Survival of the cohort of 786 NB patients in the Cangelosi et al. *in silico* dataset, in relation to *ACTA2* gene expression values, evaluated by the Cox model.

| | | Univariable analysis | | | Multivariable analysis | | |
|---|---|---|---|---|---|---|---|
| **Gene expression** | **N/O** | **HR** | **95%CI** | ***p*** | **HR** | **95%CI** | ***p*** |
| *Overall Survival* | | | | | | | |
| Median | | | | *0.254* | | | *0.585* |
| ≤ 4.518 (*reference*) | 393/107 | 1 | - | | 1 | - | |
| > 4.518 | 393/122 | 1.2 | *0.90 – 1.5* | | 1.1 | *0.83– 1.4* | |
| Tertiles | | | | *0.230* | | | *0.957* |
| ≤ 4.065 (*reference*) | 262/77 | 1 | - | | 1 | - | |
| 4.065 – 4.954 | 262/67 | 0.86 | *0.62 – 1.2* | | 0.95 | *0.69 – 1.3* | |
| > 4.954 | 262/85 | 1.1 | *0.83 – 1.5* | | 0.99 | *0.73 – 1.4* | |
| Continuous variable | 786/229 | 0.97 | *0.86 – 1.1* | *0.664* | 0.99 | *0.89 – 1.1* | *0.914* |
| *Event Free Survival* | | | | | | | |
| Median | | | | *0.083* | | | *0.251* |
| ≤ 4.518 (*reference*) | 389/148 | 1 | - | | 1 | - | |
| > 4.518 | 380/172 | 1.2 | *0.97 – 1.5* | | 1.1 | *0.91 – 1.4* | |
| Tertiles | | | | *0.046* | | | *0.186* |
| ≤ 4.065 (*reference*) | 259/101 | 1 | - | | 1 | - | |
| 4.065 – 4.954 | 257/96 | 0.93 | *0.70 – 1.2* | | 1.00 | *0.75 – 1.3* | |
| > 4.954 | 253/123 | 1.3 | *1.0 – 1.7* | | 1.2 | *0.91 – 1.6* | |
| Continuous variable | 769/320 | 1.08 | *0.97 – 1.2* | *0.150* | 1.07 | *0.97 – 1.17* | *0.199* |

N/O = Number of patients/Outcome (deaths for the overall and events for the event free survival). HR: Hazard Ratio. Multivariable analysis: HRs are adjusted by *MYCN* status, age, and stage at diagnosis.

**Supplementary Table S15.** Survival of the cohort of 498 NB patients in the SEQC-498 *in silico* dataset, in relation to *ANKRD1* gene expression values, evaluated by the Cox model.

| | | Univariable analysis | | | Multivariable analysis | | |
|---|---|---|---|---|---|---|---|
| **Gene expression** | **N/O** | **HR** | **95%CI** | ***p*** | **HR** | **95%CI** | ***p*** |
| *Overall Survival* | | | | | | | |
| Median | | | | *0.028* | | | *0.227* |
| ≤ 1.236 (*reference*) | 249/61 | 1 | - | | 1 | - | |
| > 1.236 | 249/44 | 0.65 | *0.44 – 0.96* | | 0.78 | *0.53 –1.2* | |
| Tertiles | | | | *0.021* | | | *0.326* |
| ≤ 1.155 (*reference*) | 166/44 | 1 | - | | 1 | - | |
| 1.155 – 1.399 | 166/32 | 0.69 | *0.44 – 1.1* | | 0.67 | *0.43 – 1.1* | |
| > 1.399 | 166/29 | 0.58 | *0.36 – 0.93* | | 0.82 | *0.51 – 1.3* | |
| Continuous variable | 498/105 | 1.00 | *0.94 – 1.06* | *0.915* | 1.04 | *0.98 – 1.1* | *0.318* |
| *Event Free Survival* | | | | | | | |
| Median | | | | *0.218* | | | *0.549* |
| ≤ 1.236 (*reference*) | 249/96 | 1 | - | | 1 | - | |
| > 1.236 | 249/87 | 0.83 | *0.62 – 1.1* | | 0.91 | *0.68 – 1.2* | |
| Tertiles | | | | *0.113* | | | *0.421* |
| ≤ 1.155 (*reference*) | 166/66 | 1 | - | | 1 | - | |
| 1.155 – 1.399 | 166/63 | 0.88 | *0.63 – 1.2* | | 0.89 | *0.63 – 1.3* | |
| > 1.399 | 166/54 | 0.75 | *0.52 – 1.1* | | 0.86 | *0.59 – 1.3* | |
| Continuous variable | 498/183 | 1.05 | *1.02 – 1.09* | *0.014* | 1.07 | *1.03 – 1.11* | *0.002* |

**Supplementary Table S16.** Survival of the cohort of 786 NB patients in the Cangelosi et al. *in silico* dataset, in relation to *ANRKD22* gene expression values, evaluated by the Cox model.

| | | Univariable analysis | | | Multivariable analysis | | |
|---|---|---|---|---|---|---|---|
| **Gene expression** | **N/O** | **HR** | **95%CI** | ***p*** | **HR** | **95%CI** | ***p*** |
| *Overall Survival* | | | | | | | |
| Median | | | | *0.623* | | | *0.177* |
| ≤ 0.562 (*reference*) | 393/111 | 1 | - | | 1 | - | |
| > 0.562 | 393/118 | 1.1 | *0.82 – 1.4* | | 1.2 | *0.92 – 1.6* | |
| Tertiles | | | | *0.569* | | | *0.679* |
| ≤ -0.202 (*reference*) | 262/86 | 1 | - | | 1 | - | |
| -0.202 – 1.171 | 262/61 | 0.66 | *0.48 – 0.92* | | 1.1 | *0.78 – 1.5* | |
| > 1.171 | 262/82 | 0.92 | *0.68 – 1.2* | | 1.1 | *0.78 – 1.4* | |
| Continuous variable | 786/229 | 1.02 | *0.95 – 1.09* | *0.602* | 1.06 | *0.99 – 1.1* | *0.098* |
| *Event Free Survival* | | | | | | | |
| Median | | | | | | | |
| ≤ 0.562 (*reference*) | | | | *0.211* | | | *0.197* |
| > 0.562 | 384/151 | 1 | - | | 1 | - | |
| Tertiles | 385/169 | 1.2 | *0.92 – 1.4* | | 1.2 | *0.93 – 1.4* | |
| ≤ -0.202 (*reference*) | | | | *0.817* | | | *0.654* |
| -0.202 – 1.171 | 254/112 | 1 | - | | 1 | - | |
| > 1.171 | 259/91 | 0.76 | *0.58 – 1.0* | | 1.0 | *0.75 – 1.3* | |
| Median | 256/117 | 1.0 | *0.79 – 1.3* | | 1.1 | *0.82 – 1.4* | |
| Continuous variable | 769/320 | 1.03 | *0.97 – 1.09* | *0.385* | 1.04 | *0.98 – 1.09* | *0.217* |

**Supplementary Table S17.** Survival of the cohort of 786 NB patients in the Cangelosi et al. *in silico* dataset, in relation to *ANXA11* gene expression values, evaluated by the Cox model.

| | | Univariable analysis | | | Multivariable analysis | | |
|---|---|---|---|---|---|---|---|
| **Gene expression** | **N/O** | **HR** | **95%CI** | ***p*** | **HR** | **95%CI** | ***p*** |
| *Overall Survival* | | | | | | | |
| Median | | | | *0.410* | | | *0.028* |
| ≤ 2.922 (*reference*) | 393/108 | 1 | - | | 1 | - | |
| > 2.922 | 393/121 | 1.1 | *0.86 – 1.4* | | 1.3 | *1.0 – 1.7* | |
| Tertiles | | | | *0.523* | | | *0.157* |
| ≤ 2.578 (*reference*) | 262/79 | 1 | - | | 1 | - | |
| 2.578 – 3.253 | 262/62 | 0.74 | *0.53 – 1.0* | | 1.1 | *0.81– 1.6* | |
| > 3.253 | 262/88 | 1.1 | *0.81 – 1.5* | | 1.2 | *0.92 – 1.7* | |
| Continuous variable | 786/229 | 1.04 | *0.89 – 1.21* | *0.652* | 1.13 | *0.98 – 1.3* | *0.097* |
| *Event Free Survival* | | | | | | | |
| Median | | | | *0.037* | | | *0.004* |
| ≤ 2.922 (*reference*) | 393/147 | 1 | - | | 1 | - | |
| > 2.922 | 376/173 | 1.3 | *1.0 – 1.6* | | 1.4 | *1.1 – 1.7* | |
| Tertiles | | | | *0.100* | | | *0.038* |
| ≤ 2.578 (*reference*) | 262/101 | 1 | - | | 1 | - | |
| 2.578 – 3.253 | 261/102 | 1.0 | *0.76 – 1.3* | | 1.3 | *1.0 – 1.8* | |
| > 3.253 | 246/117 | 1.2 | *0.96– 1.6* | | 1.3 | *1.0 – 1.7* | |
| Continuous variable | 769/320 | 1.11 | *0.97 – 1.28* | *0.120* | 1.16 | *1.02 – 1.32* | *0.023* |

**Supplementary Table S18.** Survival of the cohort of 786 NB patients in the Cangelosi et al. *in silico* dataset, in relation to *ATAD1* gene expression values, evaluated by the Cox model.

| | | Univariable analysis | | | Multivariable analysis | | |
|---|---|---|---|---|---|---|---|
| **Gene expression** | **N/O** | **HR** | **95%CI** | ***p*** | **HR** | **95%CI** | ***p*** |
| *Overall Survival* | | | | | | | |
| Median | | | | *0.156* | | | *0.389* |
| ≤ 4.096 (*reference*) | 393/105 | 1 | - | | 1 | - | |
| > 4.096 | 393/124 | 1.2 | *0.93 – 1.6* | | 1.1 | *0.86 – 1.5* | |
| Tertiles | | | | *0.292* | | | *0.811* |
| ≤ 3.935 (*reference*) | 262/73 | 1 | - | | 1 | - | |
| 3.935 – 4.259 | 262/71 | 0.95 | *0.69 – 1.3* | | 0.97 | *0.70 – 1.4* | |
| > 4.259 | 262/85 | 1.2 | *0.86 – 1.6* | | 1.0 | *0.75 – 1.4* | |
| Continuous variable | 786/229 | 1.01 | *0.76 – 1.34* | *0.947* | 0.98 | *0.76 – 1.26* | *0.868* |
| *Event Free Survival* | | | | | | | |
| Median | | | | *0.828* | | | *0.753* |
| ≤ 4.096 (*reference*) | 377/155 | 1 | - | | 1 | - | |
| > 4.096 | 392/165 | 1.0 | *0.82 – 1.3* | | 0.96 | *0.77 – 1.2* | |
| Tertiles | | | | *0.424* | | | *0.937* |
| ≤ 3.935 (*reference*) | 247/99 | 1 | - | | 1 | - | |
| 3.935 – 4.259 | 260/106 | 1.0 | *0.76 – 1.3* | | 0.99 | *0.75 – 1.3* | |
| > 4.259 | 262/115 | 1.1 | *0.85 – 1.5* | | 1.0 | *0.76 – 1.3* | |
| Continuous variable | 769/320 | 1.03 | *0.80 – 1.34* | *0.808* | 0.99 | *0.78 – 1.27* | *0.955* |

**Supplementary Table S19.** Survival of the cohort of 786 NB patients in the Cangelosi et al. *in silico* dataset, in relation to *BMPR1A* gene expression values, evaluated by the Cox model.

| | | Univariable analysis | | | Multivariable analysis | | |
|---|---|---|---|---|---|---|---|
| **Gene expression** | **N/O** | **HR** | **95%CI** | ***p*** | **HR** | **95%CI** | ***p*** |
| *Overall Survival* | | | | | | | |
| Median | | | | *< 0.001* | | | *0.764* |
| ≤ 3.070 (*reference*) | 393/93 | 1 | - | | 1 | - | |
| > 3.070 | 393/136 | 1.6 | *1.2 – 2.1* | | 0.96 | *0.73 – 1.3* | |
| Tertiles | | | | *< 0.001* | | | *0.601* |
| ≤ 2.924 (*reference*) | 262/63 | 1 | - | | 1 | - | |
| 2.924 – 3.249 | 262/68 | 1.1 | *0.77 – 1.5* | | 1.1 | *0.79 – 1.6* | |
| > 3.249 | 262/98 | 1.8 | *1.3 – 2.4* | | 0.93 | *0.67– 1.3* | |
| Continuous variable | 786/229 | 1.92 | *1.4 – 2.7* | *< 0.001* | 0.94 | *0.70 – 1.2* | *0.654* |
| *Event Free Survival* | | | | | | | |
| Median | | | | *0.003* | | | *0.937* |
| ≤ 3.070 (*reference*) | 377/138 | 1 | - | | 1 | - | |
| > 3.070 | 392/182 | 1.4 | *1.1 – 1.8* | | 0.99 | *0.79 – 1.2* | |
| Tertiles | | | | *0.002* | | | *0.907* |
| ≤ 2.924 (*reference*) | 247/91 | 1 | - | | 1 | - | |
| 2.924 – 3.249 | 261/101 | 1.1 | *0.82 – 1.4* | | 1.1 | *0.81 – 1.4* | |
| > 3.249 | 261/128 | 1.5 | *1.2 – 2.0* | | 0.99 | *0.74 – 1.3* | |
| Continuous variable | 764/320 | 1.63 | *1.22– 2.17* | *0.001* | 0.99 | *0.75 – 1.29* | *0.919* |

**Supplementary Table S20.** Survival of the cohort of 498 NB patients in the SEQC-498 *in silico* dataset, in relation to *C10orf116* gene expression values, evaluated by the Cox model.

| | | Univariable analysis | | | Multivariable analysis | | |
|---|---|---|---|---|---|---|---|
| **Gene expression** | **N/O** | **HR** | **95%CI** | ***p*** | **HR** | **95%CI** | ***p*** |
| *Overall Survival* | | | | | | | |
| Median | | | | *0.043* | | | *0.386* |
| ≤ 3.999 (*reference*) | 249/62 | 1 | - | | 1 | - | |
| > 3.999 | 249/43 | 0.67 | *0.45 – 0.99* | | 0.84 | *0.56 – 1.3* | |
| Tertiles | | | | *0.006* | | | *0.031* |
| ≤ 3.293 (*reference*) | 166/47 | 1 | - | | 1 | - | |
| 3.293 – 4.982 | 166/31 | 0.59 | *0.38 – 0.93* | | 0.81 | *0.51 – 1.3* | |
| > 4.982 | 166/27 | 0.53 | *0.33 – 0.85* | | 0.58 | *0.36 – 0.96* | |
| Continuous variable | 498/105 | 0.97 | *0.90 – 1.03* | *0.272* | 0.98 | *0.92 – 1.03* | *0.441* |
| *Event Free Survival* | | | | | | | |
| Median | | | | *0.288* | | | *0.970* |
| ≤ 3.999 (*reference*) | 249/97 | 1 | - | | 1 | - | |
| > 3.999 | 249/86 | 0.85 | *0.64 – 1.1* | | 0.99 | *0.73 – 1.3* | |
| Tertiles | | | | *0.234* | | | *0.511* |
| ≤ 3.293 (*reference*) | 166/67 | 1 | - | | 1 | - | |
| 3.293 – 4.982 | 166/58 | 0.82 | *0.58 – 1.2* | | 1.0 | *0.71 – 1.5* | |
| > 4.982 | 166/58 | 0.81 | *0.57 – 1.1* | | 0.88 | *0.61 – 1.3* | |
| Continuous variable | 498/183 | 0.98 | *0.94 – 1.03* | *0.474* | 0.99 | *0.95 – 1.03* | *0.636* |

**Supplementary Table S21.** Survival of the cohort of 709 NB patients in the E-MTAB-1781 *in silico* dataset, in relation to *C10orf57* (alias *FLJ3263*, Agilent probe: A_23_P97853) gene expression values, evaluated by the Cox model.

| | | Univariable analysis | | | Multivariable analysis | | |
|---|---|---|---|---|---|---|---|
| **Gene expression** | **N/O** | **HR** | **95%CI** | ***p*** | **HR** | **95%CI** | ***p*** |
| *Overall Survival* | | | | | | | |
| Median | | | | *< 0.001* | | | *0.993* |
| ≤ -0.170 (*reference*) | 354/55 | 1 | - | | 1 | - | |
| > -0.170 | 355/106 | 2.0 | *1.5 – 2.8* | | 1.00 | *0.71 – 1.4* | |
| Tertiles | | | | *< 0.001* | | | *0.920* |
| ≤ -0.301 (*reference*) | 236/33 | 1 | - | | 1 | - | |
| -0.301 – -0.041 | 236/48 | 1.5 | *0.96 – 2.3* | | 0.99 | *0.63 – 1.6* | |
| > -0.041 | 237/80 | 2.7 | *1.8 – 4.0* | | 0.98 | *0.64 – 1.5* | |
| Continuous variable | 709/161 | 3.56 | *2.21 – 5.74* | *< 0.001* | 0.72 | *0.44 – 1.2* | *0.178* |
| *Event Free Survival* | | | | | | | |
| Median | | | | *< 0.001* | | | *0.084* |
| ≤ -0.170 (*reference*) | 343/92 | 1 | - | | 1 | - | |
| > -0.170 | 352/164 | 1.9 | *1.5 – 2.5* | | 1.3 | *0.97 – 1.7* | |
| Tertiles | | | | *< 0.001* | | | *0.046* |
| ≤ -0.301 (*reference*) | 227/55 | 1 | - | | 1 | - | |
| -0.301 – -0.041 | 232/80 | 1.5 | *1.1 – 2.1* | | 1.3 | *0.89 – 1.8* | |
| > -0.041 | 236/121 | 2.5 | *1.8 – 3.5* | | 1.4 | *1.0 – 2.0* | |
| Continuous variable | 695/256 | 2.73 | *1.87 – 3.98* | *< 0.001* | 1.07 | *0.72 – 1.59* | *0.742* |

**Supplementary Table S22.** Survival of the cohort of 786 NB patients in the Cangelosi et al. *in silico* dataset, in relation to *C10prf58* (alias *FAM213A*) gene expression values, evaluated by the Cox model.

| | | Univariable analysis | | | Multivariable analysis | | |
|---|---|---|---|---|---|---|---|
| **Gene expression** | **N/O** | **HR** | **95%CI** | ***p*** | **HR** | **95%CI** | ***p*** |
| *Overall Survival* | | | | | | | |
| Median | | | | *< 0.001* | | | *0.003* |
| ≤ 4.032 (*reference*) | 393/62 | 1 | - | | 1 | - | |
| > 4.032 | 393/167 | 3.2 | *2.4 – 4.4* | | 1.6 | *1.2 – 2.2* | |
| Tertiles | | | | *< 0.001* | | | *0.082* |
| ≤ 3.804 (*reference*) | 262/43 | 1 | - | | 1 | - | |
| 3.804 – 4.260 | 262/74 | 1.8 | *1.3 – 2.7* | | 1.3 | *0.86 – 1.9* | |
| > 4.260 | 262/112 | 3.1 | *2.2 – 4.5* | | 1.4 | *0.96 – 2.0* | |
| Continuous variable | 786/229 | 2.57 | *2.04 – 3.25* | *< 0.001* | 1.21 | *0.94 – 1.57* | *0.137* |
| *Event Free Survival* | | | | | | | |
| Median | | | | *< 0.001* | | | *0.016* |
| ≤ 4.032 (*reference*) | 377/113 | 1 | - | | 1 | - | |
| > 4.032 | 392/207 | 2.1 | *1.7 – 2.6* | | 1.4 | *1.1 – 1.7* | |
| Tertiles | | | | *< 0.001* | | | *0.168* |
| ≤ 3.804 (*reference*) | 247/73 | 1 | - | | 1 | - | |
| 3.804 – 4.260 | 261/112 | 1.6 | *1.2 – 2.2* | | 1.3 | *0.95 – 1.7* | |
| > 4.260 | 261/135 | 2.1 | *1.6 – 2.8* | | 1.3 | *0.93 – 1.7* | |
| Continuous variable | 769/320 | 1.86 | *1.5 – 2.3* | *< 0.001* | 1.15 | *0.92 – 1.4* | *0.224* |

**Supplementary Table S23.** Survival of the cohort of 498 NB patients in the SEQC-498. *in silico* dataset, in relation to *CFL1P1* gene expression values, evaluated by the Cox model.

| | | Univariable analysis | | | Multivariable analysis | | |
|---|---|---|---|---|---|---|---|
| **Gene expression** | **N/O** | **HR** | **95%CI** | ***p*** | **HR** | **95%CI** | ***p*** |
| *Overall Survival* | | | | | | | |
| Median | | | | *0.045* | | | *0.035* |
| ≤ 2.078 (*reference*) | 249/61 | 1 | - | | 1 | - | |
| > 2.078 | 249/44 | 0.67 | *0.46 – 0.99* | | 0.65 | *0.44 – 0.97* | |
| Tertiles | | | | *0.070* | | | *0.286* |
| ≤ 1.882 (*reference*) | 166/48 | 1 | - | | 1 | - | |
| 1.882 – 2.342 | 166/22 | 0.40 | *0.24 – 0.66* | | 0.57 | *0.34 – 0.96* | |
| > 2.342 | 166/35 | 0.69 | *0.45 – 1.1* | | 0.79 | *0.51 – 1.2* | |
| Continuous variable | 498/105 | 0.71 | *0.48 – 1.04* | *0.075* | 0.82 | *0.57 – 1.2* | *0.262* |
| *Event Free Survival* | | | | | | | |
| Median | | | | *0.029* | | | *0.043* |
| ≤ 2.078 (*reference*) | 249/104 | 1 | - | | 1 | - | |
| > 2.078 | 249/79 | 0.72 | *0.54 – 0.97* | | 0.73 | *0.54 – 0.99* | |
| Tertiles | | | | *0.012* | | | *0.084* |
| ≤ 1.882 (*reference*) | 166/82 | 1 | - | | 1 | - | |
| 1.882 – 2.342 | 166/43 | 0.46 | *0.32 – 0.67* | | 0.60 | *0.41 – 0.88* | |
| > 2.342 | 166/58 | 0.67 | *0.48 – 0.94* | | 0.75 | *0.52 – 1.1* | |
| Continuous variable | 498/183 | 0.74 | *0.55 – 1.00* | *0.043* | 0.82 | *0.62 – 1.1* | *0.156* |

**Supplementary Table S24.** Survival of the cohort of of 786 NB patients in the Cangelosi et al. *in silico* dataset, in relation to *CH25H* gene expression values, evaluated by the Cox model.

| | | Univariable analysis | | | Multivariable analysis | | |
|---|---|---|---|---|---|---|---|
| **Gene expression** | **N/O** | **HR** | **95%CI** | ***p*** | **HR** | **95%CI** | ***p*** |
| *Overall Survival* | | | | | | | |
| Median | | | | *0.724* | | | *0.559* |
| ≤ 0.587 (*reference*) | 393/110 | 1 | - | | 1 | - | |
| > 0.587 | 393/119 | 1.0 | *0.81 – 1.4* | | 1.1 | *0.83 – 1.4* | |
| Tertiles | | | | *0.780* | | | *0.568* |
| ≤ 0.264 (*reference*) | 262/71 | 1 | - | | 1 | - | |
| 0.264 – 1.058 | 262/81 | 1.2 | *0.84 – 1.6* | | 1.2 | *0.90 – 1.7* | |
| > 1.058 | 262/77 | 1.0 | *0.76 – 1.4* | | 1.1 | *0.80 – 1.5* | |
| Continuous variable | 486/229 | 0.97 | *0.86 – 1.1* | *0.564* | 1.01 | *0.91 – 1.13* | *0.789* |
| *Event Free Survival* | | | | | | | |
| Median | | | | *0.870* | | | *0.793* |
| ≤ 0.587 (*reference*) | 389/160 | 1 | - | | 1 | - | |
| > 0.587 | 380/160 | 0.98 | *0.79 – 1.2* | | 0.97 | *0.78 – 1.2* | |
| Tertiles | | | | *0.527* | | | *0.648* |
| ≤ 0.264 (*reference*) | 261/109 | 1 | - | | 1 | - | |
| 0.264 – 1.058 | 256/109 | 1.0 | *0.78 – 1.3* | | 0.99 | *0.76 – 1.3* | |
| > 1.058 | 252/102 | 0.92 | *0.70 – 1.2* | | 0.94 | *0.72 – 1.2* | |
| Continuous variable | 769/320 | 0.95 | *0.86 – 1.04* | *0.306* | 0.98 | *0.89 –1.07* | *0.665* |

**Supplementary Table S25.** Survival of the cohort of 498 NB patients in the SEQC-498 *in silico* dataset, in relation to *DYDC1* gene expression, evaluated by the Cox model.

| | | Univariable analysis | | | Multivariable analysis | | |
|---|---|---|---|---|---|---|---|
| **Gene expression** | **N/O** | **HR** | **95%CI** | ***p*** | **HR** | **95%CI** | ***p*** |
| *Overall Survival* | | | | *< 0.001* | | | *0.023* |
| ≤ 1.01* (*reference)* | 321/43 | 1 | - | | 1 | - | |
| ≤ 1.01* | 177/62 | 3.1 | *2.1 – 4.5* | | 1.6 | *1.1 – 2.4* | |
| *Event Free Survival* | | | | *< 0.001* | | | *0.007* |
| ≤ 1.01* (*reference)* | 321/89 | 1 | - | | 1 | - | |
| > 1.01 | 177/94 | 2.3 | *1.8 – 3.1* | | 1.5 | *1.1 – 2.1* | |

* Allegedly corresponding to the absence of gene expression.

**Supplementary Table S26.** Association between *DYDC1* gene expression and main patients characteristics at diagnosis in a cohort of 498 NB patients in the SEQC-498 *in silico* dataset.

| | Gene expression ≤ 1.01* | | Gene expression > 1.01 | | ***p*** |
|---|---|---|---|---|---|
| **Patients characteristics** | N | % | N | % | |
| *MYCN* status | | | | | *< 0.001* |
| Not amplified | 283 | 70.6 | 118 | 29.4 | |
| Amplified | 36 | 39.1 | 56 | 60.9 | |
| Age | | | | | *< 0.001* |
| ≤ 18 months | 224 | 73.4 | 81 | 26.6 | |
| > 18 months | 97 | 50.3 | 96 | 49.7 | |
| Stage | | | | | *< 0.001* |
| Localized | 191 | 72.9 | 71 | 27.1 | |
| Stage 4 | 90 | 49.2 | 93 | 50.8 | |
| Stage 4S | 40 | 75.5 | 13 | 24.5 | |
| Total | 321 | 64.5 | 177 | 35.5 | |

* Allegedly corresponding to the absence of gene expression.

**Supplementary Table S27.** Survival of the cohort of 498 NB patients in the SEQC-498. *in silico* dataset, in relation to *DYDC2* gene expression values, evaluated by the Cox model.

| | | Univariable analysis | | | Multivariable analysis | | |
|---|---|---|---|---|---|---|---|
| **Gene expression** | **N/O** | **HR** | **95%CI** | ***p*** | **HR** | **95%CI** | ***p*** |
| *Overall Survival* | | | | | | | |
| Median | | | | *< 0.001* | | | *0.290* |
| ≤ 1.373 (*reference*) | 249/24 | 1 | - | | 1 | - | |
| > 1.373 | 249/81 | 4.1 | *2.6 – 6.4* | | 1.3 | *0.79 – 2.2* | |
| Tertiles | | | | *< 0.001* | | | *0.105* |
| ≤ 1.255 (*reference*) | 166/13 | 1 | - | | 1 | - | |
| 1.255 – 1.563 | 166/27 | 2.2 | *1.1 – 4.2* | | 1.0 | *0.52 – 2.0* | |
| > 1.563 | 166/65 | 6.4 | *3.5 – 11.6* | | 1.5 | *0.77 – 2.9* | |
| Continuous variable | 498/105 | 2.14 | *1.75 – 2.60* | *< 0.001* | 1.53 | *1.10 – 2.12* | *0.021* |
| *Event Free Survival* | | | | | | | |
| Median | | | | *< 0.001* | | | *0.551* |
| ≤ 1.373 (*reference*) | 249/63 | 1 | - | | 1 | - | |
| > 1.373 | 249/120 | 2.2 | *1.6 – 3.0* | | 1.1 | *0.78 – 1.6* | |
| Tertiles | | | | *< 0.001* | | | *0.635* |
| ≤ 1.255 (*reference*) | 166/39 | 1 | - | | 1 | - | |
| 1.255 – 1.563 | 166/57 | 1.6 | *1.0 – 2.4* | | 1.0 | *0.66 – 1.6* | |
| > 1.563 | 166/87 | 2.8 | *1.9 – 4.0* | | 1.1 | *0.71 – 1.7* | |
| Continuous variable | 498/183 | 1.9 | *1.6 – 2.3* | *< 0.001* | 1.35 | *1.01 – 1.80* | *0.055* |

**Supplementary Table S28.** Survival of the cohort of 498 NB patients in the SEQC-498 *in silico* dataset, in relation to *EIF5AL1* gene expression values, evaluated by the Cox model.

| | | Univariable analysis | | | Multivariable analysis | | |
|---|---|---|---|---|---|---|---|
| **Gene expression** | **N/O** | **HR** | **95%CI** | ***p*** | **HR** | **95%CI** | ***p*** |
| *Overall Survival* | | | | | | | |
| Median | | | | *< 0.001* | | | *0.157* |
| ≤ 67.788 (*reference*) | 249/35 | 1 | - | | 1 | - | |
| > 67.788 | 249/70 | 2.3 | *1.5 – 3.4* | | 1.4 | *0.88 – 2.2* | |
| Tertiles | | | | *< 0.001* | | | *0.012* |
| ≤ 60.288 (*reference*) | 166/25 | 1 | - | | 1 | - | |
| 60.288 – 74.043 | 166/19 | 0.76 | *0.42 – 1.4* | | 1.1 | *0.59 – 2.0* | |
| > 74.043 | 166/61 | 3.0 | *1.9 – 4.7* | | 1.9 | *1.1 – 3.1* | |
| Continuous variable | 498/105 | 1.02 | *1.01 – 1.03* | *< 0.001* | 1.01 | *1.00 – 1.01* | *0.163* |
| *Event Free Survival* | | | | | | | |
| Median | | | | *< 0.001* | | | *0.036* |
| ≤ 67.788 (*reference*) | 249/73 | 1 | - | | 1 | - | |
| > 67.788 | 249/110 | 1.7 | *1.3 – 2.3* | | 1.4 | *1.0 – 2.0* | |
| Tertiles | | | | *< 0.001* | | | *0.011* |
| ≤ 60.288 (*reference*) | 166/52 | 1 | - | | 1 | - | |
| 60.288 – 74.043 | 166/47 | 0.94 | *0.64 – 1.4* | | 1.2 | *0.81 – 1.8* | |
| > 74.043 | 166/84 | 2.0 | *1.4 – 2.8* | | 1.6 | *1.1 – 2.4* | |
| Continuous variable | 498/183 | 1.01 | *1.00 – 1.02* | *< 0.001* | 1.01 | *1.00 – 1.01* | *0.019* |

**Supplementary Table S29.** Survival of the cohort of 498 NB patients in the SEQC-498. *in silico* dataset, in relation to *FAM22A* gene expression values, evaluated by the Cox model.

| | | Univariable analysis | | | Multivariable analysis | | |
|---|---|---|---|---|---|---|---|
| **Gene expression** | **N/O** | **HR** | **95%CI** | ***p*** | **HR** | **95%CI** | ***p*** |
| *Overall Survival* | | | | | | | |
| Median | | | | *< 0.001* | | | *0.069* |
| ≤ 6.476 (*reference*) | 249/78 | 1 | - | | 1 | - | |
| > 6.476 | 249/27 | 0.28 | *0.18 – 0.44* | | 0.65 | *0.40 – 1.0* | |
| Tertiles | | | | *< 0.001* | | | *0.056* |
| ≤ 5.292 (*reference*) | 166/64 | 1 | - | | 1 | - | |
| 5.292 – 7.470 | 166/22 | 0.28 | *0.17 – 0.46* | | 0.68 | *0.40 – 1.2* | |
| > 7.470 | 166/19 | 0.22 | *0.13 – 0.37* | | 0.60 | *0.34 – 1.1* | |
| Continuous variable | 498/105 | 0.76 | *0.69 – 0.83* | *< 0.001* | 0.95 | *0.87 – 1.04* | *0.258* |
| *Event Free Survival* | | | | | | | |
| Median | | | | *< 0.001* | | | *0.027* |
| ≤ 6.476 (*reference*) | 249/120 | 1 | - | | 1 | - | |
| > 6.476 | 249/63 | 0.43 | *0.31 – 0.58* | | 0.68 | *0.49 – 0.96* | |
| Tertiles | | | | *< 0.001* | | | *0.016* |
| ≤ 5.292 (*reference*) | 166/91 | 1 | - | | 1 | - | |
| 5.292 – 7.470 | 166/52 | 0.47 | *0.33 – 0.66* | | 0.78 | *0.53 – 1.1* | |
| > 7.470 | 166/40 | 0.33 | *0.23 – 0.48* | | 0.60 | *0.40 – 0.92* | |
| Continuous variable | 498/183 | 0.82 | *0.77 – 0.88* | *< 0.001* | 0.93 | *0.87 – 1.0* | *0.044* |

**Supplementary Table S30.** Survival of the cohort of 498 NB patients in the SEQC-498. *in silico* dataset, in relation to *FAM22D* gene expression values, evaluated by the Cox model.

| | | Univariable analysis | | | Multivariable analysis | | |
|---|---|---|---|---|---|---|---|
| **Gene expression** | **N/O** | **HR** | **95%CI** | ***p*** | **HR** | **95%CI** | ***p*** |
| *Overall Survival* | | | | | | | |
| Median | | | | *< 0.001* | | | *0.022* |
| ≤ 9.545 (*reference*) | 249/80 | 1 | - | | 1 | - | |
| > 9.545 | 249/25 | 0.25 | *0.16 – 0.39* | | 0.57 | *0.35 – 0.93* | |
| Tertiles | | | | *< 0.001* | | | *0.029* |
| ≤ 7.601 (*reference*) | 166/62 | 1 | - | | 1 | - | |
| 7.601 – 11.590 | 166/28 | 0.35 | *0.22 – 0.55* | | 0.93 | *0.56 – 1.5* | |
| > 11.590 | 166/15 | 0.17 | *0.10 – 0.30* | | 0.50 | *0.27 – 0.93* | |
| Continuous variable | 498/105 | 0.82 | *0.78 – 0.87* | *< 0.001* | 0.94 | *0.88 – 0.99* | *0.023* |
| *Event Free Survival* | | | | | | | |
| Median | | | | *< 0.001* | | | *0.017* |
| ≤ 9.545 (*reference*) | 249/121 | 1 | - | | 1 | - | |
| > 9.545 | 249/62 | 0.42 | *0.31 – 0.57* | | 0.66 | *0.47 – 0.93* | |
| Tertiles | | | | *< 0.001* | | | *0.012* |
| ≤ 7.601 (*reference*) | 166/91 | 1 | - | | 1 | - | |
| 7.601 – 11.590 | 166/53 | 0.48 | *0.34 – 0.67* | | 0.80 | *0.54 – 1.2* | |
| > 11.590 | 166/39 | 0.31 | *0.22 – 0.46* | | 0.59 | *0.39 – 0.89* | |
| Continuous variable | 498/183 | 0.88 | *0.84 – 0.91* | *< 0.001* | 0.94 | *0.90 – 0.98* | *0.002* |

**Supplementary Table S31.** Survival of the cohort of 786 NB patients in the Cangelosi et al. *in silico* dataset, in relation to *FAM35A* gene expression values, evaluated by the Cox model.

| | | Univariable analysis | | | Multivariable analysis | | |
|---|---|---|---|---|---|---|---|
| **Gene expression** | **N/O** | **HR** | **95%CI** | ***p*** | **HR** | **95%CI** | ***p*** |
| *Overall Survival* | | | | | | | |
| Median | | | | *0.029* | | | *0.037* |
| ≤ 3.536 (*reference*) | 393/102 | 1 | - | | 1 | - | |
| > 3.536 | 393/127 | 1.3 | *1.0 – 1.7* | | 0.74 | *0.55 – 0.98* | |
| Tertiles | | | | *0.039* | | | *0.077* |
| ≤ 3.396 (*reference*) | 262/75 | 1 | - | | 1 | - | |
| 3.396 – 3.697 | 262/60 | 0.77 | *0.55 – 1.1* | | 0.86 | *0.61 – 1.2* | |
| > 3.697 | 262/94 | 1.4 | *1.0 – 1.8* | | 0.74 | *0.53 – 1.0* | |
| Continuous variable | 786/229 | 1.26 | *0.91 – 1.7* | *0.161* | 0.82 | *0.64 – 1.1* | *0.124* |
| *Event Free Survival* | | | | | | | |
| Median | | | | *0.001* | | | *0.746* |
| ≤ 3.536 (*reference*) | 377/137 | 1 | - | | 1 | - | |
| > 3.536 | 392/183 | 1.4 | *1.1 – 1.8* | | 1.0 | *0.82 – 1.3* | |
| Tertiles | | | | *0.003* | | | *0.714* |
| ≤ 3.396 (*reference*) | 246/91 | 1 | - | | 1 | - | |
| 3.396 – 3.697 | 262/102 | 1.1 | *0.81 – 1.4* | | 1.1 | *0.84 – 1.5* | |
| > 3.697 | 261/127 | 1.5 | *1.1 – 1.9* | | 1.1 | *0.79 – 1.4* | |
| Continuous variable | 769/320 | 1.55 | *1.15 – 2.09* | *0.004* | 1.05 | *0.80 – 1.37* | *0.740* |

**Supplementary Table S32.** Survival of the cohort of 786 NB patients in the Cangelosi et al. *in silico* dataset, in relation to *FAS* gene expression values, evaluated by the Cox model.

| | | Univariable analysis | | | Multivariable analysis | | |
|---|---|---|---|---|---|---|---|
| **Gene expression** | **N/O** | **HR** | **95%CI** | ***p*** | **HR** | **95%CI** | ***p*** |
| *Overall Survival* | | | | | | | |
| Median | | | | *0.207* | | | *0.906* |
| ≤ 1.871 (*reference*) | 393/122 | 1 | - | | 1 | - | |
| > 1.871 | 393/107 | 0.85 | *0.65 – 1.1* | | 0.98 | *0.76 – 1.3* | |
| Tertiles | | | | *0.112* | | | *0.324* |
| ≤ 1.626 (*reference*) | 262/87 | 1 | - | | 1 | - | |
| 1.626 – 2.143 | 262/70 | 0.77 | *0.56 – 1.1* | | 0.89 | *0.65 – 1.2* | |
| > 2.143 | 262/72 | 0.78 | *0.57 – 1.1* | | 0.85 | *0.62 – 1.2* | |
| Continuous variable | 786/229 | 0.85 | *0.70 – 1.04* | *0.118* | 0.96 | *0.80 – 1.15* | *0.644* |
| *Event Free Survival* | | | | | | | |
| Median | | | | *0.456* | | | *0.958* |
| ≤ 1.871 (*reference*) | 378/161 | 1 | - | | 1 | - | |
| > 1.871 | 391/159 | 0.92 | *0.74 – 1.1* | | 1.00 | *0.81 – 1.3* | |
| Tertiles | | | | *0.337* | | | *0.600* |
| ≤ 1.626 (*reference*) | 247/108 | 1 | - | | 1 | - | |
| 1.626 – 2.143 | 260/105 | 0.90 | *0.69 – 1.2* | | 0.98 | *0.75 – 1.3* | |
| > 2.143 | 262/107 | 0.88 | *0.67 – 1.1* | | 0.93 | *0.71 – 1.2* | |
| Continuous variable | 769/320 | 0.97 | *0.82 – 1.15* | *0.732* | 1.03 | *0.88 – 1.22* | *0.679* |

**Supplementary Table S33.** Survival of the cohort of 786 NB patients in the Cangelosi et al. *in silico* dataset, in relation to *FLJ37201* gene expression values, evaluated by the Cox model.

| | | Univariable analysis | | | Multivariable analysis | | |
|---|---|---|---|---|---|---|---|
| **Gene expression** | **N/O** | **HR** | **95%CI** | ***p*** | **HR** | **95%CI** | ***p*** |
| *Overall Survival* | | | | | | | |
| Median | | | | *0.171* | | | *0.019* |
| ≤ 1.418 (*reference*) | 393/123 | 1 | - | | 1 | - | |
| > 1.418 | 393/106 | 0.83 | *0.64 – 1.1* | | 0.73 | *0.56 – 0.95* | |
| Tertiles | | | | *0.306* | | | *0.063* |
| ≤ 0.887 (*reference*) | 262/90 | 1 | - | | 1 | - | |
| 0.887 – 1.956 | 262/59 | 0.61 | *0.44 – 0.85* | | 0.69 | *0.50 – 0.97* | |
| > 1.956 | 262/80 | 0.86 | *0.64 – 1.2* | | 0.75 | *0.55 – 1.0* | |
| Continuous variable | 786/229 | 0.98 | *0.89 – 1.06* | *0.580* | 0.93 | *0.86 – 1.01* | *0.107* |
| *Event Free Survival* | | | | | | | |
| Median | | | | *0.161* | | | *0.024* |
| ≤ 1.418 (*reference*) | 377/166 | 1 | - | | 1 | - | |
| > 1.418 | 392/154 | 0.85 | *0.69 – 1.1* | | 0.77 | *0.62 – 0.97* | |
| Tertiles | | | | *0.157* | | | *0.030* |
| ≤ 0.887 (*reference*) | 248/113 | 1 | - | | 1 | - | |
| 0.887 – 1.956 | 259/102 | 0.82 | *0.63 – 1.1* | | 0.85 | *0.65 – 1.1* | |
| > 1.956 | 262/105 | 0.82 | *0.63 – 1.1* | | 0.74 | *0.57 – 0.97* | |
| Continuous variable | 769/320 | 0.98 | *0.90 – 1.06* | *0.601* | 0.94 | *0.87 – 1.01* | *0.125* |

**Supplementary Table S34.** Survival of the cohort of 786 NB patients in the Cangelosi et al. *in silico* dataset, in relation to *GHITM* gene expression values, evaluated by the Cox model.

| | | Univariable analysis | | | Multivariable analysis | | |
|---|---|---|---|---|---|---|---|
| **Gene expression** | **N/O** | **HR** | **95%CI** | ***p*** | **HR** | **95%CI** | ***p*** |
| *Overall Survival* | | | | | | | |
| Median | | | | *0.310* | | | *0.270* |
| ≤ 4.571 (*reference*) | 392/120 | 1 | - | | 1 | - | |
| > 4.571 | 394/109 | 0.87 | *0.67 – 1.1* | | 1.2 | *0.89 – 1.5* | |
| Tertiles | | | | *0.929* | | | *0.165* |
| ≤ 4.435 (*reference*) | 262/81 | 1 | - | | 1 | - | |
| 4.435 – 4.726 | 262/67 | 0.78 | *0.56 – 1.1* | | 1.1 | *0.79 – 1.6* | |
| > 4.726 | 262/81 | 0.99 | *0.72 – 1.3* | | 1.3 | *0.91 – 1.7* | |
| Continuous variable | 786/229 | 0.79 | *0.55 – 1.13* | *0.191* | 1.03 | *0.73 – 1.5* | *0.859* |
| *Event Free Survival* | | | | | | | |
| Median | | | | *0.868* | | | *0.232* |
| ≤ 4.571 (*reference*) | 376/156 | 1 | - | | 1 | - | |
| > 4.571 | 393/164 | 1.0 | *0.82 – 1.3* | | 1.1 | *0.92 – 1.4* | |
| Tertiles | | | | *0.479* | | | *0.214* |
| ≤ 4.435 (*reference*) | 247/99 | 1 | - | | 1 | - | |
| 4.435 – 4.726 | 261/109 | 1.0 | *0.78 – 1.3* | | 1.2 | *0.92 – 1.6* | |
| > 4.726 | 261/112 | 1.1 | *0.84 – 1.4* | | 1.2 | *0.91 – 1.6* | |
| Continuous variable | 769/320 | 0.92 | *0.67 – 1.3* | *0.579* | 1.04 | *0.76 – 1.4* | *0.812* |

**Supplementary Table S35.** Survival of the cohort of 786 NB patients in the Cangelosi et al. *in silico* dataset, in relation to *GLUD1* gene expression values, evaluated by the Cox model.

| | | Univariable analysis | | | Multivariable analysis | | |
|---|---|---|---|---|---|---|---|
| **Gene expression** | **N/O** | **HR** | **95%CI** | ***p*** | **HR** | **95%CI** | ***p*** |
| *Overall Survival* | | | | | | | |
| Median | | | | *0.005* | | | *0.221* |
| ≤ 3.848 (*reference*) | 393/99 | 1 | - | | 1 | - | |
| > 3.848 | 393/130 | 1.4 | *1.1 – 1.9* | | 1.2 | *0.90 – 1.5* | |
| Tertiles | | | | *0.021* | | | *0.693* |
| ≤ 3.689 (*reference*) | 262/72 | 1 | - | | 1 | - | |
| 3.689 – 4.006 | 262/65 | 0.92 | *0.66 – 1.3* | | 0.97 | *0.69 – 1.4* | |
| > 4.006 | 262/92 | 1.4 | *1.0 – 1.9* | | 1.1 | *0.78 – 1.4* | |
| Continuous variable | 786/229 | 1.32 | *0.99 – 1.8* | *0.064* | 1.10 | *0.81 – 1.5* | *0.530* |
| *Event Free Survival* | | | | | | | |
| Median | | | | *< 0.001* | | | *0.004* |
| ≤ 3.848 (*reference*) | 377/131 | 1 | - | | 1 | - | |
| > 3.848 | 392/189 | 1.60 | *1.3 – 2.0* | | 1.4 | *1.1 – 1.7* | |
| Tertiles | | | | *< 0.001* | | | *0.022* |
| ≤ 3.689 (*reference*) | 246/88 | 1 | - | | 1 | - | |
| 3.689 – 4.006 | 261/97 | 1.1 | *0.83 – 1.5* | | 1.1 | *0.82 – 1.5* | |
| > 4.006 | 262/135 | 1.7 | *1.3 – 2.2* | | 1.4 | *1.0 – 1.8* | |
| Continuous variable | 769/320 | 1.7 | *1.3 – 2.2* | *< 0.001* | 1.5 | *1.2 – 2.0* | *0.002* |

**Supplementary Table S36.** Survival of the cohort of 786 NB patients in the Cangelosi et al. *in silico* dataset, in relation to *GRID1* gene expression values, evaluated by the Cox model.

| | | Univariable analysis | | | Multivariable analysis | | |
|---|---|---|---|---|---|---|---|
| **Gene expression** | **N/O** | **HR** | **95%CI** | ***p*** | **HR** | **95%CI** | ***p*** |
| *Overall Survival* | | | | | | | |
| Median | | | | *0.514* | | | *0.501* |
| ≤ 3.059 (*reference*) | 393/113 | 1 | - | | 1 | - | |
| > 3.059 | 393/116 | 1.1 | *0.84 – 1.4* | | 1.1 | *0.84 – 1.4* | |
| Tertiles | | | | *0.122* | | | *0.111* |
| ≤ 2.712 (*reference*) | 262/70 | 1 | - | | 1 | - | |
| 2.712 – 3.388 | 262/78 | 1.2 | *0.85 – 1.6* | | 1.1 | *0.80 – 1.6* | |
| > 3.388 | 262/81 | 1.3 | *0.93 – 1.8* | | 1.3 | *0.94 – 1.8* | |
| Continuous variable | 786/229 | 1.15 | *0.96 – 1.38* | *0.125* | 1.18 | *0.99 – 1.41* | *0.059* |
| *Event Free Survival* | | | | | | | |
| Median | | | | *0.873* | | | *0.993* |
| ≤ 3.059 (*reference*) | 388/165 | 1 | - | | 1 | - | |
| > 3.059 | 381/155 | 1.0 | *0.82 – 1.3* | | 1.0 | *0.80 – 1.3* | |
| Tertiles | | | | *0.329* | | | *0.458* |
| ≤ 2.712 (*reference*) | 259/109 | 1 | - | | 1 | - | |
| 2.712 – 3.388 | 259/103 | 0.98 | *0.75 – 1.3* | | 0.95 | *0.72 – 1.3* | |
| > 3.388 | 251/108 | 1.1 | *0.88 – 1.5* | | 1.1 | *0.84 – 1.5* | |
| Continuous variable | 769/320 | 1.03 | *0.89 – 1.2* | *0.699* | 1.04 | *0.90 – 1.2* | *0.597* |

**Supplementary Table S37.** Survival of the cohort of 786 NB patients in the Cangelosi et al. *in silico* dataset, in relation to *HTR7* gene expression values, evaluated by the Cox model.

| | | Univariable analysis | | | Multivariable analysis | | |
|---|---|---|---|---|---|---|---|
| **Gene expression** | **N/O** | **HR** | **95%CI** | ***p*** | **HR** | **95%CI** | ***p*** |
| *Overall Survival* | | | | | | | |
| Median | | | | *0.001* | | | *0.282* |
| ≤ 0.906 (*reference*) | 393/134 | 1 | - | | 1 | - | |
| > 0.906 | 393/95 | 0.65 | *0.50 – 0.85* | | 0.86 | *0.66 – 1.1* | |
| Tertiles | | | | *< 0.001* | | | *0.160* |
| ≤ 0.604 (*reference*) | 262/95 | 1 | - | | 1 | - | |
| 0.604 – 1.294 | 262/77 | 0.76 | *0.56 – 1.0* | | 0.86 | *0.63 – 1.2* | |
| > 1.294 | 262/57 | 0.53 | *0.38 – 0.74* | | 0.79 | *0.57 – 1.1* | |
| Continuous variable | 786/229 | 0.71 | *0.61 – 0.85* | *< 0.001* | 0.88 | *0.73 – 1.1* | *0.186* |
| *Event Free Survival* | | | | | | | |
| Median | | | | *0.219* | | | *0.853* |
| ≤ 0.906 (*reference*) | 381/165 | 1 | - | | 1 | - | |
| > 0.906 | 388/155 | 0.87 | *0.70 – 1.1* | | 1.0 | *0.82 – 1.3* | |
| Tertiles | | | | *0.045* | | | *0.783* |
| ≤ 0.604 (*reference*) | 255/113 | 1 | - | | 1 | - | |
| 0.604 – 1.294 | 253/110 | 0.93 | *0.72 – 1.2* | | 1.0 | *0.79 – 1.3* | |
| > 1.294 | 261/97 | 0.76 | *0.58 – 0.99* | | 0.96 | *0.73 – 1.3* | |
| Continuous variable | 769/320 | 0.85 | *0.74 – 0.97* | *0.015* | 0.97 | *0.84 – 1.1* | *0.703* |

**Supplementary Table S38.** Survival of the cohort of 786 NB patients in the Cangelosi et al. *in silico* dataset, in relation to *IFIT1* gene expression values, evaluated by the Cox model.

| | | Univariable analysis | | | Multivariable analysis | | |
|---|---|---|---|---|---|---|---|
| **Gene expression** | **N/O** | **HR** | **95%CI** | ***p*** | **HR** | **95%CI** | ***p*** |
| *Overall Survival* | | | | | | | |
| Median | | | | *0.615* | | | *0.858* |
| ≤ 2.074 (*reference*) | 393/111 | 1 | - | | 1 | - | |
| > 2.074 | 393/118 | 1.1 | *0.82 – 1.4* | | 0.98 | *0.75 – 1.3* | |
| Tertiles | | | | *0.939* | | | *0.733* |
| ≤ 1.638 (*reference*) | 262/82 | 1 | - | | 1 | - | |
| 1.638 – 2.675 | 262/63 | 0.72 | *0.52 – 1.0* | | 0.84 | *0.60 – 1.2* | |
| > 2.675 | 262/84 | 1.0 | *0.74 – 1.4* | | 0.95 | *0.70 – 1.3* | |
| Continuous variable | 786/229 | 0.98 | *0.89 – 1.08* | *0.661* | 0.96 | *0.88 – 1.06* | *0.442* |
| *Event Free Survival* | | | | | | | |
| Median | | | | *0.873* | | | *0.537* |
| ≤ 2.074 (*reference*) | 380/155 | 1 | - | | 1 | - | |
| > 2.074 | 389/165 | 1.0 | *0.82 – 1.3* | | 0.93 | *0.75 – 1.2* | |
| Tertiles | | | | *0.989* | | | *0.487* |
| ≤ 1.638 (*reference*) | 252/106 | 1 | - | | 1 | - | |
| 1.638 – 2.675 | 258/101 | 0.87 | *0.66 – 1.1* | | 0.94 | *0.71 – 1.2* | |
| > 2.675 | 259/113 | 1.0 | *0.77 – 1.3* | | 0.91 | *0.69 – 1.2* | |
| Continuous variable | 769/320 | 0.98 | *0.90 – 1.1* | *0.580* | 0.96 | *0.88 – 1.0* | *0.302* |

**Supplementary Table S39.** Survival of the cohort of 498 NB patients in the SEQC-498. *in silico* dataset, in relation to *IFIT1L* (alias *IFIT1B*) gene expression values, evaluated by the Cox model.

| | | Univariable analysis | | | Multivariable analysis | | |
|---|---|---|---|---|---|---|---|
| **Gene expression** | **N/O** | **HR** | **95%CI** | ***p*** | **HR** | **95%CI** | ***p*** |
| *Overall Survival* | | | | | | | |
| Median | | | | *0.359* | | | *0.127* |
| ≤ 1.074 (*reference*) | 249/48 | 1 | - | | 1 | - | |
| > 1.074 | 249/57 | 1.2 | *0.81 – 1.8* | | 1.4 | *0.92 – 2.0* | |
| Tertiles | | | | *0.590* | | | *0.223* |
| ≤ 1.048 (*reference*) | 163/35 | 1 | - | | 1 | - | |
| 1.048 – 1.106 | 168/30 | 0.78 | *0.48 – 1.3* | | 0.91 | *0.55 – 1.5* | |
| > 1.106 | 167/40 | 1.1 | *0.71 – 1.8* | | 1.3 | *0.84 – 2.1* | |
| Continuous variable | 498/105 | 3.29 | *1.05 – 10.3* | *0.069* | 7.79 | *2.29 – 26.5* | *0.005* |
| *Event Free Survival* | | | | | | | |
| Median | | | | *0.239* | | | *0.333* |
| ≤ 1.074 (*reference*) | 249/97 | 1 | - | | 1 | - | |
| > 1.074 | 249/86 | 0.84 | *0.63 – 1.1* | | 0.86 | *0.64 – 1.2* | |
| Tertiles | | | | *0.311* | | | *0.456* |
| ≤ 1.048 (*reference*) | 163/67 | 1 | - | | 1 | - | |
| 1.048 – 1.106 | 168/56 | 0.79 | *0.55 – 1.1* | | 0.87 | *0.61 – 1.2* | |
| > 1.106 | 167/60 | 0.84 | *0.59 – 1.2* | | 0.88 | *0.62 – 1.2* | |
| Continuous variable | 498/183 | 0.89 | *0.27 – 2.90* | *0.848* | 0.97 | *0.29 – 3.3* | *0.956* |

**Supplementary Table S40.** Survival of the cohort of 786 NB patients in the Cangelosi et al. *in silico* dataset, in relation to *IFIT3* gene expression values, evaluated by the Cox model.

| | | Univariable analysis | | | Multivariable analysis | | |
|---|---|---|---|---|---|---|---|
| **Gene expression** | **N/O** | **HR** | **95%CI** | ***p*** | **HR** | **95%CI** | ***p*** |
| *Overall Survival* | | | | | | | |
| Median | | | | *0.563* | | | *0.177* |
| ≤ 2.459 (*reference*) | 393/118 | 1 | - | | 1 | - | |
| > 2.459 | 393/111 | 0.93 | *0.71 – 1.2* | | 0.84 | *0.64 – 1.1* | |
| Tertiles | | | | *0.984* | | | *0.216* |
| ≤ 2.096 (*reference*) | 262/87 | 1 | - | | 1 | - | |
| 2.096 – 2.879 | 262/55 | 0.57 | *0.41 – 0.80* | | 0.68 | *0.48 – 0.95* | |
| > 2.879 | 262/87 | 1.0 | *0.74 – 1.3* | | 0.82 | *0.61 – 1.1* | |
| Continuous variable | 786/229 | 0.93 | *0.84 – 1.03* | *0.160* | 0.93 | *0.84 – 1.02* | *0.131* |
| *Event Free Survival* | | | | | | | |
| Median | | | | *0.442* | | | *0.840* |
| ≤ 2.459 (*reference*) | 381/151 | 1 | - | | 1 | - | |
| > 2.459 | 388/169 | 1.1 | *0.88 – 1.4* | | 0.98 | *0.78 – 1.2* | |
| Tertiles | | | | *0.424* | | | *0.708* |
| ≤ 2.096 (*reference*) | 252/103 | 1 | - | | 1 | - | |
| 2.096 – 2.879 | 258/100 | 0.92 | *0.70 – 1.2* | | 1.0 | *0.79 – 1.4* | |
| > 2.879 | 259/117 | 1.1 | *0.85 – 1.4* | | 0.95 | *0.72 – 1.2* | |
| Continuous variable | 769/320 | 0.96 | *0.88 – 1.1* | *0.416* | 0.95 | *0.87 – 1.0* | *0.290* |

**Supplementary Table S41.** Survival of the cohort of 786 NB patients in the Cangelosi et al. *in silico* dataset, in relation to *IFIT5* gene expression values, evaluated by the Cox model.

| | | Univariable analysis | | | Multivariable analysis | | |
|---|---|---|---|---|---|---|---|
| **Gene expression** | **N/O** | **HR** | **95%CI** | ***p*** | **HR** | **95%CI** | ***p*** |
| *Overall Survival* | | | | | | | |
| Median | | | | *< 0.001* | | | *0.480* |
| ≤ 3.253 (*reference*) | 393/83 | 1 | - | | 1 | - | |
| > 3.253 | 393/146 | 2.0 | *1.5 – 2.6* | | 1.1 | *0.83 – 1.5* | |
| Tertiles | | | | *< 0.001* | | | *0.758* |
| ≤ 3.031 (*reference*) | 261/54 | 1 | - | | 1 | - | |
| 3.031 – 3.473 | 263/76 | 1.5 | *1.0 – 2.1* | | 1.3 | *0.93 – 1.9* | |
| > 3.473 | 262/99 | 2.1 | *1.5 – 2.9* | | 0.99 | *0.70 – 1.4* | |
| Continuous variable | 786/229 | 1.47 | *1.21 – 1.79* | *< 0.001* | 0.92 | *0.74 – 1.1* | *0.479* |
| *Event Free Survival* | | | | | | | |
| Median | | | | *< 0.001* | | | *0.988* |
| ≤ 3.253 (*reference*) | 380/134 | 1 | - | | 1 | - | |
| > 3.253 | 389/186 | 1.5 | *1.2 – 1.9* | | 1.0 | *0.79 – 1.3* | |
| Tertiles | | | | *0.001* | | | *0.628* |
| ≤ 3.031 (*reference*) | 249/85 | 1 | - | | 1 | - | |
| 3.031 – 3.473 | 260/109 | 1.3 | *0.99 – 1.7* | | 1.2 | *0.88 – 1.6* | |
| > 3.473 | 260/126 | 1.6 | *1.2 – 2.1* | | 0.94 | *0.70 – 1.3* | |
| Continuous variable | 769/320 | 1.26 | *1.06 – 1.51* | *0.010* | 0.91 | *0.75 – 1.1* | *0.314* |

**Supplementary Table S42.** Survival of the cohort of 786 NB patients in the Cangelosi et al. *in silico* dataset, in relation to *KIF20B* gene expression values, evaluated by the Cox model.

| | | Univariable analysis | | | Multivariable analysis | | |
|---|---|---|---|---|---|---|---|
| **Gene expression** | **N/O** | **HR** | **95%CI** | ***p*** | **HR** | **95%CI** | ***p*** |
| *Overall Survival* | | | | | | | |
| Median | | | | *< 0.001* | | | *0.213* |
| ≤ 2.923 (*reference*) | 393/82 | 1 | - | | 1 | - | |
| > 2.923 | 393/147 | 2.1 | *1.6 – 2.7* | | 1.2 | *0.90 – 1.6* | |
| Tertiles | | | | *< 0.001* | | | *0.951* |
| ≤ 2.592 (*reference*) | 262/60 | 1 | - | | 1 | - | |
| 2.592 – 3.203 | 262/63 | 1.1 | *0.76 – 1.5* | | 0.87 | *0.61 – 1.3* | |
| > 3.203 | 262/106 | 2.0 | *1.5 – 2.8* | | 0.97 | *0.69 – 1.4* | |
| Continuous variable | 786/229 | 1.39 | *1.19 – 1.63* | *< 0.001* | 1.05 | *0.92 – 1.1* | *0.412* |
| *Event Free Survival* | | | | | | | |
| Median | | | | *< 0.001* | | | *< 0.001* |
| ≤ 2.923 (*reference*) | 377/111 | 1 | - | | 1 | - | |
| > 2.923 | 392/209 | 2.2 | *1.8 – 2.8* | | 1.6 | *1.2 – 2.0* | |
| Tertiles | | | | *< 0.001* | | | *0.006* |
| ≤ 2.592 (*reference*) | 246/77 | 1 | - | | 1 | - | |
| 2.592 – 3.203 | 261/93 | 1.2 | *0.89 – 1.6* | | 1.1 | *0.80 – 1.5* | |
| > 3.203 | 262/150 | 2.3 | *1.8 – 3.1* | | 1.5 | *1.1 – 2.0* | |
| Continuous variable | 769/320 | 1.60 | *1.39 – 1.8* | *< 0.001* | 1.29 | *1.12 – 1.48* | *< 0.001* |

**Supplementary Table S43.** Survival of the cohort of 498 NB patients in the SEQC-498 *in silico* dataset, in relation to *KILLIN* (alias *KLLN*) gene expression values, evaluated by the Cox model.

| | | Univariable analysis | | | Multivariable analysis | | |
|---|---|---|---|---|---|---|---|
| **Gene expression** | **N/O** | **HR** | **95%CI** | ***p*** | **HR** | **95%CI** | ***p*** |
| *Overall Survival* | | | | | | | |
| Median | | | | *0.003* | | | *0.753* |
| ≤ 3.272 (*reference*) | 249/63 | 1 | - | | 1 | - | |
| > 3.272 | 249/42 | 0.56 | *0.38 – 0.82* | | 0.93 | *0.60 – 1.5* | |
| Tertiles | | | | *0.001* | | | *0.989* |
| ≤ 2.915 (*reference*) | 166/47 | 1 | - | | 1 | - | |
| 2.915 – 3.618 | 166/29 | 0.48 | *0.30 – 0.77* | | 1.02 | *0.61 – 1.72* | |
| > 3.618 | 166/29 | 0.47 | *0.30 – 0.75* | | 1.00 | *0.59 – 1.7* | |
| Continuous variable | 498/105 | 0.86 | *0.69 – 1.1* | *0.144* | 1.09 | *0.93 – 1.27* | *0.309* |
| *Event Free Survival* | | | | | | | |
| Median | | | | *0.025* | | | *0.532* |
| ≤ 3.272 (*reference*) | 249/101 | 1 | - | | 1 | - | |
| > 3.272 | 249/82 | 0.72 | *0.54 – 0.96* | | 0.90 | *0.66 –1.2* | |
| Tertiles | | | | *0.007* | | | *0.402* |
| ≤ 2.915 (*reference*) | 166/72 | 1 | - | | 1 | - | |
| 2.915 – 3.618 | 166/57 | 0.69 | *0.49 – 0.98* | | 0.94 | *0.64 – 1.4* | |
| > 3.618 | 166/54 | 0.62 | *0.43 – 0.88* | | 0.84 | *0.57 – 1.3* | |
| Continuous variable | 498/183 | 0.89 | *0.76 – 1.05* | *0.149* | 1.00 | *0.87 – 1.14* | *0.972* |

**Supplementary Table S44.** Survival of the cohort of 786 NB patients in the Cangelosi et al. *in silico* dataset, in relation to *LDB3* gene expression values, evaluated by the Cox model.

| | | Univariable analysis | | | Multivariable analysis | | |
|---|---|---|---|---|---|---|---|
| **Gene expression** | **N/O** | **HR** | **95%CI** | ***p*** | **HR** | **95%CI** | ***p*** |
| *Overall Survival* | | | | | | | |
| Median | | | | *0.041* | | | *0.002* |
| ≤ 1.127 (*reference*) | 393/101 | 1 | - | | 1 | - | |
| > 1.127 | 393/128 | 1.3 | *1.0 – 1.7* | | 1.5 | *1.2 – 2.0* | |
| Tertiles | | | | *0.051* | | | *0.014* |
| ≤ 0.836 (*reference*) | 262/74 | 1 | - | | 1 | - | |
| 0.836 – 1.426 | 262/60 | 0.78 | *0.56 – 1.1* | | 1.0 | *0.72 – 1.4* | |
| > 1.426 | 262/95 | 1.3 | *0.98 – 1.8* | | 1.5 | *1.1 – 2.0* | |
| Continuous variable | 786/229 | 1.15 | *1.01 – 1.30* | *0.035* | 1.11 | *0.99 – 1.23* | *0.080* |
| *Event Free Survival* | | | | | | | |
| Median | | | | *0.420* | | | *0.086* |
| ≤ 1.127 (*reference*) | 393/157 | 1 | - | | 1 | - | |
| > 1.127 | 376/163 | 1.1 | *0.88 – 1.4* | | 1.2 | *0.97 – 1.5* | |
| Tertiles | | | | *0.572* | | | *0.268* |
| ≤ 0.836 (*reference*) | 262/116 | 1 | - | | 1 | - | |
| 0.836 – 1.426 | 262/86 | 0.68 | *0.51 – 0.90* | | 0.82 | *0.62 – 1.1* | |
| > 1.426 | 245/118 | 1.1 | *0.84 – 1.4* | | 1.2 | *0.90 – 1.5* | |
| Continuous variable | 769/320 | 1.10 | *0.98 – 1.23* | *0.106* | 1.08 | *0.97 – 1.19* | *0.168* |

**Supplementary Table S45.** Survival of the cohort of 786 NB patients in the Cangelosi et al. *in silico* dataset, in relation to *LIPA* gene expression values, evaluated by the Cox model.

| | | Univariable analysis | | | Multivariable analysis | | |
|---|---|---|---|---|---|---|---|
| **Gene expression** | **N/O** | **HR** | **95%CI** | ***p*** | **HR** | **95%CI** | ***p*** |
| *Overall Survival* | | | | | | | |
| Median | | | | *0.069* | | | *0.768* |
| ≤ 4.171 (*reference*) | 393/125 | 1 | - | | 1 | - | |
| > 4.171 | 393/104 | 0.79 | *0.61 – 1.0* | | 1.0 | *0.79 – 1.4* | |
| Tertiles | | | | *0.146* | | | *0.655* |
| ≤ 3.838 (*reference*) | 262/85 | 1 | - | | 1 | - | |
| 3.838 – 4.479 | 262/72 | 0.81 | *0.59 – 1.1* | | 0.88 | *0.64 – 1.2* | |
| > 4.479 | 262/72 | 0.79 | *0.58 – 1.1* | | 1.1 | *0.79 – 1.5* | |
| Continuous variable | 786/229 | 0.77 | *0.64 – 0.93* | *0.006* | 0.96 | *0.80 – 1.16* | *0.673* |
| *Event Free Survival* | | | | | | | |
| Median | | | | *0.532* | | | *0.505* |
| ≤ 4.171 (*reference*) | 377/160 | 1 | - | | 1 | - | |
| > 4.171 | 392/160 | 0.93 | *0.75 – 1.2* | | 1.1 | *0.86 – 1.4* | |
| Tertiles | | | | *0.795* | | | *0.353* |
| ≤ 3.838 (*reference*) | 247/107 | 1 | - | | 1 | - | |
| 3.838 – 4.479 | 261/101 | 0.88 | *0.67 – 1.2* | | 0.93 | *0.70 – 1.2* | |
| > 4.479 | 261/112 | 0.96 | *0.74 – 1.3* | | 1.1 | *0.87 – 1.5* | |
| Continuous variable | 769/320 | 0.92 | *0.78 – 1.1* | *0.280* | 1.0 | *0.89 – 1.2* | *0.566* |

**Supplementary Table S46.** Survival of the cohort of 498 NB patients in the SEQC-498 *in silico* dataset, in relation to *LIPF* gene expression, evaluated by the Cox model.

| | | Univariable analysis | | | Multivariable analysis | | |
|---|---|---|---|---|---|---|---|
| **Gene expression** | **N/O** | **HR** | **95%CI** | ***p*** | **HR** | **95%CI** | ***p*** |
| *Overall Survival* | | | | *0.089* | | | *0.750* |
| ≤ 1.01* (*reference*) | 287/67 | 1 | - | | 1 | - | |
| ≤ 1.01* | 211/38 | 0.71 | *0.48 – 1.1* | | 1.1 | *0.71 – 1.6* | |
| *Event Free Survival* | | | | *0.339* | | | *0.864* |
| ≤ 1.01* (*reference*) | 287/111 | 1 | - | | 1 | - | |
| > 1.01 | 211/72 | 0.87 | *0.64 – 1.2* | | 1.0 | *0.75 – 1.4* | |

* Allegedly corresponding to the absence of gene expression.

**Supplementary Table S47.** Association between *LIPF* gene expression and main patients' characteristics at diagnosis in a cohort of 498 NB patients in the SEQC-498 *in silico* dataset.

| | Gene expression ≤ 1.01* | | Gene expression > 1.01 | | ***p*** |
|---|---|---|---|---|---|
| **Patients characteristics** | N | % | N | % | |
| *MYCN* status | | | | | *< 0.001* |
| Not amplified | 216 | 53.9 | 185 | 46.1 | |
| Amplified | 69 | 75.0 | 23 | 25.0 | |
| Age | | | | | *0.102* |
| ≤ 18 months | 167 | 54.8 | 138 | 45.3 | |
| > 18 months | 120 | 62.2 | 73 | 37.8 | |
| Stage | | | | | *0.465* |
| Localized | 146 | 55.7 | 116 | 44.3 | |
| Stage 4 | 112 | 61.2 | 71 | 38.8 | |
| Stage 4S | 29 | 54.7 | 24 | 45.3 | |
| Total | 287 | 57.6 | 177 | 35.5 | |

* Allegedly corresponding to the absence of gene expression.

**Supplementary Table S48.** Survival of the cohort of 498 NB patients in the SEQC-498 *in silico* dataset, in relation to *LIPJ* gene expression values, evaluated by the Cox model.

| | | Univariable analysis | | | Multivariable analysis | | |
|---|---|---|---|---|---|---|---|
| **Gene expression** | **N/O** | **HR** | **95%CI** | ***p*** | **HR** | **95%CI** | ***p*** |
| *Overall Survival* | | | | | | | |
| Median | | | | *< 0.001* | | | *0.947* |
| ≤ 1.219 (*reference*) | 248/72 | 1 | - | | 1 | - | |
| > 1.219 | 250/33 | 0.36 | *0.24 – 0.55* | | 0.98 | *0.58 – 1.7* | |
| Tertiles | | | | *< 0.001* | | | *0.900* |
| ≤ 1.153 (*reference*) | 166/55 | 1 | - | | 1 | - | |
| 1.153 – 1.284 | 166/29 | 0.44 | *0.28 – 0.70* | | 1.3 | *0.78 – 2.2* | |
| > 1.284 | 166/21 | 0.30 | *0.18 – 0.49* | | 1.0 | *0.53 – 1.9* | |
| Continuous variable | 498/105 | 0.02 | *0.01 – 0.09* | *< 0.001* | 0.72 | *0.16 – 3.22* | *0.660* |
| *Event Free Survival* | | | | | | | |
| Median | | | | *< 0.001* | | | *0.811* |
| ≤ 1.219 (*reference*) | 248/111 | 1 | - | | 1 | - | |
| > 1.219 | 250/72 | 0.56 | *0.42 – 0.76* | | 0.96 | *0.67 – 1.4* | |
| Tertiles | | | | *< 0.001* | | | *0.486* |
| ≤ 1.153 (*reference*) | 166/81 | 1 | - | | 1 | - | |
| 1.153 – 1.284 | 166/57 | 0.62 | *0.44 – 0.87* | | 1.1 | *0.74 – 1.6* | |
| > 1.284 | 166/45 | 0.46 | *0.32 – 0.66* | | 0.86 | *0.55 – 1.3* | |
| Continuous variable | 498/183 | 0.11 | *0.04 – 0.30* | *< 0.001* | 0.60 | *0.21 – 1.74* | *0.342* |

**Supplementary Table S49.** Survival of the cohort of 498 NB patients in the SEQC-498 *in silico* dataset, in relation to *LIPM* gene expression, evaluated by the Cox model.

| | | Univariable analysis | | | Multivariable analysis | | |
|---|---|---|---|---|---|---|---|
| **Gene expression** | **N/O** | **HR** | **95%CI** | ***p*** | **HR** | **95%CI** | ***p*** |
| *Overall Survival* | | | | *0.166* | | | *0.479* |
| ≤ 1.01* (*reference)* | 309/70 | 1 | - | | 1 | - | |
| ≤ 1.01* | 189/35 | 0.75 | *0.50 – 1.1* | | 1.2 | *0.75 – 1.9* | |
| *Event Free Survival* | | | | *0.066* | | | *0.417* |
| ≤ 1.01* (*reference)* | 309/122 | 1 | - | | 1 | - | |
| > 1.01 | 189/61 | 0.75 | *0.55 – 1.0* | | 0.87 | *0.63 – 1.2* | |

* Allegedly corresponding to the absence of gene expression.

**Supplementary Table S50.** Association between *LIPM* gene expression and main patients characteristics at diagnosis in a cohort of 498 NB patients in the SEQC-498 *in silico* dataset.

| | Gene expression ≤ 1.01* | | Gene expression > 1.01 | | ***p*** |
|---|---|---|---|---|---|
| **Patients characteristics** | N | % | N | % | |
| *MYCN* status | | | | | *< 0.001* |
| Not amplified | 231 | 57.6 | 170 | 42.4 | |
| Amplified | 78 | 84.8 | 14 | 15.20 | |
| Age | | | | | *0.813* |
| ≤ 18 months | 188 | 61.6 | 117 | 38.4 | |
| > 18 months | 121 | 62.7 | 72 | 37.3 | |
| Stage | | | | | *0.588* |
| Localized | 157 | 59.9 | 105 | 40.1 | |
| Stage 4 | 118 | 64.5 | 65 | 35.5 | |
| Stage 4S | 34 | 64.2 | 19 | 35.8 | |
| Total | 309 | 62.1 | 189 | 37.1 | |

* Allegedly corresponding to the absence of gene expression.

**Supplementary Table S51.** Survival of the cohort of 498 NB patients in the SEQC-498 *in silico* dataset, in relation to *LIPN* gene expression, evaluated by the Cox model.

| | | Univariable analysis | | | Multivariable analysis | | |
|---|---|---|---|---|---|---|---|
| **Gene expression** | **N/O** | **HR** | **95%CI** | ***p*** | **HR** | **95%CI** | ***p*** |
| *Overall Survival* | | | | *0.131* | | | *0.044* |
| ≤ 1.01* (*reference)* | 336/64 | 1 | - | | 1 | - | |
| ≤ 1.01* | 162/41 | 1.4 | *0.91 – 2.0* | | 1.5 | *1.0 – 2.3* | |
| *Event Free Survival* | | | | *0.323* | | | *0.310* |
| ≤ 1.01* (*reference)* | 336/118 | 1 | - | | 1 | - | |
| > 1.01 | 162/65 | 1.2 | *0.86 – 1.6* | | 1.2 | *0.86 – 1.6* | |

* Allegedly corresponding to the absence of gene expression.

**Supplementary Table S52.** Association between *LIPN* gene expression and main patients characteristics at diagnosis in a cohort of 498 NB patients in the SEQC-498 *in silico* dataset.

| | Gene expression ≤ 1.01* | | Gene expression > 1.01 | | ***p*** |
|---|---|---|---|---|---|
| **Patients characteristics** | N | % | N | % | |
| *MYCN* status | | | | | *0.814* |
| Not amplified | 271 | 67.6 | 130 | 32.4 | |
| Amplified | 61 | 66.3 | 31 | 33.7 | |
| Age | | | | | *0.306* |
| ≤ 18 months | 211 | 69.2 | 94 | 30.8 | |
| > 18 months | 125 | 64.8 | 68 | 35.2 | |
| Stage | | | | | *0.695* |
| Localized | 181 | 69.1 | 81 | 30.9 | |
| Stage 4 | 121 | 66.1 | 62 | 33.9 | |
| Stage 4S | 34 | 64.2 | 19 | 35.8 | |
| Total | 336 | 67.5 | 162 | 32.5 | |

* Allegedly corresponding to the absence of gene expression.

**Supplementary Table S53.** Survival of the cohort of 498 NB patients in the SEQC-498. *in silico* dataset, in relation to *LOC219347(alias: TMEM254AS1)* gene expression values, evaluated by the Cox model.

| | | Univariable analysis | | | Multivariable analysis | | |
|---|---|---|---|---|---|---|---|
| **Gene expression** | **N/O** | **HR** | **95%CI** | ***p*** | **HR** | **95%CI** | ***p*** |
| *Overall Survival* | | | | | | | |
| Median | | | | *0.819* | | | *0.310* |
| ≤ 2.839 (*reference*) | 249/53 | 1 | - | | 1 | - | |
| > 2.839 | 249/52 | 0.96 | *0.65 – 1.4* | | 0.81 | *0.55 – 1.2* | |
| Tertiles | | | | *0.895* | | | *0.199* |
| ≤ 2.416 (*reference*) | 166/32 | 1 | - | | 1 | - | |
| 2.416 – 3.297 | 166/40 | 1.2 | *0.76 – 1.9* | | 0.98 | *0.61 – 1.6* | |
| > 3.297 | 166/33 | 0.97 | *0.60 – 1.6* | | 0.73 | *0.44 – 1.2* | |
| Continuous variable | 498/105 | 1.01 | *0.86 – 1.18* | *0.900* | 0.92 | *0.78 – 1.09* | *0.343* |
| *Event Free Survival* | | | | | | | |
| Median | | | | *0.738* | | | *0.398* |
| ≤ 2.839 (*reference*) | 249/92 | 1 | - | | 1 | - | |
| > 2.839 | 249/91 | 0.95 | *0.71 – 1.3* | | 0.88 | *0.65 – 1.2* | |
| Tertiles | | | | *0.551* | | | *0.136* |
| ≤ 2.416 (*reference*) | 166/60 | 1 | - | | 1 | - | |
| 2.416 – 3.297 | 166/64 | 1.0 | *0.73 – 1.5* | | 0.98 | *0.69 – 1.4* | |
| > 3.297 | 166/59 | 0.90 | *0.63 – 1.3* | | 0.75 | *0.52 – 1.1* | |
| Continuous variable | 498/183 | 1.03 | *0.91 – 1.16* | *0.652* | 0.97 | *0.85 – 1.1* | *0.592* |

**Supplementary Table S54.** Survival of the cohort of 498 NB patients in the SEQC-498 *in silico* dataset, in relation to *LOC439994* gene expression values, evaluated by the Cox model.

| | | Univariable analysis | | | Multivariable analysis | | |
|---|---|---|---|---|---|---|---|
| **Gene expression** | **N/O** | **HR** | **95%CI** | ***p*** | **HR** | **95%CI** | ***p*** |
| *Overall Survival* | | | | | | | |
| Median | | | | *0.917* | | | *0.909* |
| ≤ 8.121 (*reference*) | 249/52 | 1 | - | | 1 | - | |
| > 8.121 | 249/53 | 1.0 | *0.70 –1.5* | | 0.98 | *0.66 – 1.4* | |
| Tertiles | | | | *0.485* | | | *0.348* |
| ≤ 7.086 (*reference*) | 166/34 | 1 | - | | 1 | - | |
| 7.086 – 8.948 | 166/32 | 0.93 | *0.57 – 1.5* | | 0.96 | *0.59 – 1.6* | |
| > 8.948 | 166/39 | 1.2 | *0.74 – 1.9* | | 1.3 | *0.78 – 2.0* | |
| Continuous variable | 498/105 | 1.04 | *0.96 – 1.12* | *0.315* | 1.01 | *0.94 – 1.09* | *0.725* |
| *Event Free Survival* | | | | | | | |
| Median | | | | *0.877* | | | *0.878* |
| ≤ 8.121 (*reference*) | 249/91 | 1 | - | | 1 | - | |
| > 8.121 | 249/92 | 1.0 | *0.77 – 1.4* | | 1.0 | *0.76 – 1.4* | |
| Tertiles | | | | *0.679* | | | *0.457* |
| ≤ 7.086 (*reference*) | 166/60 | 1 | - | | 1 | - | |
| 7.086 – 8.948 | 166/60 | 0.97 | *0.68 – 1.4* | | 1.0 | *0.70 – 1.4* | |
| > 8.948 | 166/63 | 1.1 | *0.76 – 1.5* | | 1.1 | *0.80 – 1.6* | |
| Continuous variable | 498/183 | 1.01 | *0.95 – 1.1* | *0.675* | 1.0 | *0.95 – 1.1* | *0.811* |

**Supplementary Table S55.** Survival of the cohort of 498 NB patients in the SEQC-498 *in silico* dataset, in relation to *LOC642361* gene expression values, evaluated by the Cox model.

| | | Univariable analysis | | | Multivariable analysis | | |
|---|---|---|---|---|---|---|---|
| **Gene expression** | **N/O** | **HR** | **95%CI** | ***p*** | **HR** | **95%CI** | ***p*** |
| *Overall Survival* | | | | | | | |
| Median | | | | *0.064* | | | *0.451* |
| ≤ 8.493 (*reference*) | 249/45 | 1 | - | | 1 | - | |
| > 8.493 | 249/60 | 1.4 | *0.98 – 2.1* | | 1.2 | *0.78 – 1.7* | |
| Tertiles | | | | *0.063* | | | *0.860* |
| ≤ 7.584 (*reference*) | 166/32 | 1 | - | | 1 | - | |
| 7.584 – 9.351 | 165/28 | 0.87 | *0.52 – 1.4* | | 0.92 | *0.55 – 1.5* | |
| > 9.351 | 167/45 | 1.5 | *0.96 – 2.4* | | 0.95 | *0.60 – 1.5* | |
| Continuous variable | 498/105 | 1.13 | *1.04 – 1.22* | *0.003* | 1.02 | *0.94 – 1.09* | *0.690* |
| *Event Free Survival* | | | | | | | |
| Median | | | | *0.024* | | | *0.247* |
| ≤ 8.493 (*reference*) | 249/79 | 1 | - | | 1 | - | |
| > 8.493 | 249/104 | 1.4 | *1.0 – 1.9* | | 1.2 | *0.88 – 1.6* | |
| Tertiles | | | | *0.005* | | | *0.385* |
| ≤ 7.584 (*reference*) | 166/51 | 1 | - | | 1 | - | |
| 7.584 – 9.351 | 165/56 | 1.1 | *0.76 – 1.6* | | 1.1 | *0.75 – 1.6* | |
| > 9.351 | 167/76 | 1.6 | *1.2 – 2.3* | | 1.2 | *0.81 – 1.7* | |
| Continuous variable | 498/183 | 1.09 | *1.03 – 1.16* | *0.004* | 1.02 | *0.96 – 1.09* | *0.431* |

**Supplementary Table S56.** Survival of the cohort of 498 NB patients in the SEQC-498 *in silico* dataset, in relation to *LOC650623* gene expression values, evaluated by the Cox model.

| | | Univariable analysis | | | Multivariable analysis | | |
|---|---|---|---|---|---|---|---|
| **Gene expression** | **N/O** | **HR** | **95%CI** | ***p*** | **HR** | **95%CI** | ***p*** |
| *Overall Survival* | | | | | | | |
| Median | | | | *0.520* | | | *0.207* |
| ≤ 11.615 (*reference*) | 249/56 | 1 | - | | 1 | - | |
| > 11.615 | 249/49 | 0.88 | *0.60 – 1.3* | | 0.78 | *0.53 – 1.1* | |
| Tertiles | | | | *< 0.001* | | | *0.347* |
| ≤ 10.052 (*reference*) | 166/42 | 1 | - | | 1 | - | |
| 10.052 – 13.799 | 165/27 | 0.62 | *0.38 – 1.0* | | 0.83 | *0.51 – 1.4* | |
| > 13.799 | 167/36 | 0.87 | *0.56 – 1.4* | | 0.81 | *0.51 – 1.3* | |
| Continuous variable | 498/105 | 0.985 | *0.946 –1.03* | *0.455* | 0.98 | *0.95 – 1.02* | *0.341* |
| *Event Free Survival* | | | | | | | |
| Median | | | | *0.808* | | | *0.244* |
| ≤ 11.615 (*reference*) | 249/93 | 1 | - | | 1 | - | |
| > 11.615 | 249/90 | 0.96 | *0.72 – 1.3* | | 0.84 | *0.62 – 1.1* | |
| Tertiles | | | | *0.818* | | | *0.698* |
| ≤ 10.052 (*reference*) | 166/62 | 1 | - | | 1 | - | |
| 10.052 – 13.799 | 165/57 | 0.90 | *0.63 – 1.3* | | 1.0 | *0.70 – 1.5* | |
| > 13.799 | 167/64 | 1.0 | *0.73 – 1.5* | | 0.93 | *0.65 – 1.3* | |
| Continuous variable | 498/183 | 1.00 | *0.97 – 1.02* | *0.831* | 0.99 | *0.96 – 1.02* | *0.370* |

**Supplementary Table S57.** Survival of the cohort of 498 NB patients in the SEQC-498 *in silico* dataset, in relation to *LOC728190* gene expression values, evaluated by the Cox model.

| | | Univariable analysis | | | Multivariable analysis | | |
|---|---|---|---|---|---|---|---|
| **Gene expression** | **N/O** | **HR** | **95%CI** | ***p*** | **HR** | **95%CI** | ***p*** |
| *Overall Survival* | | | | | | | |
| Median | | | | *0.002* | | | *0.085* |
| ≤ 7.325 (*reference*) | 248/39 | 1 | - | | 1 | - | |
| > 7.325 | 250/66 | 1.8 | *1.2 – 2.7* | | 1.4 | *0.95 – 2.1* | |
| Tertiles | | | | *0.008* | | | *0.124* |
| ≤ 6.601 (*reference*) | 166/28 | 1 | - | | 1 | - | |
| 6.601 – 8.152 | 166/30 | 1.1 | *0.66 – 1.8* | | 1.1 | *0.64 – 1.8* | |
| > 8.152 | 166/47 | 1.8 | *1.1 – 2.9* | | 1.4 | *0.89 – 2.3* | |
| Continuous variable | 498/105 | 1.14 | *1.03 – 1.25* | *0.011* | 1.08 | *0.98 – 1.19* | *0.132* |
| *Event Free Survival* | | | | | | | |
| Median | | | | *0.005* | | | *0.083* |
| ≤ 7.325 (*reference*) | 248/77 | 1 | - | | 1 | - | |
| > 7.325 | 250/106 | 1.5 | *1.1 – 2.0* | | 1.3 | *0.96 – 1.8* | |
| Tertiles | | | | *0.018* | | | *0.098* |
| ≤ 6.601 (*reference*) | 166/51 | 1 | - | | 1 | - | |
| 6.601 – 8.152 | 166/62 | 1.3 | *0.88 – 1.8* | | 1.2 | *0.84 – 1.8* | |
| > 8.152 | 166/70 | 1.5 | *1.1 – 2.2* | | 1.4 | *0.94 – 2.0* | |
| Continuous variable | 498/183 | 1.07 | *1.00 – 1.16* | *0.066* | 1.05 | *0.97 – 1.13* | *0.229* |

**Supplementary Table S58.** Survival of the cohort of 498 NB patients in the SEQC-498 *in silico* dataset, in relation to *LRIT1* gene expression, evaluated by the Cox model.

| | | Univariable analysis | | | Multivariable analysis | | |
|---|---|---|---|---|---|---|---|
| **Gene expression** | **N/O** | **HR** | **95%CI** | ***p*** | **HR** | **95%CI** | ***p*** |
| *Overall Survival* | | | | *0.858* | | | *0.729* |
| ≤ 1.01* (*reference)* | 397/84 | 1 | - | | 1 | - | |
| ≤ 1.01* | 101/21 | 0.96 | *0.59 – 1.5* | | 1.1 | *0.67 – 1.8* | |
| *Event Free Survival* | | | | *0.658* | | | *0.226* |
| ≤ 1.01* (*reference)* | 397/144 | 1 | - | | 1 | - | |
| > 1.01 | 101/39 | 1.1 | *0.76 – 1.5* | | 1.3 | *0.88 – 1.8* | |

* Allegedly corresponding to the absence of gene expression.

**Supplementary Table S59.** Association between *LRIT1* gene expression and main patients characteristics at diagnosis in a cohort of 498 NB patients in the SEQC-498 *in silico* dataset.

| | Gene expression ≤ 1.01* | | Gene expression > 1.01 | | ***p*** |
|---|---|---|---|---|---|
| **Patients characteristics** | N | % | N | % | |
| *MYCN* status | | | | | *0.180* |
| Not amplified | 315 | 78.6 | 86 | 21.5 | |
| Amplified | 78 | 84.8 | 14 | 15.2 | |
| Age | | | | | *0.624* |
| ≤ 18 months | 241 | 79.0 | 64 | 21.0 | |
| > 18 months | 156 | 80.8 | 37 | 19.2 | |
| Stage | | | | | *0.260* |
| Localized | 206 | 78.6 | 56 | 21.4 | |
| Stage 4 | 152 | 83.1 | 31 | 16.9 | |
| Stage 4S | 39 | 73.6 | 14 | 26.4 | |
| Total | 397 | 79.7 | 101 | 20.3 | |

* Allegedly corresponding to the absence of gene expression.

**Supplementary Table S60.** Survival of the cohort of 498 NB patients in the SEQC-498. *in silico* dataset, in relation to *LRIT2* gene expression values, evaluated by the Cox model.

| | | Univariable analysis | | | Multivariable analysis | | |
|---|---|---|---|---|---|---|---|
| **Gene expression** | **N/O** | **HR** | **95%CI** | ***p*** | **HR** | **95%CI** | ***p*** |
| *Overall Survival* | | | | | | | |
| Median | | | | *0.021* | | | *0.915* |
| ≤ 1.236 (*reference*) | 249/62 | 1 | - | | 1 | - | |
| > 1.236 | 249/43 | 0.63 | *0.43 – 0.94* | | 1.0 | *0.69 – 1.5* | |
| Tertiles | | | | *0.059* | | | *0.961* |
| ≤ 1.148 (*reference*) | 165/40 | 1 | - | | 1 | - | |
| 1.148 – 1.383 | 167/38 | 0.90 | *0.58 – 1.4* | | 1.3 | *0.85 – 2.1* | |
| > 1.383 | 166/27 | 0.62 | *0.38 – 1.0* | | 0.98 | *0.60 – 1.6* | |
| Continuous variable | 498/105 | 0.46 | *0.24 – 0.88* | *0.009* | 0.67 | *0.38 – 1.2* | *0.141* |
| *Event Free Survival* | | | | | | | |
| Median | | | | *0.006* | | | *0.416* |
| ≤ 1.236 (*reference*) | 249/106 | 1 | - | | 1 | - | |
| > 1.236 | 249/77 | 0.67 | *0.50 – 0.89* | | 0.88 | *0.65 – 1.2* | |
| Tertiles | | | | *0.019* | | | *0.394* |
| ≤ 1.148 (*reference*) | 165/70 | 1 | - | | 1 | - | |
| 1.148 – 1.383 | 167/62 | 0.83 | *0.59 – 1.2* | | 1.0 | *0.71 – 1.4* | |
| > 1.383 | 166/51 | 0.65 | *0.45 – 0.93* | | 0.85 | *0.59 – 1.2* | |
| Continuous variable | 498/183 | 0.59 | *0.38 – 0.93* | *0.014* | 0.74 | *0.49 – 1.12* | *0.147* |

**Supplementary Table S61.** Survival of the cohort of 498 NB patients in the SEQC-498. *in silico* dataset, in relation to *MAT1A* gene expression values, evaluated by the Cox model.

| | | Univariable analysis | | | Multivariable analysis | | |
|---|---|---|---|---|---|---|---|
| **Gene expression** | **N/O** | **HR** | **95%CI** | ***p*** | **HR** | **95%CI** | ***p*** |
| *Overall Survival* | | | | | | | |
| Median | | | | *0.006* | | | *0.731* |
| ≤ 1.066 (*reference*) | 249/40 | 1 | - | | 1 | - | |
| > 1.066 | 249/65 | 1.7 | *1.2 – 2.6* | | 1.1 | *0.72 – 1.6* | |
| Tertiles | | | | *< 0.001* | | | *0.516* |
| ≤ 1.040 (*reference*) | 166/23 | 1 | - | | 1 | - | |
| 1.040 – 1.104 | 166/34 | 1.5 | *0.90 – 2.6* | | 1.2 | *0.68 – 2.0* | |
| > 1.104 | 166/48 | 2.3 | *1.4 – 3.7* | | 1.2 | *0.72 – 2.0* | |
| Continuous variable | 498/105 | 0.99 | *0.96 – 1.02* | *0.135* | 0.99 | *0.97 – 1.02* | *0.579* |
| *Event Free Survival* | | | | | | | |
| Median | | | | *0.010* | | | *0.518* |
| ≤ 1.066 (*reference*) | 249/77 | 1 | - | | 1 | - | |
| > 1.066 | 249/106 | 1.5 | *1.1 – 2.0* | | 1.1 | *0.82 – 1.5* | |
| Tertiles | | | | *0.021* | | | *0.796* |
| ≤ 1.040 (*reference*) | 166/54 | 1 | - | | 1 | - | |
| 1.040 – 1.104 | 166/53 | 0.97 | *0.66 – 1.4* | | 0.81 | *0.55 –1.2* | |
| > 1.104 | 166/76 | 1.5 | *1.1 – 2.1* | | 1.0 | *0.72 – 1.5* | |
| Continuous variable | 498/183 | 1.00 | *0.99 – 1.003* | *0.964* | 1.002 | *0.999 – 1.01* | *0.229* |

**Supplementary Table S62.** Survival of the cohort of 498 NB patients in the SEQC-498 *in silico* dataset, in relation to *MBL1P* gene expression values, evaluated by the Cox model.

| | | Univariable analysis | | | Multivariable analysis | | |
|---|---|---|---|---|---|---|---|
| **Gene expression** | **N/O** | **HR** | **95%CI** | ***p*** | **HR** | **95%CI** | ***p*** |
| *Overall Survival* | | | | | | | |
| Median | | | | *0.041* | | | *0.675* |
| ≤ 1.743 (*reference*) | 249/61 | 1 | - | | 1 | - | |
| > 1.743 | 249/44 | 0.67 | *0.45 – 0.99* | | 1.1 | *0.68 – 1.8* | |
| Tertiles | | | | *0.013* | | | *0.968* |
| ≤ 1.414 (*reference*) | 165/36 | 1 | - | | 1 | - | |
| 1.414 – 2.233 | 167/50 | 1.4 | *0.90 – 2.1* | | 1.6 | *1.0 – 2.5* | |
| > 2.233 | 166/19 | 0.47 | *0.27 – 0.82* | | 0.83 | *0.44 – 1.6* | |
| Continuous variable | 498/105 | 0.71 | *0.56 – 0.91* | *0.003* | 0.91 | *0.69 – 1.2* | *0.489* |
| *Event Free Survival* | | | | | | | |
| Median | | | | *0.078* | | | *0.410* |
| ≤ 1.743 (*reference*) | 249/99 | 1 | - | | 1 | - | |
| > 1.743 | 249/84 | 0.77 | *0.58 – 1.0* | | 0.87 | *0.61 – 1.2* | |
| Tertiles | | | | *0.037* | | | *0.307* |
| ≤ 1.414 (*reference*) | 165/63 | 1 | - | | 1 | - | |
| 1.414 – 2.233 | 167/72 | 1.1 | *0.78 – 1.5* | | 1.1 | *0.76 – 1.5* | |
| > 2.233 | 166/48 | 0.67 | *0.46 – 0.97* | | 0.79 | *0.51 – 1.2* | |
| Continuous variable | 498/183 | 0.83 | *0.70 – 0.98* | *0.025* | 0.90 | *0.75 – 1.1* | *0.287* |

**Supplementary Table S63.** Survival of the cohort of 786 NB patients in the Cangelosi et al. *in silico* dataset, in relation to *MINPP1* gene expression values, evaluated by the Cox model.

| | | Univariable analysis | | | Multivariable analysis | | |
|---|---|---|---|---|---|---|---|
| **Gene expression** | **N/O** | **HR** | **95%CI** | ***p*** | **HR** | **95%CI** | ***p*** |
| *Overall Survival* | | | | | | | |
| Median | | | | *< 0.001* | | | *0.992* |
| ≤ 2.989 (*reference*) | 393/91 | 1 | - | | 1 | - | |
| > 2.989 | 393/138 | 1.7 | *1.3 – 2.2* | | 1.0 | *0.76 – 1.3* | |
| Tertiles | | | | *< 0.001* | | | *0.297* |
| ≤ 2.813 (*reference*) | 262/58 | 1 | - | | 1 | - | |
| 2.813 – 3.169 | 262/61 | 1.0 | *0.72 – 1.5* | | 0.76 | *0.53 – 1.1* | |
| > 3.169 | 262/110 | 2.2 | *1.6 – 3.0* | | 1.1 | *0.81 – 1.6* | |
| Continuous variable | 786/229 | 1.97 | *1.47 – 2.65* | *< 0.001* | 1.13 | *0.87 – 1.46* | *0.354* |
| *Event Free Survival* | | | | | | | |
| Median | | | | *< 0.001* | | | *0.235* |
| ≤ 2.989 (*reference*) | 376/128 | 1 | - | | 1 | - | |
| > 2.989 | 393/192 | 1.6 | *1.3 – 2.0* | | 1.1 | *0.91 – 1.4* | |
| Tertiles | | | | *< 0.001* | | | *0.015* |
| ≤ 2.813 (*reference*) | 246/74 | 1 | - | | 1 | - | |
| 2.813 – 3.169 | 261/105 | 1.4 | *1.0 – 1.9* | | 1.1 | *0.84 – 1.5* | |
| > 3.169 | 262/141 | 2.2 | *1.7 – 2.9* | | 1.4 | *1.1 – 1.9* | |
| Continuous variable | 769/320 | 2.15 | *1.66 – 2.79* | *< 0.001* | 1.42 | *1.09 – 1.83* | *0.007* |

**Supplementary Table S64.** Survival of the cohort of 786 NB patients in the Cangelosi et al. *in silico* dataset, in relation to *MMRN2* gene expression values, evaluated by the Cox model.

| | | Univariable analysis | | | Multivariable analysis | | |
|---|---|---|---|---|---|---|---|
| **Gene expression** | **N/O** | **HR** | **95%CI** | ***p*** | **HR** | **95%CI** | ***p*** |
| *Overall Survival* | | | | | | | |
| Median | | | | *0.007* | | | *0.583* |
| ≤ 2.863 (*reference*) | 393/131 | 1 | - | | 1 | - | |
| > 2.863 | 393/98 | 0.70 | *0.54 – 0.91* | | 0.93 | *0.71 – 1.2* | |
| Tertiles | | | | *0.006* | | | *0.269* |
| ≤ 2.525 (*reference*) | 262/93 | 1 | - | | 1 | - | |
| 2.525 – 3.187 | 262/70 | 0.71 | *0.52 – 0.97* | | 1.0 | *0.74 – 1.4* | |
| > 3.187 | 262/66 | 0.65 | *0.47 – 0.89* | | 0.83 | *0.60 – 1.1* | |
| Continuous variable | 786/229 | 0.82 | *0.69 – 0.96* | *0.017* | 1.02 | *0.88 – 1.18* | *0.795* |
| *Event Free Survival* | | | | | | | |
| Median | | | | *0.044* | | | *0.688* |
| ≤ 2.863 (*reference*) | 393/176 | 1 | - | | 1 | - | |
| > 2.863 | 376/144 | 0.80 | *0.64 – 0.99* | | 0.95 | *0.76 – 1.2* | |
| Tertiles | | | | *0.022* | | | *0.351* |
| ≤ 2.525 (*reference*) | 262/126 | 1 | - | | 1 | - | |
| 2.525 – 3.187 | 260/96 | 0.70 | *0.53 – 0.91* | | 0.89 | *0.68 – 1.2* | |
| > 3.187 | 247/98 | 0.74 | *0.57 – 0.96* | | 0.88 | *0.67 –1.2* | |
| Continuous variable | 769/320 | 0.87 | *0.75 – 1.00* | *0.054* | 1.00 | *0.87 – 1.1* | *0.972* |

**Supplementary Table S65.** Survival of the cohort of 786 NB patients in the Cangelosi et al. *in silico* dataset, in relation to *NRG3* gene expression values, evaluated by the Cox model.

| | | Univariable analysis | | | Multivariable analysis | | |
|---|---|---|---|---|---|---|---|
| **Gene expression** | **N/O** | **HR** | **95%CI** | ***p*** | **HR** | **95%CI** | ***p*** |
| *Overall Survival* | | | | | | | |
| Median | | | | *0.002* | | | *0.026* |
| ≤ 1.714 (*reference*) | 393/135 | 1 | - | | 1 | - | |
| > 1.714 | 393/94 | 0.66 | *0.51– 0.86* | | 0.74 | *0.56 – 0.97* | |
| Tertiles | | | | *0.018* | | | *0.126* |
| ≤ 1.097 (*reference*) | 262/92 | 1 | - | | 1 | - | |
| 1.097 – 2.249 | 262/70 | 0.73 | *0.53 – 0.99* | | 0.83 | *0.61 – 1.1* | |
| > 2.249 | 262/67 | 0.69 | *0.50 – 0.94* | | 0.78 | *0.57 – 1.1* | |
| Continuous variable | 786/229 | 0.93 | *0.86 – 1.00* | *0.078* | 0.96 | *0.89 – 1.03* | *0.299* |
| *Event Free Survival* | | | | | | | |
| Median | | | | *< 0.001* | | | *0.007* |
| ≤ 1.714 (*reference*) | 389/187 | 1 | - | | 1 | - | |
| > 1.714 | 380/133 | 0.67 | *0.54 – 0.84* | | 0.73 | *0.58 – 0.92* | |
| Tertiles | | | | *0.002* | | | *0.017* |
| ≤ 1.097 (*reference*) | 259/129 | 1 | - | | 1 | - | |
| 1.097 – 2.249 | 257/100 | 0.72 | *0.56 – 0.94* | | 0.78 | *0.60 – 1.0* | |
| > 2.249 | 253/91 | 0.66 | *0.50 – 0.86* | | 0.72 | *0.55 – 0.95* | |
| Continuous variable | 769/320 | 0.93 | *0.87 – 0.99* | *0.025* | 0.95 | *0.90 – 1.02* | *0.145* |

**Supplementary Table S66.** Survival of the cohort of 498 NB patients in the SEQC-498 *in silico* dataset, in relation to *OPN4* gene expression values, evaluated by the Cox model.

| | | Univariable analysis | | | Multivariable analysis | | |
|---|---|---|---|---|---|---|---|
| **Gene expression** | **N/O** | **HR** | **95%CI** | ***p*** | **HR** | **95%CI** | ***p*** |
| *Overall Survival* | | | | | | | |
| Median | | | | *0.578* | | | *0.764* |
| ≤ 1.062 (*reference*) | 248/54 | 1 | - | | 1 | - | |
| > 1.062 | 250/51 | 0.90 | *0.61 – 1.3* | | 0.94 | *0.64 –1.4* | |
| Tertiles | | | | *0.244* | | | *0.441* |
| ≤ 1.036 (*reference*) | 165/40 | 1 | - | | 1 | - | |
| 1.036 – 1.107 | 167/32 | 0.73 | *0.46 – 1.2* | | 0.68 | *0.42 – 1.1* | |
| > 1.107 | 166/33 | 0.76 | *0.48 – 1.2* | | 0.84 | *0.52 – 1.3* | |
| Continuous variable | 498/105 | 1.45 | *0.54 – 3.94* | *0.483* | 1.52 | *0.54 – 4.26* | *0.450* |
| *Event Free Survival* | | | | | | | |
| Median | | | | *0.360* | | | *0.586* |
| ≤ 1.062 (*reference*) | 248/95 | 1 | - | | 1 | - | |
| > 1.062 | 250/88 | 0.87 | *0.65 – 1.2* | | 0.92 | *0.68 – 1.2* | |
| Tertiles | | | | *0.111* | | | *0.288* |
| ≤ 1.036 (*reference*) | 165/68 | 1 | - | | 1 | - | |
| 1.036 – 1.107 | 167/58 | 0.76 | *0.54 – 1.1* | | 0.75 | *0.53 – 1.1* | |
| > 1.107 | 166/57 | 0.75 | *0.53 – 1.1* | | 0.83 | *0.58 – 1.2* | |
| Continuous variable | 498/183 | 1.13 | *0.49 – 2.59* | *0.782* | 1.21 | *0.52 – 2.83* | *0.671* |

**Supplementary Table S67.** Survival of the cohort of 786 NB patients in the Cangelosi et al. *in silico* dataset, in relation to *PANK1* gene expression values, evaluated by the Cox model.

| | | Univariable analysis | | | Multivariable analysis | | |
|---|---|---|---|---|---|---|---|
| **Gene expression** | **N/O** | **HR** | **95%CI** | ***p*** | **HR** | **95%CI** | ***p*** |
| *Overall Survival* | | | | | | | |
| Median | | | | *< 0.001* | | | *0.312* |
| ≤ 2.773 (*reference*) | 393/88 | 1 | - | | 1 | - | |
| > 2.773 | 393/141 | 1.8 | *1.4 – 2.4* | | 1.2 | *0.87 – 1.5* | |
| Tertiles | | | | *< 0.001* | | | *0.096* |
| ≤ 2.516 (*reference*) | 262/61 | 1 | - | | 1 | - | |
| 2.516 – 3.026 | 262/60 | 0.99 | *0.69 – 1.4* | | 0.88 | *0.61 – 1.3* | |
| > 3.026 | 262/108 | 2.1 | *1.5 – 2.9* | | 1.3 | *0.92 – 1.8* | |
| Continuous variable | 786/229 | 1.57 | *1.29 – 1.93* | *< 0.001* | 1.12 | *0.90 – 1.38* | *0.327* |
| *Event Free Survival* | | | | | | | |
| Median | | | | *< 0.001* | | | *0.009* |
| ≤ 2.773 (*reference*) | 380/122 | 1 | - | | 1 | - | |
| > 2.773 | 389/198 | 1.8 | *1.5 – 2.3* | | 1.4 | *1.1 – 1.7* | |
| Tertiles | | | | *< 0.001* | | | *< 0.001* |
| ≤ 2.516 (*reference*) | 251/79 | 1 | - | | 1 | - | |
| 2.516 – 3.026 | 259/97 | 1.3 | *0.95 – 1.7* | | 1.2 | *0.86 – 1.6* | |
| > 3.026 | 259/144 | 2.3 | *1.7 – 3.0* | | 1.6 | *1.2 – 2.2* | |
| Continuous variable | 769/320 | 1.61 | *1.35 – 1.91* | *< 0.001* | 1.30 | *1.08 – 1.57* | *0.006* |

**Supplementary Table S68.** Survival of the cohort of 786 NB patients in the Cangelosi et al. *in silico* dataset, in relation to *PCDH21* (alias *CDHR1*) gene expression values, evaluated by the Cox model.

| | | Univariable analysis | | | Multivariable analysis | | |
|---|---|---|---|---|---|---|---|
| **Gene expression** | **N/O** | **HR** | **95%CI** | ***p*** | **HR** | **95%CI** | ***p*** |
| *Overall Survival* | | | | | | | |
| Median | | | | *0.222* | | | *0.590* |
| ≤ 1.923 (*reference*) | 393/123 | 1 | - | | 1 | - | |
| > 1.923 | 393/106 | 0.85 | *0.66 – 1.1* | | 1.1 | *0.83 – 1.4* | |
| Tertiles | | | | *0.463* | | | *0.211* |
| ≤ 1.376 (*reference*) | 262/82 | 1 | - | | 1 | - | |
| 1.376 – 2.383 | 262/73 | 0.87 | *0.64 – 1.2* | | 1.1 | *0.77 – 1.5* | |
| > 2.383 | 262/74 | 0.89 | *0.65 – 1.2* | | 1.2 | *0.89 – 1.7* | |
| Continuous variable | 786/229 | 0.92 | *0.84 – 1.01* | *0.079* | 1.01 | *0.92 – 1.11* | *0.825* |
| *Event Free Survival* | | | | | | | |
| Median | | | | *0.030* | | | *0.406* |
| ≤ 1.923 (*reference*) | 387/176 | 1 | - | | 1 | - | |
| > 1.923 | 382/144 | 0.78 | *0.63 – 0.98* | | 0.91 | *0.73 – 1.1* | |
| Tertiles | | | | *0.058* | | | *0.775* |
| ≤ 1.376 (*reference*) | 260/113 | 1 | - | | 1 | - | |
| 1.376 – 2.383 | 255/116 | 1.0 | *0.81 – 1.4* | | 1.2 | *0.91 – 1.5* | |
| > 2.383 | 254/91 | 0.76 | *0.58 – 1.0* | | 0.95 | *0.72 – 1.3* | |
| Continuous variable | 769/320 | 0.93 | *0.86 – 1.00* | *0.054* | 0.99 | *0.91 – 1.07* | *0.732* |

**Supplementary Table S69.** Survival of the cohort of 786 NB patients in the Cangelosi et al. *in silico* dataset, in relation to *PLAC9* gene expression values, evaluated by the Cox model.

| | | Univariable analysis | | | Multivariable analysis | | |
|---|---|---|---|---|---|---|---|
| **Gene expression** | **N/O** | **HR** | **95%CI** | ***p*** | **HR** | **95%CI** | ***p*** |
| *Overall Survival* | | | | | | | |
| Median | | | | *0.526* | | | *0.646* |
| ≤ 1.501 (*reference*) | 393/118 | 1 | - | | 1 | - | |
| > 1.501 | 393/111 | 0.92 | *0.71 – 1.2* | | 1.1 | *0.82 – 1.4* | |
| Tertiles | | | | *0.288* | | | *0.646* |
| ≤ 1.118 (*reference*) | 262/82 | 1 | - | | 1 | - | |
| 1.118 – 1.897 | 262/75 | 0.90 | *0.66 – 1.2* | | 0.97 | *0.71 – 1.3* | |
| > 1.897 | 262/72 | 0.84 | *0.61 – 1.2* | | 0.93 | *0.68 – 1.3* | |
| Continuous variable | 786/229 | 0.96 | *0.85 – 1.10* | *0.592* | 1.00 | *0.88 – 1.12* | *0.974* |
| *Event Free Survival* | | | | | | | |
| Median | | | | *0.280* | | | *0.892* |
| ≤ 1.501 (*reference*) | 393/170 | 1 | - | | 1 | - | |
| > 1.501 | 376/150 | 0.89 | *0.71 – 1.1* | | 0.98 | *0.79 – 1.2* | |
| Tertiles | | | | *0.123* | | | *0.358* |
| ≤ 1.118 (*reference*) | 262/120 | 1 | - | | 1 | - | |
| 1.118 – 1.897 | 262/103 | 0.81 | *0.62 – 1.1* | | 0.90 | *0.69 – 1.2* | |
| > 1.897 | 245/97 | 0.81 | *0.62 – 1.1* | | 0.88 | *0.68 – 1.2* | |
| Continuous variable | 769/320 | 0.97 | *0.86 – 1.09* | *0.610* | 0.99 | *0.89 – 1.11* | *0.901* |

**Supplementary Table S70.** Survival of the cohort of 786 NB patients in the Cangelosi et al. *in silico* dataset, in relation to *PTEN* gene expression values, evaluated by the Cox model.

| | | Univariable analysis | | | Multivariable analysis | | |
|---|---|---|---|---|---|---|---|
| **Gene expression** | **N/O** | **HR** | **95%CI** | ***p*** | **HR** | **95%CI** | ***p*** |
| *Overall Survival* | | | | | | | |
| Median | | | | *0.009* | | | *0.190* |
| ≤ 4.383 (*reference*) | 393/130 | 1 | - | | 1 | - | |
| > 4.383 | 393/99 | 0.71 | *0.54 – 0.92* | | 0.84 | *0.64 – 1.1* | |
| Tertiles | | | | *0.004* | | | *0.048* |
| ≤ 4.233 (*reference*) | 262/101 | 1 | - | | 1 | - | |
| 4.233 – 4.552 | 262/54 | 0.46 | *0.33 – 0.64* | | 0.62 | *0.44 – 0.87* | |
| > 4.552 | 262/74 | 0.65 | *0.48 – 0.88* | | 0.75 | *0.55 – 1.0* | |
| Continuous variable | 786/229 | 0.66 | *0.51 – 0.85* | *0.003* | 0.86 | *0.70 – 1.06* | *0.164* |
| *Event Free Survival* | | | | | | | |
| Median | | | | *0.257* | | | *0.702* |
| ≤ 4.383 (*reference*) | 379/164 | 1 | - | | 1 | - | |
| > 4.383 | 390/156 | 0.88 | *0.71 – 1.1* | | 0.96 | *0.77 – 1.2* | |
| Tertiles | | | | *0.474* | | | *0.702* |
| ≤ 4.233 (*reference*) | 248/113 | 1 | - | | 1 | - | |
| 4.233 – 4.552 | 261/94 | 0.72 | *0.55 – 0.95* | | 0.84 | *0.64 – 1.1* | |
| > 4.552 | 260/113 | 0.91 | *0.70 – 1.2* | | 0.95 | *0.73 – 1.2* | |
| Continuous variable | 769/320 | 0.87 | *0.68 – 1.12* | *0.293* | 0.98 | *0.80 – 1.2* | *0.832* |

**Supplementary Table S71.** Survival of the cohort of 709 NB patients in the E-MTAB-1781 *in silico* dataset, in relation to *RGR* gene expression values, evaluated by the Cox model.

| | | Univariable analysis | | | Multivariable analysis | | |
|---|---|---|---|---|---|---|---|
| **Gene expression** | **N/O** | **HR** | **95%CI** | ***p*** | **HR** | **95%CI** | ***p*** |
| *Overall Survival* | | | | | | | |
| Median | | | | *0.031* | | | *0.554* |
| ≤ -5.690 (*reference*) | 353/90 | 1 | - | | 1 | - | |
| > -5.690 | 356/71 | 0.71 | *0.52 – 0.97* | | 0.91 | *0.66 – 1.2* | |
| Tertiles | | | | *0.082* | | | *0.554* |
| ≤ -6.745 (*reference*) | 236/59 | 1 | - | | 1 | - | |
| -6.745 – -2.613 | 236/54 | 0.84 | *0.58 – 1.2* | | 1.0 | *0.69 – 1.5* | |
| > -2.613 | 237/48 | 0.71 | *0.49 – 1.0* | | 0.89 | *0.60 – 1.3* | |
| Continuous variable | 709/161 | 0.94 | *0.89 – 1.00* | *0.049* | 0.97 | *0.92 – 1.03* | *0.410* |
| *Event Free Survival* | | | | | | | |
| Median | | | | *0.012* | | | *0.206* |
| ≤ -5.690 (*reference*) | 349/144 | 1 | - | | 1 | - | |
| > -5.690 | 346/112 | 0.73 | *0.57 – 0.93* | | 0.85 | *0.66 –1.1* | |
| Tertiles | | | | *0.193* | | | *0.503* |
| ≤ -6.745 (*reference*) | 232/90 | 1 | - | | 1 | - | |
| -6.745 – -2.613 | 235/89 | 0.94 | *0.70 –1.3* | | 1.1 | *0.79 – 1.4* | |
| > -2.613 | 228/77 | 0.82 | *0.60 – 1.1* | | 0.90 | *0.66 – 1.2* | |
| Continuous variable | 695/256 | 0.95 | *0.91 – 1.0* | *0.051* | 0.97 | *0.93 – 1.0* | *0.201* |

**Supplementary Table S72.** Survival of the cohort of 786 NB patients in the Cangelosi et al. *in silico* dataset, in relation to *RNLS* gene expression values, evaluated by the Cox model.

| | | Univariable analysis | | | Multivariable analysis | | |
|---|---|---|---|---|---|---|---|
| **Gene expression** | **N/O** | **HR** | **95%CI** | ***p*** | **HR** | **95%CI** | ***p*** |
| *Overall Survival* | | | | | | | |
| Median | | | | *0.519* | | | *0.083* |
| ≤ 1.653 (*reference*) | 393/120 | 1 | - | | 1 | - | |
| > 1.653 | 393/109 | 0.92 | *0.71 – 1.2* | | 1.3 | *0.97 – 1.7* | |
| Tertiles | | | | *0.445* | | | *0.065* |
| ≤ 1.428 (*reference*) | 262/91 | 1 | - | | 1 | - | |
| 1.428 – 1.843 | 262/56 | 0.56 | *0.40 – 0.79* | | 1.0 | *0.70 – 1.4* | |
| > 1.843 | 262/82 | 0.90 | *0.67 – 1.2* | | 1.3 | *0.98 – 1.8* | |
| Continuous variable | 786/229 | 0.84 | *0.73 – 0.98* | *0.033* | 1.14 | *0.98 – 1.34* | *0.082* |
| *Event Free Survival* | | | | | | | |
| Median | | | | *0.901* | | | *0.137* |
| ≤ 1.653 (*reference*) | 382/160 | 1 | - | | 1 | - | |
| > 1.653 | 387/160 | 1.0 | *0.81 – 1.3* | | 1.2 | *0.95 – 1.5* | |
| Tertiles | | | | *0.902* | | | *0.094* |
| ≤ 1.428 (*reference*) | 256/114 | 1 | - | | 1 | - | |
| 1.428 – 1.843 | 255/92 | 0.78 | *0.59 – 1.0* | | 1.1 | *0.82 – 1.5* | |
| > 1.843 | 258/114 | 1.0 | *0.78 – 1.3* | | 1.3 | *0.96 –1.7* | |
| Continuous variable | 769/320 | 0.93 | *0.80 – 1.1* | *0.342* | 1.1 | *0.98 – 1.3* | *0.081* |

**Supplementary Table S73.** Survival of the cohort of 786 NB patients in the Cangelosi et al. *in silico* dataset, in relation to *RPP30* gene expression values, evaluated by the Cox model.

| | | Univariable analysis | | | Multivariable analysis | | |
|---|---|---|---|---|---|---|---|
| **Gene expression** | **N/O** | **HR** | **95%CI** | ***p*** | **HR** | **95%CI** | ***p*** |
| *Overall Survival* | | | | | | | |
| Median | | | | *0.021* | | | *0.782* |
| ≤ 3.175 (*reference*) | 393/101 | 1 | - | | 1 | - | |
| > 3.175 | 393/128 | 1.4 | *1.0 – 1.8* | | 0.96 | *0.74 – 1.3* | |
| Tertiles | | | | *0.028* | | | *0.356* |
| ≤ 3.017 (*reference*) | 262/70 | 1 | - | | 1 | - | |
| 3.017 – 3.346 | 262/68 | 0.98 | *0.70 – 1.4* | | 1.1 | *0.79 – 1.6* | |
| > 3.346 | 262/91 | 1.4 | *1.0 – 1.9* | | 0.87 | *0.63 – 1.2* | |
| Continuous variable | 786/229 | 1.48 | *1.07 – 2.05* | *0.019* | 0.87 | *0.65 – 1.15* | *0.327* |
| *Event Free Survival* | | | | | | | |
| Median | | | | *0.018* | | | *0.854* |
| ≤ 3.175 (*reference*) | 387/145 | 1 | - | | 1 | - | |
| > 3.175 | 382/175 | 1.3 | *1.0 – 1.6* | | 1.0 | *0.81 – 1.3* | |
| Tertiles | | | | *0.037* | | | *0.696* |
| ≤ 3.017 (*reference*) | 257/93 | 1 | - | | 1 | - | |
| 3.017 – 3.346 | 259/114 | 1.3 | *0.98 – 1.7* | | 1.3 | *1.0 – 1.8* | |
| > 3.346 | 253/113 | 1.3 | *1.0 – 1.8* | | 0.95 | *0.72 – 1.3* | |
| Continuous variable | 769/320 | 1.41 | *1.07– 1.84* | *0.015* | 0.96 | *0.74 – 1.3* | *0.777* |

**Supplementary Table S74.** Survival of the cohort of 498 NB patients in the SEQC-498 *in silico* dataset, in relation to *SFTPA1* gene expression, evaluated by the Cox model.

| | | **Univariable analysis** | | | **Multivariable analysis** | | |
|---|---|---|---|---|---|---|---|
| **Gene expression** | **N/O** | **HR** | **95%CI** | ***p*** | **HR** | **95%CI** | ***p*** |
| *Overall Survival* | | | | *0.782* | | | *0.890* |
| ≤ 1.01* (*reference)* | 335/71 | 1 | - | | 1 | - | |
| ≤ 1.01* | 163/341 | 0.94 | *0.63 – 1.4* | | 1.0 | *0.68 – 1.6* | |
| *Event Free Survival* | | | | *0.710* | | | *0.417* |
| ≤ 1.01* (*reference)* | 335/120 | 1 | - | | 1 | - | |
| > 1.01 | 163/63 | 1.1 | *0.78 – 1.4* | | 1.1 | *0.83 – 1.6* | |

* Allegedly corresponding to the absence of gene expression.

**Supplementary Table S75.** Association between *SFTPA1* gene expression and main patients characteristics at diagnosis in a cohort of 498 NB patients in the SEQC-498 *in silico* dataset.

| | Gene expression ≤ 1.01* | | Gene expression > 1.01 | | ***p*** |
|---|---|---|---|---|---|
| **Patients characteristics** | N | % | N | % | |
| *MYCN* status | | | | | *0.006* |
| Not amplified | 259 | 64.6 | 142 | 35.4 | |
| Amplified | 73 | 79.4 | 19 | 20.7 | |
| Age | | | | | *0.670* |
| ≤ 18 months | 203 | 66.6 | 102 | 33.4 | |
| > 18 months | 132 | 68.4 | 61 | 31.6 | |
| Stage | | | | | *0.977* |
| Localized | 177 | 67.6 | 85 | 32.4 | |
| Stage 4 | 123 | 67.2 | 60 | 32.8 | |
| Stage 4S | 35 | 66.0 | 18 | 34.0 | |
| Total | 335 | 67.3 | 163 | 32.73 | |

* Allegedly corresponding to the absence of gene expression.

**Supplementary Table S76.** Survival of the cohort of 498 NB patients in the SEQC-498 *in silico* dataset, in relation to *SFTPA2* gene expression values, evaluated by the Cox model.

| | | Univariable analysis | | | Multivariable analysis | | |
|---|---|---|---|---|---|---|---|
| **Gene expression** | **N/O** | **HR** | **95%CI** | ***p*** | **HR** | **95%CI** | ***p*** |
| *Overall Survival* | | | | | | | |
| Median | | | | *< 0.001* | | | *0.575* |
| ≤ 1.086 (*reference*) | 248/66 | 1 | - | | 1 | - | |
| > 1.086 | 250/39 | 0.52 | *0.35 – 0.77* | | 0.88 | *0.57 – 1.4* | |
| Tertiles | | | | *0.007* | | | *0.318* |
| ≤ 1.053 (*reference*) | 164/42 | 1 | - | | 1 | - | |
| 1.053 – 1.122 | 168/38 | 0.82 | *0.53 – 1.3* | | 0.90 | *0.57 – 1.4* | |
| > 1.122 | 166/25 | 0.51 | *0.31 – 0.83* | | 0.77 | *0.45 – 1.3* | |
| Continuous variable | 498/105 | 0.06 | *0.01 – 0.40* | *< 0.001* | 0.33 | *0.05 – 2.06* | *0.196* |
| *Event Free Survival* | | | | | | | |
| Median | | | | *0.033* | | | *0.761* |
| ≤ 1.086 (*reference*) | 248/101 | 1 | - | | 1 | - | |
| > 1.086 | 250/82 | 0.73 | *0.54 – 0.98* | | 0.95 | *0.69 – 1.3* | |
| Tertiles | | | | *0.013* | | | *0.119* |
| ≤ 1.053 (*reference*) | 164/67 | 1 | - | | 1 | - | |
| 1.053 – 1.122 | 168/67 | 0.90 | *0.64 – 1.3* | | 0.91 | *0.64 – 1.3* | |
| > 1.122 | 166/49 | 0.63 | *0.43 – 0.91* | | 0.73 | *0.50 – 1.1* | |
| Continuous variable | 498/183 | 0.24 | *0.07 – 0.77* | *0.008* | 0.43 | *0.13 – 1.43* | *0.142* |

**Supplementary Table S77.** Survival of the cohort of 709 NB patients in the E-MTAB-1781 *in silico* dataset, in relation to *SFTPD* gene expression values, evaluated by the Cox model.

| | | Univariable analysis | | | Multivariable analysis | | |
|---|---|---|---|---|---|---|---|
| **Gene expression** | **N/O** | **HR** | **95%CI** | ***p*** | **HR** | **95%CI** | ***p*** |
| *Overall Survival* | | | | | | | |
| Median | | | | *0.010* | | | *0.495* |
| ≤ -0.519 (*reference*) | 354/66 | 1 | - | | 1 | - | |
| > -0.519 | 355/95 | 1.5 | *1.1 – 2.1* | | 1.1 | *0.81 – 1.5* | |
| Tertiles | | | | *0.009* | | | *0.369* |
| ≤ -0.854 (*reference*) | 236/36 | 1 | - | | 1 | - | |
| -0.854 – -0.205 | 236/64 | 1.9 | *1.2 – 2.8* | | 1.1 | *0.74 – 1.7* | |
| > -0.205 | 237/61 | 1.8 | *1.2 – 2.7* | | 1.2 | *0.79 – 1.9* | |
| Continuous variable | 709/161 | 1.36 | *1.11 – 1.66* | *0.002* | 1.19 | *0.95 – 1.5* | *0.119* |
| *Event Free Survival* | | | | | | | |
| Median | | | | *0.256* | | | *0.475* |
| ≤ -0.519 (*reference*) | 353/120 | 1 | - | | 1 | - | |
| > -0.519 | 342/136 | 1.2 | *0.90 – 1.5* | | 0.91 | *0.71 – 1.2* | |
| Tertiles | | | | *0.257* | | | *0.460* |
| ≤ -0.854 (*reference*) | 235/75 | 1 | - | | 1 | - | |
| -0.854 – -0.205 | 235/93 | 1.2 | *0.91 – 1.7* | | 0.91 | *0.66 – 1.2* | |
| > -0.205 | 225/88 | 1.2 | *0.88 – 1.6* | | 0.88 | *0.64 – 1.2* | |
| Continuous variable | 695/256 | 1.12 | *0.96 – 1.31* | *0.124* | 0.96 | *0.83 – 1.12* | *0.646* |

**Supplementary Table S78.** Survival of the cohort of 498 NB patients in the SEQC-498 *in silico* dataset, in relation to *SH2D4B* gene expression values, evaluated by the Cox model.

| | | Univariable analysis | | | Multivariable analysis | | |
|---|---|---|---|---|---|---|---|
| **Gene expression** | **N/O** | **HR** | **95%CI** | ***p*** | **HR** | **95%CI** | ***p*** |
| *Overall Survival* | | | | | | | |
| Median | | | | *0.959* | | | *0.667* |
| ≤ 1.091 (*reference*) | 248/52 | 1 | - | | 1 | - | |
| > 1.091 | 250/53 | 0.99 | *0.68 – 1.5* | | 0.92 | *0.62 – 1.4* | |
| Tertiles | | | | *0.755* | | | *0.243* |
| ≤ 1.064 (*reference*) | 165/37 | 1 | - | | 1 | - | |
| 1.064 – 1.125 | 165/31 | 0.78 | *0.49 – 1.3* | | 1.0 | *0.62 – 1.6* | |
| > 1.125 | 168/37 | 0.93 | *0.59 – 1.5* | | 0.76 | *0.48 – 1.2* | |
| Continuous variable | 498/105 | 2.03 | *0.36 – 11.60* | *0.440* | 0.56 | *0.09 – 3.40* | *0.519* |
| *Event Free Survival* | | | | | | | |
| Median | | | | *0.694* | | | *0.780* |
| ≤ 1.091 (*reference*) | 248/87 | 1 | - | | 1 | - | |
| > 1.091 | 250/96 | 1.1 | *0.79 – 1.4* | | 0.96 | *0.71 – 1.3* | |
| Tertiles | | | | *0.275* | | | *0.931* |
| ≤ 1.064 (*reference*) | 165/55 | 1 | - | | 1 | - | |
| 1.064 – 1.125 | 165/57 | 0.97 | *0.67 – 1.4* | | 1.1 | *0.76 – 1.6* | |
| > 1.125 | 168/71 | 1.2 | *0.85 – 1.7* | | 0.99 | *0.69 – 1.4* | |
| Continuous variable | 498/183 | 3.50 | *1.04 – 11.8* | *0.057* | 1.02 | *0.28 – 3.81* | *0.972* |

**Supplementary Table S79.** Survival of the cohort of 498 NB patients in the SEQC-498 *in silico* dataset, in relation to *SLC16A12* gene expression values, evaluated by the Cox model.

| | | Univariable analysis | | | Multivariable analysis | | |
|---|---|---|---|---|---|---|---|
| **Gene expression** | **N/O** | **HR** | **95%CI** | ***p*** | **HR** | **95%CI** | ***p*** |
| *Overall Survival* | | | | | | | |
| Median | | | | *< 0.001* | | | *0.836* |
| ≤ 2.070 (*reference*) | 249/71 | 1 | - | | 1 | - | |
| > 2.070 | 249/34 | 0.42 | *0.28 – 0.63* | | 1.0 | *0.67 – 1.6* | |
| Tertiles | | | | *< 0.001* | | | *0.861* |
| ≤ 1.559 (*reference*) | 166/60 | 1 | - | | 1 | - | |
| 1.559 – 2.847 | 166/26 | 0.36 | *0.22 – 0.56* | | 0.96 | *0.60 – 1.5* | |
| > 2.847 | 166/19 | 0.25 | *0.15 – 0.42* | | 0.96 | *0.53 – 1.7* | |
| Continuous variable | 498/105 | 0.84 | *0.75 – 0.94* | *< 0.001* | 1.01 | *0.93 – 1.09* | *0.887* |
| *Event Free Survival* | | | | | | | |
| Median | | | | *< 0.001* | | | *0.518* |
| ≤ 2.070 (*reference*) | 249/113 | 1 | - | | 1 | - | |
| > 2.070 | 249/70 | 0.54 | *0.40 – 0.73* | | 0.90 | *0.65 – 1.2* | |
| Tertiles | | | | *< 0.001* | | | *0.418* |
| ≤ 1.559 (*reference*) | 166/86 | 1 | - | | 1 | - | |
| 1.559 – 2.847 | 166/58 | 0.59 | *0.42 – 0.82* | | 1.1 | *0.79 – 1.6* | |
| > 2.847 | 166/39 | 0.37 | *0.25 – 0.54* | | 0.81 | *0.53 – 1.2* | |
| Continuous variable | 498/183 | 0.87 | *0.81 – 0.94* | *< 0001* | 0.96 | *0.90 – 1.02* | *0.209* |

**Supplementary Table S80.** Survival of the cohort of 786 NB patients in the Cangelosi et al. *in silico* dataset, in relation to *SNCG* gene expression values, evaluated by the Cox model.

| | | Univariable analysis | | | Multivariable analysis | | |
|---|---|---|---|---|---|---|---|
| **Gene expression** | **N/O** | **HR** | **95%CI** | ***p*** | **HR** | **95%CI** | ***p*** |
| *Overall Survival* | | | | | | | |
| Median | | | | *0.008* | | | *0.868* |
| ≤ 1.774 (*reference*) | 393/130 | 1 | - | | 1 | - | |
| > 1.774 | 393/99 | 0.70 | *0.54 – 0.92* | | 1.0 | *0.77 – 1.4* | |
| Tertiles | | | | *0.004* | | | *0.568* |
| ≤ 1.294 (*reference*) | 262/98 | 1 | - | | 1 | - | |
| 1.294 – 2.297 | 262/61 | 0.57 | *0.41 – 0.78* | | 0.91 | *0.65 – 1.3* | |
| > 2.297 | 262/70 | 0.65 | *0.48 – 0.88* | | 1.1 | *0.80 – 1.6* | |
| Continuous variable | 786/229 | 0.82 | *0.73 – 0.92* | *< 0.001* | 1.0 | *0.89 – 1.1* | *0.972* |
| *Event Free Survival* | | | | | | | |
| Median | | | | *< 0.001* | | | *0.182* |
| ≤ 1.774 (*reference*) | 393/186 | 1 | - | | 1 | - | |
| > 1.774 | 376/134 | 0.69 | *0.55 – 0.86* | | 0.85 | *0.67 – 1.1* | |
| Tertiles | | | | *< 0.001* | | | *0.165* |
| ≤ 1.294 (*reference*) | 262/131 | 1 | - | | 1 | - | |
| 1.294 – 2.297 | 262/106 | 0.76 | *0.59 – 0.98* | | 1.0 | *0.76 – 1.3* | |
| > 2.297 | 245/83 | 0.60 | *0.45 – 0.78* | | 0.80 | *0.59 – 1.1* | |
| Continuous variable | 769/320 | 0.79 | *0.72 – 0.88* | *< 0.001* | 0.89 | *0.80 – 0.98* | *0.022* |

**Supplementary Table S81.** Survival of the cohort of 786 NB patients in the Cangelosi et al. *in silico* dataset, in relation to *STAMBPL1* gene expression values, evaluated by the Cox model.

| | | Univariable analysis | | | Multivariable analysis | | |
|---|---|---|---|---|---|---|---|
| **Gene expression** | **N/O** | **HR** | **95%CI** | ***p*** | **HR** | **95%CI** | ***p*** |
| *Overall Survival* | | | | | | | |
| Median | | | | *< 0.001* | | | *0.444* |
| ≤ 2.844 (*reference*) | 393/91 | 1 | - | | 1 | - | |
| > 2.844 | 393/138 | 1.7 | *1.3 – 2.2* | | 1.1 | *0.84 – 1.5* | |
| Tertiles | | | | *< 0.001* | | | *0.690* |
| ≤ 2.662 (*reference*) | 262/61 | 1 | - | | 1 | - | |
| 2.662 – 3.029 | 262/74 | 1.3 | *0.91 – 1.8* | | 1.1 | *0.76 – 1.5* | |
| > 3.029 | 262/94 | 1.8 | *1.3 – 2.5* | | 1.1 | *0.75 – 1.5* | |
| Continuous variable | 786/229 | 1.91 | *1.46 – 2.50* | *< 0.001* | 1.1 | *0.87 – 1.4* | *0.388* |
| *Event Free Survival* | | | | | | | |
| Median | | | | *0.003* | | | *0.305* |
| ≤ 2.844 (*reference*) | 376/138 | 1 | - | | 1 | - | |
| > 2.844 | 393/182 | 1.4 | *1.1 – 1.8* | | 1.1 | *0.89 – 1.4* | |
| Tertiles | | | | *0.005* | | | *0.495* |
| ≤ 2.662 (*reference*) | 246/91 | 1 | - | | 1 | - | |
| 2.662 – 3.029 | 261/104 | 1.1 | *0.84 – 1.5* | | 1.0 | *0.75 – 1.3* | |
| > 3.029 | 262/125 | 1.5 | *1.1 – 1.9* | | 1.1 | *0.82 – 1.5* | |
| Continuous variable | 769/320 | 1.61 | *1.28– 2.03* | *< 0.001* | 1.19 | *0.94 – 1.49* | *0.138* |

**Supplementary Table S82.** Survival of the cohort of 786 NB patients in the Cangelosi et al. *in silico* dataset, in relation to *TSPAN14* gene expression values, evaluated by the Cox model.

| | | Univariable analysis | | | Multivariable analysis | | |
|---|---|---|---|---|---|---|---|
| **Gene expression** | **N/O** | **HR** | **95%CI** | ***p*** | **HR** | **95%CI** | ***p*** |
| *Overall Survival* | | | | | | | |
| Median | | | | *< 0.001* | | | *0.003* |
| ≤ 3.486 (*reference*) | 393/90 | 1 | - | | 1 | - | |
| > 3.486 | 393/139 | 1.7 | *1.3 – 2.2* | | 1.5 | *1.1 – 2.0* | |
| Tertiles | | | | *< 0.001* | | | *0.025* |
| ≤ 3.303 (*reference*) | 262/60 | 1 | - | | 1 | - | |
| 3.303 – 3.687 | 262/66 | 1.1 | *0.79 – 1.6* | | 1.5 | *1.1 – 2.2* | |
| > 3.687 | 262/103 | 1.9 | *1.4 – 2.6* | | 1.5 | *1.1 – 2.0* | |
| Continuous variable | 786/229 | 1.59 | *1.24 – 2.04* | *< 0.001* | 1.2 | *0.98 – 1.6* | *0.069* |
| *Event Free Survival* | | | | | | | |
| Median | | | | *< 0.001* | | | *0.002* |
| ≤ 3.486 (*reference*) | 393/136 | 1 | - | | 1 | - | |
| > 3.486 | 376/184 | 1.6 | *1.3 – 2.0* | | 1.4 | *1.1 – 1.8* | |
| Tertiles | | | | *< 0.001* | | | *0.010* |
| ≤ 3.303 (*reference*) | 262/88 | 1 | - | | 1 | - | |
| 3.303 – 3.687 | 261/100 | 1.2 | *0.89 – 1.6* | | 1.4 | *1.0 – 1.8* | |
| > 3.687 | 246/132 | 1.8 | *1.4 – 2.3* | | 1.4 | *1.1 – 1.9* | |
| Continuous variable | 769/320 | 1.67 | *1.33 – 2.09* | *< 0.001* | 1.38 | *1.11 – 1.71* | *0.004* |

**Supplementary Table S83.** Survival of the cohort of 498 NB patients in the SEQC-498 *in silico* dataset, in relation to *WAPAL* gene expression values, evaluated by the Cox model.

| | | Univariable analysis | | | Multivariable analysis | | |
|---|---|---|---|---|---|---|---|
| **Gene expression** | **N/O** | **HR** | **95%CI** | ***p*** | **HR** | **95%CI** | ***p*** |
| *Overall Survival* | | | | | | | |
| Median | | | | *0.001* | | | *0.073* |
| ≤ 49.436 (*reference*) | 249/37 | 1 | - | | 1 | - | |
| > 49.436 | 249/68 | 1.9 | *1.3 – 2.9* | | 1.4 | *0.96 –2.2* | |
| Tertiles | | | | *< 0.001* | | | *0.027* |
| ≤ 44.612 (*reference*) | 166/22 | 1 | - | | 1 | - | |
| 44.612 – 54.125 | 166/29 | 1.3 | *0.75 – 2.3* | | 1.4 | *0.79 – 2.4* | |
| > 54.125 | 166/54 | 2.7 | *1.6 – 4.4* | | 1.7 | *1.1 – 2.9* | |
| Continuous variable | 498/105 | 1.04 | *1.03 – 1.06* | *< 0.001* | 1.03 | *1.01 – 1.04* | *< 0.001* |
| *Event Free Survival* | | | | | | | |
| Median | | | | *< 0.001* | | | *0.007* |
| ≤ 49.436 (*reference*) | 249/68 | 1 | - | | 1 | - | |
| > 49.436 | 249/115 | 1.8 | *1.4 – 2.5* | | 1.5 | *1.1 – 2.1* | |
| Tertiles | | | | *< 0.001* | | | *0.008* |
| ≤ 44.612 (*reference*) | 166/44 | 1 | - | | 1 | - | |
| 44.612 – 54.125 | 166/53 | 1.2 | *0.79 – 1.8* | | 1.1 | *0.76 – 1.7* | |
| > 54.125 | 166/86 | 2.2 | *1.6 – 3.2* | | 1.6 | *1.1 – 2.4* | |
| Continuous variable | 498/183 | 1.04 | *1.02 – 1.05* | *< 0.001* | 1.02 | *1.01 – 1.03* | *< 0.001* |
